# Tobacco- and alcohol-attributable burden of early-onset lip, oral cavity, and pharyngeal cancer in 204 countries and territories from 1990 to 2019, with projections to 2040

**DOI:** 10.3389/fonc.2024.1429972

**Published:** 2024-10-10

**Authors:** Xingzhu Dai, Yuanhao Liang

**Affiliations:** ^1^ Department of Stomatology, Guangdong Provincial People’s Hospital (Guangdong Academy of Medical Sciences), Southern Medical University, Guangzhou, China; ^2^ Clinical Experimental Center, Jiangmen Key Laboratory of Clinical Biobanks and Translational Research, Jiangmen Central Hospital, Jiangmen, China

**Keywords:** early-onset, lip and oral cavity cancer, other pharyngeal cancer, burden of disease, risk factors, estimated annual percentage change

## Abstract

**Background:**

Rising trends in early-onset Lip and oral cavity cancer (LOC) and Other pharyngeal cancer (OPC) burden had been observed. This study aimed to evaluate the burdens of LOC and OPC attributable to tobacco and alcohol in young adults aged 15-49 years from 1990 to 2040.

**Methods:**

Tobacco- and alcohol-attributable death and disability-adjusted life years (DALYs) for LOC and OPC and the corresponding population-attributable fraction were obtained from Global Burden of Disease Study 2019 for individuals aged 15-49 years. Estimated annual percent change was calculated to quantify the temporal trend of disease burden between 1990 and 2019. The Bayesian age-period-cohort model was used to predict the age-standardized mortality rate from 2020 to 2040.

**Results:**

In 2019, an estimated 16,887 deaths and 799,495 DALYs for tobacco- and alcohol-attributable early-onset LOC, and 8,402 deaths and 386,418 DALYs for early-onset OPC attributable to tobacco and alcohol were reported globally. Despite the global decrease in age-standardized mortality and DALYs rates of tobacco- and alcohol-attributable LOC and OPC in young adults aged 15-49 years between 1990 and 2019, certain regions experienced increases, such as regions of Asia, Eastern Europe, and Western Sub-Saharan Africa. Moreover, a growing age-standardized mortality in individuals aged <34 years was found. The socio-demographic index level was positively associated with a faster reduction of early-onset LOC and OPC DALYs attributable to alcohol use and smoking, except for that due to chewing tobacco. Furthermore, projections have also indicated an expected increase in the age-standardized mortality for tobacco- and alcohol-attributable early-onset LOC and OPC.

**Conclusions:**

Significant regional and demographic disparities in tobacco and alcohol-related early-onset LOC and OPC burden and their attributable proportion highlight a need for tailored age- and region-appropriate interventions to reduce the future LOC and OPC burden among young adults.

## 1 Introduction

The Global Cancer Observatory (GLOBOCAN) 2020 estimated nearly 20 million newly diagnosed cancer cases and 10 million cancer-related deaths across the globe in 2020, with the global cancer burden projected to reach 28.4 million by 2040 (1). Lip and oral cavity cancer (LOC) and Other pharyngeal cancer (OPC) are relatively uncommon globally but contribute significantly to the cancer burden in specific regions such as South and Central Asia, Eastern and Western Europe, and Australasia (1–3). Notably, Lip, oral cavity, and pharyngeal (including nasopharynx) cancers are on the rise globally, with an expected increase of 62% from 529,500 cases in 2012 to 856,000 cases in 2035 (3). The UK has seen a substantial 18% increase in oral and oropharyngeal cancer incidence among males and a 30% increase among females from 1990 to 1999, mirroring similar upward trends observed in several developed countries over the past few decades (4–8). Furthermore, a study has reported a rising trend in the incidence of Lip, oral cavity, and pharyngeal (excluding nasopharynx) cancer worldwide from 1990 to 2017, particularly among females and in low- and middle-income countries (9). While LOC and OPC are typically associated with the elderly, a significant increase in incidence has been observed among young adults (<50 years old at diagnosis) (9–12). For instance, the cancer incidence rates for oral cavity and pharynx increased by 1.2% and 0.7% per year in male and female patients aged 20-49 years in the USA during 2011 to 2015 (13). Importantly, early-onset LOC and OPC have substantial economic and social impact and can result in a greater number of person-years of life lost compared to later-onset cancers (14). Reports indicate that younger patients exhibit more aggressive progression and a higher risk of local recurrence or regional/distant metastases compared to older patients (15–17). In summary, the evolving epidemiological patterns of LOC and OPC, along with the steady increase in early-onset cases, present a growing global public health challenge.

The majority of deaths and DALYs burden from LOC and OPC are attributable to potentially modifiable risk factors such as tobacco use and alcohol consumption, accounted for 62.2% [95% UI, 57.5%-66.5%] of all LOC deaths and 61.87% [95% UI, 56.18%-66.84%] of overall OPC deaths in 2019 globally (2). It was noteworthy that the risk-attributable mortality rate for LOC was rising among individuals under the age of 45, particularly in middle and middle-high socio-demographic index (SDI) areas (18). The detrimental effects of tobacco and alcohol use on treatment effectiveness and complications in head and neck cancer has been extensively researched (19, 20). In addition, these modifiable risk factors for early-onset LOC and OPC are also prevalent in other chronic diseases, indicating that controlling these factors can have broader benefits beyond just preventing LOC and OPC (21). These significant epidemiologic shifts seem to reflect changes in exposure to risk factors among younger people; therefore, tracking and monitoring regional and national discrepancies in early-onset LOC and OPC burden due to modifiable risk factors is crucial for tailored prevention strategies and prioritize resource allocation to reduce the future LOC and OPC burden worldwide.

Studies have documented the overall and risk-attributable burden and trend in LOC and OPC from 1990 to 2019 (2, 18), as well as global incidence and trends of oral and oropharynx cancer before the age of 45 years during 1975-2016 (11). No study has specifically described the global burden of early-onset LOC and OPC attributable to tobacco and alcohol and its secular trend, however, and the variations by gender and between regions or countries with different levels of socio-economic development. This lack of information might hinder the development of preventive strategies to address this issue at global, regional, and national levels. Therefore, our study aims to inform and underpin health policy by analyzing recent epidemiological data from the Global Burden of Diseases, Injuries, and Risk Factors (GBD) Study 2019. The current study describes the global epidemiological pattern of tobacco- and alcohol-attributable LOC and OPC burden in young adults aged 15-49 years and demonstrates the temporal trends and variations in specific geographic and demographic contexts.

## 2 Methods

### 2.1 Data source and data collection

The data in this report is from the publicly available GBD Study 2019, a collaborative project led by the Institute for Health Metrics and Evaluation (IHME). It provides estimations of region-, country-, age- and sex-specific incidence, prevalence, mortality, years of life lost (YLLs), years lived with disability (YLDs), and DALYs for 369 diseases and injuries, as well as 87 risk factors, across 204 countries and territories, from 1990 to 2019 (22, 23). We extracted annual data on risk-attributable LOC and OPC deaths and DALYs burden in young adults under 50 from 1990 to 2019 across the globe, 5 socio-demographic index quintiles, 21 GBD world regions, and 204 countries and territories using the Global Health Data Exchange (GHDx) query tool (http://ghdx.healthdata.org/gbd-results-tool) in the current study.

### 2.2 Case identification

Since there has been a lack of consensus on the cut-off values of age considered to define a young patient (24). Previous studies have applied a dichotomy at 40, 45, or 49 years, unavoidably resulting in varying results (11, 17, 25). In order to enable consistent analysis and interpretation of current evidence on early-onset LOC and OPC burden, the age range for young adults has been arbitrarily defined as 15-49 years in this study (26). The case definition for early-onset LOC and OPC were cancer case diagnosed in individuals aged 15 to 49 years. The list of International Classification of Diseases 9th revision (ICD-9) and 10th revision (ICD-10) codes mapped to the LOC and OPC in the GBD Study 2019 has been detailed in a previous study (2). LOC is defined as per the ICD-10 with diagnosis codes C00-C08, and OPC with codes C09-C10 and C12-C13. Nasopharyngeal cancer (codes C11) was excluded from this study because it is commonly linked to Epstein-Barr virus infection, whereas cancers that occur in other pharyngeal sites are often associated with tobacco use, alcohol intake, and HPV infection (3).

### 2.3 Modifiable risk factors for early-onset LOC and OPC

The GBD Study 2019 has identified three attributable risk factors (smoking, chewing tobacco, and alcohol use) for early-onset LOC and 2 risk factors (smoking and alcohol use) related to early-onset OPC (22). Definitions of exposure to these attributable risk factors are as follows: 1) Smoking was defined as current smokers (currently use any smoked tobacco product) and former smokers (quit using all smoked tobacco products for at least six months); 2) Chewing tobacco was defined as the current use of chewing tobacco, including local products such as betel quid with tobacco, at any frequency; 3) Alcohol use was defined as average daily alcohol consumption of pure alcohol in current drinkers who had consumed alcohol during the past 12 months.

### 2.4 Estimation of early-onset LOC and OPC burdens attributable to tobacco and alcohol

The relative risks for tobacco/alcohol−LOC/OPC pairs were estimated by prospective cohort studies and population-based case-control studies. Population attributable fraction (PAF) represents the proportion of early-onset LOC and OPC burdens that would decrease in a given population and time if their exposure to a risk factor had reduced to the theoretical minimum risk exposure level (TMREL) at which the minimum risk occurs (22). PAF were estimated under the comparative risk assessment framework for each individual risk factor using the intake of specific risk factor, the estimated relative risk, and the TMREL. The estimation of PAF for combinations of risk factors was corrected for overestimation of the combined effects by a mediation matrix, as the effect of an individual risk factor on disease can be mediated through the intermediate one (22). The attributable burden was calculated by multiplying the overall burden measure of early-onset LOC and OPC by the PAF.

### 2.5 Mortality and DALYs Estimates

LOC/OPC-specific mortality was estimated by Cause of Death Ensemble Model after adjusting for selected covariates, using input data collected from vital registration data and cancer registry data coded to ICD system or household mortality surveys known as verbal autopsy. Additionally, years lived with disability (YLDs) for LOC and OPC were calculated by multiplying the prevalence with the corresponding disability weights, where 0 means no health loss and 1 indicates health loss equivalent to death. Years of life lost (YLLs) was computed from the observed number of deaths from LOC and OPC multiplied by the global standard life expectancy at the age of death. DALYs were the sum of the corresponding YLDs and YLLs. The estimation methods of mortality and DALYs for LOC and OPC has been described in a previous study (2).

### 2.6 Summary exposure value of tobacco and alcohol

In the GBD Study 2019, summary exposure value (SEV) represents the relative risk-weighted prevalence of each individual attributable risk factor and is on a scale from 0 (when no excess risk for a population exists) to 100% (when the population is at the highest level of risk). The annualized rate of change (ARC) was used to measure the temporal variation of SEV of risk factors, on which a decline in SEV indicates reduced exposure to the risk factor in the population, and vice versa.

### 2.7 Socio-demographic index

The SDI metric is a composite measure of socio‐demographic development status strongly correlated with health outcomes. This summary indicator comprises three indices: 1) total fertility rate for those younger than 25 years of age; 2) mean education for those aged 15 years or older; and 3) lag-distributed income per capita. The composite SDI is calculated by rescaling these three indices for a given location-year to obtain the geometric mean. According to the calculated SDI score, regions and countries are classified into five distinct quintiles: high SDI (0.805-1), high-middle SDI (0.690-0.805), middle SDI (0.608-0.690), low-middle SDI (0.455-0.608), and low SDI (0-0.455) (23).

### 2.8 Statistical analysis

In this study, the age-standardized rate (per 10^5^ population), including age-standardized mortality rate (ASMR) and age-standardized DALYs rate (ASDR), was calculated using the following formula:


$$\text{ASR=}\frac{\sum_{i=1}^{A}a_{i}w_{i}}{\sum_{i=1}^{A}w_{i}}\times 100,000$$

where $a_{i}$ denotes the age-specific rate in the $i^{th}$ age subgroup and $w_{i}$ represents the number of individuals in the same age class of the GBD world age-standard population came from the GBD Study 2019 Population Estimates 1950-2019 (27). These age-standardized measures enable a comparable assessment of the tobacco- and alcohol-attributable early-onset LOC and OPC burden across different populations (28).

The estimated annual percentage changes (EAPCs) in ASMR, ASDR, and age-specific rate of mortality and DALYs were used to quantify the epidemic trends of tobacco- and alcohol-attributable early-onset LOC and OPC burden. EAPC was widely used to assess the temporal trend of disease burden (29). A regression line was fitted to the natural logarithm of the rates, denoted as y=α+βx+ϵ , where y=ln(ASR) , and x=calendar year . The EAPC was calculated as 100×(exp(β)−1) , and the corresponding 95% confidence interval (CI) could be obtained from the linear regression model.

In addition, Pearson correlation analysis was conducted to evaluate the relationship between ASMR and ASDR with SDI quintile, age-specific rate of mortality and DALYs with SDI quintile, EAPCs in ASMR and ASDR with SDI quintile, as well as PAF of risk factors with SDI quintile. Furthermore, the age-standardized mortality of tobacco- and alcohol-attributable early-onset LOC and OPC from 2020 to 2040 were projected by using the Bayesian age-period-cohort (BAPC) model integrating nested Laplace approximations (30). The global age-standard population came from World Standards database constructed by the World Health Organization (https://seer.cancer.gov/stdpopulations/world.who.html), and population forecast data was collected from the GBD Study 2019 Global Fertility, Mortality, Migration, and Population Forecasts 2017-2100 (31). The R package “BAPC” streamlines the implementation of the BAPC model, allowing for the generation of well-calibrated probabilistic forecasts with relatively narrow uncertainty ranges.

All statistics analysis and mapping were done with R software, version 4.1.0 (R Foundation for Statistical Computing). A *P* < 0.05 was regarded as significant.

## 3 Results

### 3.1 The global burden of tobacco- and alcohol-attributable early-onset LOC and OPC in 2019

**Figure 1:**
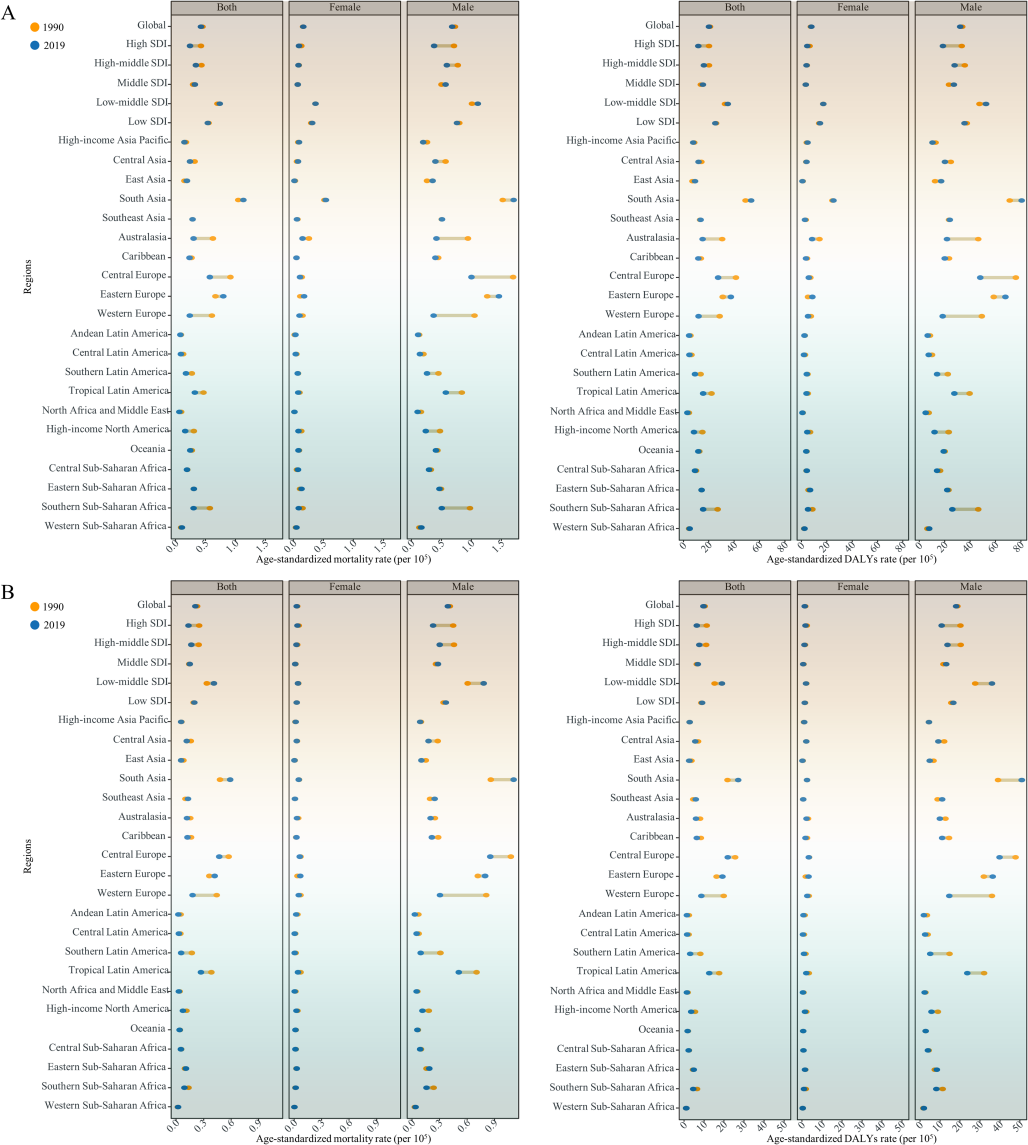
Age-standardized mortality and DALYs rates of modifiable risk-associated early-onset **(A)** LOC and **(B)** OPC among males and females and both sexes combined in 1990 and 2019, globally and for 5 Socio-demographic Index (SDI) quintiles and 21 GBD regions. DALYs, Disability adjusted life years; LOC, Lip and oral cavity cancer; OPC, Other pharyngeal cancer.

**Figure 2:**
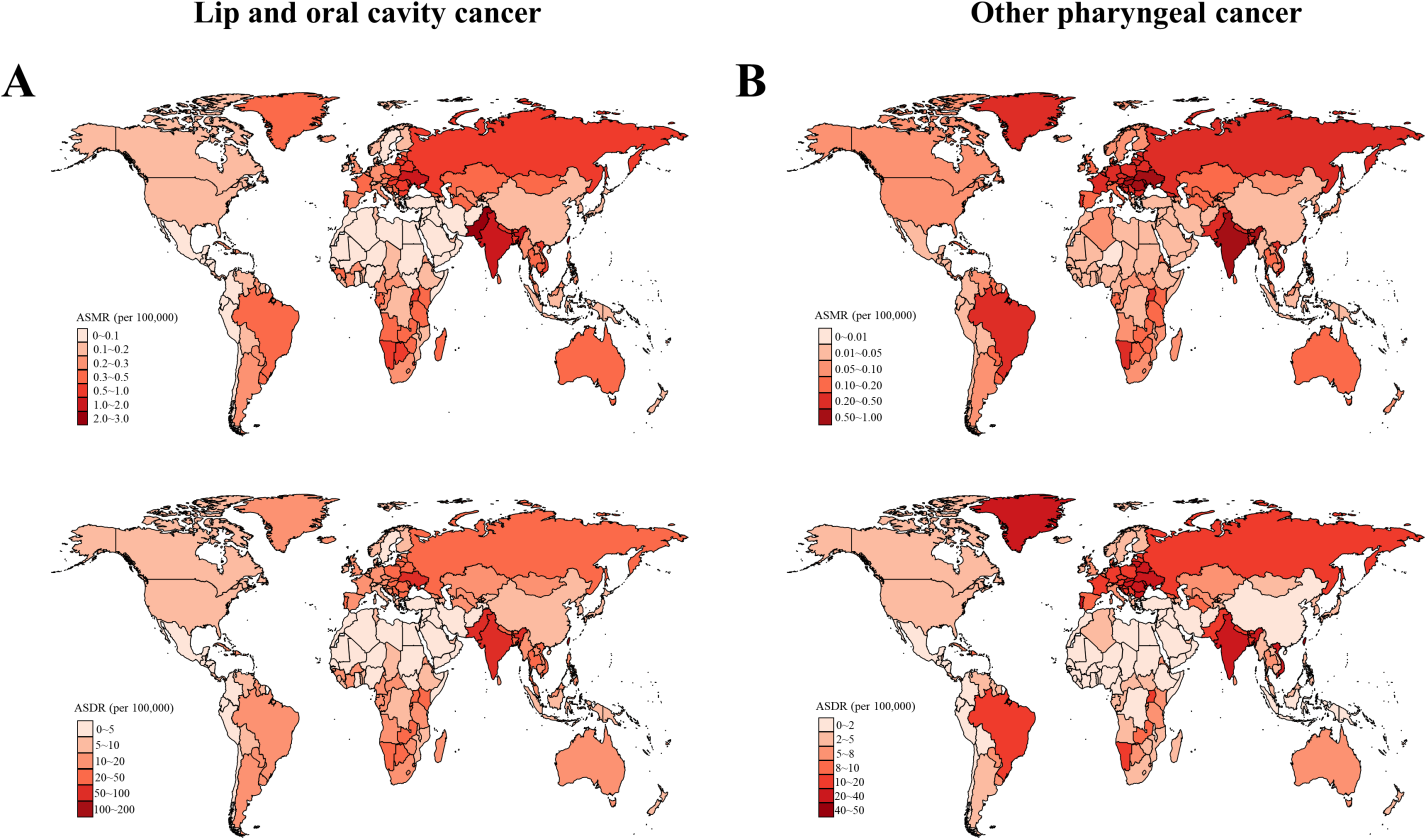
Age-standardized mortality and DALYs rates of **(A)** early-onset lip and oral cavity cancer (LOC) and **(B)** early-onset other pharyngeal cancer (OPC) due to modifiable risks for both sexes combined in 2019, by country. DALYs, Disability adjusted life years.

Globally, an estimated 16,887 (95% uncertainty interval [UI]: 14,245 to 19,688) deaths from tobacco- and alcohol-attributable LOC among young adults aged 15-49 years were reported in 2019. The global crude mortality rate and ASMR were 0.43 (95% UI: 0.36 to 0.5) and 0.42 (95% UI: 0.42 to 0.43) per 10^5^, respectively (**Supplementary Tables S1**, **S2**). The number of DALYs due to tobacco and alcohol-related early-onset LOC globally in 2019 was 799,495 (95% UI: 672,639 to 930,964), with a crude DALYs rate of 20.32 (95% UI: 17.09 to 23.66) per 10^5^ and an ASDR of 20 (95% UI: 19.95 to 20.04) per 10^5^ (**Supplementary Tables S2**, **S3**). In 2019, by SDI category, the low-middle SDI regions had the highest ASMR and ASDR for tobacco- and alcohol-attributable early-onset LOC (0.74 [95% UI: 0.72 to 0.75] deaths and 34.76 [95% UI: 34.64 to 34.89] DALYs per 10^5^, respectively). Among 21 GBD regions, South Asia had the highest ASMR (1.13 [95% UI:1.11 to 1.15]) and ASDR (53.26 [95% UI: 53.11 to 53.42]) of tobacco- and alcohol-attributable early-onset LOC for both sexes combined, followed by Eastern Europe and Central Europe (**Figure 1A** and **Supplementary Table S2**). At the national level, the greatest burden of early-onset LOC attributable to tobacco and alcohol was seen in Palau, followed by Taiwan (Province of China) and Pakistan (**Figure 2A**). According to the country and territory analysis, no significant association between ASMR and ASDR rate with SDI was found (**Supplementary Figure S1A**), although there was a weak positive correlation between age-specific rate of mortality and DALYs with SDI (**Supplementary Figure S2A**).

In 2019, tobacco- and alcohol-attributable early-onset OPC was responsible for 8,402 (95% UI: 6,909 to 9,946) deaths and 386,418 (95% UI: 316,466 to 458,971) DALYs globally (**Supplementary Tables S4**, **S5**). The global crude mortality rate, ASMR, crude DALYs rate, and ASDR per 10^5^ for early-onset OPC attributable to tobacco and alcohol were 0.21 (95% UI: 0.18 to 0.25), 0.21 (95% UI: 0.21 to 0.21), 9.82 (95% UI: 8.04 to 11.66), and 9.65 (95% UI: 9.62 to 9.68), respectively (**Supplementary Tables S4**, **S6**). The highest ASMR (0.41 [95% UI: 0.4-0.42]) and ASDR (18.9 ([95% UI: 18.81-18.99]) for tobacco- and alcohol-attributable early-onset OPC were also reported in the low-middle SDI quintile (**Figure 1B** and **Supplementary Table S6**). At the GBD regional level, the South Asia had the highest ASMR and ASDR for tobacco- and alcohol-attributable early-onset OPC (0.59 [95% UI: 0.57 to 0.6] deaths and 26.96 [95% UI: 26.85 to 27.07] DALYs per 10^5^, respectively), followed by Central Europe and Eastern Europe (**Figure 1B** and **Supplementary Table S6**). According to the distribution of national-level ASMR and ASDR for early-onset OPC attributable to tobacco and alcohol in 2019, Taiwan (Province of China), Slovakia, and Romania were the top 3 countries and territories with the greatest burden (**Figure 2B**). Moreover, countries with high-middle and high SDI generally had a higher ASMR, ASDR, and crude mortality and DALYs rates for tobacco- and alcohol-attributable early-onset OPC (all models, *P* < 0.001; **Supplementary Figures S1B** and **S2B**).

The ASMR and ASDR for tobacco- and alcohol-attributable early-onset LOC and OPC varied by sex and age. These indicators were higher in males than in females globally and in all 5 SDI categories and 21 GBD regions (**Supplementary Tables S2** and **S6**). Both the ASMR and crude mortality rate for tobacco- and alcohol-attributable early-onset LOC and OPC were higher in males than in females across the globe in 2019, with the highest male-to-female ratio of mortality observed in East Asia (**Supplementary Figure S3**). In addition, the age-specific mortality and DALYs rates for tobacco- and alcohol-attributable early-onset LOC and OPC increased with age (**Supplementary Figures S4**, **S5**). Further analysis of age distribution revealed that the differences in the mortality and DALYs between young male adults and young female adults were mainly due to variances in the older age groups (35-39, 40-44, and 45-49 years). Notably, this gap narrowed over time in countries with high- and high-middle SDI (**Supplementary Figures S6**, **S7**).

### 3.2 Temporal trend of tobacco- and alcohol-attributable early-onset LOC from 1990 to 2019

**Figure 3:**
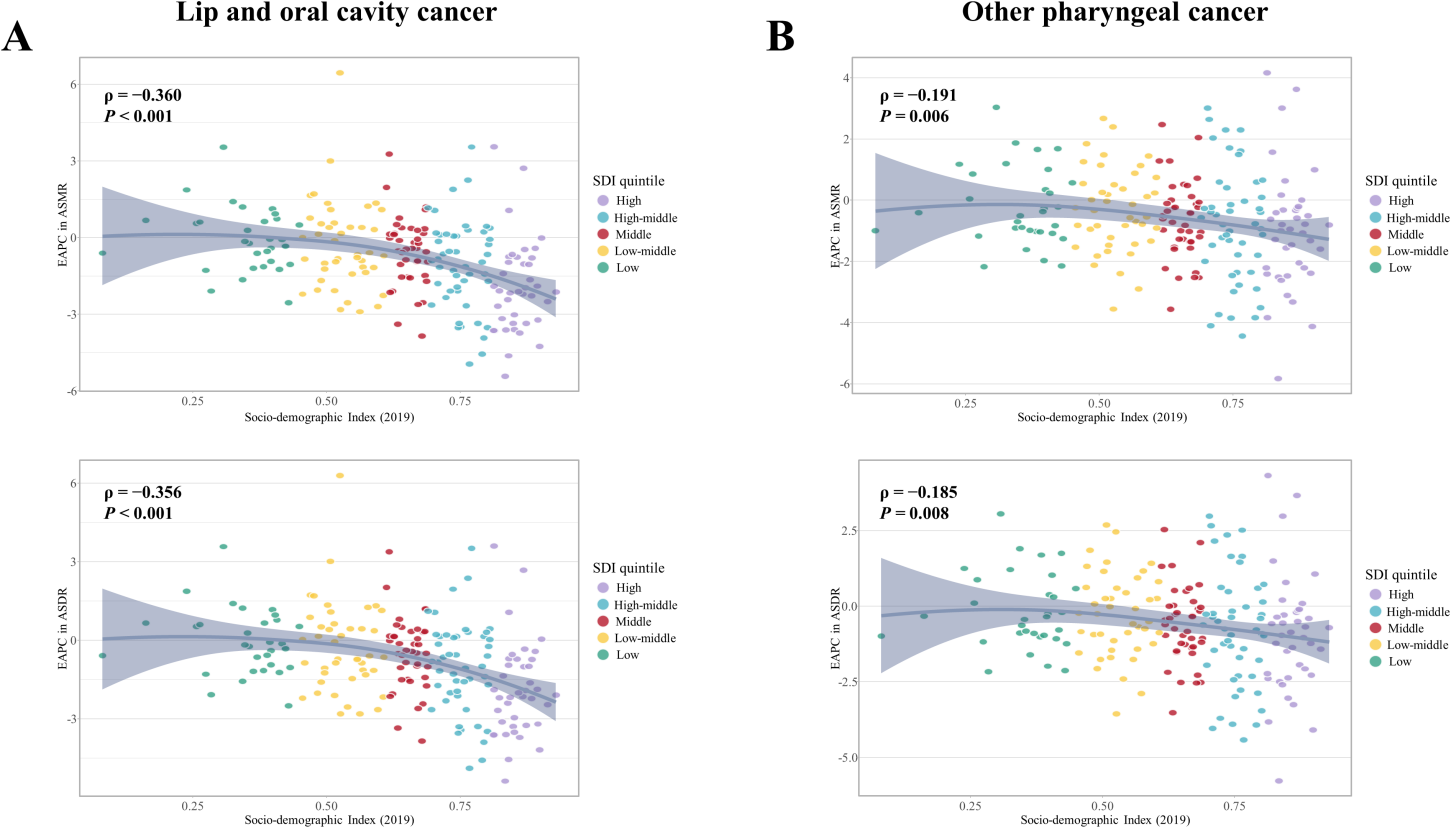
Association between the EAPC in ASMR and ASDR for **(A)** early-onset LOC and **(B)** early-onset OPC attributable to modifiable risks from 1990 to 2019, with Socio-demographic Index (SDI) in 2019. EAPC, Estimated annual percent changes; ASMR, Age-standardized mortality rate; ASDR, Age-standardized disability adjusted life years rate; LOC, Lip and oral cavity cancer; OPC, Other pharyngeal cancer.

From 1990 to 2019, the number of deaths and DALYs due to tobacco- and alcohol-attributable early-onset LOC, as well as the corresponding crude rates, increased globally for both sexes, with a greater increase in females than in males (**Supplementary Figure S8A** and **S8C**). Over the same period, the global ASMR and ASDR showed a decreasing trend for both sexes, with faster average annual percentage decreases observed in males (**Supplementary Table S2**). Our study has found that the SDI level was positively associated with faster reduction in ASMR and ASDR for early-onset LOC attributable to tobacco and alcohol (**Figure 3A**). Generally, older age groups (40-44 and 45-49 years) were associated with faster reductions or slower increases in the mortality and DALYs rate across all 5 SDI regions, while the 25-29 age group showed the opposite trend (**Supplementary Figure S4** and **Supplementary Table S7**).

When categorized by GBD regions, most regions experienced a decline in ASMR and ASDR of tobacco- and alcohol-attributable early-onset LOC whereas several regions (e.g., East and South Asia, Western Sub-Saharan Africa, and Eastern Europe) showed an increasing trend (**Figure 1A**). The largest increases in ASMR among female individuals between 1990 and 2019 occurred in Andean Latin America (EAPC = 1.74 [95% CI: 1.63 to 1.85]), Eastern Europe (EAPC = 1.66 [95% CI: 1.46 to 1.85]), and Central Sub-Saharan Africa (EAPC = 1 [95% CI: 0.97 to 1.03]). In comparison, East Asia (EAPC = 1.57 [95% CI: 1.34 to 1.8]) and Western Sub-Saharan Africa (EAPC = 0.72 [95% CI: 0.67 to 0.77]) had the fastest increases in ASMR among males (**Supplementary Table S2**). Moreover, the largest increase in ASDR was found in Eastern Europe (EAPC = 1.65 [95% CI: 1.29 to 2.01]) for females and East Asia (EAPC = 1.55 [95% CI: 1.33 to1.77]) for males (**Supplementary Table S2**). At the country level, Cabo Verde and Turkmenistan recorded the largest increases in ASMR and ASDR for males and females, respectively (**Supplementary Figure S9**).

### 3.3 Temporal trend of tobacco- and alcohol-attributable early-onset OPC from 1990 to 2019

Global deaths due to tobacco- and alcohol-attributable early-onset OPC rose from 4,866 (4,254 to 5,488) in 1990 to 8,402 (95% UI:6,909 to 9,946) in 2019 (**Supplementary Table S4**). Tobacco- and alcohol-attributable DALYs for early-onset OPC increased by 73.9% between 1990 and 2019 (**Supplementary Table S5**). During the study period, the ASMR and ASDR decreased by an average of 0.56% (95% CI: −0.64 to −0.48) and 0.49% (95% CI: −0.58 to −0.4) per year globally, respectively. Faster reductions in the ASMR and ASDR for early-onset OPC attributable to tobacco and alcohol were observed in females compared to males (**Supplementary Table S6**). In addition, the high and high-middle SDI quintiles showed a decreasing trend in the ASMR and ASDR for both sexes, whereas the largest increases of them were found in the low-middle SDI quintile (**Figure 1B**). The annual percent changes of ASMR (ρ = −0.191, *P* = 0.006) and ASDR (ρ = −0.185, *P* = 0.008) illustrated negative correlation with SDI level in 2019 (**Figure 3B**).

From 1990 to 2019, in term of GBD regions, the largest increases in the ASMR and ASDR for tobacco- and alcohol-attributable early-onset OPC among males were found in South and Southeast Asia (**Figure 1B** and **Supplementary Table S6**). Regions of Eastern Europe (EAPC = 2.15 [95% CI: 2.12 to 2.18]) and Western Sub-Saharan Africa (EAPC = 1.96 [95% CI: 1.78 to 2.14]) had the largest increase in ASMR in females. Eastern Europe (EAPC = 1.95 [95% CI: 1.68 to 2.21]) also had the fastest increase in ASDR in female individuals, however, the second fastest ASDR increase occurred in Oceania (EAPC = 0.4 [95% CI: 0.35 to 0.44]; **Figure 1B** and **Supplementary Table S6**). At the country level, for males, Guam had the largest increase in both ASMR (EAPC = 4.94 [95% CI: 4.36 to 5.53]) and ASDR (EAPC = 5.08 [95% CI: 4.48 to 5.69]), while France reported the largest reduction in both ASMR (EAPC = −6.24 [95% CI: −6.72 to −5.76]) and ASDR (EAPC = −6.2 [95% CI: −6.68 to −5.73]). For female individuals, Vietnam and Saint Kitts and Nevis were the countries with the largest increases and the fastest reductions in both ASMR and ASDR, respectively (**Supplementary Figure S10**). Similarly, faster reductions or slower increases in the mortality and DALYs rate were observed in older age (40-44 and 45-49 years), and the opposite in the 20-24 age group (**Supplementary Figure S5** and **Supplementary Table S8**).

### 3.4 PAF of individual risk factor-attributable early-onset LOC

In 2019, the proportions of DALYs due to smoking-, chewing tobacco-, and alcohol use-attributable early-onset LOC was 17.11%, 18.75%, and 30.07% globally, respectively (**Supplementary Figure S11A**). The proportion of early-onset LOC deaths attributable to tobacco and alcohol mirrored the same pattern of the tobacco- and alcohol-attributable proportion of DALYs burden. From 1990 to 2019, alcohol use had the highest PAF of DALYs in high, high-middle, and middle SDI quintiles, whereas chewing tobacco accounted for the highest PAF of DALYs in low-middle and low SDI quintiles (**Supplementary Figure S12A**). The proportion of early-onset LOC DALYs attributable to smoking declined in all 5 SDI quintiles between 1990 and 2019. The proportion of early-onset LOC DALYs attributable to chewing tobacco either increased or remained stable over time across all SDI settings, except for the low SDI quintile. However, a decrease in the proportion of early-onset LOC DALYs attributable to alcohol consumption was found only in the high SDI quintile (**Supplementary Figure S12A**). Moreover, the SDI level was positively associated with a higher proportion of tobacco- and alcohol-attributable early-onset LOC DALYs for modifiable risks combined, alcohol use, and smoking, except for chewing tobacco, which showed a negative correlation (**Supplementary Figure S13A**). Over this period, the proportion of early-onset LOC DALYs due to chewing tobacco increased significantly in countries with relative lower chewing tobacco-attributable early-onset LOC burden (**Supplementary Figure S14A**).

By GBD regions and risk factors, the region in which the highest proportion of early-onset LOC deaths and DALYs burden attributable to smoking, chewing tobacco, and alcohol use in 2019 for both sexes combined was Eastern Europe, South Asia, and Eastern Europe, respectively (**Supplementary Figure S11A**). Higher proportions of early-onset LOC deaths and DALYs burden attributable to smoking and alcohol use were observed in males compared to females across 21 GBD regions. However, the chewing tobacco-attributable proportions was higher in females than in males in most GBD regions (except for regions of Central Asia, North Africa and Middle East, and High-income North America), with this burden concentrated in South and Southeast Asia, Oceania, and regions of sub-Saharan Africa (**Supplementary Figure S15A**).

Tobacco- and alcohol-attributable proportion of early-onset LOC DALYs increased with age (**Supplementary Table S9**). In 2019, across all age group of young adults, alcohol consumption was the primary attributable risk factor globally and in the high, high-middle, and middle SDI quintiles, whereas chewing tobacco responsible for the highest proportion of early-onset LOC DALYs burden in low-middle and low quintiles (**Supplementary Table S9**).

### 3.5 PAF of individual risk factor-attributable early-onset OPC

In 2019, smoking and alcohol consumption were responsible for 27.80% and 32.79% of early-onset OPC DALYs globally, respectively (**Supplementary Figure S11B**). Alcohol consumption accounted for a larger proportion of early-onset OPC DALYs than smoking in high and high-middle SDI quintiles from 1990 to 2019. However, the primary contributor to early-onset OPC DALYs shifted from smoking in 1990 to alcohol use in 2019 among middle, low-middle, and low SDI regions (**Supplementary Figure S12B**). Between 1990 and 2019, all 5 SDI quintiles showed a decreasing trend in the proportion of early-onset OPC DALYs attributable to smoking, while the proportion of early-onset OPC DALYs attributable to alcohol consumption only declined in the high SDI quintile (**Supplementary Figure S12B**). The largest increase in the proportion of early-onset LOC and OPC due to alcohol use and smoking was observed in Mozambique and Afghanistan, respectively (**Supplementary Figure S14**). Additionally, there was a positive association between SDI level and the proportion of early-onset OPC DALYs attributable to tobacco and alcohol combined, alcohol consumption, and smoking (**Supplementary Figure S13B**).

The highest PAF of early-onset OPC burden attributable to smoking and alcohol use, in both sexes combined, was reported in Eastern Europe (**Supplementary Figure S11B**). The proportion of early-onset OPC burden attributable to smoking and alcohol use was higher in males than in females globally and in all 21 GBD regions (**Supplementary Figure S15B**). The proportions of early-onset OPC DALYs attributable to tobacco and alcohol combined, alcohol consumption, and smoking all increased with age (**Supplementary Table S10**). Notably, contributions to early-onset OPC DALYs were greater for alcohol consumption than smoking among individuals in the younger age group of under 45 years old both globally and in all 5 SDI quintiles (**Supplementary Table S10**).

### 3.6 The summary exposure value for tobacco and alcohol

In 2019, the SEVs of tobacco and alcohol were higher in males aged 15-49 years versus females at the same age group globally (11.1 vs 3.3 for alcohol use; 13.9 vs 2.7 for smoking; 7.5 vs 3 for chewing tobacco; **Supplementary Tables S11**, **S13**). Globally, the SEV of alcohol use increased by an annual 0.04% from 1990 to 2019 in young male adults but decreased by −0.13% per year in young female adults (**Supplementary Table S11**). The high SDI quintile had the highest SEV of alcohol use in 2019 and the largest decrease in the SEV of alcohol use over the past 30 years, for both sexes (**Supplementary Table S11**). When classified by GBD areas, Australasia and regions of Europe (Central, Eastern, and Western) have the highest SEV of alcohol use for both sexes. Additionally, the fastest increases in the SEV of alcohol use between 1990 and 2019 were found in East, South, and Southeast Asia for both sexes (**Supplementary Table S11**).

The SEV of smoking showed a decreasing trend for both young male adults (average change −0.25% per year [95% UI: −0.27 to −0.23]) and young female adults (average change −0.44% per year [95% UI: −0.47 to −0.42]) globally (**Supplementary Table S12**). From 1990 to 2019, the SEV of smoking decreased across all 5 SDI quintiles and the majority of GBD regions, with the largest decrease recorded in the high SDI quintile and Tropical Latin America, respectively, for both sexes (**Supplementary Table S12**). In 2019, in both sexes, compared with regions with a low SDI, the SEV of smoking in regions with a high SDI was higher, and the highest SEV of smoking was found in regions of Europe (**Supplementary Table S12**).

During the past three decades, there was a slight increase in the SEV of chewing tobacco in both sexes (average change 0.09% and 0.11% per year for young male adults and young female adults, respectively; **Supplementary Table S13**). Different from the SEV of alcohol consumption and smoking, regions with a low SDI had the higher SEV of chewing tobacco. Moreover, the SEV of chewing tobacco decreased in low-middle and low SDI quintiles but increased in high, high-middle, and middle SDI quintiles (**Supplementary Table S13**). Notably, although a decreasing trend was seen, the SEV of chewing tobacco in South Asia was far more than the rest GBD regions. Between 1990 to 2019, Central Asia and regions of High-income Asia Pacific experienced the fastest increase in the SEV of chewing tobacco for young male adults and young female adults, respectively (**Supplementary Table S13**).

When stratified by age group, the SEV of these risk factors in people aged 15-49 years increased with age (in both sexes combined) globally and in all 5 SDI quintiles (**Supplementary Table S14**). The SEVs of alcohol use and smoking decreased in every age subgroup of young adults globally, while the decreasing trend in the SEV of chewing tobacco was only observed in the 45-49 age group (**Supplementary Table S14**). The SEV of alcohol consumption increased in all age groups in the middle, low-middle, and low SDI quintiles and the younger age group (15-19 years, 20-24 years, and 25-49 years) in the high-middle SDI quintile (**Supplementary Table S14**). The SEV of smoking decreased in all age groups among all 5 SDI quintiles; however, the high, high-middle, and middle SDI quintiles witnessed an increasing trend in the SEV of chewing tobacco across all age groups (**Supplementary Table S14**).

**Figure 4:**
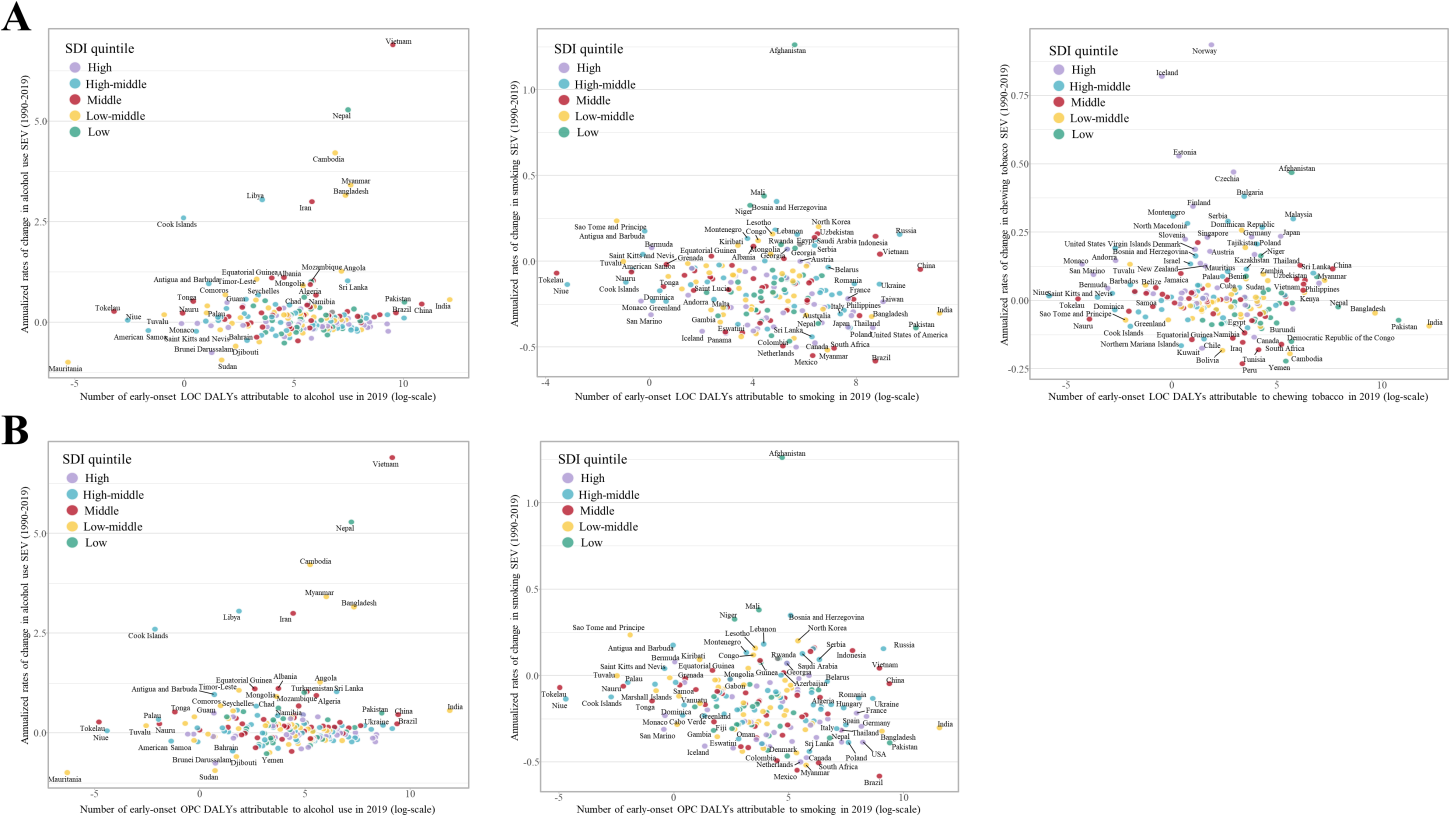
Comparison of annualized rate of change in risk exposure measured by summary exposure values (SEVs) for population aged 15-49 years (in both sexes combined) from 1990 to 2019 with the corresponding tobacco- and alcohol-attributable disability adjusted life years (DALYs) for **(A)** early-onset lip and oral cavity cancer (LOC) and **(B)** early-onset other pharyngeal cancer (OPC) in 2019, by country. Countries with different levels of socio-economic development are indicated by different colors.

At the national level, Bulgaria, Estonia, and Palau had the highest SEV of alcohol use (22.1 [95% UI: 18.5 to 25.7]), smoking (20.6 [95% UI: 15 to 27.6]), and chewing tobacco (29 [95% UI: 24.2 to 34.1]), respectively (**Supplementary Figure S16**). The greatest increase in national-level SEV of alcohol use, smoking, and chewing tobacco in both sexes combined from 1990 to 2019 was found in Vietnam, Afghanistan, and Norway, respectively (**Figure 4** and **Supplementary Figures S17**–**S19**). However, Albania, rather than Afghanistan, had the fastest increase in the SEV of smoking in young female adults (**Supplementary Figure S18**). Comparisons of annualized rate of changes (ACRs) in the SEV of these risk factors in young adults between 1990 and 2019 with the corresponding tobacco- and alcohol-attributable DALYs for early-onset LOC and OPC in 2019 shows that countries with lower early-onset LOC and OPC burden attributable to alcohol use were mainly those with larger cumulative improvements in exposure to alcohol consumption; however, this was the opposite when considering tobacco consumption (**Figure 4** and **Supplementary Figures S17**–**S19**).

### 3.7 Projections of ASMR for tobacco- and alcohol-attributable early-onset LOC and OPC through 2040

**Figure 5:**
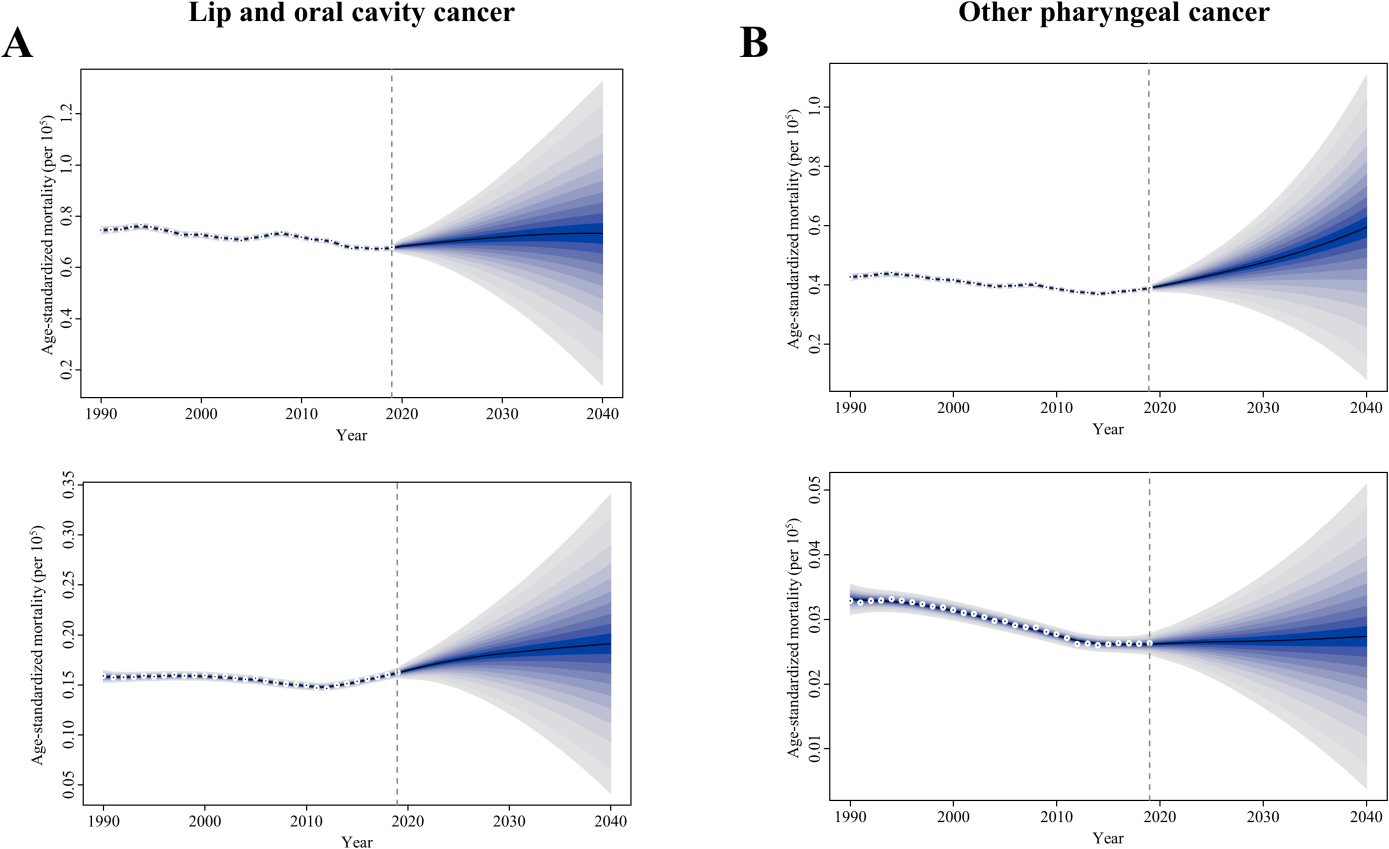
Trends of age-standardized mortality rate in the tobacco- and alcohol-attributable **(A)** early-onset lip and oral cavity cancer (LOC) and **(B)** early-onset other pharyngeal cancer (OPC), for males (upper) and females (bottom): observed rate (1990–2019) and predicted rates (2020–2040). The blue region in shows the upper and lower limits of the 95% uncertainty interval (UI).

The prediction and the changing trends of ASMR for tobacco- and alcohol-attributable early-onset LOC and OPC by sex were depicted by sex in **Figure 5**. In general, the ASMR of LOC attributable to tobacco and alcohol in young adults showed a slight increasing trend for both sexes globally from 2020 to 2040. By 2040, the globally projected ASMR of tobacco- and alcohol-attributable early-onset LOC is 0.73 deaths per 10^5^ population for males and 0.19 deaths per 10^5^ population for females (**Figure 5A**). When classified by attributable risk factors, the ASMR of smoking-attributable early-onset LOC is projected to decline to 0.21 and 0.01 deaths per 10^5^ population for males and females globally in 2040, respectively (**Supplementary Figure S20A**). From 2020 to 2040, the ASMR of early-onset LOC attributable to chewing tobacco tends to trend upward for both sexes, with a projection of 0.21 and 0.15 deaths per 10^5^ population for males and females globally in 2040, respectively (**Supplementary Figure S20B**). Additionally, the projected ASMR of alcohol consumption-attributable early-onset LOC remains stable globally for both sexes, reaching 0.04 and 0.46 deaths per 10^5^ population for males and females by 2040, respectively (**Supplementary Figure S20C**).

Concerning risk-related early-onset OPC, the ASMR is on the rise in males but remains stable in females between 2020 and 2040. It is projected that globally in 2040, the ASMR of risk-related OPC is 0.59 and 0.03 deaths per 10^5^ population among young male adults and young female adults, respectively (**Figure 5B**). Furthermore, from 2020 to 2040, globally projected increases were seen in the ASMR of smoking- and alcohol use-related early-onset OPC in males (0.27 and 0.40 deaths per 10^5^ population attributable to smoking and alcohol consumption in 2040, respectively; **Supplementary Figure S21**). However, by 2040, the ASMR of early-onset OPC attributable to smoking and alcohol consumption in females declines to 0.01 deaths and increases 0.02 deaths per 10^5^ population, respectively (**Supplementary Figure S21**).

## 4 Discussion

In this study, we conducted a comprehensive evaluation of spatial patterns and temporal trends of lip, oral cavity, and other pharyngeal cancer deaths and DALYs due to modifiable risks among young adults under 50 years old across 204 countries and territories globally from 1990 to 2019. Deaths and DALYs of tobacco- and alcohol-attributable LOC and OPC have increased substantially and accounted for a significant proportion of the overall LOC and OPC burden among young adults, underscoring the necessity to diminish the impact of preventable early-onset LOC and OPC burden worldwide. The ASMR and ASDR of tobacco- and alcohol-attributable early-onset LOC and OPC have decreased over the past 30 years for both sexes globally; however, there were geographical and demographic disparities. From 1990 to 2019, increases occurred in several regions, including regions of Asia (East, South, and Southeast), Eastern Europe, and Western Sub-Saharan Africa. In 2019, South Asia, Central Europe, and Eastern Europe continue to be the regions with the largest tobacco- and alcohol-attributable burden for early-onset LOC and OPC in terms of the age-standardized death and DALY rates among young adults. These findings are in accordance with the high estimated age-standardized LOC and OPC incidence rate in these regions (1, 2). Risks-related burden of early-onset LOC and OPC varied across the globe, which may be ascribed to the regional variation in exposure level to attributable risk factors and accessibility to treatment and healthcare (32–37). Furthermore, we projected the global ASMR for tobacco- and alcohol-attributable early-onset LOC and OPC up to 2040, indicating a rising trajectory from 2020 to 2040. To our knowledge, this is the first analysis to describe the global burden for risk-related early-onset LOC and OPC, based on the GBD Study 2019.

From 1990 to 2019, the crude mortality rate for tobacco- and alcohol-attributable LOC and OPC increased annually by 0.47% (95% CI: 0.37 to 0.56) and 0.3% (95% CI: 0.19 to 0.41) among young adults globally, respectively. Particularly, the increasing age-specific mortality rate of early-onset LOC and OPC in the younger age brackets of 20-24 and 25-29 made the largest contribution to the overall worldwide increase, suggesting the rising trend in risk-related early-onset LOC and OPC mortality in younger generation. These findings are in accordance with previous reports (12, 18), confirming an increasing trends in mortality and DALYs of overall and tobacco- and alcohol-attributable oral cancer have been observed in patients aged below 45 globally between 1990 to 2019. However, upon adjusting for population age distribution, the ASMRs for risk-associated early-onset LOC and OPC showed a downward trend, indicating the remarkable progress in the prevention, detection and treatment of LOC and OPC in recent decades (25, 32, 38–42). When further stratifying young adults into the 15-34 years and 35-49 years groups, we found a significant increase in the ASMR among individuals aged 15-34 years and a decline in the age group 35-49 years from 1990 to 2019 (**Supplementary Figure S22**). This finding revealed the important increasing trend in the younger age group, which would otherwise have been masked by the much higher burden in the older age subgroup. Therefore, interventions to prevent and control LOC and OPC in the population aged 15-34 years are a public health priority. Notably, according to estimates from the age-period-cohort analysis for the overall or tobacco-attributable oral cancer burden for all ages, the cohort effect varied by location and sex. In the continuous birth cohort in China, the relative risk (RR) of mortality for oral cancer increased steadily in males but decreased in females (43). However, for the most countries in Europe, the later cohort exhibits lower RR of deaths for oral cancer than the early cohort (44). Additionally, males born in later cohort have a higher RR of mortality for tobacco-attributable oral cancer in China and India, whereas later cohort have a lower RR of mortality in the USA (45). Nevertheless, the cohort effect for tobacco- and alcohol-attributable LOC and OPC among young adults remained to be elucidated.

In the present study, the favorable trends in tobacco- and alcohol-attributable burden of early-onset LOC and OPC have been mainly driven by sharp declines in prevalence of smoking among young people globally (34). Meanwhile, a significant increase was observed in South Asia, Eastern Europe, and Western Sub-Saharan Africa. Similarly, many studies of the aforementioned regions have documented increasing trends in LOC and OPC burden among young adults. Epidemiological studies in India showed that notification rates for new oral cancer cases in young adults between the ages of 15-44 years increased significantly (46, 47). Garavello and colleagues have also reported a rising oral and pharyngeal mortality among the population aged 35-64 years in some central and eastern European countries during 1975-2004 (48). Increasing trend of tobacco- and alcohol-attributable LOC and OPC mortality and DALYs among young adults can likely be attributed to the patterns in smokeless tobacco products or alcohol use, such as the growing prevalence of chewing tobacco in South Asia, especially common among those aged 15-19 years (33, 49), as well as increased alcohol consumption in South Asia and many Eastern European countries over recent decades (50). The current study had also found that in countries with a high SDI, alcohol use still accounted for the highest proportion of the burden for early-onset LOC and OPC, but the attributable fraction showed a downward trend, as well as the exposure level measured by the summary exposure value (SEV). Instead, the consumption of smokeless tobacco products is becoming a cause of concern and require special attention; however, there was less widespread implementation of the WHO Framework Convention on Tobacco Control (FCTC) measures on smokeless tobacco products (51). Therefore, policymakers must be proactively aware of these shifts and established more robust tobacco control measures to curb this epidemic more effectively.

Moreover, socioeconomic status was closely associated with the risk and outcome of LOC and OPC (32, 52). Similar patterns of change were found in the present study; the SDI level was positively associated with faster reduction in ASMR and ASDR for early-onset LOC and OPC attributable to tobacco and alcohol. One possible explanation is that regions with low SDI may have relatively higher exposure to risk factors and lower levels of received health care. There is evidence that, starting from the 20th century, the burden of unhealthy lifestyle had shifted from high socioeconomic groups towards the disadvantaged one (53). Although the period during which young patients indulged in the risk factors of tobacco and alcohol is significantly shorter than that of the older age group, the risk increases not only monotonically with the duration but also with the level of exposure. For example, the alcohol use-associated health risks were greater for younger populations than older populations, and heavy alcohol intake was particularly concentrated in males aged 15-39 (36). In addition, the consideration of LOC and POC is often overlooked for younger patients, potentially resulting in delayed diagnosis, treatment, and a compromised prognosis (54). The rising trend of risk-associated LOC and OPC burden in younger individuals (15-34 years) indicates a necessity to tackle age-related inequalities in their diagnosis.

The results of this study show that the burden of risk-related LOC and OPC were higher in males than in females for the truncated age group (15-49 years), which showed a similar pattern to that seen for all ages (2). Globally, the decline in age-standardized tobacco- and alcohol-attributable death and DALY rates for early-onset LOC was faster in males, while for early-onset OPC, it was more pronounced in females. The reason for this disparity is largely influenced by varying prevalence of exposure to tobacco and alcohol, which were generally higher among males than females (33, 34, 36). However, the tobacco and alcohol industries target women and girls, especially those living in low- and middle-income countries, through aggressive marketing tactics, making it more acceptable among them (55). Health outcomes from tobacco and alcohol use vary by gender due to sex-specific factors (55). Furthermore, chewing tobacco was culturally widely acceptable and the primary contributor to early-onset LOC burden among females in South Asia (33). Therefore, restricting such marketing practices and culturally relevant education is urgently needed for young adults.

Human papillomavirus (HPV) infection is indeed a recognized risk factor for cancers of the oral cavity, tonsils, and oropharynx, and this is particularly relevant in certain regions of the world (56, 57). Over the past decades, the incidence of both HPV-positive and HPV-negative OPC has risen, with evidence indicating that HPV-positive cases are increasing at a faster rate (58). HPV-associated OPC incidence is higher in wealthier countries (57). Smoking has been linked to poorer survival outcomes in HPV-associated OPC and, as our analysis shows, contributed significantly to OPC-related deaths and DALYs in high-income regions (59). This underscores the critical role of smoking as a risk factor, even in areas where HPV-associated OPC is more prevalent. Additionally, two studies have shown that the median age at diagnosis for HPV-positive OPC increased from 53 to 58 years between 1998 and 2013, and from 52 to 59 years between 2002 and 2017, respectively (60, 61). A significant rise in incidence has also been observed among white men aged 65 and older, with nearly 10% of cases occurring in individuals 70 years and older (62). As a result, the burden of HPV-positive OPC is increasingly shifting towards older men. This study has assessed the burden of LOC and OPC attributed to tobacco use and alcohol consumption. These risk factors were selected based on the risk-outcome pairs identified in the GBD study 2019, which met the World Cancer Research Fund’s criteria for convincing or probable evidence. Prior Mendelian randomization analyses have also shown that smoking and alcohol consumption independently contribute to the development of LOC and OPC (63). However, it is essential to also consider the rates of HPV infection and vaccination when estimating historical OPC incidence and predicting future trends. To address this limitation, we recommend developing and applying a range of analytical methods to enhance and validate the findings of this study.

Our study has some limitations. Firstly, the accuracy and robustness of our estimates for risk-related LOC and OPC burden among young adults were compromised by methodological defects in the GBD Study 2019. Raw cancer registry data and exposure measurement are incomplete and not available in some countries, particularly low- and middle-income countries. Even when data was accessible, the preferred case definition or measurement method may vary across geography and time (22, 23). Secondly, the smoking and chewing tobacco were modeled with lower age restrictions of 30 years in the GBD Study 2019; therefore, estimates were not generated for these risk factors in the age groups of 15-19 years, 20-24 years, and 25-29 years. Trends in the burden of tobacco- and alcohol-attributable LOC and OPC among young adults aged 15-49 years identified in the current study should be interpreted with caution (22). Thirdly, the burden of early-onset LOC and OPC attributable to other well-established risk factors, such as HPV infection (Oropharyngeal cancers), ultraviolet radiation (Lip cancer), and dietary risk factors, have not been estimated in the present study (3). Fourthly, our study did not more specifically estimate the global distribution and trend of risk-attributable early-onset LOC and OPC by anatomical subsite, which involve different risk factors, molecular characteristics, and prognosis (3). Fifthly, as the data in GBD Study 2019 is at the country level, our results did not take into account the differences variations in demographic characteristics, health behaviors, and economic development patterns at the sub-national level. Although the current study could not fully estimate the impacts of all modifiable risks on early-onset LOC and OPC burden, the results were estimated from the most recent accessible resources to produce relatively valid estimates.

## 5 Conclusions

In summary, the growing burden of early-onset lip, oral cavity, and other pharyngeal cancers poses a global public health challenge. Although the ASMR and ASDR of tobacco- and alcohol-attributable early-onset LOC and OPC have decreased worldwide, certain regions showed increases. The considerable increase in the absolute number of deaths and DALYs indicates that the overall burden of early-onset LOC and OPC attributable modifiable risks remains substantial. Our study emphasizes that effective tobacco and alcohol control is the critical step toward addressing the LOC and OPC cancer burden among young adults. Policymakers, health-care providers, and communities at risk s should be aware the geographic variations and epidemiological trends of risk-related LOC and OPC burden in young adults and should tailor age-and region-appropriate interventions.

## Data Availability

The original contributions presented in the study are included in the article/**Supplementary Material**. Further inquiries can be directed to the corresponding author/s.
