# Supplementary material for: Tobacco- and alcohol-attributable burden of early-onset lip, oral cavity, and pharyngeal cancer in 204 countries and territories from 1990 to 2019, with projections to 2040
Source: Front Oncol. 2024 Oct 10;14:1429972. doi: 10.3389/fonc.2024.1429972 (PMC11499062; doi:10.3389/fonc.2024.1429972)

**Title:** Tobacco- and alcohol-attributable Burden of Early-onset Lip, Oral cavity, and Pharyngeal Cancer in 204 Countries and Territories from 1990 to 2019, with Projections to 2040

**Running title:** Risk-related Early-onset LOC and OPC

**Authors:** Xingzhu Dai[1], Yuanhao Liang[2,*]

**Affiliations:**

[1] Department of Stomatology, Guangdong Provincial People's Hospital (Guangdong Academy of Medical Sciences), Southern Medical University, Guangzhou, China

[2] Clinical Experimental Center, Jiangmen Key Laboratory of Clinical Biobanks and Translational Research, Jiangmen Central Hospital, Jiangmen, China

[*] Corresponding author

**Supplementary Table S1. Global and regional death cases and age-specific mortality rates for tobacco- and alcohol-attributable early-onset Lip and oral cavity cancer, both sexes combined, in 1990 and 2019, and change in absolute deaths and age-specific mortality rate from 1990 to 2019**

| Characteristics | 1990 death | | 2019 death | | 1990-2019 death | |
|---|---|---|---|---|---|---|
| | Case number<br>No. (95% UI) | Rate per 100,000<br>No. (95% UI) | Case number<br>No. (95% UI) | Rate per 100,000<br>No. (95% UI) | Percent change in case number (%) | EAPC<br>No. (95% CI) |
| **Overall** | 9,802 (8,685-10,886) | 0.36 (0.32-0.4) | 16,887 (14,245-19,688) | 0.43 (0.36-0.5) | 72.3 | 0.47 (0.37 to 0.56) |
| **Sex** | | | | | | |
| Male | 8,086 (7,026-9,140) | 0.59 (0.51-0.67) | 13,680 (11,235-16,107) | 0.69 (0.56-0.81) | 69.2 | 0.44 (0.33 to 0.55) |
| Female | 1,716 (1,389-2,049) | 0.13 (0.10-0.15) | 3,207 (2,527-3,983) | 0.16 (0.13-0.20) | 86.9 | 0.59 (0.46 to 0.73) |
| **Socio-demographic index** | | | | | | |
| High | 1,828 (1,681-1,958) | 0.42 (0.39-0.46) | 1,365 (1,192-1,538) | 0.29 (0.25-0.33) | −25.3 | −1.64 (−1.97 to −1.3) |
| High -middle | 2,218 (2,010-2,419) | 0.37 (0.33-0.4) | 2,936 (2,538-3,331) | 0.4 (0.35-0.46) | 32.4 | −0.2 (−0.42 to 0.03) |
| Middle | 1,965 (1,688-2,269) | 0.22 (0.19-0.25) | 4,312 (3,495-5,208) | 0.34 (0.28-0.41) | 119.4 | 1.77 (1.63 to 1.91) |
| Low-middle | 2,861 (2,381-3,423) | 0.53 (0.44-0.63) | 6,138 (5,004-7,415) | 0.66 (0.54-0.79) | 114.5 | 0.77 (0.7 to 0.85) |
| Low | 927 (745-1,145) | 0.4 (0.32-0.49) | 2,132 (1,694-2,682) | 0.39 (0.31-0.5) | 130 | −0.08 (−0.19 to 0.03) |
| **GBD regions** | | | | | | |
| High-income Asia Pacific | 174 (157-189) | 0.19 (0.17-0.2) | 167 (148-185) | 0.21 (0.18-0.23) | −4 | −0.03 (−0.4 to 0.33) |
| Central Asia | 69 (60-81) | 0.21 (0.18-0.24) | 116 (98-138) | 0.24 (0.2-0.28) | 68.1 | −0.15 (−0.47 to 0.16) |
| East Asia | 795 (644-960) | 0.12 (0.09-0.14) | 1,763 (1,397-2,190) | 0.24 (0.19-0.29) | 121.8 | 2.97 (2.7 to 3.24) |
| South Asia | 4363 (3,620-5,169) | 0.82 (0.68-0.98) | 9,790 (7,792-12,141) | 1 (0.8-1.25) | 124.4 | 0.74 (0.65 to 0.84) |
| Southeast Asia | 487 (401-578) | 0.21 (0.17-0.24) | 1,053 (806-1,344) | 0.29 (0.22-0.37) | 116.2 | 1.09 (1 to 1.19) |
| Australasia | 68 (61-76) | 0.63 (0.57-0.7) | 48 (40-58) | 0.36 (0.3-0.43) | −29.4 | −1.93 (−2.27 to −1.58) |
| Caribbean | 43 (36-50) | 0.23 (0.2-0.27) | 59 (45-75) | 0.25 (0.19-0.31) | 37.2 | 0.35 (0.26 to 0.43) |
| Central Europe | 549 (504-591) | 0.9 (0.83-0.97) | 407 (334-483) | 0.77 (0.63-0.92) | −25.9 | −1.53 (−1.91 to −1.16) |
| Eastern Europe | 693 (621-784) | 0.63 (0.56-0.71) | 966 (808-1,137) | 0.98 (0.82-1.16) | 39.4 | 0.78 (0.34 to 1.23) |
| Western Europe | 1200 (1,104-1,287) | 0.62 (0.57-0.67) | 593 (523-659) | 0.31 (0.27-0.35) | −50.6 | −2.93 (−3.27 to −2.6) |
| Andean Latin America | 16 (12-20) | 0.09 (0.07-0.11) | 28 (20-39) | 0.09 (0.06-0.12) | 75 | 0.14 (−0.03 to 0.3) |

| | | | | | | |
|---|---|---|---|---|---|---|
| Central Latin America | 77 (67-86) | 0.09 (0.08-0.11) | 118 (94-145) | 0.09 (0.07-0.11) | 53.2 | −0.4 (−0.53 to −0.27) |
| Southern Latin America | 66 (58-74) | 0.27 (0.24-0.3) | 66 (55-78) | 0.19 (0.16-0.23) | 0 | −1.38 (−1.56 to −1.2) |
| Tropical Latin America | 286 (248-321) | 0.36 (0.32-0.41) | 408 (346-469) | 0.34 (0.29-0.39) | 42.7 | −0.5 (−0.95 to −0.05) |
| North Africa and Middle East | 103 (76-134) | 0.06 (0.05-0.08) | 199 (151-257) | 0.06 (0.05-0.08) | 93.2 | −0.58 (−0.7 to −0.46) |
| High-income North America | 444 (405-480) | 0.3 (0.27-0.32) | 301 (269-331) | 0.18 (0.16-0.2) | −32.2 | −1.99 (−2.2 to −1.79) |
| Oceania | 6 (4-9) | 0.2 (0.14-0.29) | 14 (9-21) | 0.21 (0.14-0.31) | 133.3 | 0.43 (0.3 to 0.56) |
| Central Sub-Saharan Africa | 34 (24-47) | 0.14 (0.1-0.19) | 86 (59-120) | 0.14 (0.1-0.19) | 152.9 | 0 (−0.22 to 0.23) |
| Eastern Sub-Saharan Africa | 167 (129-213) | 0.2 (0.16-0.26) | 422 (328-536) | 0.21 (0.16-0.27) | 152.7 | 0.1 (0.06 to 0.14) |
| Southern Sub-Saharan Africa | 105 (90-120) | 0.4 (0.34-0.46) | 122 (100-146) | 0.29 (0.24-0.34) | 16.2 | −2.07 (−2.56 to −1.58) |
| Western Sub-Saharan Africa | 56 (44-71) | 0.07 (0.05-0.08) | 161 (123-206) | 0.07 (0.06-0.1) | 187.5 | 0.33 (0.26 to 0.4) |

**Supplementary Table S2. Global and regional age-standardized mortality rates and age-standardized DALYs rates for tobacco- and alcohol-attributable early-onset Lip and oral cavity cancer, in 1990 and 2019, and change in age-standardized rates from 1990 to 2019**

| Characteristics | Death | | | DALYs | | |
|---|---|---|---|---|---|---|
| | ASMR in1990 per 100,000 No. (95% UI) | ASMR in 2019 per 100,000 No. (95% UI) | EAPC 1990~2019 No. (95% CI) | ASDR in1990 per 100,000 No. (95% UI) | ASDR in 2019 per 100,000 No. (95% UI) | EAPC 1990~2019 No. (95% CI) |
| **Global** | | | | | | |
| Both | 0.45 (0.44-0.45) | 0.42 (0.42-0.43) | −0.3 (−0.34 to −0.26) | 20.79 (20.73-20.85) | 20 (19.95-20.04) | −0.25 (−0.29 to −0.2) |
| Male | 0.72 (0.71-0.74) | 0.68 (0.67-0.69) | −0.29 (−0.35 to −0.23) | 33.65 (33.54-33.76) | 32.14 (32.06-32.22) | −0.23 (−0.3 to −0.17) |
| Female | 0.16 (0.15-0.16) | 0.16 (0.16-0.17) | −0.17 (−0.28 to −0.06) | 7.43 (7.38-7.48) | 7.67 (7.64-7.71) | −0.13 (−0.25 to −0.02) |
| **High SDI** | | | | | | |
| Both | 0.42 (0.4-0.44) | 0.24 (0.23-0.25) | −2.13 (−2.35 to −1.91) | 19.95 (19.82-20.08) | 11.68 (11.59-11.77) | −2.06 (−2.28 to −1.84) |
| Male | 0.71 (0.68-0.73) | 0.38 (0.36-0.39) | −2.34 (−2.58 to −2.11) | 33.32 (33.08-33.56) | 18.43 (18.27-18.58) | −2.28 (−2.51 to −2.05) |
| Female | 0.13 (0.12-0.14) | 0.09 (0.08-0.1) | −1.33 (−1.51 to −1.14) | 6.37 (6.28-6.47) | 4.57 (4.5-4.64) | −1.24 (−1.44 to −1.05) |
| **High-middle SDI** | | | | | | |
| Both | 0.43 (0.42-0.45) | 0.34 (0.33-0.35) | −1.24 (−1.39 to −1.09) | 20.05 (19.92-20.17) | 15.99 (15.91-16.08) | −1.16 (−1.32 to −1) |
| Male | 0.77 (0.74-0.8) | 0.59 (0.57-0.61) | −1.33 (−1.48 to −1.17) | 35.65 (35.42-35.88) | 27.7 (27.55-27.86) | −1.25 (−1.42 to −1.09) |
| Female | 0.09 (0.08-0.09) | 0.08 (0.07-0.09) | −0.49 (−0.6 to −0.38) | 4.08 (4.01-4.15) | 3.95 (3.9-4.01) | −0.44 (−0.57 to −0.3) |
| **Middle SDI** | | | | | | |
| Both | 0.29 (0.28-0.3) | 0.32 (0.31-0.33) | 0.53 (0.42 to 0.64) | 13.57 (13.48-13.66) | 15.3 (15.23-15.36) | 0.56 (0.45 to 0.68) |
| Male | 0.5 (0.48-0.52) | 0.57 (0.55-0.58) | 0.7 (0.56 to 0.83) | 23.27 (23.11-23.43) | 27.14 (27.02-27.27) | 0.73 (0.59 to 0.87) |
| Female | 0.07 (0.06-0.07) | 0.07 (0.06-0.07) | −0.04 (−0.14 to 0.05) | 3.23 (3.18-3.29) | 3.36 (3.31-3.4) | −0.03 (−0.14 to 0.07) |
| **Low-middle SDI** | | | | | | |
| Both | 0.7 (0.67-0.72) | 0.74 (0.72-0.75) | 0.13 (0.08 to 0.18) | 32.64 (32.47-32.82) | 34.76 (34.64-34.89) | 0.18 (0.11 to 0.25) |
| Male | 1.01 (0.97-1.05) | 1.11 (1.08-1.13) | 0.37 (0.3 to 0.43) | 47.32 (47.03-47.61) | 52.41 (52.2-52.63) | 0.42 (0.32 to 0.51) |
| Female | 0.36 (0.34-0.38) | 0.37 (0.35-0.38) | −0.34 (−0.5 to −0.19) | 17.21 (17.03-17.39) | 17.17 (17.04-17.29) | −0.32 (−0.49 to −0.15) |

| | | | | | | |
|---|---|---|---|---|---|---|
| **Low SDI** | | | | | | |
| Both | 0.55 (0.52-0.58) | 0.54 (0.52-0.55) | −0.18 (−0.33 to −0.04) | 25.69 (25.45-25.93) | 25.05 (24.9-25.21) | −0.16 (−0.31 to −0.01) |
| Male | 0.8 (0.75-0.84) | 0.76 (0.73-0.79) | −0.21 (−0.36 to −0.07) | 37.34 (36.93-37.75) | 35.5 (35.24-35.76) | −0.19 (−0.34 to −0.05) |
| Female | 0.3 (0.27-0.32) | 0.31 (0.29-0.33) | −0.06 (−0.21 to 0.09) | 13.93 (13.7-14.17) | 14.64 (14.48-14.8) | −0.06 (−0.24 to 0.12) |
| **High-income Asia Pacific** | | | | | | |
| Both | 0.17 (0.15-0.19) | 0.15 (0.13-0.16) | −0.78 (−1.17 to −0.39) | 8.45 (8.28-8.62) | 7.63 (7.47-7.78) | −0.66 (−1.06 to −0.26) |
| Male | 0.26 (0.23-0.29) | 0.2 (0.17-0.22) | −1.27 (−1.7 to −0.84) | 12.66 (12.37-12.94) | 10.28 (10.03-10.53) | −1.12 (−1.57 to −0.67) |
| Female | 0.08 (0.06-0.09) | 0.09 (0.07-0.1) | 0.44 (0.17 to 0.71) | 4.14 (3.99-4.28) | 4.8 (4.64-4.96) | 0.53 (0.22 to 0.83) |
| **Central Asia** | | | | | | |
| Both | 0.32 (0.27-0.36) | 0.24 (0.21-0.26) | −1.23 (−1.3 to −1.15) | 13.94 (13.5-14.38) | 11.9 (11.61-12.18) | −1.17 (−1.38 to −0.95) |
| Male | 0.57 (0.48-0.65) | 0.4 (0.35-0.45) | −1.45 (−1.53 to −1.38) | 24.63 (23.8-25.46) | 20.09 (19.55-20.62) | −1.38 (−1.61 to −1.15) |
| Female | 0.06 (0.04-0.08) | 0.07 (0.05-0.09) | 0.74 (0.72 to 0.76) | 3.64 (3.37-3.91) | 3.95 (3.74-4.15) | 0.03 (−0.11 to 0.17) |
| **East Asia** | | | | | | |
| Both | 0.15 (0.14-0.15) | 0.19 (0.18-0.19) | 1.31 (1.1 to 1.51) | 6.92 (6.85-6.99) | 9.02 (8.96-9.08) | 1.29 (1.1 to 1.48) |
| Male | 0.26 (0.24-0.27) | 0.35 (0.34-0.37) | 1.57 (1.34 to 1.8) | 12.33 (12.2-12.45) | 16.99 (16.87-17.11) | 1.55 (1.33 to 1.77) |
| Female | 0.02 (0.02-0.02) | 0.01 (0.01-0.02) | −1.86 (−2.09 to −1.64) | 1 (0.97-1.03) | 0.74 (0.71-0.76) | −1.71 (−2.09 to −1.32) |
| **South Asia** | | | | | | |
| Both | 1.05 (1.02-1.07) | 1.13 (1.11-1.15) | 0.31 (0.21 to 0.4) | 48.89 (48.67-49.1) | 53.26 (53.11-53.42) | 0.35 (0.25 to 0.45) |
| Male | 1.52 (1.48-1.57) | 1.7 (1.67-1.74) | 0.55 (0.43 to 0.68) | 71.08 (70.73-71.43) | 80.61 (80.35-80.88) | 0.6 (0.46 to 0.75) |
| Female | 0.51 (0.49-0.54) | 0.54 (0.52-0.55) | −0.17 (−0.31 to −0.02) | 24.26 (24.05-24.48) | 25.09 (24.94-25.24) | −0.15 (−0.31 to 0.01) |
| **Southeast Asia** | | | | | | |
| Both | 0.28 (0.26-0.3) | 0.28 (0.27-0.3) | −0.12 (−0.18 to −0.07) | 13.09 (12.92-13.26) | 13.38 (13.26-13.5) | −0.13 (−0.22 to −0.04) |
| Male | 0.5 (0.47-0.54) | 0.51 (0.48-0.53) | −0.08 (−0.13 to −0.03) | 23.3 (22.99-23.62) | 23.91 (23.69-24.13) | −0.1 (−0.19 to −0.01) |
| Female | 0.07 (0.06-0.08) | 0.05 (0.05-0.06) | −0.76 (−0.79 to −0.73) | 3.23 (3.13-3.32) | 2.79 (2.72-2.85) | −0.72 (−0.8 to −0.64) |
| **Australasia** | | | | | | |
| Both | 0.62 (0.53-0.7) | 0.3 (0.25-0.35) | −2.34 (−2.47 to −2.21) | 30.49 (29.55-31.43) | 14.95 (14.41-15.48) | −2.27 (−2.51 to −2.04) |
| Male | 0.94 (0.79-1.08) | 0.42 (0.34-0.49) | −2.63 (−2.76 to −2.49) | 46.29 (44.66-47.93) | 21.76 (20.84-22.68) | −2.44 (−2.68 to −2.2) |
| Female | 0.26 (0.19-0.32) | 0.15 (0.11-0.19) | −1.8 (−2.02 to −1.58) | 14.19 (13.45-14.94) | 8.33 (7.87-8.79) | −1.63 (−1.9 to −1.36) |
| **Caribbean** | | | | | | |
| Both | 0.27 (0.22-0.31) | 0.23 (0.2-0.26) | −0.53 (−0.6 to −0.46) | 13.69 (13.22-14.15) | 11.64 (11.29-11.98) | −0.59 (−0.7 to −0.49) |

| | | | | | | |
|---|---|---|---|---|---|---|
| Male | 0.44 (0.36-0.53) | 0.4 (0.33-0.46) | −0.44 (−0.53 to −0.35) | 23.32 (22.45-24.19) | 19.98 (19.34-20.63) | −0.56 (−0.68 to −0.44) |
| Female | 0.05 (0.03-0.07) | 0.05 (0.03-0.07) | 0 (−0.11 to 0.11) | 4.4 (4.1-4.71) | 3.53 (3.31-3.75) | −0.88 (−0.94 to −0.81) |
| **Central Europe** | | | | | | |
| Both | 0.91 (0.86-0.97) | 0.57 (0.53-0.61) | −2.05 (−2.27 to −1.83) | 41.2 (40.7-41.69) | 27.17 (26.8-27.55) | −2.02 (−2.25 to −1.79) |
| Male | 1.69 (1.59-1.8) | 1 (0.93-1.07) | −2.24 (−2.47 to −2.01) | 75.85 (74.9-76.8) | 47.96 (47.25-48.66) | −2.21 (−2.45 to −1.96) |
| Female | 0.14 (0.11-0.16) | 0.11 (0.09-0.12) | −1.02 (−1.2 to −0.84) | 6.86 (6.63-7.09) | 5.75 (5.55-5.95) | −0.88 (−1.06 to −0.71) |
| **Eastern Europe** | | | | | | |
| Both | 0.66 (0.62-0.71) | 0.79 (0.75-0.84) | 0.16 (−0.21 to 0.53) | 30.93 (30.59-31.28) | 37.24 (36.9-37.59) | 0.25 (−0.15 to 0.65) |
| Male | 1.26 (1.17-1.35) | 1.46 (1.37-1.55) | 0.02 (−0.36 to 0.4) | 58.59 (57.92-59.27) | 67.8 (67.13-68.48) | 0.1 (−0.3 to 0.51) |
| Female | 0.11 (0.1-0.13) | 0.18 (0.16-0.2) | 1.66 (1.46 to 1.85) | 5.18 (5-5.36) | 8.61 (8.38-8.84) | 1.65 (1.29 to 2.01) |
| **Western Europe** | | | | | | |
| Both | 0.6 (0.58-0.63) | 0.24 (0.22-0.25) | −3.73 (−4.01 to −3.45) | 28.37 (28.14-28.6) | 11.78 (11.65-11.92) | −3.65 (−3.93 to −3.36) |
| Male | 1.05 (1-1.1) | 0.37 (0.34-0.39) | −4.18 (−4.49 to −3.87) | 49.15 (48.72-49.58) | 18.34 (18.1-18.57) | −4.1 (−4.42 to −3.78) |
| Female | 0.15 (0.13-0.16) | 0.1 (0.09-0.11) | −1.59 (−1.78 to −1.39) | 7.31 (7.17-7.46) | 5.16 (5.05-5.27) | −1.44 (−1.64 to −1.24) |
| **Andean Latin America** | | | | | | |
| Both | 0.09 (0.06-0.11) | 0.08 (0.06-0.09) | −0.36 (−0.41 to −0.31) | 5.4 (5.12-5.67) | 4.37 (4.19-4.55) | −0.53 (−0.67 to −0.4) |
| Male | 0.12 (0.09-0.16) | 0.11 (0.08-0.14) | −0.34 (−0.36 to −0.31) | 8.32 (7.84-8.8) | 6.38 (6.08-6.68) | −0.7 (−0.83 to −0.56) |
| Female | 0.02 (0.01-0.03) | 0.03 (0.02-0.04) | 1.74 (1.63 to 1.85) | 2.51 (2.29-2.73) | 2.41 (2.25-2.57) | 0 (−0.14 to 0.13) |
| **Central Latin America** | | | | | | |
| Both | 0.12 (0.11-0.14) | 0.09 (0.08-0.1) | −1.28 (−1.4 to −1.15) | 6.16 (5.99-6.32) | 4.53 (4.43-4.63) | −1.3 (−1.45 to −1.15) |
| Male | 0.2 (0.17-0.23) | 0.14 (0.12-0.16) | −1.32 (−1.41 to −1.22) | 9.79 (9.5-10.09) | 7.29 (7.11-7.48) | −1.29 (−1.44 to −1.15) |
| Female | 0.05 (0.03-0.06) | 0.03 (0.03-0.04) | −1.12 (−1.18 to −1.06) | 2.66 (2.53-2.78) | 1.99 (1.92-2.07) | −1.12 (−1.26 to −0.98) |
| **Southern Latin America** | | | | | | |
| Both | 0.27 (0.23-0.31) | 0.17 (0.15-0.2) | −1.71 (−1.79 to −1.63) | 13.43 (13.03-13.84) | 8.95 (8.69-9.22) | −1.64 (−1.78 to −1.5) |
| Male | 0.45 (0.38-0.52) | 0.26 (0.21-0.3) | −2.13 (−2.22 to −2.05) | 22.31 (21.56-23.06) | 13.88 (13.42-14.35) | −1.95 (−2.12 to −1.78) |
| Female | 0.07 (0.05-0.09) | 0.07 (0.05-0.09) | −0.15 (−0.17 to −0.13) | 4.78 (4.51-5.05) | 4.2 (3.98-4.41) | −0.47 (−0.5 to −0.45) |
| **Tropical Latin America** | | | | | | |
| Both | 0.47 (0.43-0.5) | 0.32 (0.3-0.34) | −1.52 (−1.92 to −1.12) | 21.95 (21.59-22.31) | 15.36 (15.14-15.57) | −1.5 (−1.9 to −1.09) |
| Male | 0.84 (0.77-0.91) | 0.57 (0.53-0.61) | −1.56 (−1.97 to −1.15) | 39.47 (38.77-40.16) | 27.41 (27-27.81) | −1.54 (−1.96 to −1.12) |
| Female | 0.1 (0.08-0.12) | 0.08 (0.07-0.09) | −0.97 (−1.33 to −0.6) | 4.95 (4.76-5.14) | 3.91 (3.79-4.04) | −0.97 (−1.37 to −0.57) |

| | | | | | | |
|---|---|---|---|---|---|---|
| **North Africa and Middle East** | | | | | | |
| Both | 0.09 (0.08-0.1) | 0.06 (0.06-0.07) | −1.48 (−1.56 to −1.41) | 4.16 (4.06-4.26) | 2.96 (2.9-3.01) | −1.49 (−1.6 to −1.39) |
| Male | 0.16 (0.14-0.18) | 0.1 (0.09-0.11) | −1.62 (−1.69 to −1.54) | 7.27 (7.08-7.45) | 4.95 (4.86-5.04) | −1.64 (−1.75 to −1.53) |
| Female | 0.02 (0.01-0.02) | 0.01 (0.01-0.02) | −0.6 (−0.66 to −0.53) | 0.87 (0.81-0.92) | 0.7 (0.67-0.73) | −0.93 (−1.02 to −0.83) |
| **High-income North America** | | | | | | |
| Both | 0.3 (0.28-0.32) | 0.16 (0.15-0.17) | −2.29 (−2.35 to −2.23) | 14.75 (14.56-14.94) | 8.14 (8.01-8.26) | −2.15 (−2.23 to −2.08) |
| Male | 0.47 (0.44-0.51) | 0.23 (0.22-0.25) | −2.54 (−2.63 to −2.45) | 22.98 (22.65-23.31) | 11.88 (11.67-12.08) | −2.4 (−2.5 to −2.29) |
| Female | 0.13 (0.11-0.14) | 0.08 (0.07-0.09) | −1.61 (−1.7 to −1.52) | 6.72 (6.56-6.88) | 4.5 (4.39-4.62) | −1.48 (−1.6 to −1.36) |
| **Oceania** | | | | | | |
| Both | 0.27 (0.27-0.27) | 0.25 (0.25-0.25) | −0.08 (−0.19 to 0.03) | 12.4 (11.48-13.31) | 11.63 (11.01-12.25) | −0.07 (−0.16 to 0.01) |
| Male | 0.43 (0.43-0.43) | 0.41 (0.41-0.41) | −0.01 (−0.13 to 0.1) | 19.95 (18.36-21.54) | 19.15 (18.05-20.25) | 0 (−0.1 to 0.09) |
| Female | 0.09 (0.09-0.09) | 0.08 (0.08-0.08) | −0.21 (−0.3 to −0.13) | 3.97 (3.35-4.58) | 3.8 (3.38-4.23) | −0.07 (−0.1 to −0.04) |
| **Central Sub-Saharan Africa** | | | | | | |
| Both | 0.19 (0.16-0.23) | 0.19 (0.16-0.21) | −0.12 (−0.34 to 0.1) | 9.8 (9.42-10.17) | 9.02 (8.78-9.26) | −0.27 (−0.55 to 0) |
| Male | 0.32 (0.25-0.39) | 0.29 (0.25-0.33) | −0.4 (−0.7 to −0.11) | 16.18 (15.47-16.88) | 13.81 (13.39-14.23) | −0.55 (−0.89− to 0.21) |
| Female | 0.06 (0.03-0.08) | 0.07 (0.05-0.09) | 1 (0.97 to 1.03) | 3.94 (3.67-4.21) | 4.21 (4.01-4.41) | 0.25 (0.14 to 0.36) |
| **Eastern Sub-Saharan Africa** | | | | | | |
| Both | 0.3 (0.27-0.33) | 0.3 (0.28-0.32) | 0 (−0.05 to 0.04) | 14.15 (13.89-14.42) | 14.18 (14-14.36) | −0.02 (−0.07 to 0.02) |
| Male | 0.49 (0.44-0.54) | 0.47 (0.43-0.5) | −0.17 (−0.24 to −0.1) | 22.93 (22.45-23.42) | 21.89 (21.57-22.21) | −0.2 (−0.28 to −0.13) |
| Female | 0.11 (0.09-0.13) | 0.13 (0.12-0.15) | 0.75 (0.72 to 0.79) | 5.63 (5.43-5.83) | 6.66 (6.5-6.81) | 0.59 (0.53 to 0.64) |
| **Southern Sub-Saharan Africa** | | | | | | |
| Both | 0.57 (0.5-0.64) | 0.3 (0.26-0.34) | −2.71 (−3.12 to −2.31) | 26.9 (26.18-27.63) | 15.38 (14.99-15.76) | −2.68 (−3.13 to −2.22) |
| Male | 0.97 (0.84-1.11) | 0.5 (0.43-0.57) | −2.84 (−3.35 to −2.33) | 46.29 (44.93-47.65) | 25.94 (25.22-26.65) | −2.8 (−3.34 to −2.25) |
| Female | 0.15 (0.11-0.19) | 0.09 (0.06-0.11) | −1.87 (−2.02 to −1.73) | 8.5 (7.99-9) | 5.15 (4.86-5.44) | −1.93 (−2.19 to −1.66) |
| **Western Sub-Saharan Africa** | | | | | | |
| Both | 0.09 (0.08-0.11) | 0.1 (0.09-0.12) | 0.36 (0.31 to 0.41) | 4.31 (4.18-4.44) | 4.87 (4.78-4.97) | 0.25 (0.16 to 0.34) |
| Male | 0.13 (0.11-0.15) | 0.16 (0.14-0.18) | 0.72 (0.67 to 0.77) | 6.22 (6.01-6.43) | 7.58 (7.41-7.76) | 0.59 (0.51 to 0.68) |
| Female | 0.04 (0.03-0.05) | 0.05 (0.04-0.05) | 0.48 (0.45 to 0.51) | 2.16 (2.05-2.28) | 2.34 (2.25-2.42) | 0.01 (−0.09 to 0.11) |

**Supplementary Table S3. Global and regional DALYs counts and age-specific DALYs rates for tobacco- and alcohol-attributable early-onset Lip and oral cavity cancer, both sexes combined, in 1990 and 2019, and change in absolute DALYs and age-specific DALYs from 1990 to 2019**

| Characteristics | 1990 DALYs | | 2019 DALYs | | 1990-2019 DALYs | |
|---|---|---|---|---|---|---|
| | Case number No. (95% UI) | Rate per 100,000 No. (95% UI) | Case number No. (95% UI) | Rate per 100,000 No. (95% UI) | Percent change in case number (%) | EAPC No. (95% CI) |
| **Overall** | 463,077 (410,946-515,002) | 17.07 (15.15-18.99) | 799,495 (672,639-930,964) | 20.32 (17.09-23.66) | 72.6 | 0.47 (0.38 to 0.56) |
| **Sex** | | | | | | |
| Male | 381,091 (331,644-429,946) | 27.73 (24.13-31.28) | 647,147 (533,486-760,168) | 32.52 (26.81-38.20) | 69.8 | 0.44 (0.33 to 0.55) |
| Female | 81,985 (66,555-97,753) | 6.13 (4.97-7.31) | 152,348 (119,602-189,367) | 7.83 (6.15-9.73) | 85.8 | 0.56 (0.42 to 0.70) |
| **Socio-demographic index** | | | | | | |
| High | 86,954 (79,666-93,543) | 20.22 (18.52-21.75) | 65,198 (56,895-73,555) | 13.87 (12.11-15.65) | −25 | −1.62 (−1.93 to −1.3) |
| High -middle | 103,972 (94,244-113,419) | 17.18 (15.57-18.74) | 137,287 (118,218-155,239) | 18.84 (16.23-21.31) | 32 | −0.19 (−0.4 to 0.02) |
| Middle | 93,671 (80,426-107,866) | 10.38 (8.91-11.95) | 204,317 (166,015-246,401) | 16.21 (13.17-19.55) | 118.1 | 1.72 (1.58 to 1.85) |
| Low-middle | 134,696 (112,065-160,444) | 24.92 (20.73-29.69) | 291,374 (238,666-350,182) | 31.22 (25.57-37.52) | 116.3 | 0.79 (0.71 to 0.87) |
| Low | 43,612 (35,055-53,665) | 18.75 (15.07-23.08) | 101,078 (80,403-126,357) | 18.71 (14.88-23.38) | 131.8 | −0.05 (−0.16 to 0.06) |
| **GBD regions** | | | | | | |
| High-income Asia Pacific | 8,350 (7,543-9,119) | 8.99 (8.12-9.82) | 8,001 (7,072-8,891) | 9.86 (8.72-10.96) | −4.2 | 0 (−0.36 to 0.37) |
| Central Asia | 3,374 (2,907-3,936) | 10.11 (8.71-11.8) | 5,609 (4,734-6,660) | 11.49 (9.7-13.64) | 66.2 | −0.19 (−0.49 to 0.1) |
| East Asia | 38,439 (31,045-46,269) | 5.57 (4.5-6.7) | 83,037 (66,171-102,679) | 11.13 (8.87-13.76) | 116 | 2.83 (2.58 to 3.08) |
| South Asia | 205,252 (170,285-243,208) | 38.78 (32.17-45.95) | 465,077 (370,683-575,460) | 47.74 (38.05-59.07) | 126.6 | 0.77 (0.67 to 0.87) |
| Southeast Asia | 23,230 (19,123-27,461) | 9.83 (8.09-11.62) | 49,578 (38,151-62,747) | 13.69 (10.54-17.33) | 113.4 | 1.01 (0.91 to 1.1) |
| Australasia | 3,283 (2,930-3,619) | 30.42 (27.16-33.54) | 2,319 (1,935-2,738) | 17.15 (14.31-20.25) | −29.4 | −1.94 (−2.28 to −1.61) |
| Caribbean | 2,032 (1,722-2,377) | 11.14 (9.44-13.03) | 2,796 (2,138-3,530) | 11.69 (8.94-14.76) | 37.6 | 0.27 (0.19 to 0.34) |
| Central Europe | 25,273 (23,207-27,162) | 41.43 (38.04-44.52) | 18,499 (15,181-21,921) | 35.09 (28.8-41.58) | −26.8 | −1.55 (−1.91 to −1.2) |
| Eastern Europe | 32,067 (28,770-36,117) | 29.07 (26.08-32.74) | 44,690 (37,554-52,336) | 45.57 (38.3-53.37) | 39.4 | 0.81 (0.38 to 1.25) |
| Western Europe | 56,658 (52,002-60,809) | 29.29 (26.89-31.44) | 28,121 (24,897-31,260) | 14.75 (13.06-16.39) | −50.4 | −2.92 (−3.24 to −2.6) |
| Andean Latin America | 803 (627-999) | 4.31 (3.37-5.36) | 1,398 (1,001-1,929) | 4.22 (3.02-5.82) | 74.1 | 0.12 (−0.05 to 0.29) |

| | | | | | | |
|---|---|---|---|---|---|---|
| Central Latin America | 3,812 (3,315-4,286) | 4.67 (4.07-5.26) | 5,771 (4,627-7,015) | 4.38 (3.51-5.33) | 51.4 | −0.46 (−0.59 to −0.32) |
| Southern Latin America | 3,080 (2,713-3,478) | 12.57 (11.07-14.2) | 3,152 (2,630-3,723) | 9.26 (7.73-10.94) | 2.3 | −1.32 (−1.48 to −1.15) |
| Tropical Latin America | 13,384 (11,643-15,002) | 17.04 (14.82-19.1) | 18,987 (16,137-21,874) | 15.93 (13.54-18.35) | 41.9 | −0.52 (−0.96 to −0.08) |
| North Africa and Middle East | 4,858 (3,578-6,316) | 2.99 (2.2-3.89) | 9,398 (7,108-12,129) | 2.82 (2.13-3.64) | 93.5 | −0.58 (−0.7 to −0.46) |
| High-income North America | 21,711 (19,775-23,469) | 14.61 (13.3-15.79) | 14,896 (13,281-16,372) | 8.93 (7.96-9.82) | −31.4 | −1.95 (−2.14 to −1.75) |
| Oceania | 303 (212-447) | 9.57 (6.68-14.13) | 681 (442-1,022) | 10.01 (6.5-15.03) | 124.8 | 0.39 (0.26 to 0.52) |
| Central Sub-Saharan Africa | 1,614 (1,141-2,214) | 6.61 (4.67-9.07) | 4,072 (2,789-5,643) | 6.55 (4.49-9.08) | 152.3 | 0 (−0.24 to 0.24) |
| Eastern Sub-Saharan Africa | 7,935 (6,122-10,055) | 9.56 (7.38-12.12) | 20,097 (15,619-25,596) | 10.1 (7.85-12.87) | 153.3 | 0.12 (0.08 to 0.16) |
| Southern Sub-Saharan Africa | 4,965 (4,264-5,681) | 19.03 (16.35-21.78) | 5,749 (4,760-6,841) | 13.59 (11.25-16.17) | 15.8 | −2.07 (−2.56 to −1.58) |
| Western Sub-Saharan Africa | 2,654 (2,060-3,319) | 3.12 (2.42-3.9) | 7,567 (5,762-9,677) | 3.52 (2.68-4.5) | 185.1 | 0.32 (0.25 to 0.4) |

**Supplementary Table S4. Global and regional death cases and age-specific mortality rates for tobacco- and alcohol-attributable early-onset Other pharyngeal cancer, both sexes combined, in 1990 and 2019, and change in absolute deaths and age-specific mortality rate from 1990 to 2019**

| Characteristics | 1990 death | | 2019 death | | 1990-2019 death | |
|---|---|---|---|---|---|---|
| | Case number No. (95% UI) | Rate per 100,000 No. (95% UI) | Case number No. (95% UI) | Rate per 100,000 No. (95% UI) | Percent change in case number (%) | EAPC No. (95% CI) |
| **Overall** | 4,866 (4,254-5,488) | 0.18 (0.16-0.2) | 8,402 (6,909-9,946) | 0.21 (0.18-0.25) | 72.7 | 0.3 (0.19 to 0.41) |
| **Sex** | | | | | | |
| Male | 4,512 (3,956-5,084) | 0.33 (0.29-0.37) | 7,880 (6,454-9,340) | 0.40 (0.32-0.47) | 74.6 | 0.37 (0.23 to 0.46) |
| Female | 353 (282-434) | 0.03 (0.02-0.03) | 522 (398-656) | 0.03 (0.02-0.03) | 47.9 | −0.22 (−0.36 to −0.08) |
| **Socio-demographic index** | | | | | | |
| High | 1,075 (1,000-1,147) | 0.25 (0.23-0.27) | 790 (702-883) | 0.17 (0.15-0.19) | −26.5 | −1.89 (−2.19 to −1.59) |
| High -middle | 1,221 (1,102-1,337) | 0.2 (0.18-0.22) | 1,445 (1,267-1,620) | 0.2 (0.17-0.22) | 18.3 | −0.97 (−1.31 to −0.62) |
| Middle | 920 (774-1,067) | 0.1 (0.09-0.12) | 1,997 (1,579-2,396) | 0.16 (0.13-0.19) | 117.1 | 1.49 (1.42 to 1.55) |
| Low-middle | 1,338 (1,057-1,689) | 0.25 (0.2-0.31) | 3,401 (2,599-4,272) | 0.36 (0.28-0.46) | 154.2 | 1.43 (1.32 to 1.54) |
| Low | 310 (224-408) | 0.13 (0.1-0.18) | 767 (572-1,011) | 0.14 (0.11-0.19) | 147.4 | 0.33 (0.24 to 0.43) |
| **GBD regions** | | | | | | |
| High-income Asia Pacific | 60 (54-65) | 0.06 (0.06-0.07) | 70 (60-80) | 0.09 (0.07-0.1) | 16.7 | 0.53 (0.16 to 0.91) |
| Central Asia | 34 (28-41) | 0.1 (0.08-0.12) | 55 (46-66) | 0.11 (0.09-0.14) | 61.8 | −0.32 (−0.61 to −0.02) |
| East Asia | 415 (328-505) | 0.06 (0.05-0.07) | 523 (423-638) | 0.07 (0.06-0.09) | 26 | 0.23 (−0.01 to 0.47) |
| South Asia | 1,951 (1,548-2,416) | 0.37 (0.29-0.46) | 5,039 (3,783-6,363) | 0.52 (0.39-0.65) | 158.3 | 1.24 (1.14 to 1.34) |
| Southeast Asia | 170 (131-209) | 0.07 (0.06-0.09) | 472 (351-627) | 0.13 (0.1-0.17) | 177.6 | 2.16 (2.03 to 2.3) |
| Australasia | 19 (16-22) | 0.18 (0.15-0.2) | 21 (17-26) | 0.16 (0.12-0.19) | 10.5 | −0.37 (−0.68 to −0.07) |
| Caribbean | 26 (22-30) | 0.14 (0.12-0.16) | 33 (25-42) | 0.14 (0.11-0.18) | 26.9 | 0.13 (−0.19 to 0.45) |
| Central Europe | 345 (319-369) | 0.57 (0.52-0.6) | 336 (278-401) | 0.64 (0.53-0.76) | −2.6 | −0.64 (−1.18 to −0.1) |
| Eastern Europe | 361 (303-428) | 0.33 (0.27-0.39) | 507 (424-600) | 0.52 (0.43-0.61) | 40.4 | 0.6 (0.16 to 1.04) |
| Western Europe | 875 (811-937) | 0.45 (0.42-0.48) | 464 (410-524) | 0.24 (0.22-0.27) | −47 | −2.91 (−3.25 to −2.58) |
| Andean Latin America | 7 (5-9) | 0.04 (0.03-0.05) | 9 (6-12) | 0.03 (0.02-0.04) | 28.6 | −1.31 (−1.63 to −0.99) |

| | | | | | | |
|---|---|---|---|---|---|---|
| Central Latin America | 28 (25-32) | 0.03 (0.03-0.04) | 39 (30-50) | 0.03 (0.02-0.04) | 39.3 | −0.73 (−0.87 to −0.59) |
| Southern Latin America | 40 (36-45) | 0.16 (0.15-0.18) | 23 (19-27) | 0.07 (0.06-0.08) | −42.5 | −3.31 (−3.78 to −2.84) |
| Tropical Latin America | 229 (204-254) | 0.29 (0.26-0.32) | 344 (295-391) | 0.29 (0.25-0.33) | 50.2 | −0.42 (−0.85 to 0.01) |
| North Africa and Middle East | 43 (32-55) | 0.03 (0.02-0.03) | 92 (71-119) | 0.03 (0.02-0.04) | 114 | −0.08 (−0.17 to 0.01) |
| High-income North America | 165 (152-177) | 0.11 (0.1-0.12) | 137 (122-151) | 0.08 (0.07-0.09) | −17 | −1.09 (−1.36 to −0.82) |
| Oceania | 1 (1-1) | 0.03 (0.02-0.04) | 2 (1-3) | 0.03 (0.02-0.05) | 100 | 0.19 (0.07 to 0.31) |
| Central Sub-Saharan Africa | 8 (5-12) | 0.03 (0.02-0.05) | 22 (14-31) | 0.04 (0.02-0.05) | 175 | 0.14 (−0.17 to 0.44) |
| Eastern Sub-Saharan Africa | 52 (40-65) | 0.06 (0.05-0.08) | 143 (101-192) | 0.07 (0.05-0.1) | 175 | 0.46 (0.41 to 0.5) |
| Southern Sub-Saharan Africa | 25 (22-29) | 0.1 (0.08-0.11) | 37 (29-44) | 0.09 (0.07-0.1) | 48 | −0.96 (−1.2 to −0.72) |
| Western Sub-Saharan Africa | 13 (10-17) | 0.02 (0.01-0.02) | 34 (25-45) | 0.02 (0.01-0.02) | 161.5 | 0.07 (−0.05 to 0.19) |

**Supplementary Table S5. Global and regional DALYs counts and age-specific DALYs rates for tobacco- and alcohol-attributable early-onset Other pharyngeal cancer, both sexes combined, in 1990 and 2019, and change in absolute DALYs and age-specific DALYs from 1990 to 2019**

| | **1990 DALYs** | | **2019 DALYs** | | **1990-2019 DALYs** | |
|---|---|---|---|---|---|---|
| **Characteristics** | Case number No. (95% UI) | Rate per 100,000 No. (95% UI) | Case number No. (95% UI) | Rate per 100,000 No. (95% UI) | Percent change in case number (%) | EAPC No. (95% CI) |
| **Overall** | 222,148 (193,441-251,125) | 8.19 (7.13-9.26) | 386,418 (316,466-458,971) | 9.82 (8.04-11.66) | 73.9 | 0.33 (0.22 to 0.44) |
| **Sex** | | | | | | |
| Male | 205,562 (179,265-231,833) | 14.96 (13.04-16.87) | 361,808 (296,180-429,406) | 18.18 (14.89-21.58) | 76 | 0.37 (0.26 to 0.48) |
| Female | 16,586 (13,217-20,359) | 1.24 (0.99-1.52) | 24,610 (18,860-31,054) | 1.27 (0.97-1.60) | 48.4 | −0.21 (−0.34 to −0.08) |
| **Socio-demographic index** | | | | | | |
| High | 48,869 (45,312-52,224) | 11.36 (10.54-12.14) | 36,342 (32,210-40,548) | 7.73 (6.85-8.63) | −25.6 | −1.84 (−2.13 to −1.55) |
| High -middle | 55,535 (50,130-60,831) | 9.18 (8.28-10.05) | 65,666 (57,544-73,712) | 9.01 (7.9-10.12) | 18.2 | −0.96 (−1.3 to −0.63) |
| Middle | 42,297 (35,511-49,148) | 4.69 (3.94-5.45) | 91,614 (72,405-110,299) | 7.27 (5.74-8.75) | 116.6 | 1.47 (1.4 to 1.54) |
| Low-middle | 61,194 (48,421-76,919) | 11.32 (8.96-14.23) | 157,205 (119,589-197,522) | 16.84 (12.81-21.16) | 156.9 | 1.46 (1.35 to 1.58) |
| Low | 14,165 (10,198-18,564) | 6.09 (4.39-7.98) | 35,478 (26,550-46,842) | 6.57 (4.91-8.67) | 150.5 | 0.38 (0.29 to 0.48) |
| **GBD regions** | | | | | | |
| High-income Asia Pacific | 2,698 (2,456-2,928) | 2.9 (2.64-3.15) | 3,212 (2,747-3,678) | 3.96 (3.39-4.53) | 19.1 | 0.61 (0.25 to 0.98) |
| Central Asia | 1,609 (1,312-1,950) | 4.82 (3.93-5.84) | 2,595 (2,127-3,096) | 5.31 (4.36-6.34) | 61.3 | −0.35 (−0.63 to −0.07) |
| East Asia | 19,326 (15,345-23,494) | 2.8 (2.22-3.4) | 23,959 (19,439-29,123) | 3.21 (2.6-3.9) | 24 | 0.14 (−0.1 to 0.38) |
| South Asia | 89,110 (70,944-110,635) | 16.83 (13.4-20.9) | 232,998 (175,353-294,417) | 23.92 (18-30.22) | 161.5 | 1.29 (1.19 to 1.39) |
| Southeast Asia | 7,759 (6,044-9,554) | 3.28 (2.56-4.04) | 21,357 (15,876-28,084) | 5.9 (4.38-7.76) | 175.3 | 2.11 (1.98 to 2.23) |
| Australasia | 859 (743-988) | 7.96 (6.89-9.16) | 952 (763-1,179) | 7.04 (5.64-8.72) | 10.8 | −0.37 (−0.67 to −0.07) |
| Caribbean | 1,190 (1,013-1,377) | 6.53 (5.55-7.55) | 1,509 (1,162-1,922) | 6.31 (4.86-8.04) | 26.8 | 0.08 (−0.23 to 0.4) |
| Central Europe | 15,552 (14,371-16,652) | 25.49 (23.56-27.3) | 14,984 (12,434-17,830) | 28.42 (23.59-33.82) | −3.7 | −0.67 (−1.18 to −0.15) |
| Eastern Europe | 16,354 (13,755-19,317) | 14.82 (12.47-17.51) | 23,154 (19,450-27,352) | 23.61 (19.83-27.89) | 41.6 | 0.65 (0.23 to 1.07) |
| Western Europe | 39,461 (36,517-42,306) | 20.4 (18.88-21.87) | 21,112 (18,663-23,764) | 11.07 (9.79-12.46) | −46.5 | −2.89 (−3.21 to −2.56) |

| | | | | | | |
|---|---|---|---|---|---|---|
| Andean Latin America | 325 (249-415) | 1.75 (1.34-2.23) | 410 (291-572) | 1.24 (0.88-1.73) | 26.2 | −1.32 (−1.64 to −1) |
| Central Latin America | 1,346 (1,161-1,519) | 1.65 (1.42-1.86) | 1,821 (1,397-2,310) | 1.38 (1.06-1.75) | 35.3 | −0.79 (−0.94 to −0.64) |
| Southern Latin America | 1,806 (1,597-2,026) | 7.37 (6.52-8.27) | 1,054 (871-1,243) | 3.1 (2.56-3.65) | −41.6 | −3.25 (−3.72 to −2.77) |
| Tropical Latin America | 10,403 (9,291-11,574) | 13.25 (11.83-14.74) | 15,564 (13,331-17,687) | 13.06 (11.18-14.84) | 49.6 | −0.45 (−0.87 to −0.04) |
| North Africa and Middle East | 1,957 (1,484-2,525) | 1.2 (0.91-1.55) | 4,218 (3,264-5,449) | 1.26 (0.98-1.63) | 115.5 | −0.09 (−0.18 to 0) |
| High-income North America | 7,807 (7,164-8,417) | 5.25 (4.82-5.66) | 6,614 (5,887-7,304) | 3.97 (3.53-4.38) | −15.3 | −1.05 (−1.31 to −0.79) |
| Oceania | 47 (32-66) | 1.48 (1.03-2.08) | 102 (68-153) | 1.5 (1-2.24) | 117 | 0.19 (0.07 to 0.32) |
| Central Sub-Saharan Africa | 384 (249-561) | 1.57 (1.02-2.3) | 998 (661-1,399) | 1.61 (1.06-2.25) | 159.9 | 0.14 (−0.17 to 0.45) |
| Eastern Sub-Saharan Africa | 2,371 (1,824-2,962) | 2.86 (2.2-3.57) | 6,517 (4,637-8,758) | 3.28 (2.33-4.4) | 174.9 | 0.46 (0.42 to 0.5) |
| Southern Sub-Saharan Africa | 1,185 (1,029-1,355) | 4.54 (3.95-5.19) | 1,695 (1,368-2,023) | 4.01 (3.23-4.78) | 43 | −1 (−1.23 to −0.77) |
| Western Sub-Saharan Africa | 600 (456-772) | 0.7 (0.54-0.91) | 1,594 (1,184-2,133) | 0.74 (0.55-0.99) | 165.7 | 0.13 (0.01 to 0.25) |

**Supplementary Table S6. Global and regional age-standardized mortality rates and age-standardized DALYs rates for tobacco- and alcohol-attributable early-onset Other pharyngeal cancer, in 1990 and 2019, and change in age-standardized rates from 1990 to 2019**

| Characteristics | Death | | | DALYs | | |
|---|---|---|---|---|---|---|
| | ASMR in1990 per 100,000 No. (95% UI) | ASMR in 2019 per 100,000 No. (95% UI) | EAPC 1990~2019 No. (95% CI) | ASDR in1990 per 100,000 No. (95% UI) | ASDR in 2019 per 100,000 No. (95% UI) | EAPC 1990~2019 No. (95% CI) |
| **Global** | | | | | | |
| Both | 0.23 (0.22-0.23) | 0.21 (0.21-0.21) | −0.56 (−0.64 to −0.48) | 10.22 (10.18-10.27) | 9.65 (9.62-9.68) | −0.49 (−0.58 to −0.4) |
| Male | 0.41 (0.4-0.42) | 0.39 (0.38-0.4) | −0.48 (−0.56 to −0.39) | 18.59 (18.51-18.67) | 17.95 (17.89-18.01) | −0.41 (−0.5 to −0.32) |
| Female | 0.03 (0.03-0.04) | 0.03 (0.02-0.03) | −1.03 (−1.12 to −0.94) | 1.52 (1.5-1.54) | 1.24 (1.23-1.26) | −0.97 (−1.07 to −0.86) |
| **High SDI** | | | | | | |
| Both | 0.25 (0.24-0.26) | 0.13 (0.13-0.14) | −2.5 (−2.68 to −2.31) | 11.29 (11.19-11.39) | 6.33 (6.27-6.4) | −2.41 (−2.58 to −2.23) |
| Male | 0.45 (0.43-0.47) | 0.23 (0.22-0.24) | −2.7 (−2.89 to −2.51) | 20.11 (19.93-20.3) | 10.73 (10.62-10.85) | −2.62 (−2.81 to −2.44) |
| Female | 0.05 (0.04-0.05) | 0.03 (0.03-0.04) | −1.25 (−1.49 to −1.02) | 2.33 (2.28-2.39) | 1.72 (1.68-1.76) | −1.14 (−1.37 to −0.91) |
| **High-middle SDI** | | | | | | |
| Both | 0.25 (0.23-0.26) | 0.16 (0.16-0.17) | −2.11 (−2.36 to −1.87) | 10.95 (10.86-11.04) | 7.54 (7.48-7.6) | −2.05 (−2.31 to −1.8) |
| Male | 0.45 (0.43-0.48) | 0.3 (0.29-0.31) | −2.18 (−2.43 to −1.93) | 20.23 (20.06-20.41) | 13.72 (13.62-13.83) | −2.13 (−2.39 to −1.86) |
| Female | 0.03 (0.03-0.04) | 0.02 (0.02-0.03) | −1.17 (−1.29 to −1.05) | 1.47 (1.43-1.51) | 1.21 (1.18-1.24) | −1.06 (−1.22 to −0.91) |
| **Middle SDI** | | | | | | |
| Both | 0.14 (0.13-0.15) | 0.15 (0.14-0.15) | 0.11 (0.05 to 0.16) | 6.33 (6.27-6.39) | 6.82 (6.78-6.87) | 0.16 (0.08 to 0.23) |
| Male | 0.26 (0.25-0.27) | 0.28 (0.27-0.29) | 0.27 (0.2 to 0.33) | 11.62 (11.51-11.73) | 13.03 (12.94-13.11) | 0.31 (0.23 to 0.39) |
| Female | 0.01 (0.01-0.02) | 0.01 (0.01-0.01) | −0.9 (−0.96 to −0.85) | 0.69 (0.67-0.71) | 0.57 (0.55-0.58) | −0.8 (−0.89 to −0.71) |
| **Low-middle SDI** | | | | | | |
| Both | 0.33 (0.32-0.35) | 0.41 (0.4-0.42) | 0.72 (0.66 to 0.78) | 15.14 (15.02-15.26) | 18.9 (18.81-18.99) | 0.78 (0.68 to 0.87) |
| Male | 0.6 (0.58-0.63) | 0.78 (0.76-0.8) | 0.9 (0.83 to 0.98) | 27.55 (27.32-27.77) | 35.89 (35.7-36.07) | 0.96 (0.85 to 1.07) |
| Female | 0.04 (0.04-0.05) | 0.04 (0.04-0.04) | −0.48 (−0.57 to −0.38) | 2.06 (2-2.11) | 1.97 (1.93-2.01) | −0.46 (−0.61 to −0.31) |

| | | | | | | |
|---|---|---|---|---|---|---|
| **Low SDI** | | | | | | |
| Both | 0.19 (0.17-0.2) | 0.2 (0.19-0.21) | 0.22 (0.19 to 0.24) | 8.55 (8.42-8.69) | 8.95 (8.86-9.04) | 0.24 (0.18 to 0.29) |
| Male | 0.34 (0.32-0.37) | 0.37 (0.35-0.39) | 0.26 (0.23 to 0.3) | 15.57 (15.3-15.83) | 16.54 (16.37-16.72) | 0.3 (0.23 to 0.36) |
| Female | 0.03 (0.02-0.03) | 0.03 (0.02-0.03) | −0.01 (−0.07 to 0.04) | 1.45 (1.39-1.51) | 1.34 (1.3-1.38) | −0.28 (−0.34 to −0.22) |
| **High-income Asia Pacific** | | | | | | |
| Both | 0.06 (0.05-0.07) | 0.05 (0.05-0.06) | −0.38 (−0.78 to 0.03) | 2.68 (2.59-2.76) | 2.74 (2.66-2.82) | −0.26 (−0.69 to 0.18) |
| Male | 0.09 (0.08-0.11) | 0.09 (0.07-0.1) | −0.51 (−0.99 to −0.04) | 4.37 (4.22-4.53) | 4.43 (4.28-4.57) | −0.37 (−0.87 to 0.12) |
| Female | 0.01 (0.01-0.02) | 0.02 (0.01-0.02) | 0.63 (0.49 to 0.77) | 0.96 (0.9-1.02) | 0.97 (0.91-1.03) | 0.2 (0.03 to 0.36) |
| **Central Asia** | | | | | | |
| Both | 0.16 (0.13-0.19) | 0.11 (0.1-0.13) | −1.48 (−1.62 to −1.35) | 6.93 (6.62-7.23) | 5.53 (5.34-5.71) | −1.48 (−1.71 to −1.25) |
| Male | 0.28 (0.22-0.33) | 0.18 (0.15-0.21) | −1.73 (−1.82 to −1.65) | 12.07 (11.5-12.64) | 9.15 (8.81-9.49) | −1.7 (−1.93 to −1.47) |
| Female | 0.03 (0.02-0.04) | 0.03 (0.02-0.04) | −0.39 (−0.42 to −0.36) | 2.08 (1.89-2.27) | 2.02 (1.89-2.16) | −0.38 (−0.56 to −0.2) |
| **East Asia** | | | | | | |
| Both | 0.08 (0.07-0.08) | 0.05 (0.05-0.06) | −1.59 (−1.71 to −1.47) | 3.59 (3.54-3.64) | 2.54 (2.51-2.57) | −1.57 (−1.74 to −1.41) |
| Male | 0.15 (0.14-0.16) | 0.1 (0.1-0.11) | −1.47 (−1.58 to −1.36) | 6.66 (6.56-6.75) | 4.85 (4.79-4.91) | −1.46 (−1.62 to −1.3) |
| Female | 0 (0-0.01) | 0 (0-0) | −2.58 (−2.64 to −2.51) | 0.22 (0.21-0.24) | 0.14 (0.13-0.15) | −2.25 (−2.51 to −1.99) |
| **South Asia** | | | | | | |
| Both | 0.48 (0.46-0.49) | 0.59 (0.57-0.6) | 0.75 (0.69 to 0.82) | 21.67 (21.53-21.82) | 26.96 (26.85-27.07) | 0.81 (0.71 to 0.9) |
| Male | 0.86 (0.83-0.89) | 1.11 (1.08-1.13) | 0.96 (0.88 to 1.04) | 38.93 (38.67-39.2) | 50.83 (50.62-51.05) | 1.02 (0.92 to 1.12) |
| Female | 0.05 (0.05-0.06) | 0.05 (0.05-0.05) | −0.29 (−0.38 to −0.2) | 2.43 (2.36-2.49) | 2.37 (2.32-2.41) | −0.28 (−0.41 to −0.15) |
| **Southeast Asia** | | | | | | |
| Both | 0.1 (0.09-0.11) | 0.13 (0.12-0.14) | 0.8 (0.75 to 0.85) | 4.56 (4.47-4.65) | 5.76 (5.69-5.83) | 0.78 (0.72 to 0.84) |
| Male | 0.19 (0.18-0.21) | 0.24 (0.23-0.26) | 0.8 (0.74 to 0.87) | 8.77 (8.59-8.95) | 11.02 (10.88-11.16) | 0.79 (0.72 to 0.86) |
| Female | 0.01 (0.01-0.01) | 0.01 (0.01-0.01) | 0.22 (0.18 to 0.27) | 0.52 (0.48-0.55) | 0.48 (0.45-0.5) | −0.38 (−0.52 to −0.25) |
| **Australasia** | | | | | | |
| Both | 0.15 (0.11-0.19) | 0.12 (0.09-0.15) | −0.86 (−0.94 to −0.77) | 7.97 (7.56-8.37) | 5.95 (5.67-6.24) | −0.85 (−1 to −0.71) |
| Male | 0.25 (0.18-0.31) | 0.2 (0.15-0.25) | −0.72 (−0.82 to −0.62) | 12.72 (12-13.44) | 9.86 (9.34-10.38) | −0.76 (−0.92 to −0.6) |
| Female | 0.04 (0.02-0.07) | 0.03 (0.01-0.04) | −1.42 (−1.6 to −1.23) | 2.94 (2.66-3.23) | 2.2 (2-2.4) | −0.86 (−1.02 to −0.7) |
| **Caribbean** | | | | | | |
| Both | 0.16 (0.13-0.2) | 0.12 (0.1-0.15) | −0.92 (−1.16 to −0.69) | 8.3 (7.93-8.68) | 6.28 (6.02-6.53) | −0.89 (−1.24 to −0.55) |

| | | | | | | |
|---|---|---|---|---|---|---|
| Male | 0.28 (0.22-0.35) | 0.21 (0.17-0.26) | −0.91 (−1.21 to −0.62) | 14.39 (13.69-15.09) | 11 (10.53-11.48) | −0.85 (−1.23 to −0.47) |
| Female | 0.02 (0.01-0.04) | 0.02 (0.01-0.04) | 0.46 (0.39 to 0.52) | 2.42 (2.2-2.64) | 1.68 (1.54-1.82) | −1.26 (−1.41 to −1.11) |
| **Central Europe** | | | | | | |
| Both | 0.57 (0.53-0.61) | 0.47 (0.43-0.5) | −1.17 (−1.56 to −0.78) | 25.48 (25.1-25.87) | 21.73 (21.4-22.06) | −1.16 (−1.55 to −0.77) |
| Male | 1.08 (0.99-1.16) | 0.85 (0.79-0.92) | −1.29 (−1.7 to −0.89) | 47.74 (47-48.49) | 39.67 (39.03-40.3) | −1.28 (−1.68 to −0.87) |
| Female | 0.07 (0.05-0.08) | 0.06 (0.05-0.07) | −0.67 (−0.98 to −0.36) | 3.48 (3.32-3.63) | 3.27 (3.13-3.42) | −0.61 (−0.91 to −0.3) |
| **Eastern Europe** | | | | | | |
| Both | 0.36 (0.33-0.39) | 0.42 (0.39-0.45) | −0.13 (−0.42 to 0.15) | 16.17 (15.93-16.42) | 19.14 (18.9-19.39) | −0.02 (−0.36 to 0.31) |
| Male | 0.72 (0.65-0.78) | 0.79 (0.73-0.86) | −0.28 (−0.55 to −0.01) | 31.79 (31.28-32.29) | 36.29 (35.8-36.77) | −0.16 (−0.5 to 0.17) |
| Female | 0.04 (0.03-0.04) | 0.06 (0.05-0.08) | 2.15 (2.12 to 2.18) | 1.75 (1.65-1.85) | 3.19 (3.06-3.32) | 1.95 (1.68 to 2.21) |
| **Western Europe** | | | | | | |
| Both | 0.44 (0.42-0.46) | 0.18 (0.17-0.19) | −3.85 (−4.15 to −3.55) | 19.73 (19.53-19.92) | 8.55 (8.43-8.66) | −3.76 (−4.07 to −3.45) |
| Male | 0.81 (0.76-0.85) | 0.3 (0.28-0.33) | −4.15 (−4.46 to −3.84) | 35.92 (35.55-36.29) | 14.53 (14.32-14.74) | −4.08 (−4.4 to −3.75) |
| Female | 0.07 (0.06-0.08) | 0.05 (0.04-0.06) | −1.47 (−1.82 to −1.12) | 3.31 (3.22-3.41) | 2.53 (2.46-2.61) | −1.3 (−1.64 to −0.95) |
| **Andean Latin America** | | | | | | |
| Both | 0.05 (0.04-0.06) | 0.03 (0.02-0.03) | −2.1 (−2.43 to −1.77) | 2.3 (2.12-2.47) | 1.28 (1.19-1.37) | −2.05 (−2.33 to −1.76) |
| Male | 0.07 (0.05-0.09) | 0.04 (0.03-0.05) | −2.11 (−2.43 to −1.79) | 3.38 (3.1-3.67) | 1.88 (1.73-2.02) | −2.06 (−2.33 to −1.78) |
| Female | 0.03 (0-0.06) | 0.01 (0-0.05) | −2.01 (−2.37 to −1.65) | 1.23 (1.08-1.39) | 0.68 (0.61-0.76) | −1.99 (−2.26 to −1.72) |
| **Central Latin America** | | | | | | |
| Both | 0.05 (0.04-0.06) | 0.03 (0.02-0.03) | −1.8 (−1.92 to −1.68) | 2.27 (2.18-2.37) | 1.42 (1.37-1.47) | −1.77 (−1.94 to −1.61) |
| Male | 0.07 (0.05-0.09) | 0.05 (0.04-0.06) | −1.32 (−1.41 to −1.23) | 3.72 (3.55-3.88) | 2.5 (2.4-2.59) | −1.5 (−1.63 to −1.37) |
| Female | 0.01 (0.01-0.02) | 0 (0-0.01) | −3.11 (−3.15 to −3.07) | 0.89 (0.82-0.96) | 0.43 (0.4-0.46) | −2.71 (−2.94 to −2.47) |
| **Southern Latin America** | | | | | | |
| Both | 0.17 (0.14-0.2) | 0.05 (0.04-0.07) | −4.01 (−4.4 to −3.63) | 8.12 (7.81-8.43) | 2.96 (2.82-3.1) | −3.62 (−4.1 to −3.15) |
| Male | 0.31 (0.24-0.37) | 0.09 (0.07-0.12) | −4.18 (−4.58 to −3.78) | 14.76 (14.16-15.35) | 5.07 (4.82-5.33) | −3.87 (−4.36 to −3.39) |
| Female | 0.02 (0.01-0.03) | 0 (0-0.01) | −4.49 (−4.54 to −4.44) | 1.68 (1.52-1.84) | 0.92 (0.83-1.01) | −1.98 (−2.33 to −1.64) |
| **Tropical Latin America** | | | | | | |
| Both | 0.38 (0.35-0.42) | 0.27 (0.25-0.29) | −1.52 (−1.89 to −1.15) | 17.42 (17.1-17.73) | 12.57 (12.38-12.76) | −1.51 (−1.88 to −1.13) |
| Male | 0.7 (0.64-0.77) | 0.51 (0.47-0.55) | −1.45 (−1.83 to −1.07) | 31.95 (31.34-32.55) | 23.65 (23.28-24.02) | −1.45 (−1.84 to −1.06) |
| Female | 0.07 (0.05-0.08) | 0.04 (0.03-0.05) | −1.81 (−2.14 to −1.48) | 3.33 (3.17-3.48) | 2.08 (2-2.17) | −1.69 (−2.06 to −1.32) |

| | | | | | | |
|---|---|---|---|---|---|---|
| **North Africa and Middle East** | | | | | | |
| Both | 0.04 (0.03-0.04) | 0.03 (0.02-0.03) | −0.98 (−1.04 to −0.91) | 1.72 (1.66-1.77) | 1.34 (1.31-1.38) | −1.04 (−1.13 to −0.95) |
| Male | 0.06 (0.05-0.07) | 0.05 (0.04-0.05) | −0.74 (−0.85 to −0.63) | 2.68 (2.58-2.78) | 2.22 (2.16-2.28) | −0.82 (−0.94 to −0.7) |
| Female | 0.01 (0.01-0.02) | 0.01 (0-0.01) | −2.79 (−2.81 to −2.77) | 0.7 (0.65-0.74) | 0.34 (0.32-0.36) | −2.65 (−2.73 to −2.56) |
| **High-income North America** | | | | | | |
| Both | 0.11 (0.1-0.12) | 0.07 (0.06-0.08) | −1.52 (−1.55 to −1.49) | 5.39 (5.28-5.5) | 3.53 (3.45-3.61) | −1.42 (−1.47 to −1.36) |
| Male | 0.18 (0.16-0.2) | 0.11 (0.1-0.12) | −1.72 (−1.75 to −1.69) | 8.87 (8.67-9.07) | 5.65 (5.52-5.79) | −1.57 (−1.63 to −1.52) |
| Female | 0.04 (0.03-0.04) | 0.02 (0.02-0.03) | −1.14 (−1.26 to −1.03) | 1.99 (1.91-2.07) | 1.46 (1.4-1.52) | −0.83 (−0.95 to −0.71) |
| **Oceania** | | | | | | |
| Both | 0.04 (0.04-0.04) | 0.04 (0.04-0.04) | −0.3 (−0.43 to −0.18) | 1.78 (1.5-2.06) | 1.69 (1.5-1.89) | −0.16 (−0.28 to −0.04) |
| Male | 0.06 (0.06-0.06) | 0.06 (0.06-0.06) | −0.21 (−0.37 to −0.05) | 2.72 (2.26-3.19) | 2.75 (2.41-3.09) | 0 (−0.13 to 0.14) |
| Female | 0.02 (0.02-0.02) | 0.01 (0.01-0.01) | −0.47 (−0.6 to −0.33) | 0.5 (0.32-0.68) | 0.54 (0.41-0.68) | 0.4 (0.35 to 0.44) |
| **Central Sub-Saharan Africa** | | | | | | |
| Both | 0.05 (0.05-0.05) | 0.05 (0.05-0.05) | −0.2 (−0.56 to 0.16) | 2.4 (2.23-2.57) | 2.27 (2.16-2.38) | −0.16 (−0.51 to 0.19) |
| Male | 0.1 (0.1-0.1) | 0.09 (0.09-0.09) | −0.39 (−0.79 to 0) | 4.26 (3.94-4.59) | 3.87 (3.66-4.07) | −0.35 (−0.73 to 0.04) |
| Female | 0.02 (0.02-0.02) | 0.01 (0.01-0.01) | −0.04 (−0.32 to 0.23) | 0.67 (0.57-0.77) | 0.66 (0.59-0.72) | 0.06 (−0.16 to 0.29) |
| **Eastern Sub-Saharan Africa** | | | | | | |
| Both | 0.09 (0.08-0.11) | 0.11 (0.09-0.12) | 0.46 (0.41 to 0.5) | 4.42 (4.28-4.56) | 4.82 (4.72-4.92) | 0.34 (0.29 to 0.39) |
| Male | 0.16 (0.13-0.18) | 0.19 (0.16-0.21) | 0.59 (0.56 to 0.63) | 7.37 (7.11-7.63) | 8.29 (8.1-8.48) | 0.47 (0.4 to 0.54) |
| Female | 0.02 (0.02-0.03) | 0.03 (0.02-0.03) | 0.33 (0.28 to 0.38) | 1.54 (1.44-1.63) | 1.41 (1.35-1.48) | −0.41 (−0.47 to −0.35) |
| **Southern Sub-Saharan Africa** | | | | | | |
| Both | 0.13 (0.1-0.16) | 0.09 (0.07-0.1) | −1.53 (−1.65 to −1.41) | 6.46 (6.16-6.76) | 4.5 (4.31-4.68) | −1.59 (−1.77 to −1.41) |
| Male | 0.23 (0.18-0.29) | 0.16 (0.12-0.19) | −1.55 (−1.78 to −1.33) | 11.3 (10.75-11.86) | 8.12 (7.78-8.47) | −1.53 (−1.78 to −1.28) |
| Female | 0.01 (0-0.02) | 0.02 (0.01-0.02) | 0.67 (0.59 to 0.75) | 1.78 (1.6-1.95) | 0.96 (0.87-1.05) | −1.84 (−1.95 to −1.72) |
| **Western Sub-Saharan Africa** | | | | | | |
| Both | 0.02 (0.01-0.02) | 0.02 (0.02-0.02) | 0.32 (0.21 to 0.43) | 0.99 (0.94-1.05) | 1.02 (0.98-1.06) | −0.02 (−0.11 to 0.08) |
| Male | 0.03 (0.02-0.04) | 0.04 (0.03-0.04) | 0.6 (0.5 to 0.69) | 1.64 (1.54-1.73) | 1.85 (1.78-1.93) | 0.36 (0.26 to 0.46) |
| Female | 0.001 (0-0.003) | 0.002 (0.001-0.004) | 1.96 (1.78 to 2.14) | 0.26 (0.22-0.29) | 0.23 (0.21-0.26) | −0.38 (−0.42 to −0.35) |

**Supplementary Table S7. The temporal trend in the age-specific DALYs rate for early-onset Lip and oral cavity cancer attributable to tobacco and alcohol across age groups by SDI quintile, 1990-2019**

| Characteristics | All risk factors | | Smoking | | Chewing tobacco | | Alcohol use | |
|---|---|---|---|---|---|---|---|---|
| | Rate in 2019 per 100,000 No. (95% UI) | EAPC 1990~2019 No. (95% CI) | Rate in 2019 per 100,000 No. (95% UI) | EAPC 1990~2019 No. (95% CI) | Rate in 2019 per 100,000 No. (95% UI) | EAPC 1990~2019 No. (95% CI) | Rate in 2019 per 100,000 No. (95% UI) | EAPC 1990~2019 No. (95% CI) |
| **Global** | | | | | | | | |
| 15-19 years | 0.4 (0.3-0.5) | −0.4 (−0.5 to −0.3) | NA | NA | NA | NA | 0.4 (0.3-0.5) | −0.4 (−0.5 to −0.3) |
| 20-24 years | 1.6 (1.2-2.1) | 0.7 (0.6 to 0.9) | NA | NA | NA | NA | 1.6 (1.2-2.1) | 0.7 (0.6 to 0.9) |
| 25-29 years | 3.4 (2.5-4.3) | 1.1 (1 to 1.2) | NA | NA | NA | NA | 3.4 (2.5-4.3) | 1.1 (1 to 1.2) |
| 30-34 years | 11.7 (9.6-14.1) | 0.6 (0.6 to 0.7) | 1.5 (0.8-2.2) | −0.9 (−0.9 to −0.8) | 5.5 (3.7-7.4) | 1 (0.8 to 1.1) | 6.8 (5.1-8.6) | 0.9 (0.8 to 1) |
| 35-39 years | 24.2 (19.9-28.7) | 0.5 (0.4 to 0.6) | 5.8 (3.4-8.3) | −0.9 (−1 to −0.8) | 10.3 (7-14) | 1.4 (1.2 to 1.5) | 13.9 (10.7-17.3) | 0.7 (0.5 to 0.9) |
| 40-44 years | 45.6 (38-54.2) | −0.3 (−0.4 to −0.3) | 15.5 (9.6-21.2) | −1.4 (−1.5 to −1.3) | 17.3 (11.9-24) | 0.6 (0.5 to 0.7) | 25.2 (19.9-31.1) | −0.2 (−0.3 to −0.1) |
| 45-49 years | 71.9 (61.1-83.4) | −0.7 (−0.8 to −0.6) | 30.4 (20.7-40.1) | −1.4 (−1.6 to −1.3) | 23.8 (16.4-32.5) | 0.1 (0 to 0.2) | 39.4 (31.2-47.8) | −0.6 (−0.7 to −0.5) |
| **High SDI** | | | | | | | | |
| 15-19 years | 0.4 (0.3-0.5) | −2.8 (−3 to −2.6) | NA | NA | NA | NA | 0.4 (0.3-0.5) | −2.8 (−3 to −2.6) |
| 20-24 years | 1.2 (1-1.4) | −1.6 (−1.8 to −1.4) | NA | NA | NA | NA | 1.2 (1-1.4) | −1.6 (−1.8 to −1.4) |
| 25-29 years | 2.4 (2-2.8) | −1.1 (−1.2 to −0.9) | NA | NA | NA | NA | 2.4 (2-2.8) | −1.1 (−1.2 to −0.9) |
| 30-34 years | 5.3 (4.6-6.2) | −1 (−1.2 to −0.8) | 1.2 (0.7-1.8) | −1.7 (−1.9 to −1.4) | 0.2 (0.1-0.4) | 0.1 (0 to 0.2) | 4.6 (3.8-5.4) | −1 (−1.2 to −0.8) |
| 35-39 years | 12 (10.1-14) | −1.4 (−1.6 to −1.3) | 4.7 (2.8-6.5) | −1.9 (−2.1 to −1.8) | 0.4 (0.2-0.7) | −0.6 (−0.7 to −0.5) | 9.6 (7.8-11.4) | −1.5 (−1.6 to −1.4) |
| 40-44 years | 26 (22-30.4) | −2.2 (−2.4 to −2) | 12.7 (8.3-17.1) | −2.6 (−2.8 to −2.3) | 0.8 (0.5-1.4) | −1 (−1.1 to −0.9) | 19.8 (16-23.7) | −2.3 (−2.5 to −2.1) |
| 45-49 years | 45.9 (40.3-52.9) | −2.3 (−2.6 to −2.1) | 24.9 (17.6-31.8) | −2.7 (−3 to −2.3) | 1.4 (0.8-2.3) | −1.2 (−1.4 to −1) | 34.4 (28.8-40.7) | −2.4 (−2.7 to −2.1) |
| **High-middle SDI** | | | | | | | | |
| 15-19 years | 0.5 (0.4-0.7) | −0.9 (−1 to −0.7) | NA | NA | NA | NA | 0.5 (0.4-0.7) | −0.9 (−1 to −0.7) |
| 20-24 years | 1.4 (1.1-1.8) | −0.4 (−0.5 to −0.3) | NA | NA | NA | NA | 1.4 (1.1-1.8) | −0.4 (−0.5 to −0.3) |
| 25-29 years | 2.8 (2.2-3.5) | 0.4 (0.2 to 0.5) | NA | NA | NA | NA | 2.8 (2.2-3.5) | 0.4 (0.2 to 0.5) |
| 30-34 years | 7.7 (6.4-9.2) | 0.5 (0.3 to 0.7) | 1.6 (0.9-2.3) | −0.4 (−0.7 to −0.2) | 1.3 (0.7-2.2) | 1.6 (1.2 to 1.9) | 6.1 (4.8-7.4) | 0.6 (0.3 to 0.8) |
| 35-39 years | 17.3 (14.5-20.4) | −0.3 (−0.5 to 0) | 6.2 (3.8-8.4) | −1.1 (−1.4 to −0.8) | 2.7 (1.5-4.4) | 2 (1.7 to 2.3) | 13 (10.3-15.9) | −0.2 (−0.5 to 0.1) |

| | | | | | | | | |
|---|---|---|---|---|---|---|---|---|
| 40-44 years | 36 (30.6-41.9) | −1.3 (−1.5 to −1.2) | 16.4 (10.9-21.5) | −2.1 (−2.3 to −1.9) | 4.8 (2.7-8.1) | 1.2 (1 to 1.4) | 26.1 (21.3-31.3) | −1.3 (−1.5 to −1.1) |
| 45-49 years | 62.1 (53.2-71) | −1.6 (−1.8 to −1.3) | 33.1 (23.6-41.9) | −2.1 (−2.4 to −1.8) | 6.8 (3.9-10.8) | 0.5 (0.2 to 0.8) | 44.3 (36.2-52.2) | −1.6 (−1.9 to −1.3) |
| **Middle SDI** | | | | | | | | |
| 15-19 years | 0.4 (0.3-0.6) | 0.9 (0.7 to 1.1) | NA | NA | NA | NA | 0.4 (0.3-0.6) | 0.9 (0.7 to 1.1) |
| 20-24 years | 1.5 (1.1-2.1) | 1.3 (1.2 to 1.4) | NA | NA | NA | NA | 1.5 (1.1-2.1) | 1.3 (1.2 to 1.4) |
| 25-29 years | 3.1 (2.3-4.1) | 1.4 (1.2 to 1.6) | NA | NA | NA | NA | 3.1 (2.3-4.1) | 1.4 (1.2 to 1.6) |
| 30-34 years | 9.1 (7.1-11.3) | 0.7 (0.6 to 0.7) | 1.3 (0.7-1.9) | −0.9 (−1 to −0.7) | 3.2 (2-4.6) | 1.1 (0.9 to 1.2) | 6.3 (4.7-8.2) | 1 (0.9 to 1.1) |
| 35-39 years | 18.8 (14.9-23.4) | 0.9 (0.8 to 1) | 4.6 (2.7-6.6) | −0.6 (−0.7 to −0.5) | 6.5 (4-9.7) | 1.8 (1.6 to 2) | 12.4 (9.3-16) | 1.3 (1.2 to 1.4) |
| 40-44 years | 33.8 (26.9-41.6) | 0.5 (0.4 to 0.5) | 11.5 (6.9-16.2) | −0.7 (−0.8 to −0.5) | 10.3 (6.3-15.6) | 0.9 (0.7 to 1) | 21.5 (16.1-27.7) | 1 (0.9 to 1.1) |
| 45-49 years | 54.4 (44.3-66.4) | 0.4 (0.2 to 0.6) | 23.9 (15.6-32.7) | −0.3 (−0.5 to −0.1) | 14.1 (8.8-20.8) | 0.4 (0.1 to 0.6) | 33.4 (25.3-42.4) | 1.1 (0.9 to 1.3) |
| **Low-middle SDI** | | | | | | | | |
| 15-19 years | 0.4 (0.2-0.6) | 1.2 (1 to 1.3) | NA | NA | NA | NA | 0.4 (0.2-0.6) | 1.2 (1 to 1.3) |
| 20-24 years | 2.1 (1.3-3) | 1.9 (1.7 to 2.1) | NA | NA | NA | NA | 2.1 (1.3-3) | 1.9 (1.7 to 2.1) |
| 25-29 years | 4.8 (3.2-6.6) | 2 (1.8 to 2.2) | NA | NA | NA | NA | 4.8 (3.2-6.6) | 2 (1.8 to 2.2) |
| 30-34 years | 21.9 (16.8-27.7) | 0.6 (0.5 to 0.7) | 2 (1-3.1) | −0.8 (−0.9 to −0.7) | 14 (9.3-19.2) | 0.2 (0.1 to 0.4) | 9.8 (6.6-13.4) | 1.9 (1.9 to 2) |
| 35-39 years | 44 (34.6-55.4) | 0.7 (0.6 to 0.9) | 8.4 (4.7-12.9) | −0.6 (−0.8 to −0.5) | 25.8 (17.7-35) | 0.5 (0.3-0.6) | 20.6 (14.3-28) | 2.2 (2 to 2.4) |
| 40-44 years | 80.7 (62.6-101.3) | 0.1 (0 to 0.2) | 22.8 (13.4-33.7) | −0.8 (−0.9 to −0.7) | 44.9 (30.3-62.5) | −0.2 (−0.4-−0.1) | 34.6 (24.5-47) | 1.6 (1.5 to 1.8) |
| 45-49 years | 122.1 (97.9-152) | −0.1 (−0.2 to −0.1) | 42.7 (26.8-62) | −0.9 (−1 to −0.8) | 65.4 (46.1-87.8) | −0.4 (−0.5-−0.3) | 48.9 (34.3-65.8) | 1.4 (1.2 to 1.6) |
| **Low SDI** | | | | | | | | |
| 15-19 years | 0.3 (0.2-0.5) | 0.3 (0.1 to 0.4) | NA | NA | NA | NA | 0.3 (0.2-0.5) | 0.3 (0.1 to 0.4) |
| 20-24 years | 1.4 (0.9-1.9) | 1.2 (1 to 1.4) | NA | NA | NA | NA | 1.4 (0.9-1.9) | 1.2 (1 to 1.4) |
| 25-29 years | 3.1 (2-4.4) | 1.5 (1.3 to 1.7) | NA | NA | NA | NA | 3.1 (2-4.4) | 1.5 (1.3 to 1.7) |
| 30-34 years | 13.6 (10.1-17.9) | −0.1 (−0.2 to 0) | 1.3 (0.6-2.1) | −0.8 (−1 to −0.7) | 8.6 (5.6-12.4) | −0.4 (−0.6-−0.2) | 5.8 (3.8-7.9) | 0.8 (0.8 to 0.9) |
| 35-39 years | 27.8 (21.1-36.7) | 0.1 (0 to 0.3) | 5.2 (2.7-8) | −0.6 (−0.7 to −0.4) | 16.1 (10.5-23.7) | −0.2 (−0.4-0.1) | 12 (8-16.8) | 1.2 (1.1 to 1.3) |
| 40-44 years | 57.7 (43.4-74.9) | −0.3 (−0.5 to −0.1) | 15.6 (8.3-24.4) | −0.8 (−1 to −0.5) | 31.1 (20.5-45.6) | −0.6 (−0.9-−0.4) | 23.1 (15.9-31.6) | 0.6 (0.5 to 0.6) |
| 45-49 years | 96.4 (74.1-124.4) | −0.2 (−0.4 to −0.1) | 31.7 (17.9-48.1) | −0.6 (−0.9 to −0.4) | 49.7 (32.6-70.5) | −0.5 (−0.6-−0.3) | 37.1 (25.5-50.8) | 0.6 (0.5 to 0.7) |

The smoking and chewing tobacco risk factors were modeled with lower age restrictions of 30 years in the GBD 2019 study; thus, estimates were not produced for these risk factors in the age groups of 15-19 years, 20- 24 years, and 25-29 years.

**Supplementary Table S8. The temporal trend in the age-specific DALYs for early-onset Other pharyngeal cancer attributable to tobacco and alcohol across age groups by SDI quintile, 1990-2019**

| Characteristics | All risk factors | | Smoking | | Alcohol use | |
|---|---|---|---|---|---|---|
| | ASR in 2019 per 100,000 No. (95% UI) | EAPC 1990~2019 No. (95% CI) | ASR in 2019 per 100,000 No. (95% UI) | EAPC 1990~2019 No. (95% CI) | ASR in 2019 per 100,000 No. (95% UI) | EAPC 1990~2019 No. (95% CI) |
| **Global** | | | | | | |
| 15-19 years | NA | NA | NA | NA | NA | NA |
| 20-24 years | 0.8 (0.5-1.2) | 1.9 (1.7 to 2) | NA | NA | 0.8 (0.5-1.2) | 1.9 (1.7 to 2) |
| 25-29 years | 1.3 (0.9-1.7) | 1.6 (1.4 to 1.7) | NA | NA | 1.3 (0.9-1.7) | 1.6 (1.4 to 1.7) |
| 30-34 years | 3.4 (2.5-4.3) | 1.3 (1.1 to 1.4) | 0.9 (0.6-1.3) | −0.2 (−0.3 to −0.1) | 2.8 (2-3.7) | 1.7 (1.5 to 1.8) |
| 35-39 years | 9.1 (7.1-11.1) | 0.5 (0.3 to 0.7) | 4.1 (2.7-5.6) | −0.5 (−0.7 to −0.3) | 6.7 (4.9-8.7) | 1 (0.8 to 1.3) |
| 40-44 years | 22.2 (17.8-26.6) | −0.5 (−0.7 to −0.4) | 12.9 (9-16.8) | −1.2 (−1.4 to −1.1) | 14.9 (11-19) | −0.2 (−0.4 to 0) |
| 45-49 years | 41.1 (34.2-48.5) | −0.9 (−1.1 to −0.8) | 27.5 (20.8-34.2) | −1.4 (−1.5 to −1.2) | 25.8 (19.6-32.8) | −0.7 (−0.8 to −0.6) |
| **High SDI** | | | | | | |
| 15-19 years | NA | NA | NA | NA | NA | NA |
| 20-24 years | 0.4 (0.3-0.4) | −0.8 (−0.9 to −0.7) | NA | NA | 0.4 (0.3-0.4) | −0.8 (−0.9 to −0.7) |
| 25-29 years | 0.4 (0.4-0.5) | −0.9 (−1 to −0.9) | NA | NA | 0.4 (0.4-0.5) | −0.9 (−1 to −0.9) |
| 30-34 years | 1.2 (1-1.4) | −0.8 (−0.9 to −0.7) | 0.4 (0.3-0.6) | −1.2 (−1.3 to −1.1) | 1 (0.8-1.2) | −0.8 (−1 to −0.7) |
| 35-39 years | 4.2 (3.5-4.8) | −2.2 (−2.4 to −2.1) | 2.2 (1.6-2.9) | −2.5 (−2.7 to −2.4) | 3.1 (2.5-3.8) | −2.4 (−2.6 to −2.2) |
| 40-44 years | 13.4 (11.4-15.4) | −2.8 (−3 to −2.7) | 8.5 (6.3-10.5) | −3.1 (−3.3 to −2.9) | 9.6 (7.7-11.5) | −3 (−3.2 to −2.8) |
| 45-49 years | 32.5 (28.6-36.7) | −2.3 (−2.6 to −2.1) | 22.1 (17.4-26.7) | −2.5 (−2.8 to −2.3) | 23.2 (18.8-27.3) | −2.4 (−2.7 to −2.2) |
| **High-middle SDI** | | | | | | |
| 15-19 years | NA | NA | NA | NA | NA | NA |
| 20-24 years | 0.5 (0.4-0.6) | −0.8 (−1 to −0.6) | NA | NA | 0.5 (0.4-0.6) | −0.8 (−1 to −0.6) |
| 25-29 years | 0.7 (0.5-0.9) | −0.4 (−0.5 to −0.3) | NA | NA | 0.7 (0.5-0.9) | −0.4 (−0.5 to −0.3) |
| 30-34 years | 1.8 (1.5-2.1) | −0.7 (−0.8 to −0.5) | 0.6 (0.4-0.8) | −1.3 (−1.5 to −1.1) | 1.5 (1.2-1.8) | −0.5 (−0.7 to −0.3) |
| 35-39 years | 5.9 (5-6.8) | −1.4 (−1.7 to −1.1) | 3.2 (2.3-4) | −1.9 (−2.2 to −1.5) | 4.5 (3.6-5.4) | −1.2 (−1.6 to −0.9) |

| | | | | | | |
|---|---|---|---|---|---|---|
| 40-44 years | 16.7 (14.6-19) | −2.3 (−2.7 to −2) | 10.6 (8-13.2) | −2.7 (−3.1 to −2.4) | 12.2 (9.9-14.5) | −2.2 (−2.5 to −1.9) |
| 45-49 years | 35.8 (31.2-40.6) | −2.2 (−2.5 to −1.8) | 25 (19.9-30) | −2.4 (−2.8 to −2.1) | 25.4 (20.7-30) | −2 (−2.4 to −1.7) |
| **Middle SDI** | | | | | | |
| 15-19 years | NA | NA | NA | NA | NA | NA |
| 20-24 years | 0.7 (0.4-1) | 2 (1.9 to 2.2) | NA | NA | 0.7 (0.4-1) | 2 (1.9 to 2.2) |
| 25-29 years | 1 (0.7-1.3) | 1.7 (1.5 to 1.9) | NA | NA | 1 (0.7-1.3) | 1.7 (1.5 to 1.9) |
| 30-34 years | 2.4 (1.8-3.2) | 0.9 (0.8 to 1) | 0.5 (0.3-0.8) | −1.1 (−1.2 to −0.9) | 2.1 (1.5-2.9) | 1.4 (1.3 to 1.6) |
| 35-39 years | 6.8 (5.2-8.6) | 0.8 (0.6 to 1) | 2.7 (1.7-3.8) | −0.7 (−0.8 to −0.5) | 5.3 (3.8-7) | 1.6 (1.4 to 1.8) |
| 40-44 years | 15.8 (12.1-19.8) | 0.1 (0.1 to 0.2) | 8.2 (5.5-11) | −0.9 (−1 to −0.8) | 11.3 (8.3-14.7) | 1 (0.9 to 1.1) |
| 45-49 years | 28.3 (22.2-34.2) | −0.2 (−0.3 to 0) | 17.6 (12.7-22.5) | −0.8 (−1 to −0.7) | 18.7 (13.7-24.4) | 0.8 (0.6 to 0.9) |
| **Low-middle SDI** | | | | | | |
| 15-19 years | NA | NA | NA | NA | NA | NA |
| 20-24 years | 1.4 (0.8-2.3) | 2.8 (2.6 to 3) | NA | NA | 1.4 (0.8-2.3) | 2.8 (2.6 to 3) |
| 25-29 years | 2.5 (1.5-3.6) | 2.1 (2 to 2.3) | NA | NA | 2.5 (1.5-3.6) | 2.1 (2 to 2.3) |
| 30-34 years | 7.5 (5.2-10.2) | 2 (1.8 to 2.1) | 2 (1.1-3) | 0.4 (0.2 to 0.5) | 6.2 (3.9-8.7) | 2.7 (2.5 to 2.8) |
| 35-39 years | 18.8 (13.7-24.7) | 1.6 (1.5 to 1.8) | 8.4 (5.2-12.1) | 0.4 (0.2 to 0.6) | 13.4 (8.8-18.6) | 2.8 (2.5 to 3) |
| 40-44 years | 44.8 (33.2-57.7) | 0.9 (0.8 to 1) | 26.1 (16.6-35.9) | 0.1 (0 to 0.2) | 27.8 (18.7-39.2) | 2.1 (1.9 to 2.2) |
| 45-49 years | 76.9 (58.1-97.4) | 0.3 (0.2 to 0.4) | 52.4 (37-70.1) | −0.3 (−0.4 to −0.2) | 42 (28.1-59.6) | 1.6 (1.4 to 1.8) |
| **Low SDI** | | | | | | |
| 15-19 years | NA | NA | NA | NA | NA | NA |
| 20-24 years | 0.7 (0.4-1.3) | 2.6 (2.2 to 3) | NA | NA | 0.7 (0.4-1.3) | 2.6 (2.2 to 3) |
| 25-29 years | 1.2 (0.6-1.9) | 2.1 (1.8 to 2.4) | NA | NA | 1.2 (0.6-1.9) | 2.1 (1.8 to 2.4) |
| 30-34 years | 3 (1.9-4.6) | 0.9 (0.7 to 1) | 0.9 (0.4-1.5) | −0.2 (−0.3 to 0) | 2.4 (1.4-3.7) | 1.4 (1.3 to 1.5) |
| 35-39 years | 8.1 (5.4-11.8) | 1 (0.8 to 1.2) | 3.7 (2-5.9) | 0.2 (−0.1 to 0.4) | 5.7 (3.5-8.7) | 1.9 (1.6 to 2.1) |
| 40-44 years | 19.5 (13.3-26.9) | 0.1 (0 to 0.2) | 11 (6.6-16.7) | −0.5 (−0.7 to −0.4) | 12 (7.6-17.7) | 1 (0.9 to 1.1) |
| 45-49 years | 39.8 (28.6-53.6) | 0 (−0.1 to 0.1) | 25.6 (16.9-36.9) | −0.5 (−0.6 to −0.4) | 22.1 (14-31.9) | 1 (0.8 to 1.1) |

The smoking and chewing tobacco risk factors were modeled with lower age restrictions of 30 years in the GBD 2019 study; thus, estimates were not produced for these risk factors in the age groups of 15-19 years, 20- 24 years, and 25-29 years.

**Supplementary Table S9. Population attributable fraction (PAF) for early-onset Lip and oral cavity cancer DALYs by SDI quintile and age in 1990 and 2019**

| Characteristics | All risk factors | | Smoking | | Chewing tobacco | | Alcohol use | |
|---|---|---|---|---|---|---|---|---|
| | PAF (%) in 1990 | PAF (%) in 2019 | PAF (%) in 1990 | PAF (%) in 2019 | PAF (%) in 1990 | PAF (%) in 2019 | PAF (%) in 1990 | PAF (%) in 2019 |
| **Global** | | | | | | | | |
| 15-19 years | 8.3 (6.2-10.6) | 7.5 (5.3-10.1) | NA | NA | NA | NA | 8.3 (6.2-10.6) | 7.5 (5.3-10.1) |
| 20-24 years | 16.1 (12.4-19.8) | 16.6 (12.2-21.3) | NA | NA | NA | NA | 16.1 (12.4-19.8) | 16.6 (12.2-21.3) |
| 25-29 years | 21 (16.6-25.7) | 23.1 (17.4-28.7) | NA | NA | NA | NA | 21 (16.6-25.7) | 23.1 (17.4-28.7) |
| 30-34 years | 44.6 (38.9-50.4) | 45.7 (39.2-52) | 8.6 (5-12.4) | 5.8 (3.2-8.6) | 18.7 (13.2-24.1) | 21.5 (15-27.6) | 24.4 (19.3-29.4) | 26.4 (20.4-32) |
| 35-39 years | 54 (47.8-60) | 53 (46.6-59.2) | 18.9 (11.9-25.8) | 12.8 (7.7-17.8) | 18.5 (12.9-24.3) | 22.5 (15.8-29.4) | 29.7 (24.4-35) | 30.5 (24.3-36.6) |
| 40-44 years | 61.4 (54.9-67.2) | 57.3 (51.1-63.5) | 28 (19-35.9) | 19.5 (12.5-26) | 18.7 (13.1-24.3) | 21.7 (15.6-28.3) | 32.9 (27.1-38.2) | 31.7 (25.4-37.8) |
| 45-49 years | 65.4 (59.5-70.9) | 61.4 (55.5-67) | 34.6 (25-42.9) | 26 (17.7-33.3) | 17.8 (12.7-23.2) | 20.3 (14.4-26.1) | 34.5 (28.9-40.2) | 33.7 (27.4-39.5) |
| **High SDI** | | | | | | | | |
| 15-19 years | 42.2 (35.1-48.7) | 35.1 (28.2-41.5) | NA | NA | NA | NA | 42.2 (35.1-48.7) | 35.1 (28.2-41.5) |
| 20-24 years | 52.9 (45.3-59.4) | 46.8 (39.5-53.5) | NA | NA | NA | NA | 52.9 (45.3-59.4) | 46.8 (39.5-53.5) |
| 25-29 years | 54 (46.2-60.7) | 48 (40.5-55) | NA | NA | NA | NA | 54 (46.2-60.7) | 48 (40.5-55) |
| 30-34 years | 63.8 (57.2-69.7) | 54.7 (47.5-61) | 19 (11.3-26.4) | 12.7 (7.3-18.3) | 2.2 (1.3-3.5) | 2.3 (1.3-3.7) | 54.8 (47-61.3) | 47.3 (39.8-54.2) |
| 35-39 years | 71.4 (65.1-77.3) | 61.2 (53.9-67.6) | 32.9 (21.3-42.9) | 23.9 (15-32.5) | 1.9 (1.1-3.2) | 2.1 (1.2-3.4) | 57.2 (49.1-63.7) | 48.8 (41-55.6) |
| 40-44 years | 77 (70.8-82.1) | 67.5 (60.3-73.7) | 42.4 (30.2-53.3) | 32.9 (22-42.9) | 1.9 (1.1-3.1) | 2.2 (1.2-3.6) | 59.9 (52.3-66.4) | 51.3 (43.4-58.2) |
| 45-49 years | 79.8 (74.2-84.4) | 72 (65.4-77.6) | 48.7 (37.6-57.7) | 39 (28.4-48.7) | 1.8 (1.1-3) | 2.2 (1.3-3.6) | 60.6 (52.9-67.2) | 53.9 (45.9-60.8) |
| **High-middle SDI** | | | | | | | | |
| 15-19 years | 18.6 (14.4-22.6) | 19.2 (14.6-24) | NA | NA | NA | NA | 18.6 (14.4-22.6) | 19.2 (14.6-24) |
| 20-24 years | 29.6 (23.9-35.1) | 30.4 (24-36.9) | NA | NA | NA | NA | 29.6 (23.9-35.1) | 30.4 (24-36.9) |
| 25-29 years | 34.9 (28.7-41) | 37.2 (30.1-44.1) | NA | NA | NA | NA | 34.9 (28.7-41) | 37.2 (30.1-44.1) |
| 30-34 years | 49.9 (43.8-55.6) | 52.3 (45.6-58.4) | 13.2 (7.9-18.6) | 10.9 (6.3-15.4) | 6.2 (3.7-9.1) | 9.1 (5-13.9) | 39 (32.2-45) | 41.3 (33.9-47.9) |
| 35-39 years | 61.6 (55.2-67.3) | 61.5 (55.3-67.4) | 26.8 (17.4-35.2) | 22 (14-29.5) | 5.5 (3.3-8.1) | 9.4 (5.4-14.3) | 45.8 (38.9-52) | 46.2 (38.6-52.7) |
| 40-44 years | 70.3 (64.1-75.5) | 67.5 (61.3-72.9) | 38.4 (27-47.7) | 30.7 (20.8-39.8) | 5.6 (3.4-8.4) | 9 (5.3-14.2) | 50.6 (43.3-57) | 49 (41.4-55.6) |

| | | | | | | | | |
|---|---|---|---|---|---|---|---|---|
| 45-49 years | 74.4 (68.6-79.3) | 70.9 (64.7-76) | 45.3 (34.5-54.6) | 37.8 (27.4-46.7) | 5.3 (3.3-7.8) | 7.7 (4.5-11.5) | 52.6 (45.4-58.9) | 50.6 (43.2-57.1) |
| **Middle SDI** | | | | | | | | |
| 15-19 years | 7.5 (5.3-9.9) | 11 (7.8-14.5) | NA | NA | NA | NA | 7.5 (5.3-9.9) | 11 (7.8-14.5) |
| 20-24 years | 16.9 (12.7-21.2) | 23 (17.4-28.7) | NA | NA | NA | NA | 16.9 (12.7-21.2) | 23 (17.4-28.7) |
| 25-29 years | 21.7 (16.9-26.6) | 29.9 (23.5-36.1) | NA | NA | NA | NA | 21.7 (16.9-26.6) | 29.9 (23.5-36.1) |
| 30-34 years | 38.7 (32.8-44.3) | 47.5 (41-53.7) | 7.8 (4.4-11.4) | 6.6 (3.7-9.8) | 12.7 (8.3-17.5) | 16.6 (10.7-22.7) | 24.6 (19.1-29.9) | 32.8 (26.2-39.2) |
| 35-39 years | 47.5 (41.2-53.8) | 54.1 (47.5-60.5) | 17.1 (10.6-23.3) | 13.2 (7.8-18.5) | 13.5 (8.5-19.5) | 18.7 (12-26) | 28.3 (22.7-34.2) | 35.7 (28.6-42.3) |
| 40-44 years | 54.6 (47.7-60.7) | 57.8 (51.1-64) | 25 (16.4-32.9) | 19.7 (12.6-26.5) | 15.1 (9.4-21.5) | 17.6 (11.5-24.9) | 30.2 (23.9-36.2) | 36.8 (29.9-43.4) |
| 45-49 years | 57.6 (50.9-64) | 61 (54.8-66.7) | 30.9 (21.4-39.3) | 26.7 (18.4-34.6) | 14.9 (9.5-21.2) | 15.7 (10-21.8) | 29.8 (23.8-35.7) | 37.4 (30.4-43.9) |
| **Low-middle SDI** | | | | | | | | |
| 15-19 years | 2.9 (1.7-4.5) | 4.3 (2.6-6.4) | NA | NA | NA | NA | 2.9 (1.7-4.5) | 4.3 (2.6-6.4) |
| 20-24 years | 8.4 (5.4-12.1) | 12 (7.9-16.6) | NA | NA | NA | NA | 8.4 (5.4-12.1) | 12 (7.9-16.6) |
| 25-29 years | 11.9 (8-16.4) | 17.7 (12.2-23.8) | NA | NA | NA | NA | 11.9 (8-16.4) | 17.7 (12.2-23.8) |
| 30-34 years | 43.6 (36.1-51.8) | 44.8 (36.8-52.6) | 5.9 (3.1-9.2) | 4.1 (2.2-6.3) | 30.2 (21.4-39.2) | 28.6 (20-36.9) | 13.9 (9.6-19.2) | 20.2 (14.5-26.8) |
| 35-39 years | 51.3 (42.9-59.6) | 52 (44.2-59.6) | 13.8 (8.1-20) | 9.9 (5.6-14.2) | 31.8 (22.6-41.6) | 30.5 (21.8-39.5) | 17.1 (12.2-22.8) | 24.4 (18.1-30.9) |
| 40-44 years | 56.9 (48.7-64.6) | 55.2 (47.9-62.6) | 20.9 (13.3-29) | 15.6 (9.6-21.7) | 33.6 (23.9-43.5) | 30.7 (22.3-39.8) | 16.9 (12-22) | 23.7 (17.7-30.5) |
| 45-49 years | 60.5 (53.1-68) | 58.8 (51.4-65.7) | 26.7 (18-35.3) | 20.5 (13.4-27.6) | 33.6 (24.4-43.1) | 31.5 (22.9-39.8) | 16.8 (11.9-22.8) | 23.6 (17.8-29.8) |
| **Low SDI** | | | | | | | | |
| 15-19 years | 4.4 (2.6-6.5) | 5 (2.9-7.4) | NA | NA | NA | NA | 4.4 (2.6-6.5) | 5 (2.9-7.4) |
| 20-24 years | 9.7 (6.2-13.9) | 11.9 (7.7-16.5) | NA | NA | NA | NA | 9.7 (6.2-13.9) | 11.9 (7.7-16.5) |
| 25-29 years | 12.3 (8.2-17.1) | 15.6 (10.6-21) | NA | NA | NA | NA | 12.3 (8.2-17.1) | 15.6 (10.6-21) |
| 30-34 years | 40 (32.4-47.6) | 38.6 (31.1-46.6) | 4.8 (2.4-7.6) | 3.6 (1.8-5.6) | 27.1 (19.4-35.6) | 24.3 (17-32.5) | 13.5 (9.2-17.9) | 16.4 (11.1-21.9) |
| 35-39 years | 46.3 (38.9-53.5) | 44.2 (36.6-51.7) | 10.6 (6.2-15.6) | 8.2 (4.6-12.2) | 28.8 (20.7-37.2) | 25.5 (17.5-34.1) | 15.5 (10.6-20.8) | 19.1 (13-25.3) |
| 40-44 years | 50.8 (43-57.9) | 48.1 (40.4-56.1) | 16.4 (10.3-22.5) | 12.9 (7.6-18.7) | 29.2 (20.9-37.9) | 25.9 (17.8-34.4) | 16.2 (11.5-21.2) | 19.2 (14-25.2) |
| 45-49 years | 53.8 (46.1-60.8) | 51.3 (43.9-58.3) | 20.5 (12.8-27.6) | 16.8 (10.4-23.6) | 29.2 (21.3-37.7) | 26.5 (18.6-34.5) | 16.8 (12.1-22) | 19.8 (13.9-25.5) |

The smoking and chewing tobacco risk factors were modeled with lower age restrictions of 30 years in the GBD 2019 study; thus, estimates were not produced for these risk factors in the age groups of 15-19 years, 20- 24 years, and 25-29 years.

**Supplementary Table S10. Population attributable fraction (PAF) for early-onset other pharyngeal cancer DALYs by SDI quintile and age in 1990 and 2019**

| Characteristics | | All risk factors | | Smoking | | Alcohol use | |
|---|---|---|---|---|---|---|---|
| | | PAF (%) in 1990 | PAF (%) in 2019 | PAF (%) in 1990 | PAF (%) in 2019 | PAF (%) in 1990 | PAF (%) in 2019 |
| **Global** | | | | | | | |
| | 15-19 years | NA | NA | NA | NA | NA | NA |
| | 20-24 years | 13.5 (9.5-17.9) | 17.3 (11.8-23.5) | NA | NA | 13.5 (9.5-17.9) | 17.3 (11.8-23.5) |
| | 25-29 years | 15.3 (10.9-20.4) | 20.8 (14.8-27.1) | NA | NA | 15.3 (10.9-20.4) | 20.8 (14.8-27.1) |
| | 30-34 years | 27 (21.5-33) | 32.4 (25.4-39.6) | 10.6 (6.7-14.3) | 8.7 (5.4-12.4) | 20.2 (14.5-25.9) | 27 (19.9-34.8) |
| | 35-39 years | 42.6 (36.2-49.1) | 41.9 (34.7-48.7) | 25.1 (17.7-32) | 18.9 (12.7-25.3) | 27.8 (21.3-34.4) | 30.8 (23.2-38.4) |
| | 40-44 years | 56.7 (49.5-62.7) | 51.2 (44-58) | 39.6 (30.1-47.6) | 29.6 (21.6-37) | 34.5 (27.5-41.5) | 34.3 (26.1-41.6) |
| | 45-49 years | 62.8 (56.5-68.2) | 57.8 (50.9-64.4) | 47.6 (38.5-55) | 38.6 (29.8-46.7) | 36.1 (28.8-43.1) | 36.2 (28.2-43.8) |
| **High SDI** | | | | | | | |
| | 15-19 years | NA | NA | NA | NA | NA | NA |
| | 20-24 years | 53.4 (43.9-61.7) | 48 (38.3-56.4) | NA | NA | 53.4 (43.9-61.7) | 48 (38.3-56.4) |
| | 25-29 years | 54.3 (45.2-62.4) | 47.8 (38.4-55.8) | NA | NA | 54.3 (45.2-62.4) | 47.8 (38.4-55.8) |
| | 30-34 years | 68 (60.1-74.5) | 58.3 (50.3-65.2) | 28.8 (19.8-37.3) | 21.1 (14-28.1) | 56 (46.7-64.3) | 48 (38.9-56.2) |
| | 35-39 years | 78.6 (72.2-84.1) | 68.8 (61.3-75) | 46.6 (35-56.6) | 37 (26.6-46.3) | 60.6 (51.5-68.8) | 51.6 (42-60) |
| | 40-44 years | 83.6 (78-88.2) | 75.7 (68.8-81.6) | 57.2 (45.6-66.4) | 47.9 (36.7-57.6) | 62.4 (53.3-70.6) | 54.6 (45-62.9) |
| | 45-49 years | 86 (81.2-89.9) | 80 (74-84.9) | 63.1 (53.7-71.1) | 54.4 (44.4-63.2) | 62.7 (53.7-71) | 57.2 (47.7-65.4) |
| **High-middle SDI** | | | | | | | |
| | 15-19 years | NA | NA | NA | NA | NA | NA |
| | 20-24 years | 32.5 (24.9-39.7) | 33 (25.1-40.9) | NA | NA | 32.5 (24.9-39.7) | 33 (25.1-40.9) |
| | 25-29 years | 35.9 (28.4-43.2) | 37.5 (29.3-45) | NA | NA | 35.9 (28.4-43.2) | 37.5 (29.3-45) |
| | 30-34 years | 51.5 (43.8-58) | 50.4 (42.6-57.5) | 21.4 (14.6-28.2) | 17.5 (11.7-23.4) | 41 (32.6-48.6) | 42.1 (33.8-50.3) |
| | 35-39 years | 66.9 (59.7-73) | 63.2 (55.6-69.5) | 40.1 (30-48.9) | 33.7 (24.7-42.1) | 48.6 (39.6-56.9) | 48.2 (39.4-56.2) |
| | 40-44 years | 76.4 (70.1-81.5) | 71.7 (64.4-77.5) | 53.8 (43-62.4) | 45.5 (35.1-54.3) | 53.4 (44.4-61.7) | 52.4 (43.5-60.6) |

| | | | | | | | |
|---|---|---|---|---|---|---|---|
| | 45-49 years | 80.3 (74.8-84.7) | 76.3 (70.1-81.3) | 60.6 (50.9-68.3) | 53.4 (43.6-61.4) | 54.9 (46-63.1) | 54.2 (45.8-62.5) |
| **Middle SDI** | | | | | | | |
| | 15-19 years | NA | NA | NA | NA | NA | NA |
| | 20-24 years | 15.4 (10.7-20.9) | 20.6 (14-28.3) | NA | NA | 15.4 (10.7-20.9) | 20.6 (14-28.3) |
| | 25-29 years | 17 (11.9-22.7) | 23.7 (17.1-30.7) | NA | NA | 17 (11.9-22.7) | 23.7 (17.1-30.7) |
| | 30-34 years | 28.2 (22.4-34.6) | 34.6 (27.1-42.4) | 10 (6.4-13.8) | 7.5 (4.7-10.6) | 21.7 (15.8-28.1) | 30.2 (22.4-38.5) |
| | 35-39 years | 41.7 (35-48.4) | 43.9 (36.4-51.5) | 23.8 (16.5-30.6) | 17.3 (11.6-23) | 26.8 (20.1-33.7) | 34.3 (26-42.4) |
| | 40-44 years | 52.4 (44.8-59.3) | 51.9 (44.3-58.7) | 35.5 (26.2-43.3) | 26.9 (19.2-34.1) | 30.4 (22.9-37.9) | 37.3 (28.7-45.3) |
| | 45-49 years | 57.1 (49.7-63.2) | 57.4 (49.9-64) | 42.7 (33.4-50.6) | 35.8 (27.2-43.6) | 29.9 (22.4-37.5) | 38.1 (29.9-46.3) |
| **Low-middle SDI** | | | | | | | |
| | 15-19 years | NA | NA | NA | NA | NA | NA |
| | 20-24 years | 7.6 (4.3-11.9) | 13.8 (8.5-20.6) | NA | NA | 7.6 (4.3-11.9) | 13.8 (8.5-20.6) |
| | 25-29 years | 9.5 (5.9-14.5) | 17.3 (11.3-23.7) | NA | NA | 9.5 (5.9-14.5) | 17.3 (11.3-23.7) |
| | 30-34 years | 18.6 (13-24.5) | 28.7 (21.3-36.7) | 7.3 (4.3-10.6) | 7.6 (4.5-11.1) | 12.9 (7.9-18.6) | 23.5 (16.2-31.8) |
| | 35-39 years | 28.9 (22.5-36.4) | 36.6 (28.7-44.6) | 17.4 (11.8-23.6) | 16.3 (10.7-22.6) | 15.9 (10.5-22.2) | 26 (18.2-34) |
| | 40-44 years | 41.6 (33.6-49.2) | 44.9 (36.7-52.5) | 29.9 (21.3-38) | 26.2 (18-33.7) | 19.3 (13.1-25.8) | 27.8 (19.9-35.8) |
| | 45-49 years | 47.8 (39.5-55.2) | 49.5 (41.8-57.2) | 37.9 (28.5-45.7) | 33.7 (25.2-42.1) | 19.1 (12.3-26) | 27 (19.1-35.6) |
| **Low SDI** | | | | | | | |
| | 15-19 years | NA | NA | NA | NA | NA | NA |
| | 20-24 years | 10.1 (5.6-16.2) | 18.2 (10.8-27.3) | NA | NA | 10.1 (5.6-16.2) | 18.2 (10.8-27.3) |
| | 25-29 years | 11.6 (6.5-18.4) | 20.9 (13-29.8) | NA | NA | 11.6 (6.5-18.4) | 20.9 (13-29.8) |
| | 30-34 years | 20.9 (14.2-29.4) | 29.9 (21.6-38.5) | 8 (4.4-12.6) | 8.8 (5.1-13.1) | 14.6 (8.4-22.4) | 24 (15.6-32.8) |
| | 35-39 years | 29.8 (21.6-38.7) | 36.5 (27.8-45.2) | 17 (10.4-24.4) | 16.4 (10.7-22.7) | 16.9 (10.3-25.1) | 25.6 (17.3-34.2) |
| | 40-44 years | 40.8 (32.5-49.3) | 43.3 (34.7-52) | 27.9 (19.2-36.8) | 24.5 (16.6-32.4) | 19.6 (12.8-27.6) | 26.7 (18.3-35) |
| | 45-49 years | 46.9 (38.6-55.5) | 48.4 (40.2-56.3) | 34.8 (25.4-43.7) | 31.2 (22.7-39.6) | 20.4 (13.1-28.3) | 26.9 (18.8-35.5) |

The smoking and chewing tobacco risk factors were modeled with lower age restrictions of 30 years in the GBD 2019 study; thus, estimates were not produced for these risk factors in the age groups of 15-19 years, 20- 24 years, and 25-29 years.

**Supplementary Table S11. Global and regional age-specific summary exposure value (SEV) of alcohol use among individuals aged 15-49 years, for both sexes, in 1990 and 2019, with the annualized rate of change (ARC) from 1990 to 2019**

| Characteristics | Male | | | Female | | |
|---|---|---|---|---|---|---|
| | Age-specific SEV in 1990 (95% UI) | Age-specific SEV in 2019 (95% UI) | ARC (%) (95% UI) | Age-specific SEV in 1990 (95% UI) | Age-specific SEV in 2019 (95% UI) | ARC (%) (95% UI) |
| **Overall** | 10.7 (7.6-14.5) | 11.1 (8.1-14.9) | 0.04 (−0.01 to 0.1) | 3.8 (2.6-5.6) | 3.3 (2.2-4.9) | −0.13 (−0.17 to −0.09) |
| **Socio-demographic index** | | | | | | |
| High | 19.4 (14.2-25.9) | 17.4 (12.5-23.2) | −0.11 (−0.14 to −0.07) | 12 (8.3-17.3) | 10.9 (7.5-15.6) | −0.09 (−0.14 to −0.05) |
| High -middle | 14.2 (10.2-19) | 15.1 (11.1-20) | 0.06 (−0.01 to 0.14) | 4.9 (3.3-7.3) | 4.6 (3.1-6.9) | −0.07 (−0.13 to 0.01) |
| Middle | 8.7 (6.1-12) | 11.1 (8.1-14.7) | 0.29 (0.18 to 0.41) | 1.4 (0.9-2.2) | 1.9 (1.2-2.8) | 0.34 (0.21 to 0.52) |
| Low-middle | 5.5 (3.8-7.9) | 7.5 (5.4-10.2) | 0.37 (0.21 to 0.57) | 0.9 (0.6-1.4) | 1.3 (0.8-1.9) | 0.41 (0.27 to 0.6) |
| Low | 5 (3.4-7.4) | 6 (4.2-8.6) | 0.2 (0.08 to 0.36) | 1.8 (1.1-3) | 2.1 (1.3-3.3) | 0.14 (0.03 to 0.3) |
| **GBD regions** | | | | | | |
| High-income Asia Pacific | 18.7 (13.3-25.5) | 19.2 (13.7-25.9) | 0.03 (−0.07 to 0.14) | 12.2 (8.3-17.4) | 12.5 (8.7-17.8) | 0.03 (−0.1 to 0.17) |
| Central Asia | 11.9 (8.3-16.4) | 12.6 (9-17.2) | 0.06 (−0.05 to 0.19) | 3.7 (2.2-5.8) | 3.4 (2.1-5.5) | −0.07 (−0.25 to 0.14) |
| East Asia | 10.4 (7.2-14.6) | 14.8 (10.7-19.7) | 0.42 (0.22 to 0.69) | 0.8 (0.5-1.4) | 1.4 (0.9-2.2) | 0.7 (0.21 to 1.49) |
| South Asia | 3.9 (2.5-5.7) | 6.2 (4.4-8.5) | 0.59 (0.23 to 1.07) | 0.2 (0.1-0.4) | 0.4 (0.2-0.6) | 0.96 (0.3 to 2.09) |
| Southeast Asia | 6 (4-8.8) | 10.1 (7-14) | 0.68 (0.54 to 0.87) | 1 (0.6-1.7) | 1.7 (1-2.6) | 0.63 (0.4 to 0.92) |
| Australasia | 22.8 (16.7-30.1) | 21.9 (15.7-29.4) | −0.04 (−0.12 to 0.05) | 13.2 (8.8-19.3) | 12 (7.7-18.3) | −0.09 (−0.23 to 0.05) |
| Caribbean | 13.4 (9.4-18.6) | 14.5 (10.5-19.8) | 0.09 (0.02 to 0.16) | 4.7 (3-7.4) | 4.8 (3.1-7.4) | 0.03 (−0.08 to 0.15) |
| Central Europe | 20.6 (15.3-27.2) | 22.1 (16.4-28.8) | 0.07 (0.03 to 0.12) | 7.5 (4.7-11.8) | 8.1 (5.2-12.7) | 0.08 (−0.01 to 0.2) |
| Eastern Europe | 19.7 (14.4-26.3) | 22.1 (16.3-28.7) | 0.12 (0.04 to 0.22) | 9.8 (6.5-14.5) | 11.4 (7.5-16.4) | 0.16 (0 to 0.36) |

| | | | | | | |
|---|---|---|---|---|---|---|
| Western Europe | 23.2 (17.2-30.3) | 20.5 (14.8-27.5) | −0.11 (−0.16 to −0.07) | 13.1 (9-18.8) | 11 (7.3-16.4) | −0.16 (−0.23 to −0.1) |
| Andean Latin America | 12.7 (8.5-18.2) | 14.8 (10.4-20.5) | 0.17 (0 to 0.38) | 4.4 (2.5-7.5) | 5.2 (3.1-8.6) | 0.19 (−0.07 to 0.57) |
| Central Latin America | 14.4 (10.5-19) | 14.5 (10.6-19) | 0 (−0.05 to 0.06) | 4.2 (2.6-6.7) | 4.2 (2.6-6.4) | −0.02 (−0.14 to 0.14) |
| Southern Latin America | 21.7 (15.8-29.1) | 19.1 (13.3-26.7) | −0.12 (−0.21 to −0.04) | 12.8 (8.6-18.5) | 10.8 (6.9-16.4) | −0.15 (−0.27 to −0.04) |
| Tropical Latin America | 13.1 (9.3-17.7) | 15.4 (11.2-20.2) | 0.18 (0.07 to 0.32) | 4.8 (3.1-7.2) | 6.4 (4.1-9.4) | 0.32 (0.06 to 0.65) |
| North Africa and Middle East | 2.2 (1.5-3.2) | 2.2 (1.5-3.2) | 0 (−0.09 to 0.09) | 0.7 (0.4-1.1) | 0.6 (0.3-0.9) | −0.15 (−0.29 to 0.02) |
| High-income North America | 18 (13.1-24.2) | 17.1 (12.2-23.1) | −0.05 (−0.13 to 0.03) | 12.5 (8.7-17.6) | 12.5 (8.6-17.3) | −0.01 (−0.1 to 0.09) |
| Oceania | 6.7 (4.1-10.6) | 6 (3.4-10) | −0.11 (−0.3 to 0.1) | 0.9 (0.4-1.6) | 0.8 (0.4-1.6) | −0.07 (−0.32 to 0.28) |
| Central Sub-Saharan Africa | 6.9 (4.1-10.9) | 7.8 (4.9-11.9) | 0.13 (−0.11 to 0.49) | 3.7 (2.1-6.4) | 4.2 (2.5-6.7) | 0.13 (−0.12 to 0.51) |
| Eastern Sub-Saharan Africa | 7.5 (5.2-10.8) | 7.9 (5.4-11.3) | 0.05 (−0.04 to 0.15) | 2.9 (1.9-4.6) | 3.1 (2-4.8) | 0.06 (−0.08 to 0.25) |
| Southern Sub-Saharan Africa | 14.5 (11-18.6) | 14 (10.4-18.2) | −0.03 (−0.11 to 0.05) | 4.1 (2.8-5.8) | 4.1 (2.8-6) | 0.01 (−0.18 to 0.24) |
| Western Sub-Saharan Africa | 7.3 (5-9.9) | 8.1 (5.9-10.8) | 0.12 (−0.02 to 0.32) | 2.4 (1.5-3.9) | 2.7 (1.7-4.2) | 0.12 (−0.07 to 0.37) |

**Supplementary Table S12. Global and regional age-specific smoking summary exposure value (SEV) among individuals aged 15-49 years, for both sexes, in 1990 and 2019, with the annualized rate of change (ARC) from 1990 to 2019**

| Characteristics | Male | | | Female | | |
|---|---|---|---|---|---|---|
| | Age-specific SEV in 1990 (95% UI) | Age-specific SEV in 2019 (95% UI) | ARC (%) (95% UI) | Age-specific SEV in 1990 (95% UI) | Age-specific SEV in 2019 (95% UI) | ARC (%) (95% UI) |
| **Overall** | 18.5 (15.9-21.3) | 13.9 (11.9-16.2) | −0.25 (−0.27 to −0.23) | 4.8 (4.1-5.6) | 2.7 (2.2-3.2) | −0.44 (−0.47 to −0.42) |
| **Socio-demographic index** | | | | | | |
| High | 23.5 (20.4-27.1) | 15.6 (13.3-18.3) | −0.33 (−0.36 to −0.31) | 13.5 (11.4-15.6) | 8.2 (6.9-9.8) | −0.39 (−0.42 to −0.35) |
| High -middle | 22.3 (19.1-25.7) | 18.6 (15.9-21.6) | −0.16 (−0.19 to −0.14) | 6.2 (5.1-7.4) | 4.7 (3.9-5.5) | −0.24 (−0.28 to −0.2) |
| Middle | 18.2 (15.5-21.1) | 15.1 (12.8-17.6) | −0.17 (−0.2 to −0.14) | 1.5 (1.3-1.8) | 1.1 (0.9-1.3) | −0.31 (−0.38 to −0.24) |
| Low-middle | 13.6 (11.5-15.9) | 10.3 (8.7-12) | −0.24 (−0.27 to −0.21) | 1.3 (1-1.6) | 0.9 (0.7-1.1) | −0.32 (−0.41 to −0.22) |
| Low | 7.5 (6.3-8.8) | 6 (5-7.1) | −0.2 (−0.24 to −0.17) | 1 (0.8-1.2) | 0.8 (0.6-0.9) | −0.2 (−0.29 to −0.09) |
| **GBD regions** | | | | | | |
| High-income Asia Pacific | 29.6 (25.2-34.6) | 19.2 (15.9-23) | −0.35 (−0.4 to −0.3) | 5.5 (4.5-6.8) | 4.8 (3.8-5.9) | −0.14 (−0.25 to 0) |
| Central Asia | 19.7 (16.8-22.9) | 16.5 (14.1-19.3) | −0.16 (−0.21 to −0.11) | 1.6 (1.2-2) | 1.5 (1.2-1.8) | −0.05 (−0.21 to 0.13) |
| East Asia | 22.2 (18.8-25.8) | 21.4 (18.3-24.5) | −0.04 (−0.09 to 0.03) | 0.7 (0.5-1) | 1 (0.8-1.3) | 0.37 (0 to 0.88) |
| South Asia | 10.6 (8.8-12.6) | 7.5 (6.2-8.9) | −0.3 (−0.34 to −0.24) | 0.8 (0.6-1) | 0.6 (0.4-0.7) | −0.28 (−0.45 to −0.05) |
| Southeast Asia | 18.6 (15.6-21.8) | 16.9 (14-20.1) | −0.09 (−0.14 to −0.05) | 1 (0.8-1.2) | 0.8 (0.7-1) | −0.17 (−0.29 to −0.03) |
| Australasia | 19.4 (16.1-23.1) | 12.6 (10.4-15) | −0.35 (−0.4 to −0.3) | 15 (12.3-18.1) | 9.5 (7.7-11.7) | −0.37 (−0.43 to −0.3) |
| Caribbean | 11.3 (9.5-13.2) | 8.1 (6.6-9.5) | −0.28 (−0.34 to −0.23) | 4.6 (3.6-5.7) | 3.1 (2.5-3.8) | −0.33 (−0.44 to −0.19) |
| Central Europe | 25.2 (21.6-29.1) | 19.3 (16.4-22.5) | −0.23 (−0.27 to −0.2) | 14.3 (11.9-17) | 11.4 (9.4-13.5) | −0.21 (−0.26 to −0.15) |
| Eastern Europe | 26.3 (22.3-30.7) | 26 (22.1-30.3) | −0.01 (−0.07 to 0.05) | 5.8 (4.5-7.4) | 8.4 (6.9-10) | 0.44 (0.24 to 0.68) |

| | | | | | | |
|---|---|---|---|---|---|---|
| Western Europe | 23.4 (20.1-27.1) | 16.3 (13.7-19.3) | −0.3 (−0.34 to −0.27) | 16.6 (14-19.6) | 11.8 (9.7-14.1) | −0.29 (−0.33 to −0.25) |
| Andean Latin America | 4.9 (3.9-6) | 3.6 (2.8-4.5) | −0.27 (−0.35 to −0.18) | 1.3 (1-1.7) | 1 (0.8-1.2) | −0.23 (−0.37 to −0.06) |
| Central Latin America | 11.7 (9.4-14.1) | 6 (4.8-7.3) | −0.48 (−0.53 to −0.44) | 3.6 (2.8-4.5) | 1.8 (1.4-2.2) | −0.51 (−0.57 to −0.43) |
| Southern Latin America | 18.6 (15.3-22.1) | 14.4 (11.8-17) | −0.23 (−0.29 to −0.16) | 12.9 (10.2-16.1) | 9.9 (8-12) | −0.23 (−0.34 to −0.12) |
| Tropical Latin America | 17.2 (14.2-20.8) | 8.1 (6.6-9.8) | −0.53 (−0.58 to −0.47) | 12.5 (10-15.4) | 4.5 (3.6-5.6) | −0.64 (−0.69 to −0.58) |
| North Africa and Middle East | 19.2 (16.6-22) | 15.9 (13.6-18.4) | −0.17 (−0.21 to −0.13) | 2.2 (1.7-2.7) | 1.9 (1.5-2.2) | −0.14 (−0.27 to 0.01) |
| High-income North America | 22.4 (19.5-25.5) | 14.2 (12-16.6) | −0.37 (−0.42 to −0.32) | 17.4 (14.8-20.2) | 9.9 (8.4-11.7) | −0.43 (−0.48 to −0.37) |
| Oceania | 13.9 (11-17.1) | 11.8 (9.1-14.8) | −0.15 (−0.27 to −0.02) | 4.7 (3.4-6.1) | 3.7 (2.8-4.8) | −0.2 (−0.38 to 0) |
| Central Sub-Saharan Africa | 6.2 (5.1-7.4) | 5.2 (4.2-6.2) | −0.17 (−0.25 to −0.08) | 0.6 (0.4-0.7) | 0.5 (0.4-0.6) | −0.16 (−0.33 to 0.03) |
| Eastern Sub-Saharan Africa | 5.8 (4.8-6.9) | 4.6 (3.8-5.5) | −0.21 (−0.25 to −0.17) | 0.7 (0.5-0.8) | 0.7 (0.6-0.9) | 0.09 (−0.05 to 0.24) |
| Southern Sub-Saharan Africa | 16.7 (13.8-19.9) | 9.3 (7.6-11.1) | −0.44 (−0.5 to −0.39) | 4.3 (3.2-5.7) | 2 (1.6-2.6) | −0.53 (−0.64 to −0.38) |
| Western Sub-Saharan Africa | 5.1 (4.2-6.1) | 4.6 (3.8-5.4) | −0.09 (−0.14 to −0.03) | 0.5 (0.4-0.6) | 0.4 (0.3-0.5) | −0.12 (−0.25 to 0.04) |

**Supplementary Table S13. Global and regional age-specific summary exposure value (SEV) of chewing tobacco among individuals aged 15-49 years, for both sexes, in 1990 and 2019, with the annualized rate of change (ARC) from 1990 to 2019**

| Characteristics | Male | | | Female | | |
|---|---|---|---|---|---|---|
| | Age-specific SEV in 1990 (95% UI) | Age-specific SEV in 2019 (95% UI) | ARC (%) (95% UI) | Age-specific SEV in 1990 (95% UI) | Age-specific SEV in 2019 (95% UI) | ARC (%) (95% UI) |
| **Overall** | 6.9 (5.8-7.9) | 7.5 (6.7-8.4) | 0.09 (−0.09 to 0.32) | 2.7 (2.1-3.4) | 3 (2.6-3.6) | 0.11 (−0.17 to 0.51) |
| **Socio-demographic index** | | | | | | |
| High | 1.5 (1.2-1.8) | 1.6 (1.3-2) | 0.06 (−0.21 to 0.43) | 0.3 (0.2-0.3) | 0.3 (0.2-0.4) | 0.17 (−0.12 to 0.54) |
| High -middle | 1.9 (1.5-2.3) | 2.4 (2-2.8) | 0.24 (−0.04 to 0.62) | 0.6 (0.4-0.7) | 0.7 (0.5-0.9) | 0.18 (−0.22 to 0.82) |
| Middle | 4 (3.3-4.9) | 4.9 (4.2-5.6) | 0.2 (−0.04 to 0.53) | 1.3 (0.9-1.6) | 1.7 (1.3-2.1) | 0.33 (−0.07 to 0.97) |
| Low-middle | 19 (16-22.5) | 17 (15-19.1) | −0.11 (−0.27 to 0.11) | 9.8 (7.6-12.2) | 8.7 (7.2-10.4) | −0.11 (−0.34 to 0.24) |
| Low | 17.7 (15.2-20.2) | 14.7 (13.1-16.4) | −0.17 (−0.3 to 0) | 4.6 (3.6-5.9) | 3.8 (3.2-4.6) | −0.17 (−0.39 to 0.16) |
| **GBD regions** | | | | | | |
| High-income Asia Pacific | 0.6 (0.5-0.9) | 0.7 (0.5-0.9) | 0.07 (−0.26 to 0.55) | 0.3 (0.2-0.5) | 0.5 (0.3-0.7) | 0.48 (−0.04 to 1.31) |
| Central Asia | 3.4 (2.7-4.4) | 4.7 (3.7-5.9) | 0.36 (−0.03 to 0.89) | 0.2 (0.2-0.3) | 0.2 (0.2-0.3) | 0.07 (−0.19 to 0.41) |
| East Asia | 0.9 (0.6-1.3) | 1 (0.7-1.5) | 0.16 (−0.27 to 0.87) | 0.2 (0.1-0.3) | 0.2 (0.1-0.3) | −0.01 (−0.48 to 0.77) |
| South Asia | 30.2 (25.1-35.9) | 27.8 (24.4-31.7) | −0.08 (−0.25 to 0.14) | 13 (9.8-16.6) | 11.8 (9.6-14.4) | −0.09 (−0.35 to 0.3) |
| Southeast Asia | 2.5 (2-2.9) | 2.4 (2.1-2.9) | −0.01 (−0.21 to 0.24) | 1.4 (1.2-1.7) | 1.4 (1.2-1.7) | 0.01 (−0.21 to 0.29) |
| Australasia | 0.6 (0.4-0.8) | 0.5 (0.4-0.7) | −0.08 (−0.4 to 0.41) | 0.2 (0.2-0.3) | 0.3 (0.2-0.4) | 0.13 (−0.3 to 0.83) |
| Caribbean | 0.6 (0.5-0.7) | 0.7 (0.6-0.9) | 0.24 (−0.04 to 0.63) | 0.3 (0.2-0.3) | 0.3 (0.2-0.4) | 0.13 (−0.16 to 0.55) |
| Central Europe | 0.4 (0.3-0.4) | 0.5 (0.4-0.5) | 0.25 (0.03 to 0.52) | 0.2 (0.2-0.3) | 0.2 (0.2-0.3) | 0.14 (−0.1 to 0.48) |
| Eastern Europe | 0.3 (0.2-0.3) | 0.3 (0.2-0.3) | 0.02 (−0.29 to 0.46) | 0.2 (0.2-0.3) | 0.2 (0.2-0.3) | 0 (−0.36 to 0.6) |

| | | | | | | |
|---|---|---|---|---|---|---|
| Western Europe | 0.2 (0.2-0.2) | 0.2 (0.2-0.3) | 0.11 (−0.06 to 0.31) | 0.2 (0.1-0.2) | 0.2 (0.1-0.2) | 0.07 (−0.13 to 0.3) |
| Andean Latin America | 0.6 (0.5-0.8) | 0.5 (0.4-0.7) | −0.2 (−0.46 to 0.19) | 0.5 (0.4-0.8) | 0.4 (0.3-0.6) | −0.21 (−0.53 to 0.31) |
| Central Latin America | 0.7 (0.5-0.8) | 0.6 (0.5-0.8) | −0.05 (−0.27 to 0.25) | 0.3 (0.2-0.4) | 0.3 (0.2-0.4) | −0.02 (−0.31 to 0.38) |
| Southern Latin America | 0.2 (0.1-0.2) | 0.2 (0.1-0.2) | −0.12 (−0.37 to 0.24) | 0.2 (0.1-0.3) | 0.2 (0.1-0.3) | −0.02 (−0.37 to 0.56) |
| Tropical Latin America | 0.3 (0.2-0.4) | 0.3 (0.2-0.4) | 0.05 (−0.3 to 0.6) | 0.2 (0.2-0.4) | 0.2 (0.1-0.4) | 0 (−0.45 to 0.83) |
| North Africa and Middle East | 2.7 (2.4-3) | 2.9 (2.6-3.3) | 0.09 (−0.06 to 0.29) | 0.5 (0.4-0.6) | 0.4 (0.3-0.5) | −0.08 (−0.3 to 0.23) |
| High-income North America | 3.6 (2.8-4.5) | 3.8 (2.7-5.1) | 0.05 (−0.29 to 0.54) | 0.3 (0.2-0.5) | 0.3 (0.2-0.5) | 0.11 (−0.38 to 0.97) |
| Oceania | 6 (4.6-8) | 6 (4.4-8.1) | −0.01 (−0.32 to 0.43) | 2.4 (1.7-3.5) | 2.5 (1.7-3.8) | 0.03 (−0.4 to 0.74) |
| Central Sub-Saharan Africa | 0.6 (0.5-0.8) | 0.6 (0.5-0.8) | 0.02 (−0.26 to 0.4) | 1.6 (1.1-2.4) | 1.3 (0.9-2) | −0.18 (−0.52 to 0.41) |
| Eastern Sub-Saharan Africa | 2.6 (2.2-3.1) | 2.8 (2.4-3.2) | 0.07 (−0.14 to 0.34) | 1.5 (1.3-1.8) | 1.6 (1.3-1.8) | 0.02 (−0.21 to 0.3) |
| Southern Sub-Saharan Africa | 0.8 (0.6-1.1) | 0.6 (0.5-0.9) | −0.2 (−0.47 to 0.27) | 1.4 (0.9-2.1) | 1.3 (0.9-1.9) | −0.07 (−0.46 to 0.59) |
| Western Sub-Saharan Africa | 0.8 (0.7-0.9) | 0.8 (0.8-1) | 0.03 (−0.13 to 0.24) | 0.7 (0.6-0.9) | 0.7 (0.6-0.9) | 0.01 (−0.21 to 0.29) |

**Supplementary Table S14. Summary exposure value (SEV) of tobacco and alcohol by SDI quintiles and age in 2019, and its annualized rate of change (ARC) from 1990 to 2019**

| Characteristics | Alcohol use | | Smoking | | Chewing tobacco | |
|---|---|---|---|---|---|---|
| | Age-specific SEV in 2019 (95% UI) | ARC (%) (95% UI) | Age-specific SEV in 2019 (95% UI) | ARC (%) (95% UI) | Age-specific SEV in 2019 (95% UI) | ARC (%) (95% UI) |
| **Global** | | | | | | |
| 15-19 years | 3.5 (2.3-5.1) | −0.03 (−0.1 to 0.04) | NA | NA | NA | NA |
| 20-24 years | 6.8 (4.8-9.3) | 0 (−0.07 to 0.07) | NA | NA | NA | NA |
| 25-29 years | 8 (5.7-10.9) | −0.02 (−0.08 to 0.06) | NA | NA | NA | NA |
| 30-34 years | 7.9 (5.6-10.7) | −0.05 (−0.11 to 0.03) | 5.4 (4.6-6.1) | −0.33 (−0.36 to −0.31) | 5.1 (4.1-6.1) | 0.11 (−0.18 to 0.51) |
| 35-39 years | 8.1 (5.8-11) | −0.04 (−0.1 to 0.04) | 7.4 (6.2-8.7) | −0.33 (−0.35 to −0.31) | 5.4 (4.5-6.4) | 0.17 (−0.15 to 0.64) |
| 40-44 years | 8.4 (6.1-11.5) | −0.07 (−0.12 to 0) | 9.4 (7.9-11.2) | −0.32 (−0.34 to −0.3) | 5.5 (4.5-6.5) | 0.08 (−0.21 to 0.55) |
| 45-49 years | 8.7 (6.2-11.8) | −0.05 (−0.11 to 0.02) | 12.1 (10.2-14.1) | −0.27 (−0.29 to −0.24) | 5.2 (4.4-6.2) | −0.01 (−0.27 to 0.35) |
| **High SDI** | | | | | | |
| 15-19 years | 10 (6.6-14.2) | −0.11 (−0.17 to −0.03) | NA | NA | NA | NA |
| 20-24 years | 15.4 (10.9-20.8) | −0.1 (−0.15 to −0.04) | NA | NA | NA | NA |
| 25-29 years | 15.8 (11.3-21.4) | −0.09 (−0.15 to −0.04) | NA | NA | NA | NA |
| 30-34 years | 14.3 (10.2-19.4) | −0.12 (−0.17 to −0.06) | 8.4 (7.2-9.6) | −0.37 (−0.4 to −0.34) | 1 (0.7-1.5) | 0.05 (−0.37 to 0.77) |
| 35-39 years | 14.2 (10.2-19.4) | −0.1 (−0.16 to −0.05) | 11.1 (9.2-13.2) | −0.37 (−0.39 to −0.33) | 0.9 (0.6-1.4) | 0.06 (−0.37 to 0.87) |
| 40-44 years | 14.6 (10.5-19.9) | −0.11 (−0.15 to −0.05) | 13.3 (10.9-15.8) | −0.36 (−0.39 to −0.33) | 1 (0.7-1.4) | 0.11 (−0.32 to 0.76) |
| 45-49 years | 14.9 (10.7-20.3) | −0.09 (−0.14 to −0.04) | 15.6 (13.2-18.2) | −0.36 (−0.4 to −0.32) | 1 (0.7-1.4) | 0.15 (−0.27 to 0.86) |
| **High-middle SDI** | | | | | | |
| 15-19 years | 5.9 (3.9-8.6) | 0.1 (0 to 0.23) | NA | NA | NA | NA |
| 20-24 years | 9.9 (6.9-13.5) | 0.08 (−0.02 to 0.2) | NA | NA | NA | NA |
| 25-29 years | 10.6 (7.6-14.5) | 0.02 (−0.07 to 0.13) | NA | NA | NA | NA |
| 30-34 years | 10.2 (7.3-14.1) | −0.02 (−0.11 to 0.08) | 7.8 (6.7-8.9) | −0.24 (−0.27 to −0.2) | 1.4 (1-1.9) | 0.18 (−0.24 to 0.87) |
| 35-39 years | 10.5 (7.6-14.3) | −0.01 (−0.1 to 0.08) | 10.7 (9-12.6) | −0.21 (−0.24 to −0.18) | 1.6 (1.2-2.1) | 0.35 (−0.13 to 1.21) |
| 40-44 years | 10.9 (7.8-14.6) | −0.04 (−0.12 to 0.05) | 13.2 (11-15.7) | −0.21 (−0.24 to −0.18) | 1.7 (1.2-2.2) | 0.25 (−0.18 to 0.98) |

| | | | | | | |
|---|---|---|---|---|---|---|
| 45-49 years | 10.9 (7.9-14.7) | −0.08 (−0.15 to 0.01) | 16.1 (13.7-18.8) | −0.18 (−0.21 to −0.15) | 1.5 (1.1-2) | 0.1 (−0.28 to 0.69) |
| **Middle SDI** | | | | | | |
| 15-19 years | 3.3 (2.1-4.7) | 0.46 (0.28 to 0.67) | NA | NA | NA | NA |
| 20-24 years | 6.5 (4.6-8.8) | 0.32 (0.17 to 0.52) | NA | NA | NA | NA |
| 25-29 years | 7.4 (5.3-9.9) | 0.26 (0.12 to 0.43) | NA | NA | NA | NA |
| 30-34 years | 7 (5-9.6) | 0.18 (0.05 to 0.35) | 5.3 (4.5-6.1) | −0.23 (−0.27 to −0.18) | 3.2 (2.4-4) | 0.23 (−0.18 to 0.85) |
| 35-39 years | 7 (5.1-9.5) | 0.15 (0.01 to 0.33) | 7.1 (6-8.4) | −0.26 (−0.3 to −0.22) | 3.4 (2.7-4.3) | 0.35 (−0.09 to 1) |
| 40-44 years | 7.3 (5.2-9.8) | 0.13 (−0.01 to 0.28) | 9 (7.5-10.6) | −0.25 (−0.28 to −0.21) | 3.4 (2.6-4.3) | 0.17 (−0.22 to 0.85) |
| 45-49 years | 7.4 (5.3-10) | 0.14 (0 to 0.3) | 11.6 (9.8-13.6) | −0.18 (−0.22 to −0.14) | 3.2 (2.5-3.9) | 0.09 (−0.27 to 0.61) |
| **Low-middle SDI** | | | | | | |
| 15-19 years | 1.8 (1.1-2.7) | 0.27 (0.09 to 0.51) | NA | NA | NA | NA |
| 20-24 years | 4.1 (2.8-5.8) | 0.31 (0.12 to 0.56) | NA | NA | NA | NA |
| 25-29 years | 5.1 (3.6-7) | 0.34 (0.15 to 0.57) | NA | NA | NA | NA |
| 30-34 years | 5.1 (3.6-7) | 0.34 (0.14 to 0.58) | 3.4 (2.9-3.9) | −0.3 (−0.34 to −0.25) | 11.7 (9.5-14.4) | −0.11 (−0.37 to 0.25) |
| 35-39 years | 5.3 (3.7-7.2) | 0.34 (0.14 to 0.6) | 4.8 (4-5.7) | −0.31 (−0.35 to −0.26) | 13 (10.6-15.6) | −0.09 (−0.36 to 0.33) |
| 40-44 years | 5.4 (3.8-7.4) | 0.32 (0.12 to 0.59) | 6.4 (5.4-7.7) | −0.28 (−0.32 to −0.23) | 13.3 (10.7-16.2) | −0.15 (−0.39 to 0.29) |
| 45-49 years | 5.3 (3.7-7.3) | 0.33 (0.12 to 0.6) | 8.6 (7.2-10.2) | −0.24 (−0.28 to −0.19) | 13.7 (11.2-16.5) | −0.13 (−0.38 to 0.24) |
| **Low SDI** | | | | | | |
| 15-19 years | 1.7 (1-2.8) | 0.13 (−0.03 to 0.32) | NA | NA | NA | NA |
| 20-24 years | 3.9 (2.6-5.7) | 0.17 (0.03 to 0.34) | NA | NA | NA | NA |
| 25-29 years | 4.8 (3.3-6.9) | 0.17 (0.04 to 0.34) | NA | NA | NA | NA |
| 30-34 years | 4.9 (3.3-7.1) | 0.19 (0.05 to 0.36) | 2.2 (1.8-2.5) | −0.21 (−0.26 to −0.15) | 8.1 (6.5-9.7) | −0.17 (−0.41 to 0.14) |
| 35-39 years | 5 (3.4-7.2) | 0.21 (0.07 to 0.39) | 3.1 (2.6-3.6) | −0.21 (−0.26 to −0.16) | 8.8 (7.2-10.8) | −0.19 (−0.41 to 0.12) |
| 40-44 years | 5.2 (3.6-7.4) | 0.22 (0.06 to 0.41) | 4 (3.3-4.8) | −0.21 (−0.26 to −0.16) | 9.8 (8.1-11.6) | −0.18 (−0.4 to 0.16) |
| 45-49 years | 5.2 (3.5-7.4) | 0.2 (0.06 to 0.38) | 5.1 (4.2-6.1) | −0.2 (−0.25 to −0.15) | 11 (9.2-13) | −0.14 (−0.36 to 0.17) |

The smoking and chewing tobacco risk factors were modeled with lower age restrictions of 30 years in the GBD 2019 study; thus, estimates were not produced for these risk factors in the age groups of 15-19 years, 20- 24 years, and 25-29 years.

**Supplementary Table S15. Global and regional DALYs counts and age-specific DALYs rates for early-onset Lip and oral cavity cancer attributable to smoking, both sexes combined, in 1990 and 2019, and change in absolute DALYs and age-specific DALYs from 1990 to 2019**

| Characteristics | 1990 DALYs | | 2019 DALYs | | 1990-2019 DALYs | |
|---|---|---|---|---|---|---|
| | Case number No. (95% UI) | Rate per 100,000 No. (95% UI) | Case number No. (95% UI) | Rate per 100,000 No. (95% UI) | Percent change in case number (%) | EAPC No. (95% CI) |
| **Overall** | 199311 (139237-259200) | 7.35 (5.13-9.56) | 261308 (168833-353083) | 6.64 (4.29-8.97) | 73.9 | −0.51 (−0.68 to −0.34) |
| **Sex** | | | | | | |
| Male | 184255 (129392-239489) | 13.41 (9.42-17.43) | 244211 (159215-326386) | 12.27 (8.00-16.40) | 32.5 | −0.44 (−0.62 to −0.27) |
| Female | 15056 (9574-20757) | 1.13 (0.72-1.55) | 17098 (10041-24618) | 0.88 (0.52-1.27) | 13.6 | −1.09 (−1.32 to −0.86) |
| **Socio-demographic index** | | | | | | |
| High | 46012 (34034-56488) | 10.7 (7.91-13.13) | 30436 (20667-39547) | 6.48 (4.4-8.42) | −25.6 | −1.99 (−2.38 to −1.61) |
| High -middle | 53793 (38787-66433) | 8.89 (6.41-10.98) | 61145 (42259-78770) | 8.39 (5.8-10.81) | 18.2 | −0.82 (−1.09 to −0.55) |
| Middle | 39333 (26558-52439) | 4.36 (2.94-5.81) | 68073 (43357-94030) | 5.4 (3.44-7.46) | 116.6 | 0.93 (0.74 to 1.13) |
| Low-middle | 46927 (29952-66324) | 8.68 (5.54-12.27) | 76339 (47100-109784) | 8.18 (5.05-11.76) | 156.9 | −0.17 (−0.26 to −0.07) |
| Low | 13167 (8164-19192) | 5.66 (3.51-8.25) | 25220 (14060-38095) | 4.67 (2.6-7.05) | 150.5 | −0.59 (−0.78 to −0.4) |
| **GBD regions** | | | | | | |
| High-income Asia Pacific | 4267 (3104-5293) | 4.59 (3.34-5.7) | 3392 (2288-4381) | 4.18 (2.82-5.4) | 19.1 | −0.6 (−1.01 to −0.19) |
| Central Asia | 1567 (1099-2013) | 4.7 (3.29-6.03) | 2227 (1499-2969) | 4.56 (3.07-6.08) | 61.3 | −0.71 (−1 to −0.41) |
| East Asia | 19,055 (12,757-25,940) | 2.76 (1.85-3.76) | 44,044 (29,234-59,501) | 5.9 (3.92-7.97) | 24 | 3.16 (2.78 to 3.53) |
| South Asia | 65,570 (41,958-92,563) | 12.39 (7.93-17.49) | 109,324 (63,850-159,335) | 11.22 (6.55-16.36) | 161.5 | −0.29 (−0.42 to −0.17) |
| Southeast Asia | 12,972 (8,797-17,190) | 5.49 (3.72-7.27) | 22,782 (13,905-32,389) | 6.29 (3.84-8.95) | 175.3 | 0.32 (0.21 to 0.44) |
| Australasia | 1,546 (1,070-1,986) | 14.33 (9.91-18.4) | 851 (539-1,179) | 6.29 (3.98-8.72) | 10.8 | −2.67 (−3.05 to −2.3) |
| Caribbean | 724 (465-993) | 3.97 (2.55-5.44) | 850 (503-1,264) | 3.55 (2.1-5.29) | 26.8 | −0.1 (−0.29 to 0.1) |
| Central Europe | 14,458 (10,719-17,705) | 23.7 (17.57-29.02) | 9,759 (6,654-12,747) | 18.51 (12.62-24.18) | −3.7 | −1.91 (−2.33 to −1.48) |
| Eastern Europe | 18,050 (13,213-22,398) | 16.36 (11.98-20.3) | 24,728 (17,415-31,807) | 25.22 (17.76-32.44) | 41.6 | 0.7 (0.25 to 1.15) |

| | | | | | | |
|---|---|---|---|---|---|---|
| Western Europe | 30,183 (21,885-37,364) | 15.6 (11.31-19.32) | 13,203 (9,037-17,249) | 6.92 (4.74-9.04) | −46.5 | −3.28 (−3.65 to −2.92) |
| Andean Latin America | 137 (77-207) | 0.74 (0.41-1.11) | 180 (93-291) | 0.54 (0.28-0.88) | 26.2 | −0.96 (−1.1 to −0.82) |
| Central Latin America | 1,256 (790-1,712) | 1.54 (0.97-2.1) | 1,195 (663-1,819) | 0.91 (0.5-1.38) | 35.3 | −2.02 (−2.12 to −1.93) |
| Southern Latin America | 1,480 (1,027-1,932) | 6.04 (4.19-7.89) | 1,301 (820-1,777) | 3.82 (2.41-5.22) | −41.6 | −1.79 (−1.97 to −1.61) |
| Tropical Latin America | 7,366 (5,078-9,385) | 9.38 (6.47-11.95) | 6,305 (3,850-8,744) | 5.29 (3.23-7.34) | 49.6 | −2.2 (−2.75 to −1.65) |
| North Africa and Middle East | 3,395 (2,281-4,716) | 2.09 (1.4-2.9) | 6,415 (4,280-8,866) | 1.92 (1.28-2.66) | 115.5 | −0.74 (−0.89 to −0.6) |
| High-income North America | 11,970 (9,251-14,351) | 8.05 (6.22-9.65) | 6,216 (4,368-8,105) | 3.73 (2.62-4.86) | −15.3 | −2.94 (−3.23 to −2.66) |
| Oceania | 149 (85-240) | 4.71 (2.68-7.6) | 319 (169-540) | 4.68 (2.48-7.94) | 117 | 0.34 (0.19 to 0.49) |
| Central Sub-Saharan Africa | 459 (242-732) | 1.88 (0.99-3) | 949 (505-1,554) | 1.53 (0.81-2.5) | 159.9 | −0.92 (−1.04 to −0.79) |
| Eastern Sub-Saharan Africa | 1,764 (984-2,736) | 2.13 (1.19-3.3) | 3,720 (2,070-5,805) | 1.87 (1.04-2.92) | 174.9 | −0.49 (−0.63 to −0.35) |
| Southern Sub-Saharan Africa | 2,251 (1,451-2,943) | 8.63 (5.56-11.28) | 1,813 (1,074-2,561) | 4.28 (2.54-6.05) | 43 | −3.43 (−3.99 to −2.87) |
| Western Sub-Saharan Africa | 693 (398-1,046) | 0.81 (0.47-1.23) | 1,738 (1,012-2,692) | 0.81 (0.47-1.25) | 165.7 | −0.02 (−0.11 to 0.07) |

**Supplementary Table S16. Global and regional DALYs counts and age-specific DALYs rates for early-onset Lip and oral cavity cancer attributable to chewing tobacco, both sexes combined, in 1990 and 2019, and change in absolute DALYs and age-specific DALYs from 1990 to 2019**

| Characteristics | 1990 DALYs | | 2019 DALYs | | 1990-2019 DALYs | |
|---|---|---|---|---|---|---|
| | Case number No. (95% UI) | Rate per 100,000 No. (95% UI) | Case number No. (95% UI) | Rate per 100,000 No. (95% UI) | Percent change in case number (%) | EAPC No. (95% CI) |
| **Overall** | 135,931 (100,030-175,842) | 5.01 (3.69-6.48) | 286,874 (199,350-382,248) | 7.29 (5.07-9.71) | 111 | 1.26 (1.17 to 1.34) |
| **Sex** | | | | | | |
| Male | 87,344 (51,482-125,765) | 6.36 (3.75-9.15) | 182,161 (107,285-274,885) | 9.15 (5.39-13.82) | 108.6 | 1.31 (1.15 to 1.47) |
| Female | 48,587 (34,056-63,754) | 4.77 (2.55-4.77) | 104,712 (73,344-140,958) | 5.38 (3.77-7.25) | 115.5 | 1.10 (0.93 to 1.27) |
| **Socio-demographic index** | | | | | | |
| High | 2,046 (1,250-3,230) | 0.48 (0.29-0.75) | 2,039 (1,225-3,101) | 0.43 (0.26-0.66) | −0.3 | −0.48 (−0.69 to −0.26) |
| High -middle | 7,967 (5,135-11,196) | 1.32 (0.85-1.85) | 16,951 (10,435-25,138) | 2.33 (1.43-3.45) | 112.8 | 2 (1.81 to 2.18) |
| Middle | 24,318 (16,482-33,524) | 2.69 (1.83-3.71) | 57,416 (36,564-82,586) | 4.55 (2.9-6.55) | 136.1 | 2.03 (1.87 to 2.2) |
| Low-middle | 77,155 (57,932-98,633) | 14.28 (10.72-18.25) | 157,678 (113,042-208,421) | 16.89 (12.11-22.33) | 104.4 | 0.48 (0.38 to 0.58) |
| Low | 24,435 (17,487-31,808) | 10.51 (7.52-13.68) | 52,771 (36,544-71,452) | 9.77 (6.76-13.22) | 116 | −0.36 (−0.52 to −0.2) |
| **GBD regions** | | | | | | |
| High-income Asia Pacific | 171 (106-262) | 0.18 (0.11-0.28) | 233 (148-352) | 0.29 (0.18-0.43) | 36.3 | 1.6 (1.43 to 1.76) |
| Central Asia | 200 (115-311) | 0.6 (0.34-0.93) | 503 (276-793) | 1.03 (0.56-1.62) | 151.5 | 1.33 (0.96 to 1.71) |
| East Asia | 1,223 (706-2,030) | 0.18 (0.1-0.29) | 2,409 (1,270-4,144) | 0.32 (0.17-0.56) | 97 | 2.33 (2.15 to 2.5) |
| South Asia | 126,319 (93,302-162,509) | 23.86 (17.63-30.7) | 270,646 (188,344-361,151) | 27.78 (19.33-37.07) | 114.3 | 0.48 (0.38 to 0.58) |
| Southeast Asia | 3,002 (2,022-4,158) | 1.27 (0.86-1.76) | 4,934 (3,161-7,181) | 1.36 (0.87-1.98) | 64.4 | 0.19 (0.04 to 0.33) |
| Australasia | 51 (29-81) | 0.47 (0.27-0.75) | 40 (23-64) | 0.3 (0.17-0.47) | −21.6 | −1.54 (−1.77 to −1.3) |
| Caribbean | 40 (26-61) | 0.22 (0.14-0.33) | 70 (41-112) | 0.29 (0.17-0.47) | 75 | 1.06 (0.93 to 1.18) |
| Central Europe | 229 (137-355) | 0.37 (0.22-0.58) | 238 (137-376) | 0.45 (0.26-0.71) | 3.9 | −0.06 (−0.31 to 0.2) |
| Eastern Europe | 247 (146-382) | 0.22 (0.13-0.35) | 372 (220-580) | 0.38 (0.22-0.59) | 50.6 | 1.13 (0.73 to 1.52) |
| Western Europe | 302 (187-457) | 0.16 (0.1-0.24) | 214 (139-309) | 0.11 (0.07-0.16) | −29.1 | −1.5 (−1.71 to −1.28) |
| Andean Latin America | 32 (20-49) | 0.17 (0.11-0.26) | 44 (25-75) | 0.13 (0.08-0.23) | 37.5 | −0.81 (−0.97 to −0.65) |

| | | | | | | |
|---|---|---|---|---|---|---|
| Central Latin America | 100 (66-144) | 0.12 (0.08-0.18) | 180 (114-273) | 0.14 (0.09-0.21) | 80 | 0.26 (0.19 to 0.34) |
| Southern Latin America | 21 (12-31) | 0.09 (0.05-0.13) | 26 (15-40) | 0.08 (0.04-0.12) | 23.8 | −0.57 (−0.69 to −0.44) |
| Tropical Latin America | 143 (82-231) | 0.18 (0.1-0.29) | 236 (138-367) | 0.2 (0.12-0.31) | 65 | 0.04 (−0.27 to 0.35) |
| North Africa and Middle East | 569 (326-895) | 0.35 (0.2-0.55) | 1,338 (802-1,987) | 0.4 (0.24-0.6) | 135.1 | 0.42 (0.32 to 0.52) |
| High-income North America | 1,441 (848-2,322) | 0.97 (0.57-1.56) | 1,130 (620-1,875) | 0.68 (0.37-1.12) | −21.6 | −1.42 (−1.59 to −1.25) |
| Oceania | 63 (38-93) | 1.98 (1.21-2.95) | 157 (90-239) | 2.3 (1.33-3.52) | 149.2 | 0.57 (0.5 to 0.64) |
| Central Sub-Saharan Africa | 163 (94-262) | 0.67 (0.39-1.07) | 372 (207-602) | 0.6 (0.33-0.97) | 128.2 | −0.57 (−0.82 to −0.33) |
| Eastern Sub-Saharan Africa | 1,122 (761-1,572) | 1.35 (0.92-1.89) | 2,724 (1,837-3,743) | 1.37 (0.92-1.88) | 142.8 | 0.06 (0.02 to 0.1) |
| Southern Sub-Saharan Africa | 226 (144-341) | 0.87 (0.55-1.31) | 286 (183-429) | 0.68 (0.43-1.01) | 26.5 | −1.45 (−1.87 to −1.02) |
| Western Sub-Saharan Africa | 268 (180-385) | 0.31 (0.21-0.45) | 720 (469-1,051) | 0.33 (0.22-0.49) | 168.7 | 0.19 (0.13 to 0.26) |

**Supplementary Table S16. Global and regional DALYs counts and age-specific DALYs rates for early-onset Lip and oral cavity cancer attributable to alcohol use, both sexes combined, in 1990 and 2019, and change in absolute DALYs and age-specific DALYs from 1990 to 2019**

| Characteristics | 1990 DALYs | | 2019 DALYs | | 1990-2019 DALYs | |
|---|---|---|---|---|---|---|
| | Case number No. (95% UI) | Rate per 100,000 No. (95% UI) | Case number No. (95% UI) | Rate per 100,000 No. (95% UI) | Percent change in case number (%) | EAPC No. (95% CI) |
| **Overall** | 256,921 (213,879-302,756) | 9.47 (7.89-11.16) | 459,290 (364,232-562,390) | 11.67 (9.26-14.29) | 72.7 | 0.6 (0.51 to 0.68) |
| **Sex** | | | | | | |
| Male | 231,258 (193,869-271,888) | 16.83 (14.11-19.78) | 418,093 (332,650-513,389) | 21.01 (16.72-25.80) | 80.8 | 0.65 (0.54 to 0.75) |
| Female | 25,664 (19,876-30,961) | 1.92 (1.49-2.31) | 41,197 (30,640-51,816) | 2.12 (1.58-2.66) | 60.5 | 0.05 (−0.06 to 0.15) |
| **Socio-demographic index** | | | | | | |
| High | 68,359 (59,102-76,041) | 15.89 (13.74-17.68) | 50,582 (42,652-58,747) | 10.76 (9.08-12.5) | −26.5 | −1.7 (−2.01 to −1.4) |
| High -middle | 76,229 (64,999-86,682) | 12.59 (10.74-14.32) | 101,283 (83,354-119,771) | 13.9 (11.44-16.44) | 18.3 | −0.18 (−0.4 to 0.03) |
| Middle | 54,402 (43,536-66,411) | 6.03 (4.82-7.36) | 133,320 (101,856-167,474) | 10.58 (8.08-13.28) | 117.1 | 2.17 (2.02 to 2.33) |
| Low-middle | 42,860 (30,865-57,401) | 7.93 (5.71-10.62) | 130,704 (94,170-172,397) | 14 (10.09-18.47) | 154.2 | 2.24 (2.08 to 2.39) |
| Low | 14,948 (10,387-20,609) | 6.43 (4.47-8.86) | 43,225 (30,375-57,261) | 8 (5.62-10.6) | 147.4 | 0.85 (0.77 to 0.92) |
| **GBD regions** | | | | | | |
| High-income Asia Pacific | 6,445 (5,455-7,324) | 6.94 (5.87-7.89) | 6,396 (5,271-7,430) | 7.88 (6.5-9.16) | 16.7 | 0.08 (−0.29 to 0.44) |
| Central Asia | 2,511 (2,002-3,081) | 7.53 (6-9.23) | 4,308 (3,400-5,371) | 8.82 (6.96-11) | 61.8 | −0.12 (−0.42 to 0.19) |
| East Asia | 28,318 (21,784-35,478) | 4.1 (3.16-5.14) | 62,161 (47,806-79,423) | 8.33 (6.41-10.64) | 26 | 2.84 (2.61 to 3.07) |
| South Asia | 65,873 (47,366-89,538) | 12.44 (8.95-16.92) | 206,242 (143,752-280,172) | 21.17 (14.76-28.76) | 158.3 | 2.17 (1.97 to 2.36) |
| Southeast Asia | 12,034 (9,234-15,149) | 5.09 (3.91-6.41) | 33,568 (24,800-43,416) | 9.27 (6.85-11.99) | 177.6 | 1.97 (1.85 to 2.09) |
| Australasia | 2,691 (2,302-3,053) | 24.94 (21.33-28.29) | 1,959 (1,580-2,375) | 14.49 (11.68-17.56) | 10.5 | −1.84 (−2.17 to −1.52) |
| Caribbean | 1,635 (1,344-1,963) | 8.96 (7.37-10.76) | 2,335 (1,749-3,022) | 9.77 (7.31-12.64) | 26.9 | 0.37 (0.28 to 0.47) |
| Central Europe | 19,783 (17,169-22,179) | 32.43 (28.14-36.36) | 14,730 (11,935-17,696) | 27.94 (22.64-33.57) | −2.6 | −1.48 (−1.81 to −1.14) |
| Eastern Europe | 25,138 (21,461-28,912) | 22.79 (19.45-26.21) | 35,413 (28,774-42,493) | 36.11 (29.34-43.33) | 40.4 | 0.86 (0.42−1.31) |
| Western Europe | 46,029 (40,379-50,962) | 23.8 (20.88-26.35) | 22,628 (19,318-25,670) | 11.87 (10.13-13.46) | −47 | −2.98 (−3.3 to −2.67) |
| Andean Latin America | 704 (529-906) | 3.78 (2.84-4.86) | 1,273 (877-1,772) | 3.84 (2.65-5.35) | 28.6 | 0.29 (0.11 to 0.47) |

| | | | | | | |
|---|---|---|---|---|---|---|
| Central Latin America | 3,121 (2,568-3,629) | 3.83 (3.15-4.45) | 5,049 (3,946-6,232) | 3.83 (3-4.73) | 39.3 | −0.24 (−0.39 to −0.09) |
| Southern Latin America | 2,495 (2,106-2,871) | 10.19 (8.6-11.72) | 2,559 (2,035-3,062) | 7.52 (5.98-9) | −42.5 | −1.33 (−1.5 to −1.16) |
| Tropical Latin America | 9,318 (7,605-10,940) | 11.86 (9.68-13.93) | 15,770 (12,880-18,714) | 13.23 (10.81-15.7) | 50.2 | 0.06 (−0.4 to 0.52) |
| North Africa and Middle East | 1,707 (1,195-2,373) | 1.05 (0.74-1.46) | 3,022 (2,021-4,195) | 0.91 (0.61-1.26) | 114 | −0.92 (−1.04 to −0.79) |
| High-income North America | 15,838 (13,443-18,068) | 10.66 (9.04-12.16) | 11,734 (9,905-13,472) | 7.04 (5.94-8.08) | −17 | −1.63 (−1.79 to −1.46) |
| Oceania | 152 (94-231) | 4.79 (2.96-7.31) | 328 (187-545) | 4.82 (2.76-8.01) | 100 | 0.21 (−0.05 to 0.47) |
| Central Sub-Saharan Africa | 1,180 (755-1,701) | 4.84 (3.09-6.97) | 3,213 (2,044-4,599) | 5.17 (3.29-7.4) | 175 | 0.4 (0.02 to 0.78) |
| Eastern Sub-Saharan Africa | 6,095 (4,453-8,035) | 7.35 (5.37-9.68) | 15,926 (11,818-21,050) | 8.01 (5.94-10.58) | 175 | 0.21 (0.15 to 0.28) |
| Southern Sub-Saharan Africa | 3,862 (3,144-4,558) | 14.8 (12.05-17.47) | 4,705 (3,659-5,763) | 11.12 (8.65-13.62) | 48 | −1.92 (−2.4 to −1.43) |
| Western Sub-Saharan Africa | 1,992 (1,458-2,580) | 2.34 (1.71-3.03) | 5,971 (4,285-7,850) | 2.78 (1.99-3.65) | 161.5 | 0.46 (0.38 to 0.55) |

**Supplementary Table S17. Global and regional DALYs counts and age-specific DALYs rates for early-onset Other pharyngeal cancer attributable to smoking, both sexes combined, in 1990 and 2019, and change in absolute DALYs and age-specific DALYs from 1990 to 2019**

| Characteristics | 1990 DALYs | | 2019 DALYs | | 1990-2019 DALYs | |
|---|---|---|---|---|---|---|
| | Case number No. (95% UI) | Rate per 100,000 No. (95% UI) | Case number No. (95% UI) | Rate per 100,000 No. (95% UI) | Percent change in case number (%) | EAPC No. (95% CI) |
| **Overall** | 152,198 (117,431-184,399) | 5.61 (4.33-6.8) | 221,188 (162,874-279,004) | 5.62 (4.14-7.09) | 72.7 | −0.35 (−0.5 to −0.19) |
| **Sex** | | | | | | |
| Male | 142,694 (110,326-171,934) | 10.38 (8.03-12.51) | 209,849 (154,812-264,810) | 10.55 (7.78-13.31) | 47.1 | −0.30 (−0.46 to −0.15) |
| Female | 9,504 (6,556-12,729) | 0.71 (0.49-0.95) | 11,339 (7,559-15,443) | 0.58 (0.39-0.79) | 19.3 | −0.95 (−1.18 to −0.73) |
| **Socio-demographic index** | | | | | | |
| High | 33,646 (27,880-38,824) | 7.82 (6.48-9.03) | 23,214 (17,914-28,116) | 4.94 (3.81-5.98) | −26.5 | −2.06 (−2.39 to −1.73) |
| High -middle | 38,473 (30,687-45,376) | 6.36 (5.07-7.5) | 42,038 (32,801-50,704) | 5.77 (4.5-6.96) | 18.3 | −1.27 (−1.64 to −0.9) |
| Middle | 27,656 (20,353-34,188) | 3.06 (2.26-3.79) | 47,768 (34,146-61,529) | 3.79 (2.71-4.88) | 117.1 | 0.61 (0.48 to 0.74) |
| Low-middle | 42,979 (30,485-56,741) | 7.95 (5.64-10.5) | 88,901 (60,886-118,997) | 9.52 (6.52-12.75) | 154.2 | 0.61 (0.52 to 0.71) |
| Low | 9,388 (5,987-13,275) | 4.04 (2.57-5.71) | 19,206 (12,703-26,715) | 3.55 (2.35-4.94) | 147.4 | −0.33 (−0.4 to −0.26) |
| **GBD regions** | | | | | | |
| High-income Asia Pacific | 1,870 (1,554-2,143) | 2.01 (1.67-2.31) | 2,054 (1,523-2,557) | 2.53 (1.88-3.15) | 16.7 | 0.34 (−0.07 to 0.76) |
| Central Asia | 963 (702-1,243) | 2.89 (2.1-3.73) | 1,401 (1,046-1,756) | 2.87 (2.14-3.6) | 61.8 | −0.7 (−1 to −0.41) |
| East Asia | 12,727 (9,213-16,244) | 1.84 (1.33-2.35) | 16,296 (11,945-20,848) | 2.18 (1.6-2.79) | 26 | 0.26 (0.02 to 0.49) |
| South Asia | 61,006 (43,029-80,047) | 11.53 (8.13-15.12) | 125,863 (84,648-169,303) | 12.92 (8.69-17.38) | 158.3 | 0.37 (0.28 to 0.46) |
| Southeast Asia | 5,986 (4,243-7,654) | 2.53 (1.8-3.24) | 13,583 (9,180-18,750) | 3.75 (2.54-5.18) | 177.6 | 1.37 (1.22 to 1.52) |
| Australasia | 567 (435-694) | 5.26 (4.03-6.43) | 540 (376-727) | 3.99 (2.78-5.37) | 10.5 | −0.83 (−1.14 to −0.51) |
| Caribbean | 620 (464-772) | 3.4 (2.54-4.23) | 686 (469-936) | 2.87 (1.96-3.92) | 26.9 | −0.25 (−0.58 to 0.08) |
| Central Europe | 11,084 (9,178-12,680) | 18.17 (15.04-20.79) | 10,160 (7,667-12,668) | 19.27 (14.54-24.03) | −2.6 | −0.87 (−1.44 to −0.31) |
| Eastern Europe | 11,746 (8,969-14,774) | 10.65 (8.13-13.39) | 16,389 (12,749-20,405) | 16.71 (13-20.81) | 40.4 | 0.58 (0.15 to 1.01) |
| Western Europe | 27,147 (22,156-31,503) | 14.03 (11.45-16.29) | 13,429 (10,352-16,323) | 7.04 (5.43-8.56) | −47 | −3.11 (−3.46 to −2.76) |

| | | | | | | |
|---|---|---|---|---|---|---|
| Andean Latin America | 93 (60-131) | 0.5 (0.32-0.71) | 93 (54-139) | 0.28 (0.16-0.42) | 28.6 | −2.2 (−2.48 to −1.92) |
| Central Latin America | 660 (478-825) | 0.81 (0.59-1.01) | 661 (420-934) | 0.5 (0.32-0.71) | 39.3 | −1.79 (−1.89 to −1.69) |
| Southern Latin America | 1,191 (941-1,442) | 4.86 (3.84-5.89) | 628 (470-804) | 1.84 (1.38-2.36) | −42.5 | −3.56 (−4.03 to −3.1) |
| Tropical Latin America | 7,247 (5,740-8,628) | 9.23 (7.31-10.99) | 7,792 (5,577-9,967) | 6.54 (4.68-8.36) | 50.2 | −1.56 (−2.05 to −1.06) |
| North Africa and Middle East | 1,634 (1,186-2,159) | 1.01 (0.73-1.33) | 3,527 (2,583-4,688) | 1.06 (0.77-1.4) | 114 | −0.11 (−0.21 to −0.01) |
| High-income North America | 5,614 (4,787-6,392) | 3.78 (3.22-4.3) | 4,048 (3,221-4,860) | 2.43 (1.93-2.91) | −17 | −1.62 (−1.94 to −1.3) |
| Oceania | 30 (20-43) | 0.95 (0.62-1.36) | 62 (36-96) | 0.91 (0.53-1.41) | 100 | 0.06 (−0.01 to 0.14) |
| Central Sub-Saharan Africa | 169 (100-270) | 0.69 (0.41-1.1) | 385 (227-583) | 0.62 (0.37-0.94) | 175 | −0.5 (−0.71 to −0.3) |
| Eastern Sub-Saharan Africa | 907 (583-1,273) | 1.09 (0.7-1.53) | 2,264 (1,329-3,334) | 1.14 (0.67-1.68) | 175 | 0.19 (0.12 to 0.27) |
| Southern Sub-Saharan Africa | 702 (528-874) | 2.69 (2.02-3.35) | 791 (535-1,064) | 1.87 (1.26-2.52) | 48 | −1.9 (−2.18 to −1.61) |
| Western Sub-Saharan Africa | 235 (151-328) | 0.28 (0.18-0.38) | 536 (335-782) | 0.25 (0.16-0.36) | 161.5 | −0.38 (−0.5 to −0.26) |

**Supplementary Table S18. Global and regional DALYs counts and age-specific DALYs rates for early-onset Other pharyngeal cancer attributable to alcohol use, both sexes combined, in 1990 and 2019, and change in absolute DALYs and age-specific DALYs from 1990 to 2019**

| Characteristics | 1990 DALYs | | 2019 DALYs | | 1990-2019 DALYs | |
|---|---|---|---|---|---|---|
| | Case number No. (95% UI) | Rate per 100,000 No. (95% UI) | Case number No. (95% UI) | Rate per 100,000 No. (95% UI) | Percent change in case number (%) | EAPC No. (95% CI) |
| **Overall** | 136,213 (108,142-164,496) | 5.02 (3.99-6.07) | 260,924 (197,954-330,539) | 6.63 (5.03-8.4) | 91.6 | 0.67 (0.56 to 0.77) |
| **Sex** | | | | | | |
| Male | 126,556 (101,196-152,529) | 9.21 (7.36-11.10) | 244,939 (186,524-311,375) | 12.31 (9.37-15.65) | 93.5 | 0.70 (0.59 to 0.82) |
| Female | 9,656 (6,782-12,621) | 0.72 (0.51-0.94) | 15,986 (10,957-22,192) | 0.82 (0.56-1.14) | 65.6 | 0.18 (0.08 to 0.28) |
| **Socio-demographic index** | | | | | | |
| High | 36,352 (30,849-41,662) | 8.45 (7.17-9.69) | 26,385 (21,375-30,848) | 5.61 (4.55-6.56) | −27.4 | −1.98 (−2.25 to −1.7) |
| High -middle | 39,147 (32,367-45,534) | 6.47 (5.35-7.52) | 47,968 (39,069-56,234) | 6.58 (5.36-7.72) | 22.5 | −0.85 (−1.18 to −0.51) |
| Middle | 24,867 (18,437-31,158) | 2.76 (2.04-3.45) | 65,658 (48,942-84,102) | 5.21 (3.88-6.67) | 164 | 2.27 (2.18 to 2.35) |
| Low-middle | 28,779 (19,250-39,586) | 5.32 (3.56-7.32) | 98,472 (67,651-134,843) | 10.55 (7.25-14.45) | 242.2 | 2.64 (2.47 to 2.81) |
| Low | 7,005 (4,474-10,132) | 3.01 (1.92-4.36) | 22,359 (14,907-31,010) | 4.14 (2.76-5.74) | 219.2 | 1.32 (1.15 to 1.49) |
| **GBD regions** | | | | | | |
| High-income Asia Pacific | 1,900 (1,555-2,221) | 2.05 (1.67-2.39) | 2,316 (1,813-2,783) | 2.85 (2.23-3.43) | 21.9 | 0.65 (0.3 to 0.99) |
| Central Asia | 1,142 (851-1,453) | 3.42 (2.55-4.36) | 1,897 (1,396-2,405) | 3.89 (2.86-4.92) | 66.1 | −0.3 (−0.59 to −0.01) |
| East Asia | 13,043 (9,531-16,651) | 1.89 (1.38-2.41) | 16,511 (12,368-20,874) | 2.21 (1.66-2.8) | 26.6 | 0.18 (−0.11 to 0.47) |
| South Asia | 43,410 (28,850-59,323) | 8.2 (5.45-11.21) | 150,595 (103,304-205,546) | 15.46 (10.6-21.1) | 246.9 | 2.44 (2.29 to 2.6) |
| Southeast Asia | 3,376 (2,419-4,481) | 1.43 (1.02-1.9) | 14,526 (10,038-20,244) | 4.01 (2.77-5.59) | 330.3 | 3.86 (3.65 to 4.07) |
| Australasia | 649 (531-777) | 6.02 (4.92-7.2) | 741 (569-947) | 5.48 (4.21-7) | 14.2 | −0.27 (−0.56 to 0.03) |
| Caribbean | 873 (670-1,069) | 4.78 (3.67-5.86) | 1,169 (838-1,535) | 4.89 (3.51-6.42) | 33.9 | 0.24 (−0.07 to 0.56) |
| Central Europe | 11,402 (9,684-13,028) | 18.69 (15.87-21.36) | 11,155 (8,832-13,488) | 21.16 (16.75-25.59) | −2.2 | −0.61 (−1.1 to −0.11) |
| Eastern Europe | 11,873 (9,431-14,459) | 10.76 (8.55-13.11) | 17,037 (13,389-20,608) | 17.37 (13.65-21.02) | 43.5 | 0.7 (0.27 to 1.13) |
| Western Europe | 30,083 (25,802-34,234) | 15.55 (13.34-17.7) | 15,863 (12,993-18,592) | 8.32 (6.81-9.75) | −47.3 | −2.97 (−3.29 to −2.65) |

| | | | | | | |
|---|---|---|---|---|---|---|
| Andean Latin America | 273 (197-361) | 1.47 (1.06-1.94) | 362 (246-512) | 1.09 (0.74-1.54) | 32.6 | −1.12 (−1.44 to −0.79) |
| Central Latin America | 1,012 (807-1,212) | 1.24 (0.99-1.49) | 1,485 (1,085-1,922) | 1.13 (0.82-1.46) | 46.7 | −0.52 (−0.68 to −0.36) |
| Southern Latin America | 1,352 (1,094-1,584) | 5.52 (4.46-6.47) | 784 (608-959) | 2.3 (1.79-2.82) | −42 | −3.29 (−3.81 to −2.77) |
| Tropical Latin America | 6,531 (5,123-7,891) | 8.32 (6.52-10.05) | 11,796 (9,283-14,000) | 9.9 (7.79-11.74) | 80.6 | 0.16 (−0.28 to 0.6) |
| North Africa and Middle East | 586 (375-843) | 0.36 (0.23-0.52) | 1,236 (793-1,748) | 0.37 (0.24-0.52) | 110.9 | −0.13 (−0.21 to −0.05) |
| High-income North America | 5,252 (4,235-6,157) | 3.53 (2.85-4.14) | 4,801 (3,820-5,648) | 2.88 (2.29-3.39) | −8.6 | −0.74 (−0.96 to −0.51) |
| Oceania | 25 (14-37) | 0.78 (0.45-1.18) | 55 (32-89) | 0.81 (0.48-1.31) | 120 | 0.22 (−0.05 to 0.49) |
| Central Sub-Saharan Africa | 274 (160-416) | 1.12 (0.66-1.7) | 772 (467-1,138) | 1.24 (0.75-1.83) | 181.8 | 0.56 (0.15 to 0.96) |
| Eastern Sub-Saharan Africa | 1,842 (1,335-2,397) | 2.22 (1.61-2.89) | 5,246 (3,595-7,252) | 2.64 (1.81-3.65) | 184.8 | 0.58 (0.52 to 0.65) |
| Southern Sub-Saharan Africa | 868 (678-1,044) | 3.33 (2.6-4) | 1,314 (1,004-1,635) | 3.11 (2.37-3.87) | 51.4 | −0.82 (−1.05 to −0.6) |
| Western Sub-Saharan Africa | 448 (316-608) | 0.53 (0.37-0.71) | 1,263 (879-1,722) | 0.59 (0.41-0.8) | 181.9 | 0.33 (0.21 to 0.46) |

**Supplementary Table S19. Early-onset Lip and oral cavity cancer-related burden attributable to tobacco and alcohol in 2019 at the national level, and its temporal change from 1990 to 2019**

| Location | Deaths | | | | DALYs | | | |
|---|---|---|---|---|---|---|---|---|
| | Case number (in 2019) No. (95% UI) | Percent change in case number (%) | Rate per $10^5$ (in 2019) No. (95% UI) | EAPC No. (95% CI) | Case number (in 2019) No. (95% UI) | Percent change in case number (%) | Rate per $10^5$ (in 2019) No. (95% UI) | EAPC No. (95% CI) |
| Afghanistan | 11 (5-22) | 3.7 | 0.06 (0.03-0.12) | 2.4 (1.8 to 3) | 520 (240-995) | 3.7 | 2.86 (1.32-5.47) | 2.4 (1.9 to 3) |
| Albania | 2 (2-3) | -0.1 | 0.19 (0.13-0.27) | 1 (0.7 to 1.4) | 122 (83-172) | -0.1 | 9.41 (6.41-13.31) | 1 (0.7 to 1.3) |
| Algeria | 17 (11-26) | 1 | 0.08 (0.05-0.12) | 0 (-0.1 to 0.2) | 821 (525-1,224) | 1 | 3.66 (2.34-5.46) | 0 (-0.1 to 0.2) |
| American Samoa | 0 (0-0) | 0.3 | 0.06 (0.04-0.09) | 1.2 (0.9 to 1.5) | 1 (1-1) | 0.3 | 2.94 (1.84-4.34) | 1.1 (0.8 to 1.4) |
| Andorra | 0 (0-0) | 0.3 | 0.48 (0.3-0.73) | -0.2 (-0.4 to 0) | 9 (6-14) | 0.2 | 22.21 (14.16-33.38) | -0.3 (-0.4 to -0.1) |
| Angola | 31 (20-45) | 3.4 | 0.23 (0.15-0.33) | 1.9 (1.7 to 2.2) | 1,453 (958-2,117) | 3.5 | 10.74 (7.08-15.64) | 2 (1.8 to 2.2) |
| Antigua and Barbuda | 0 (0-0) | 1.1 | 0.15 (0.11-0.21) | 1.5 (1.3 to 1.7) | 3 (2-5) | 1.1 | 7.16 (5.03-9.64) | 1.5 (1.2 to 1.7) |
| Argentina | 51 (41-61) | 0 | 0.22 (0.18-0.27) | -1.7 (-2 to -1.5) | 2,427 (1,982-2,905) | 0 | 10.47 (8.55-12.53) | -1.6 (-1.9 to -1.4) |
| Armenia | 2 (2-3) | -0.1 | 0.15 (0.11-0.19) | -0.3 (-0.8 to 0.2) | 107 (82-138) | -0.2 | 7.23 (5.54-9.32) | -0.5 (-1 to 0) |
| Australia | 44 (37-53) | -0.3 | 0.38 (0.32-0.46) | -2.1 (-2.5 to -1.7) | 2,119 (1,748-2,528) | -0.3 | 18.36 (15.15-21.91) | -2.1 (-2.5 to -1.7) |
| Austria | 15 (12-18) | -0.5 | 0.36 (0.29-0.44) | -2.4 (-2.7 to -2.1) | 675 (554-818) | -0.5 | 16.73 (13.72-20.28) | -2.4 (-2.7 to -2.1) |
| Azerbaijan | 9 (6-12) | 1.3 | 0.15 (0.1-0.22) | 1.7 (1.2 to 2.2) | 410 (269-588) | 1.2 | 7.37 (4.84-10.57) | 1.5 (1 to 2) |
| Bahamas | 1 (0-1) | 0.4 | 0.4 (0.24-0.57) | 0 (-0.2 to 0.2) | 39 (23-55) | 0.4 | 18.82 (11.34-26.78) | -0.1 (-0.3 to 0.1) |
| Bahrain | 1 (0-1) | 1.4 | 0.07 (0.05-0.11) | -2 (-2.4 to -1.5) | 33 (21-47) | 1.3 | 3.52 (2.25-5.1) | -2.1 (-2.5 to -1.6) |
| Bangladesh | 426 (257-657) | 0.3 | 0.49 (0.3-0.76) | -0.9 (-1 to -0.7) | 19,891 (12,016-30,602) | 0.3 | 23.08 (13.95-35.52) | -0.9 (-1 to -0.8) |
| Barbados | 0 (0-0) | 0.2 | 0.26 (0.19-0.35) | 0.7 (0.4 to 1) | 17 (13-23) | 0.2 | 12.34 (8.98-16.33) | 0.6 (0.3 to 0.9) |
| Belarus | 40 (27-56) | -0.3 | 0.91 (0.61-1.28) | -1.2 (-1.6 to -0.9) | 1,799 (1,223-2,526) | -0.3 | 41.28 (28.06-57.98) | -1.3 (-1.6 to -1) |
| Belgium | 17 (14-22) | -0.4 | 0.35 (0.28-0.43) | -3 (-3.5 to -2.5) | 817 (658-1,008) | -0.4 | 16.31 (13.13-20.12) | -3 (-3.5 to -2.5) |
| Belize | 0 (0-0) | 5 | 0.14 (0.1-0.17) | 2.2 (1.8 to 2.6) | 15 (11-18) | 4.9 | 6.53 (5-8.22) | 2.1 (1.7 to 2.5) |
| Benin | 5 (3-8) | 2.3 | 0.09 (0.05-0.14) | 0.4 (0.3 to 0.5) | 235 (140-379) | 2.3 | 4.03 (2.4-6.5) | 0.4 (0.3 to 0.5) |
| Bermuda | 0 (0-0) | -0.4 | 0.3 (0.21-0.42) | -0.7 (-1.1 to -0.4) | 4 (3-5) | -0.4 | 13.72 (9.55-19.03) | -0.8 (-1.1 to -0.5) |
| Bhutan | 2 (1-4) | 0.1 | 0.51 (0.27-0.87) | -1.7 (-2 to -1.4) | 106 (57-181) | 0.1 | 24.25 (13.04-41.65) | -1.7 (-2 to -1.4) |

| | | | | | | | | |
|---|---|---|---|---|---|---|---|---|
| Bolivia | 6 (3-9) | 0.8 | 0.09 (0.05-0.15) | -0.5 (-0.6 to -0.3) | 280 (153-431) | 0.8 | 4.58 (2.5-7.05) | -0.5 (-0.6 to -0.3) |
| Bosnia and Herzegovina | 5 (3-7) | -0.2 | 0.32 (0.22-0.43) | 0.3 (0 to 0.5) | 220 (155-300) | -0.2 | 14.47 (10.21-19.76) | 0.2 (0 to 0.4) |
| Botswana | 7 (4-11) | 2.4 | 0.49 (0.27-0.8) | 0.3 (-0.4 to 1) | 307 (167-497) | 2.4 | 23.17 (12.66-37.57) | 0.3 (-0.4 to 1) |
| Brazil | 400 (340-461) | 0.4 | 0.35 (0.29-0.4) | -0.5 (-0.9 to 0) | 18,638 (15,869-21,486) | 0.4 | 16.14 (13.74-18.6) | -0.5 (-1 to -0.1) |
| Brunei Darussalam | 1 (0-1) | 0.2 | 0.19 (0.12-0.28) | -1.3 (-1.8 to -0.8) | 24 (15-34) | 0.1 | 9.05 (5.8-12.83) | -1.5 (-2 to -1) |
| Bulgaria | 26 (18-36) | 0 | 0.86 (0.6-1.2) | 0.8 (0.4 to 1.1) | 1,205 (834-1,666) | 0 | 39.76 (27.53-54.97) | 0.8 (0.4 to 1.1) |
| Burkina Faso | 17 (11-25) | 2.2 | 0.16 (0.11-0.24) | 0.5 (0.3 to 0.6) | 788 (511-1,159) | 2.2 | 7.7 (4.99-11.32) | 0.5 (0.3 to 0.6) |
| Burundi | 17 (10-28) | 0.5 | 0.31 (0.18-0.5) | -2.3 (-2.6 to -2) | 814 (476-1,295) | 0.5 | 14.85 (8.68-23.62) | -2.3 (-2.6 to -2) |
| Cabo Verde | 1 (1-2) | 20.7 | 0.39 (0.25-0.6) | 8.1 (6.2 to 10) | 54 (34-82) | 18.9 | 17.45 (11.19-26.66) | 7.7 (5.9 to 9.6) |
| Cambodia | 27 (18-41) | 2 | 0.31 (0.21-0.46) | 1.6 (1.5 to 1.7) | 1,325 (894-1,951) | 2.1 | 15.08 (10.17-22.2) | 1.7 (1.6 to 1.7) |
| Cameroon | 24 (15-37) | 2.6 | 0.17 (0.1-0.26) | 0.4 (0.4 to 0.5) | 1,130 (693-1,733) | 2.6 | 7.86 (4.82-12.05) | 0.4 (0.4 to 0.5) |
| Canada | 34 (27-41) | -0.4 | 0.21 (0.17-0.26) | -3 (-3.5 to -2.6) | 1,635 (1,334-1,994) | -0.4 | 10.11 (8.25-12.33) | -3 (-3.4 to -2.5) |
| Central African Republic | 5 (2-8) | 0.6 | 0.18 (0.08-0.33) | -0.9 (-0.9 to -0.8) | 209 (98-382) | 0.6 | 8.16 (3.81-14.93) | -0.9 (-1 to -0.8) |
| Chad | 7 (4-10) | 3.2 | 0.1 (0.05-0.15) | 1.7 (1.5 to 1.9) | 304 (167-478) | 3.2 | 4.45 (2.44-6.98) | 1.7 (1.5 to 1.9) |
| Chile | 9 (8-11) | 0.1 | 0.1 (0.08-0.12) | -0.2 (-0.4 to -0.1) | 454 (365-545) | 0.1 | 4.93 (3.97-5.93) | -0.3 (-0.4 to -0.1) |
| China | 1,414 (1,078-1,819) | 1.1 | 0.2 (0.15-0.25) | 2.9 (2.6 to 3.2) | 66,676 (51,303-85,349) | 1 | 9.25 (7.12-11.84) | 2.7 (2.4 to 3) |
| Colombia | 17 (12-24) | -0.2 | 0.07 (0.05-0.1) | -2.5 (-2.8 to -2.3) | 860 (593-1,188) | -0.2 | 3.45 (2.38-4.77) | -2.6 (-2.8 to -2.4) |
| Comoros | 1 (0-1) | 1.5 | 0.18 (0.09-0.29) | 1.1 (0.8 to 1.4) | 30 (16-50) | 1.5 | 8.05 (4.3-13.45) | 1.1 (0.8 to 1.4) |
| Republic of Congo | 6 (4-10) | 1.4 | 0.24 (0.13-0.38) | 0.1 (-0.5 to 0.7) | 300 (167-472) | 1.3 | 11.21 (6.25-17.63) | 0.1 (-0.5 to 0.6) |
| Cook Islands | 0 (0-0) | 0.8 | 0.3 (0.18-0.46) | 3.7 (3.3 to 4.1) | 1 (1-2) | 0.8 | 14.47 (8.52-21.86) | 3.7 (3.3 to 4.1) |
| Costa Rica | 3 (2-4) | 0.2 | 0.12 (0.08-0.17) | -1.6 (-1.9 to -1.3) | 150 (105-205) | 0.2 | 6.02 (4.2-8.22) | -1.6 (-2 to -1.3) |
| Côte d'Ivoire | 22 (13-34) | 1.9 | 0.17 (0.1-0.26) | 0.2 (-0.1 to 0.4) | 1,028 (630-1,593) | 1.9 | 7.87 (4.83-12.19) | 0.1 (-0.1 to 0.4) |
| Croatia | 10 (7-14) | -0.6 | 0.54 (0.37-0.76) | -3.3 (-3.6 to -3) | 459 (314-637) | -0.6 | 24.65 (16.84-34.2) | -3.4 (-3.7 to -3.1) |
| Cuba | 21 (15-28) | 0.2 | 0.39 (0.28-0.53) | 1.6 (1.4 to 1.8) | 936 (673-1,273) | 0.2 | 17.65 (12.68-23.99) | 1.4 (1.2 to 1.6) |
| Cyprus | 1 (1-1) | 0.7 | 0.16 (0.12-0.21) | -0.5 (-0.7 to -0.3) | 53 (41-69) | 0.7 | 7.7 (5.89-10.06) | -0.4 (-0.6 to -0.2) |
| Czech Republic | 26 (19-35) | -0.5 | 0.55 (0.4-0.72) | -2.9 (-3.2 to -2.6) | 1,211 (891-1,585) | -0.5 | 25.21 (18.55-33) | -2.8 (-3.1 to -2.5) |
| North Korea | 28 (16-46) | 0.3 | 0.2 (0.11-0.33) | 0.4 (0.2 to 0.6) | 1,307 (748-2,203) | 0.3 | 9.39 (5.38-15.83) | 0.3 (0.1 to 0.4) |
| Democratic Republic of the Congo | 41 (23-65) | 1.1 | 0.1 (0.06-0.16) | -0.9 (-1.4 to -0.5) | 1,920 (1,093-2,998) | 1 | 4.6 (2.62-7.18) | -1 (-1.4 to -0.5) |
| Denmark | 6 (5-8) | 0.4 | 0.24 (0.18-0.3) | -0.1 (-1.3 to 1.1) | 284 (220-364) | 0.4 | 11.07 (8.56-14.19) | -0.1 (-1.4 to 1.1) |
| Djibouti | 1 (0-2) | 2.3 | 0.16 (0.08-0.31) | 0.1 (-0.1 to 0.2) | 47 (22-92) | 2.2 | 7.39 (3.45-14.31) | 0 (-0.1 to 0.2) |

| | | | | | | | | |
|---|---|---|---|---|---|---|---|---|
| Dominica | 0 (0-0) | 0.1 | 0.32 (0.22-0.45) | 0.6 (0.3 to 0.9) | 5 (4-7) | 0.1 | 15.33 (10.61-21.33) | 0.5 (0.2 to 0.8) |
| Dominican Republic | 15 (9-23) | 2 | 0.26 (0.16-0.4) | 2.2 (2 to 2.3) | 741 (464-1,111) | 1.9 | 12.85 (8.04-19.26) | 2 (1.9 to 2.2) |
| Ecuador | 7 (5-10) | 1.5 | 0.08 (0.05-0.11) | 1.1 (0.7 to 1.4) | 349 (242-491) | 1.5 | 3.8 (2.63-5.34) | 1.1 (0.7 to 1.5) |
| Egypt | 15 (8-24) | 1.5 | 0.03 (0.02-0.05) | 0.6 (0.2 to 1) | 686 (392-1,096) | 1.5 | 1.33 (0.76-2.13) | 0.6 (0.2 to 1) |
| El Salvador | 3 (2-4) | 0.2 | 0.08 (0.05-0.11) | -0.7 (-1.2 to -0.2) | 129 (87-182) | 0.2 | 3.92 (2.65-5.54) | -0.7 (-1.2 to -0.2) |
| Equatorial Guinea | 1 (1-2) | 3.3 | 0.16 (0.08-0.3) | 0.4 (0.2 to 0.6) | 60 (29-107) | 3.6 | 8.04 (3.88-14.35) | 0.6 (0.4 to 0.8) |
| Eritrea | 7 (4-12) | 2 | 0.2 (0.11-0.34) | -0.1 (-0.5 to 0.3) | 327 (171-545) | 2 | 9.48 (4.94-15.79) | -0.1 (-0.5 to 0.3) |
| Estonia | 2 (2-3) | -0.6 | 0.41 (0.28-0.58) | -3.1 (-3.6 to -2.7) | 110 (75-155) | -0.6 | 18.98 (12.9-26.63) | -3.2 (-3.6 to -2.7) |
| Eswatini | 2 (1-4) | 1.6 | 0.36 (0.19-0.59) | 1.4 (0.8 to 2) | 102 (54-165) | 1.6 | 16.87 (8.97-27.21) | 1.4 (0.8 to 2.1) |
| Ethiopia | 55 (33-84) | 0.6 | 0.1 (0.06-0.16) | -1.8 (-2.2 to -1.3) | 2,647 (1,576-4,062) | 0.6 | 5.04 (3-7.74) | -1.6 (-2.1 to -1.2) |
| Fiji | 1 (1-2) | 0.2 | 0.24 (0.16-0.35) | 0.5 (0.2 to 0.9) | 53 (35-76) | 0.2 | 11.41 (7.59-16.4) | 0.5 (0.2 to 0.9) |
| Finland | 5 (4-5) | -0.2 | 0.19 (0.16-0.23) | -1 (-1.4 to -0.7) | 223 (187-262) | -0.2 | 9.57 (8.02-11.24) | -1 (-1.3 to -0.7) |
| France | 108 (86-135) | -0.7 | 0.38 (0.3-0.48) | -4.8 (-5.4 to -4.2) | 5,033 (4,008-6,274) | -0.7 | 17.7 (14.1-22.07) | -4.8 (-5.4 to -4.3) |
| Gabon | 3 (2-4) | 0.7 | 0.3 (0.16-0.48) | -0.8 (-1 to -0.7) | 131 (73-207) | 0.7 | 14.21 (7.88-22.46) | -0.9 (-1 to -0.8) |
| Gambia | 1 (1-2) | 2.8 | 0.1 (0.06-0.16) | 1.4 (1.1 to 1.7) | 51 (30-82) | 2.8 | 4.59 (2.71-7.34) | 1.4 (1.1 to 1.7) |
| Georgia | 8 (6-10) | 0 | 0.47 (0.37-0.6) | 2 (1.7 to 2.3) | 361 (281-458) | 0 | 21.83 (16.97-27.68) | 1.9 (1.6 to 2.2) |
| Germany | 121 (99-147) | -0.7 | 0.34 (0.28-0.41) | -3.3 (-3.9 to -2.8) | 5,708 (4,713-6,855) | -0.7 | 15.9 (13.13-19.1) | -3.3 (-3.8 to -2.8) |
| Ghana | 8 (5-13) | 1.5 | 0.05 (0.03-0.08) | -0.5 (-0.8 to -0.2) | 404 (247-604) | 1.5 | 2.43 (1.49-3.64) | -0.5 (-0.8 to -0.2) |
| Greece | 11 (9-13) | 0.3 | 0.24 (0.2-0.28) | 1.3 (1.2 to 1.4) | 509 (428-600) | 0.2 | 11.39 (9.57-13.42) | 1.1 (1 to 1.2) |
| Greenland | 0 (0-0) | -0.5 | 0.3 (0.21-0.43) | -1.5 (-1.9 to -1.1) | 4 (3-5) | -0.5 | 13.98 (9.61-19.65) | -1.6 (-2 to -1.2) |
| Grenada | 0 (0-0) | 0.4 | 0.33 (0.24-0.45) | 0.5 (0.2 to 0.9) | 8 (6-11) | 0.4 | 15.45 (11.24-20.87) | 0.4 (0 to 0.7) |
| Guam | 0 (0-0) | 2 | 0.31 (0.19-0.47) | 4.5 (4 to 5) | 12 (7-18) | 2 | 15.11 (8.85-22.18) | 4.4 (3.9 to 4.8) |
| Guatemala | 5 (3-7) | 0.7 | 0.05 (0.04-0.07) | -2.8 (-3.2 to -2.4) | 250 (174-347) | 0.8 | 2.61 (1.82-3.62) | -2.8 (-3.2 to -2.4) |
| Guinea | 13 (8-20) | 2 | 0.22 (0.13-0.35) | 1 (0.8 to 1.2) | 590 (359-919) | 2 | 10.32 (6.28-16.06) | 1 (0.8 to 1.2) |
| Guinea-Bissau | 1 (1-2) | 0.9 | 0.14 (0.09-0.22) | -0.4 (-0.4 to -0.3) | 63 (39-96) | 0.9 | 6.64 (4.13-10.16) | -0.4 (-0.4 to -0.3) |
| Guyana | 1 (1-1) | 0.3 | 0.21 (0.13-0.29) | 1.1 (0.8 to 1.3) | 40 (26-57) | 0.2 | 9.74 (6.37-13.74) | 1 (0.7 to 1.2) |
| Haiti | 12 (6-20) | 0.7 | 0.18 (0.1-0.3) | -0.9 (-1.2 to -0.7) | 556 (309-932) | 0.7 | 8.48 (4.72-14.23) | -0.9 (-1.2 to -0.6) |
| Honduras | 3 (2-5) | 1.2 | 0.06 (0.03-0.1) | -0.8 (-1 to -0.5) | 150 (83-237) | 1.2 | 2.91 (1.61-4.59) | -0.8 (-1.1 to -0.5) |
| Hungary | 59 (43-79) | -0.5 | 1.33 (0.98-1.77) | -4.2 (-5.1 to -3.4) | 2,654 (1,969-3,497) | -0.5 | 59.78 (44.34-78.77) | -4.3 (-5.1 to -3.4) |
| Iceland | 0 (0-0) | 0.2 | 0.24 (0.18-0.3) | -0.5 (-0.6 to -0.4) | 18 (14-23) | 0.2 | 11.21 (8.62-14.22) | -0.5 (-0.6 to -0.4) |
| India | 7,453 (5,759-9,424) | 1.2 | 0.98 (0.76-1.24) | 0.8 (0.7 to 0.9) | 355,122 (275,838-446,540) | 1.2 | 46.75 (36.31-58.79) | 0.8 (0.7 to 0.9) |
| Indonesia | 164 (98-254) | 1 | 0.11 (0.07-0.18) | 1 (0.9 to 1) | 7,501 (4,470-11,598) | 0.9 | 5.25 (3.13-8.12) | 0.9 (0.8 to 1) |

| | | | | | | | | |
|---|---|---|---|---|---|---|---|---|
| Iran | 17 (14-22) | 2.1 | 0.04 (0.03-0.05) | 2 (1.8 to 2.1) | 839 (653-1,041) | 2.1 | 1.78 (1.38-2.2) | 2.1 (1.9 to 2.2) |
| Iraq | 13 (8-21) | 0.7 | 0.05 (0.03-0.09) | -1.9 (-2 to -1.8) | 589 (359-958) | 0.7 | 2.55 (1.55-4.15) | -2 (-2.1 to -1.8) |
| Ireland | 5 (4-6) | 0 | 0.2 (0.16-0.25) | -1.4 (-1.6 to -1.2) | 222 (177-277) | 0 | 9.51 (7.58-11.89) | -1.4 (-1.6 to -1.1) |
| Israel | 4 (3-5) | 1.3 | 0.09 (0.06-0.11) | 0 (-0.5 to 0.5) | 186 (138-240) | 1.2 | 4.24 (3.15-5.48) | 0 (-0.4 to 0.5) |
| Italy | 73 (63-83) | -0.5 | 0.29 (0.25-0.33) | -2.3 (-2.4 to -2.2) | 3,400 (2,965-3,866) | -0.5 | 13.41 (11.69-15.24) | -2.3 (-2.4 to -2.2) |
| Jamaica | 1 (1-2) | 0.4 | 0.09 (0.06-0.13) | -0.4 (-1.1 to 0.3) | 68 (45-95) | 0.4 | 4.46 (2.95-6.22) | -0.4 (-1.1 to 0.3) |
| Japan | 120 (106-134) | -0.1 | 0.23 (0.2-0.26) | 0 (-0.4 to 0.3) | 5,789 (5,069-6,448) | -0.1 | 11.19 (9.79-12.46) | 0.1 (-0.3 to 0.4) |
| Jordan | 5 (3-7) | 2.3 | 0.08 (0.05-0.11) | -0.4 (-0.5 to -0.3) | 227 (153-326) | 2.3 | 3.59 (2.42-5.15) | -0.4 (-0.5 to -0.2) |
| Kazakhstan | 29 (23-37) | 0 | 0.32 (0.25-0.4) | -1.5 (-2 to -0.9) | 1,389 (1,092-1,743) | 0 | 14.96 (11.76-18.78) | -1.5 (-2.1 to -1) |
| Kenya | 86 (62-116) | 2.7 | 0.33 (0.24-0.45) | 1.7 (1.4 to 2) | 4,060 (2,936-5,461) | 2.7 | 15.58 (11.27-20.97) | 1.6 (1.3 to 1.9) |
| Kiribati | 1 (1-2) | 0.9 | 1.66 (1.02-2.5) | 0.5 (0.4 to 0.6) | 48 (29-72) | 0.9 | 79.76 (48.62-119.05) | 0.5 (0.4 to 0.6) |
| Kuwait | 1 (1-1) | 0.5 | 0.03 (0.02-0.05) | -1.5 (-1.9 to -1) | 46 (30-68) | 0.6 | 1.58 (1.03-2.33) | -1.5 (-1.9 to -1) |
| Kyrgyzstan | 6 (4-7) | -0.1 | 0.17 (0.13-0.22) | -1.6 (-1.9 to -1.4) | 271 (208-351) | -0.1 | 8.07 (6.18-10.44) | -1.7 (-1.9 to -1.5) |
| Laos | 10 (6-14) | 0.5 | 0.25 (0.16-0.36) | -1.2 (-1.5 to -0.9) | 473 (302-678) | 0.6 | 12.15 (7.75-17.4) | -1.1 (-1.4 to -0.8) |
| Latvia | 5 (4-7) | -0.6 | 0.65 (0.45-0.9) | -2 (-2.5 to -1.6) | 242 (171-332) | -0.6 | 29.6 (20.87-40.56) | -2.1 (-2.5 to -1.6) |
| Lebanon | 4 (2-6) | 0.5 | 0.14 (0.09-0.22) | -0.5 (-1 to 0) | 174 (110-271) | 0.5 | 6.51 (4.13-10.13) | -0.5 (-0.9 to 0) |
| Lesotho | 6 (3-9) | 2 | 0.48 (0.26-0.8) | 3 (2.7 to 3.4) | 258 (139-422) | 2 | 22.45 (12.14-36.77) | 3.1 (2.7 to 3.4) |
| Liberia | 3 (2-5) | 1.7 | 0.12 (0.07-0.19) | -0.6 (-1 to -0.1) | 139 (83-215) | 1.6 | 5.63 (3.34-8.7) | -0.6 (-1 to -0.2) |
| Libya | 3 (2-5) | 2.1 | 0.08 (0.05-0.13) | 1.8 (1.4 to 2.1) | 161 (100-249) | 2.1 | 3.91 (2.43-6.06) | 1.7 (1.4 to 2.1) |
| Lithuania | 10 (7-13) | -0.5 | 0.8 (0.56-1.11) | -1.1 (-1.5 to -0.8) | 436 (310-598) | -0.5 | 36.02 (25.61-49.33) | -1.2 (-1.6 to -0.9) |
| Luxembourg | 1 (1-1) | -0.2 | 0.36 (0.29-0.43) | -2.6 (-2.7 to -2.4) | 52 (42-62) | -0.2 | 16.87 (13.7-20.36) | -2.6 (-2.7 to -2.5) |
| Madagascar | 32 (20-47) | 1.1 | 0.24 (0.15-0.35) | -0.5 (-0.5 to -0.4) | 1,478 (963-2,177) | 1.1 | 11.19 (7.3-16.49) | -0.5 (-0.6 to -0.4) |
| Malawi | 12 (7-18) | 1.6 | 0.14 (0.08-0.2) | 0.6 (0.4 to 0.7) | 588 (357-884) | 1.6 | 6.53 (3.97-9.83) | 0.6 (0.5 to 0.8) |
| Malaysia | 35 (21-52) | 0.8 | 0.2 (0.12-0.3) | -0.9 (-1.3 to -0.5) | 1,641 (1,029-2,426) | 0.8 | 9.4 (5.89-13.9) | -1 (-1.4 to -0.5) |
| Maldives | 0 (0-0) | 1.5 | 0.11 (0.07-0.15) | -1.6 (-1.8 to -1.3) | 17 (11-23) | 1.6 | 5.21 (3.57-7.28) | -1.5 (-1.8 to -1.2) |
| Mali | 6 (4-9) | 1.9 | 0.06 (0.04-0.09) | 0.2 (0 to 0.3) | 265 (166-401) | 1.9 | 2.75 (1.72-4.15) | 0.2 (0 to 0.3) |
| Malta | 0 (0-1) | 0 | 0.25 (0.19-0.33) | -0.9 (-1.2 to -0.6) | 24 (18-31) | 0.1 | 12.03 (9.26-15.68) | -0.8 (-1.1 to -0.5) |
| Marshall Islands | 0 (0-0) | 1.3 | 0.32 (0.18-0.52) | 1.4 (1.3 to 1.5) | 5 (3-8) | 1.3 | 15.46 (8.68-24.94) | 1.4 (1.3 to 1.4) |
| Mauritania | 1 (0-1) | 0.6 | 0.03 (0.02-0.07) | -1 (-1.1 to -0.9) | 30 (15-59) | 0.6 | 1.56 (0.78-3.06) | -1 (-1.1 to -1) |
| Mauritius | 2 (2-3) | 0.7 | 0.37 (0.26-0.5) | 1.5 (0.9 to 2.1) | 113 (78-152) | 0.7 | 17.45 (12.07-23.33) | 1.4 (0.8 to 2) |
| Mexico | 62 (49-76) | 0.9 | 0.09 (0.07-0.12) | 0.7 (0.6 to 0.9) | 3,009 (2,406-3,690) | 0.9 | 4.54 (3.63-5.57) | 0.7 (0.5 to 0.9) |
| Micronesia (Federated States of) | 0 (0-0) | 0.2 | 0.35 (0.11-0.63) | 0.1 (0.1 to 0.2) | 9 (3-16) | 0.2 | 16.71 (5.26-29.75) | 0 (0 to 0.1) |

| | | | | | | | | |
|---|---|---|---|---|---|---|---|---|
| Monaco | 0 (0-0) | 0.1 | 0.19 (0.09-0.31) | 0.3 (0.1 to 0.6) | 1 (1-2) | 0.1 | 9.13 (4.27-14.68) | 0.3 (0.1 to 0.6) |
| Mongolia | 6 (4-9) | 1.1 | 0.32 (0.21-0.47) | -0.1 (-0.5 to 0.2) | 271 (182-398) | 1.2 | 14.98 (10.09-22.04) | -0.1 (-0.5 to 0.3) |
| Montenegro | 2 (1-2) | -0.1 | 0.54 (0.39-0.72) | -0.2 (-0.6 to 0.2) | 73 (53-97) | -0.1 | 24.72 (17.82-32.7) | -0.3 (-0.6 to 0.1) |
| Morocco | 14 (8-24) | 0.4 | 0.07 (0.04-0.13) | -0.4 (-0.5 to -0.2) | 638 (367-1,123) | 0.4 | 3.33 (1.91-5.86) | -0.5 (-0.6 to -0.3) |
| Mozambique | 11 (6-19) | 3.8 | 0.08 (0.04-0.15) | 3.2 (2.9 to 3.5) | 506 (265-885) | 3.8 | 3.8 (1.99-6.65) | 3.2 (2.9 to 3.5) |
| Myanmar | 71 (47-107) | 0.6 | 0.25 (0.16-0.37) | 0.4 (0.3 to 0.5) | 3,378 (2,226-5,030) | 0.6 | 11.68 (7.7-17.4) | 0.4 (0.3 to 0.5) |
| Namibia | 9 (5-14) | 2.9 | 0.74 (0.43-1.14) | 2.7 (2.3 to 3.1) | 445 (258-684) | 3 | 35.43 (20.53-54.47) | 2.7 (2.3 to 3.1) |
| Nauru | 0 (0-0) | 0.2 | 0.44 (0.23-0.66) | 0.1 (0 to 0.2) | 1 (1-2) | 0.2 | 21.41 (11.38-31.84) | 0.2 (0.1 to 0.2) |
| Nepal | 85 (52-123) | 0.7 | 0.53 (0.32-0.76) | 0 (-0.3 to 0.3) | 4,016 (2,473-5,806) | 0.7 | 24.81 (15.28-35.88) | 0 (-0.2 to 0.3) |
| Netherlands | 14 (11-18) | -0.3 | 0.19 (0.15-0.23) | -1.6 (-2 to -1.2) | 666 (529-822) | -0.3 | 8.87 (7.05-10.95) | -1.6 (-2 to -1.2) |
| New Zealand | 4 (3-5) | -0.1 | 0.2 (0.17-0.24) | -0.3 (-0.5 to -0.2) | 201 (168-236) | -0.1 | 10.11 (8.44-11.91) | -0.4 (-0.6 to -0.3) |
| Nicaragua | 1 (1-2) | 1 | 0.04 (0.03-0.06) | -0.1 (-0.4 to 0.2) | 72 (49-101) | 1 | 2.04 (1.41-2.89) | -0.1 (-0.4 to 0.2) |
| Niger | 3 (2-5) | 1.8 | 0.03 (0.02-0.06) | 0.5 (0.3 to 0.6) | 146 (75-243) | 1.8 | 1.54 (0.79-2.57) | 0.4 (0.3 to 0.6) |
| Nigeria | 36 (24-52) | 1.4 | 0.04 (0.02-0.05) | 0.1 (0 to 0.2) | 1,723 (1,154-2,520) | 1.4 | 1.71 (1.14-2.5) | 0.1 (0 to 0.2) |
| Niue | 0 (0-0) | -0.3 | 0.28 (0.15-0.46) | -0.5 (-0.6 to -0.3) | 0 (0-0) | -0.3 | 13.18 (6.78-21.99) | -0.5 (-0.7 to -0.4) |
| Macedonia | 3 (2-4) | -0.1 | 0.29 (0.21-0.4) | -1.3 (-1.7 to -0.9) | 146 (104-200) | -0.1 | 13.45 (9.61-18.41) | -1.4 (-1.8 to -0.9) |
| Northern Mariana Islands | 0 (0-0) | 1.1 | 1.11 (0.57-1.86) | 5.7 (4.9 to 6.6) | 10 (5-17) | 1 | 50.33 (25.81-84.86) | 5.4 (4.6 to 6.3) |
| Norway | 3 (3-4) | -0.3 | 0.13 (0.11-0.14) | -2.1 (-2.4 to -1.9) | 151 (129-172) | -0.3 | 6.06 (5.16-6.92) | -2.1 (-2.4 to -1.9) |
| Oman | 1 (1-2) | 0.6 | 0.03 (0.02-0.05) | -3.2 (-3.7 to -2.7) | 48 (28-82) | 0.6 | 1.54 (0.89-2.62) | -3.1 (-3.6 to -2.6) |
| Pakistan | 1,824 (1,248-2,578) | 1.8 | 1.63 (1.12-2.31) | 0.7 (0.4 to 1) | 85,943 (58,768-121,489) | 1.9 | 76.89 (52.58-108.69) | 0.8 (0.5 to 1.1) |
| Palau | 0 (0-1) | 0.7 | 4.04 (2.63-5.87) | 1.5 (1.4 to 1.5) | 18 (12-25) | 0.6 | 191.95 (125.43-272.58) | 1.4 (1.3 to 1.4) |
| Palestine | 1 (1-1) | 1.7 | 0.04 (0.03-0.05) | -0.4 (-0.6 to -0.2) | 48 (35-64) | 1.7 | 1.88 (1.36-2.53) | -0.4 (-0.6 to -0.2) |
| Panama | 2 (2-3) | 0.7 | 0.12 (0.08-0.17) | 0.3 (-0.1 to 0.7) | 121 (82-169) | 0.6 | 5.77 (3.9-8.02) | 0.2 (-0.1 to 0.6) |
| Papua New Guinea | 8 (5-14) | 1.7 | 0.17 (0.09-0.28) | 0.4 (0.2 to 0.6) | 408 (222-679) | 1.7 | 8.03 (4.38-13.36) | 0.4 (0.2 to 0.6) |
| Paraguay | 7 (5-11) | 1.1 | 0.19 (0.13-0.28) | -0.7 (-1.1 to -0.4) | 349 (232-505) | 1.1 | 9.4 (6.24-13.58) | -0.8 (-1.1 to -0.4) |
| Peru | 16 (10-23) | 0.5 | 0.09 (0.06-0.13) | 0 (-0.3 to 0.3) | 770 (495-1,132) | 0.5 | 4.32 (2.78-6.35) | -0.1 (-0.4 to 0.2) |
| Philippines | 148 (112-191) | 0.4 | 0.25 (0.19-0.33) | -1.4 (-1.6 to -1.2) | 7,189 (5,507-9,273) | 0.4 | 12.3 (9.42-15.86) | -1.4 (-1.7 to -1.2) |
| Poland | 110 (85-141) | -0.2 | 0.61 (0.47-0.78) | -1.6 (-2 to -1.2) | 5,022 (3,910-6,372) | -0.2 | 27.6 (21.48-35.01) | -1.6 (-1.9 to -1.2) |
| Portugal | 35 (27-44) | 0 | 0.74 (0.57-0.94) | -0.2 (-0.6 to 0.2) | 1,601 (1,254-2,037) | 0 | 34.13 (26.72-43.42) | -0.3 (-0.7 to 0.1) |
| Puerto Rico | 3 (2-4) | -0.6 | 0.18 (0.12-0.26) | -3 (-3.4 to -2.7) | 136 (92-192) | -0.6 | 8.59 (5.81-12.13) | -3 (-3.4 to -2.7) |
| Qatar | 1 (0-1) | 4.5 | 0.03 (0.02-0.05) | -1.6 (-2 to -1.1) | 35 (22-55) | 4.5 | 1.64 (1-2.53) | -1.5 (-1.9 to -1.1) |
| South Korea | 44 (35-54) | 0.3 | 0.17 (0.14-0.21) | 0.6 (0 to 1.2) | 2,086 (1,699-2,546) | 0.3 | 8.01 (6.52-9.77) | 0.4 (-0.1 to 1) |

| | | | | | | | | |
|---|---|---|---|---|---|---|---|---|
| Moldova | 11 (9-14) | -0.4 | 0.62 (0.49-0.76) | -2 (-2.5 to -1.5) | 520 (416-642) | -0.4 | 28.33 (22.64-34.92) | -2.1 (-2.6 to -1.6) |
| Romania | 110 (79-146) | 0.4 | 1.27 (0.91-1.69) | 1.6 (1.3 to 2) | 4,954 (3,551-6,541) | 0.3 | 57.22 (41.01-75.54) | 1.5 (1.2 to 1.9) |
| Russia | 610 (493-745) | 0.4 | 0.89 (0.72-1.09) | 0.6 (0 to 1.1) | 28,296 (22,924-34,461) | 0.4 | 41.32 (33.48-50.33) | 0.6 (0.1 to 1.1) |
| Rwanda | 20 (13-31) | 0.6 | 0.31 (0.2-0.47) | -2.2 (-2.6 to -1.8) | 985 (613-1,472) | 0.6 | 15.23 (9.48-22.76) | -2.2 (-2.6 to -1.7) |
| Saint Kitts and Nevis | 0 (0-0) | 0.2 | 0.16 (0.04-0.32) | -1.5 (-1.7 to -1.3) | 2 (1-5) | 0.1 | 7.1 (1.83-14.65) | -1.7 (-1.9 to -1.4) |
| Saint Lucia | 0 (0-1) | 0.8 | 0.45 (0.34-0.59) | 1 (0.8 to 1.3) | 20 (15-26) | 0.7 | 21.06 (16.06-27.34) | 0.9 (0.7 to 1.2) |
| Saint Vincent and the Grenadines | 0 (0-0) | 0.9 | 0.59 (0.45-0.76) | 2.3 (2 to 2.5) | 16 (12-20) | 0.8 | 27.29 (20.6-34.76) | 2.1 (1.8 to 2.3) |
| Samoa | 0 (0-0) | 0.5 | 0.2 (0.11-0.32) | 0.7 (0.4 to 1) | 10 (5-15) | 0.5 | 9.55 (5.11-14.89) | 0.6 (0.3 to 0.9) |
| San Marino | 0 (0-0) | 0.3 | 0.32 (0.15-0.55) | 0.4 (0.2 to 0.5) | 2 (1-4) | 0.3 | 15.22 (7.01-25.47) | 0.3 (0.2 to 0.5) |
| Sao Tome and Principe | 0 (0-0) | 3.4 | 0.06 (0.04-0.09) | 2.3 (2 to 2.6) | 3 (2-5) | 3.3 | 2.82 (1.71-4.46) | 2.3 (2 to 2.6) |
| Saudi Arabia | 16 (9-26) | 3.3 | 0.07 (0.04-0.11) | 1.4 (1 to 1.8) | 755 (445-1,217) | 3.4 | 3.1 (1.83-5) | 1.5 (1.1 to 1.9) |
| Senegal | 4 (2-7) | 1 | 0.05 (0.03-0.09) | -0.3 (-0.5 to -0.1) | 175 (95-297) | 1 | 2.38 (1.3-4.05) | -0.4 (-0.6 to -0.2) |
| Serbia | 23 (15-33) | -0.3 | 0.57 (0.38-0.82) | -1.1 (-1.4 to -0.8) | 1,036 (709-1,488) | -0.3 | 25.87 (17.72-37.18) | -1.1 (-1.4 to -0.8) |
| Seychelles | 1 (1-1) | 2.3 | 1.38 (0.94-1.97) | 2.2 (1.7 to 2.6) | 35 (24-49) | 2.3 | 64.63 (44.6-91.06) | 2.1 (1.6 to 2.6) |
| Sierra Leone | 5 (3-7) | 1.2 | 0.11 (0.07-0.17) | -0.6 (-0.8 to -0.5) | 220 (137-334) | 1.2 | 5.29 (3.3-8.04) | -0.7 (-0.8 to -0.5) |
| Singapore | 2 (2-3) | -0.2 | 0.07 (0.05-0.09) | -2.8 (-3.1 to -2.6) | 102 (75-132) | -0.2 | 3.3 (2.43-4.29) | -2.9 (-3.1 to -2.6) |
| Slovakia | 27 (18-40) | -0.5 | 1.04 (0.68-1.51) | -3.1 (-3.5 to -2.7) | 1,250 (820-1,798) | -0.5 | 47.51 (31.17-68.32) | -3.1 (-3.5 to -2.7) |
| Slovenia | 3 (2-5) | -0.6 | 0.35 (0.22-0.52) | -3.7 (-4.1 to -3.4) | 147 (92-215) | -0.6 | 16.4 (10.25-24.04) | -3.7 (-4 to -3.4) |
| Solomon Islands | 1 (0-2) | 2.2 | 0.34 (0.14-0.61) | 1.8 (1.6 to 2) | 53 (22-95) | 2.2 | 16.19 (6.62-28.81) | 1.8 (1.6 to 2) |
| Somalia | 6 (3-12) | 1.3 | 0.06 (0.03-0.13) | -1.7 (-2 to -1.3) | 278 (122-554) | 1.4 | 2.94 (1.29-5.85) | -1.6 (-2 to -1.2) |
| South Africa | 80 (66-96) | -0.1 | 0.26 (0.22-0.32) | -3.2 (-3.8 to -2.6) | 3,804 (3,141-4,513) | -0.1 | 12.49 (10.31-14.82) | -3.2 (-3.8 to -2.6) |
| South Sudan | 3 (1-6) | 0.4 | 0.07 (0.04-0.15) | -0.2 (-0.5 to 0.1) | 142 (69-285) | 0.4 | 3.36 (1.63-6.76) | -0.2 (-0.5 to 0) |
| Spain | 69 (54-85) | -0.5 | 0.33 (0.26-0.41) | -3.5 (-3.8 to -3.2) | 3,291 (2,640-4,069) | -0.5 | 15.93 (12.78-19.69) | -3.5 (-3.8 to -3.2) |
| Sri Lanka | 50 (31-76) | 1 | 0.46 (0.28-0.7) | 2.1 (1.6 to 2.5) | 2,319 (1,461-3,524) | 1 | 21.27 (13.4-32.32) | 2 (1.5 to 2.4) |
| Sudan | 7 (3-12) | 0.3 | 0.03 (0.02-0.06) | -1.9 (-2.3 to -1.5) | 326 (158-578) | 0.3 | 1.57 (0.76-2.77) | -1.9 (-2.4 to -1.5) |
| Suriname | 1 (0-1) | 1.1 | 0.19 (0.13-0.27) | 1.2 (0.9 to 1.6) | 26 (18-36) | 1 | 8.92 (6.22-12.54) | 1.1 (0.8 to 1.4) |
| Sweden | 4 (4-5) | -0.4 | 0.1 (0.08-0.12) | -2 (-2.2 to -1.9) | 218 (175-259) | -0.4 | 4.86 (3.91-5.78) | -2 (-2.1 to -1.8) |
| Switzerland | 9 (7-11) | -0.1 | 0.22 (0.18-0.28) | -1.6 (-2.2 to -1) | 424 (344-526) | -0.1 | 10.63 (8.61-13.17) | -1.6 (-2.2 to -1) |
| Syria | 2 (1-3) | 0.2 | 0.03 (0.02-0.04) | -0.3 (-0.6 to 0.1) | 98 (64-143) | 0.2 | 1.32 (0.87-1.93) | -0.4 (-0.7 to -0.1) |
| Taiwan | 321 (225-445) | 2.6 | 2.75 (1.92-3.81) | 4.2 (3.2 to 5.2) | 15,054 (10,631-20,816) | 2.4 | 128.82 (90.98-178.13) | 4.1 (3.1 to 5) |
| Tajikistan | 4 (2-5) | 0.8 | 0.07 (0.05-0.1) | -1.6 (-2.2 to -1) | 171 (119-237) | 0.7 | 3.45 (2.39-4.77) | -1.7 (-2.3 to -1.1) |

| | | | | | | | | |
|---|---|---|---|---|---|---|---|---|
| Thailand | 188 (124-272) | 0.4 | 0.53 (0.35-0.76) | 0 (-0.4 to 0.3) | 8,893 (5,938-12,707) | 0.3 | 24.92 (16.64-35.61) | -0.2 (-0.6 to 0.1) |
| Timor-Leste | 1 (0-1) | 1 | 0.13 (0.03-0.23) | 0.6 (0.1 to 1) | 39 (9-72) | 1 | 6.02 (1.4-11.05) | 0.6 (0.2 to 1) |
| Togo | 5 (3-7) | 1.9 | 0.12 (0.07-0.19) | 0.7 (0.6 to 0.8) | 218 (131-335) | 1.8 | 5.56 (3.33-8.53) | 0.6 (0.5 to 0.7) |
| Tokelau | 0 (0-0) | 0.2 | 0.21 (0.11-0.33) | 0.8 (0.6 to 0.9) | 0 (0-0) | 0.2 | 10.02 (5.35-15.9) | 0.7 (0.6 to 0.9) |
| Tonga | 0 (0-0) | 0.7 | 0.14 (0.08-0.23) | 1.8 (1.5 to 2.1) | 3 (2-5) | 0.7 | 6.59 (3.9-10.59) | 1.8 (1.5 to 2.1) |
| Trinidad and Tobago | 1 (1-2) | 0.1 | 0.19 (0.13-0.26) | -0.7 (-1 to -0.5) | 63 (42-87) | 0.1 | 9.01 (6.05-12.46) | -0.7 (-1 to -0.5) |
| Tunisia | 10 (6-16) | 1.3 | 0.17 (0.1-0.26) | 1.3 (1 to 1.5) | 484 (300-746) | 1.3 | 8.02 (4.96-12.35) | 1.2 (1 to 1.5) |
| Turkey | 35 (24-49) | 0 | 0.08 (0.05-0.11) | -2.3 (-2.7 to -2) | 1,653 (1,152-2,298) | 0 | 3.62 (2.52-5.03) | -2.3 (-2.6 to -2) |
| Turkmenistan | 9 (7-13) | 1.6 | 0.36 (0.26-0.48) | 1.6 (1.3 to 1.8) | 452 (334-610) | 1.6 | 17.14 (12.68-23.16) | 1.6 (1.3 to 1.8) |
| Tuvalu | 0 (0-0) | 0.4 | 0.26 (0.15-0.41) | 0.2 (0.1 to 0.3) | 1 (0-1) | 0.4 | 12.32 (7.1-19.26) | 0.2 (0.2 to 0.3) |
| Uganda | 66 (44-97) | 2.8 | 0.35 (0.23-0.52) | 1.2 (1 to 1.4) | 3,243 (2,135-4,734) | 2.9 | 17.17 (11.31-25.07) | 1.3 (1 to 1.5) |
| Ukraine | 287 (216-385) | 1.1 | 1.38 (1.04-1.85) | 2.4 (1.9 to 3) | 13,286 (10,020-17,613) | 1.1 | 63.93 (48.21-84.74) | 2.5 (1.9 to 3) |
| United Arab Emirates | 15 (7-30) | 5.3 | 0.22 (0.1-0.43) | -0.7 (-1.6 to 0.2) | 720 (334-1,418) | 5.2 | 10.34 (4.79-20.35) | -0.7 (-1.5 to 0.2) |
| UK | 92 (81-102) | 0 | 0.3 (0.26-0.33) | -0.3 (-0.5 to -0.1) | 4,529 (3,965-5,023) | 0.1 | 14.86 (13.01-16.48) | -0.3 (-0.5 to -0.1) |
| Tanzania | 74 (46-118) | 1.5 | 0.28 (0.17-0.44) | 0.2 (0.2 to 0.3) | 3,499 (2,184-5,574) | 1.5 | 13.12 (8.19-20.9) | 0.2 (0.2 to 0.3) |
| USA | 268 (238-293) | -0.3 | 0.18 (0.16-0.19) | -1.8 (-2 to -1.7) | 13,257 (11,835-14,559) | -0.3 | 8.81 (7.86-9.67) | -1.8 (-2 to -1.6) |
| United States Virgin Islands | 0 (0-0) | 0 | 0.28 (0.08-0.5) | 1 (0.7 to 1.2) | 5 (2-10) | -0.1 | 12.62 (3.64-22.47) | 0.8 (0.6 to 1) |
| Uruguay | 6 (4-7) | 0 | 0.35 (0.27-0.44) | -0.6 (-0.7 to -0.5) | 271 (214-337) | 0 | 16.46 (13-20.48) | -0.5 (-0.6 to -0.4) |
| Uzbekistan | 44 (33-56) | 2.7 | 0.24 (0.18-0.31) | 1.7 (1.1 to 2.3) | 2,177 (1,652-2,793) | 2.6 | 12.04 (9.14-15.45) | 1.6 (1 to 2.2) |
| Vanuatu | 0 (0-1) | 1.5 | 0.21 (0.11-0.39) | 0.5 (0.3 to 0.7) | 15 (8-27) | 1.5 | 10.38 (5.26-18.72) | 0.5 (0.3 to 0.7) |
| Venezuela | 21 (14-30) | 0.8 | 0.15 (0.1-0.21) | 0.3 (0.1 to 0.5) | 1,030 (694-1,439) | 0.8 | 7.15 (4.82-10) | 0.3 (0.1 to 0.4) |
| Vietnam | 355 (220-529) | 5.5 | 0.67 (0.42-1) | 5.5 (5 to 5.9) | 16,589 (10,367-24,426) | 5.5 | 31.29 (19.55-46.07) | 5.5 (5 to 5.9) |
| Yemen | 10 (6-16) | 1.2 | 0.07 (0.04-0.1) | -1.1 (-1.4 to -0.9) | 486 (292-763) | 1.2 | 3.11 (1.87-4.88) | -1.1 (-1.3 to -0.9) |
| Zambia | 30 (18-46) | 2.4 | 0.33 (0.2-0.5) | 0.8 (0.7 to 0.9) | 1,436 (882-2,166) | 2.4 | 15.91 (9.78-24) | 0.8 (0.7 to 0.9) |
| Zimbabwe | 18 (11-26) | 2 | 0.23 (0.15-0.35) | 2.1 (1.9 to 2.4) | 834 (528-1,258) | 2 | 11.08 (7.02-16.73) | 2.1 (1.8 to 2.4) |

**Supplementary Table S20. Early-onset Other pharyngeal cancer-related burden attributable to tobacco and alcohol in 2019 at the national level, and its temporal change from 1990 to 2019**

| Location | Deaths | | | | DALYs | | | |
|---|---|---|---|---|---|---|---|---|
| | Case number (in 2019) No. (95% UI) | Percent change in case number (%) | Rate per $10^5$ (in 2019) No. (95% UI) | EAPC No. (95% CI) | Case number (in 2019) No. (95% UI) | Percent change in case number (%) | Rate per $10^5$ (in 2019) No. (95% UI) | EAPC No. (95% CI) |
| Afghanistan | 3 (1-5) | 4.7 | 0.01 (0.01-0.03) | 3 (2.2 to 3.9) | 117 (60-218) | 4.7 | 0.64 (0.33-1.2) | 3.1 (2.3 to 3.9) |
| Albania | 1 (1-2) | -0.1 | 0.1 (0.07-0.15) | 1.2 (0.8 to 1.6) | 62 (41-91) | -0.1 | 4.82 (3.17-7.02) | 1.1 (0.8 to 1.5) |
| Algeria | 16 (10-23) | 1.3 | 0.07 (0.05-0.1) | 0.7 (0.6 to 0.8) | 715 (460-1,042) | 1.3 | 3.19 (2.05-4.65) | 0.7 (0.6 to 0.8) |
| American Samoa | 0 (0-0) | 1 | 0.05 (0.03-0.07) | 2.8 (2.3 to 3.3) | 1 (0-1) | 1.0 | 2.22 (1.35-3.4) | 2.8 (2.3 to 3.4) |
| Andorra | 0 (0-0) | 0.5 | 0.35 (0.22-0.52) | 0.6 (0.4 to 0.7) | 7 (4-10) | 0.5 | 16.02 (10.22-23.71) | 0.6 (0.4 to 0.8) |
| Angola | 8 (5-12) | 3 | 0.06 (0.04-0.09) | 1.5 (1.4 to 1.7) | 362 (239-538) | 3.1 | 2.68 (1.77-3.97) | 1.6 (1.4 to 1.8) |
| Antigua and Barbuda | 0 (0-0) | 0.7 | 0.11 (0.08-0.15) | 0.6 (0.2 to 0.9) | 2 (2-3) | 0.6 | 5.05 (3.62-6.8) | 0.5 (0.2 to 0.9) |
| Argentina | 17 (13-20) | -0.5 | 0.07 (0.06-0.09) | -4 (-4.6 to -3.4) | 752 (592-923) | -0.5 | 3.24 (2.55-3.98) | -4 (-4.6 to -3.4) |
| Armenia | 1 (1-1) | -0.1 | 0.05 (0.04-0.06) | 0.1 (-0.7 to 0.9) | 33 (25-42) | -0.1 | 2.23 (1.7-2.87) | -0.1 (-0.8 to 0.7) |
| Australia | 19 (15-24) | 0.1 | 0.16 (0.13-0.21) | -0.5 (-0.8 to -0.2) | 862 (675-1,088) | 0.1 | 7.47 (5.85-9.43) | -0.5 (-0.8 to -0.2) |
| Austria | 12 (9-15) | -0.2 | 0.29 (0.23-0.36) | -0.4 (-0.9 to 0.1) | 530 (418-660) | -0.2 | 13.14 (10.35-16.36) | -0.4 (-0.9 to 0.1) |
| Azerbaijan | 4 (3-6) | 1.5 | 0.08 (0.05-0.1) | 2.3 (1.8 to 2.8) | 197 (136-268) | 1.4 | 3.55 (2.45-4.82) | 2.1 (1.6 to 2.6) |
| Bahamas | 1 (0-1) | 0.3 | 0.26 (0.17-0.37) | -0.2 (-0.5 to 0.1) | 24 (16-35) | 0.3 | 11.94 (7.88-17.12) | -0.3 (-0.6 to 0) |
| Bahrain | 0 (0-0) | 1.9 | 0.02 (0.01-0.04) | -1.4 (-2 to -0.9) | 10 (7-15) | 1.8 | 1.11 (0.7-1.66) | -1.5 (-2.1 to -1) |
| Bangladesh | 212 (104-398) | 0.9 | 0.25 (0.12-0.46) | 0.3 (0.3 to 0.4) | 9,520 (4,651-17,873) | 0.9 | 11.05 (5.4-20.74) | 0.3 (0.2 to 0.4) |
| Barbados | 0 (0-0) | 0 | 0.22 (0.15-0.3) | 0.1 (-0.1 to 0.4) | 14 (10-19) | 0.0 | 9.89 (6.83-13.58) | 0 (-0.2 to 0.2) |
| Belarus | 25 (17-35) | 0.4 | 0.57 (0.39-0.81) | 0.8 (0.1 to 1.6) | 1,117 (760-1,572) | 0.4 | 25.63 (17.44-36.08) | 0.8 (0 to 1.5) |
| Belgium | 13 (10-17) | -0.2 | 0.27 (0.21-0.33) | -1.8 (-2.4 to -1.1) | 610 (480-755) | -0.1 | 12.18 (9.58-15.06) | -1.7 (-2.4 to -1.1) |
| Belize | 0 (0-0) | 3.7 | 0.07 (0.06-0.1) | 1.4 (1.2 to 1.7) | 8 (6-10) | 3.7 | 3.44 (2.61-4.45) | 1.4 (1.2 to 1.6) |
| Benin | 1 (1-1) | 1.8 | 0.02 (0.01-0.02) | -0.3 (-0.4 to -0.1) | 42 (24-66) | 1.8 | 0.72 (0.4-1.14) | -0.2 (-0.3 to -0.1) |
| Bermuda | 0 (0-0) | -0.5 | 0.18 (0.13-0.26) | -1.1 (-1.6 to -0.6) | 2 (2-3) | -0.5 | 8.28 (5.76-11.42) | -1.2 (-1.7 to -0.7) |
| Bhutan | 1 (0-2) | 0.5 | 0.24 (0.1-0.4) | -1 (-1.3 to -0.7) | 47 (20-81) | 0.4 | 10.82 (4.7-18.48) | -1 (-1.3 to -0.7) |

| | | | | | | | | |
|---|---|---|---|---|---|---|---|---|
| Bolivia | 2 (1-3) | 0.6 | 0.03 (0.02-0.05) | -1 (-1.2 to -0.8) | 88 (52-132) | 0.6 | 1.44 (0.86-2.16) | -1 (-1.2 to -0.8) |
| Bosnia and Herzegovina | 5 (3-6) | -0.1 | 0.31 (0.21-0.43) | 0.5 (0.2 to 0.8) | 211 (147-290) | -0.1 | 13.88 (9.69-19.1) | 0.5 (0.2 to 0.7) |
| Botswana | 2 (1-3) | 2.9 | 0.14 (0.08-0.24) | 0.9 (0.3 to 1.5) | 85 (48-143) | 2.9 | 6.44 (3.66-10.85) | 0.9 (0.3 to 1.5) |
| Brazil | 339 (291-387) | 0.5 | 0.29 (0.25-0.33) | -0.4 (-0.8 to 0) | 15,330 (13,113-17,444) | 0.5 | 13.27 (11.35-15.1) | -0.4 (-0.9 to 0) |
| Brunei Darussalam | 0 (0-0) | 1.8 | 0.12 (0.08-0.17) | 2.7 (2.2 to 3.2) | 14 (9-20) | 1.7 | 5.35 (3.47-7.59) | 2.5 (2 to 3) |
| Bulgaria | 18 (13-25) | 0.2 | 0.61 (0.43-0.83) | 2.1 (1.7 to 2.5) | 834 (598-1,118) | 0.2 | 27.53 (19.73-36.9) | 2.1 (1.7 to 2.5) |
| Burkina Faso | 2 (2-3) | 1.9 | 0.02 (0.01-0.03) | 0.1 (-0.1 to 0.3) | 113 (73-165) | 2.0 | 1.1 (0.72-1.61) | 0.2 (0 to 0.4) |
| Burundi | 5 (3-8) | 0.5 | 0.09 (0.05-0.15) | -2.3 (-2.6 to -2) | 214 (125-359) | 0.5 | 3.9 (2.27-6.54) | -2.3 (-2.6 to -2) |
| Cabo Verde | 0 (0-0) | 5 | 0.03 (0.02-0.05) | 3.7 (2.8 to 4.7) | 4 (3-7) | 4.9 | 1.44 (0.9-2.26) | 3.7 (2.7 to 4.6) |
| Cambodia | 5 (3-8) | 1.8 | 0.06 (0.04-0.09) | 1.4 (1.2 to 1.6) | 240 (161-360) | 1.8 | 2.73 (1.83-4.09) | 1.4 (1.3 to 1.6) |
| Cameroon | 4 (2-6) | 2 | 0.03 (0.02-0.04) | -0.3 (-0.3 to -0.2) | 196 (118-308) | 2.1 | 1.36 (0.82-2.14) | -0.1 (-0.2 to -0.1) |
| Canada | 12 (10-16) | -0.2 | 0.08 (0.06-0.1) | -1.5 (-1.8 to -1.1) | 570 (452-717) | -0.2 | 3.53 (2.8-4.44) | -1.4 (-1.8 to -1.1) |
| Central African Republic | 1 (0-2) | 0.6 | 0.04 (0.02-0.07) | -0.7 (-0.8 to -0.6) | 43 (22-81) | 0.6 | 1.7 (0.86-3.15) | -0.7 (-0.8 to -0.6) |
| Chad | 1 (1-2) | 2.6 | 0.02 (0.01-0.03) | 1.1 (1 to 1.2) | 54 (32-83) | 2.7 | 0.8 (0.46-1.22) | 1.2 (1.1 to 1.3) |
| Chile | 4 (3-5) | 0 | 0.04 (0.03-0.05) | -0.7 (-0.9 to -0.5) | 174 (140-214) | 0.0 | 1.89 (1.52-2.33) | -0.7 (-0.8 to -0.5) |
| China | 380 (286-484) | 0 | 0.05 (0.04-0.07) | -0.7 (-1.1 to -0.4) | 17,406 (13,241-22,021) | 0.0 | 2.42 (1.84-3.06) | -0.8 (-1.2 to -0.5) |
| Colombia | 6 (4-8) | -0.2 | 0.02 (0.01-0.03) | -2.5 (-2.7 to -2.4) | 263 (175-373) | -0.2 | 1.06 (0.7-1.5) | -2.6 (-2.8 to -2.4) |
| Comoros | 0 (0-0) | 1.4 | 0.05 (0.02-0.1) | 0.9 (0.5 to 1.2) | 9 (4-17) | 1.4 | 2.37 (1.08-4.62) | 0.9 (0.5 to 1.2) |
| Republic of Congo | 2 (1-3) | 1.7 | 0.07 (0.04-0.12) | 0.6 (0.1 to 1.2) | 83 (48-140) | 1.7 | 3.11 (1.78-5.23) | 0.6 (0.1 to 1.1) |
| Cook Islands | 0 (0-0) | 0.6 | 0.04 (0.02-0.05) | 3.5 (3.1 to 3.8) | 0 (0-0) | 0.7 | 1.79 (1.02-2.61) | 3.6 (3.2 to 3.9) |
| Costa Rica | 1 (1-2) | 0.3 | 0.05 (0.03-0.08) | -1.2 (-1.4 to -1) | 62 (41-88) | 0.3 | 2.5 (1.64-3.54) | -1.2 (-1.4 to -1) |
| Côte d'Ivoire | 4 (3-7) | 1.5 | 0.03 (0.02-0.05) | -0.3 (-0.5 to 0) | 198 (123-312) | 1.6 | 1.52 (0.94-2.39) | -0.2 (-0.4 to 0) |
| Croatia | 10 (7-14) | -0.5 | 0.52 (0.36-0.74) | -2.3 (-2.6 to -1.9) | 432 (298-611) | -0.5 | 23.16 (15.99-32.77) | -2.3 (-2.6 to -2) |
| Cuba | 11 (8-15) | 0 | 0.21 (0.15-0.29) | 1.2 (0.6 to 1.8) | 494 (348-671) | 0.0 | 9.32 (6.56-12.65) | 1 (0.5 to 1.6) |
| Cyprus | 0 (0-0) | 0.7 | 0.04 (0.03-0.05) | -0.5 (-0.7 to -0.3) | 12 (9-16) | 0.8 | 1.79 (1.34-2.34) | -0.3 (-0.5 to -0.1) |
| Czech Republic | 22 (16-31) | -0.1 | 0.46 (0.33-0.65) | -1 (-1.3 to -0.7) | 1,001 (707-1,391) | -0.1 | 20.84 (14.72-28.96) | -0.9 (-1.2 to -0.7) |
| North Korea | 7 (4-12) | 0 | 0.05 (0.03-0.09) | -0.6 (-0.9 to -0.3) | 327 (178-555) | 0.0 | 2.35 (1.28-3.99) | -0.6 (-0.9 to -0.4) |
| Democratic Republic of the Congo | 10 (5-17) | 1.1 | 0.02 (0.01-0.04) | -0.7 (-1.3 to -0.2) | 458 (246-749) | 1.1 | 1.1 (0.59-1.79) | -0.8 (-1.3 to -0.2) |
| Denmark | 6 (5-8) | 0.2 | 0.24 (0.18-0.32) | -0.2 (-0.9 to 0.6) | 282 (209-375) | 0.2 | 10.98 (8.14-14.63) | -0.1 (-0.8 to 0.7) |
| Djibouti | 1 (0-1) | 2.4 | 0.09 (0.04-0.16) | 0.1 (-0.1 to 0.3) | 25 (12-45) | 2.3 | 3.84 (1.82-7.07) | 0.1 (-0.1 to 0.3) |

| | | | | | | | | |
|---|---|---|---|---|---|---|---|---|
| Dominica | 0 (0-0) | -0.1 | 0.25 (0.16-0.36) | 0.1 (-0.1 to 0.3) | 4 (3-6) | -0.1 | 11.19 (7.53-16.25) | 0 (-0.2 to 0.2) |
| Dominican Republic | 9 (6-15) | 2 | 0.16 (0.1-0.25) | 2.4 (2.3 to 2.5) | 437 (259-686) | 1.9 | 7.57 (4.49-11.9) | 2.3 (2.2 to 2.4) |
| Ecuador | 2 (1-3) | 0.5 | 0.02 (0.02-0.03) | -0.8 (-1.1 to -0.6) | 106 (71-153) | 0.5 | 1.15 (0.77-1.66) | -0.8 (-1 to -0.5) |
| Egypt | 9 (5-14) | 1.6 | 0.02 (0.01-0.03) | 0.7 (0.5 to 1) | 412 (244-660) | 1.6 | 0.8 (0.47-1.28) | 0.7 (0.5 to 1) |
| El Salvador | 1 (1-1) | -0.2 | 0.03 (0.02-0.05) | -2.2 (-2.8 to -1.5) | 49 (32-70) | -0.2 | 1.49 (0.99-2.14) | -2.2 (-2.8 to -1.6) |
| Equatorial Guinea | 0 (0-1) | 4.1 | 0.05 (0.02-0.08) | 1.2 (1 to 1.4) | 16 (8-28) | 4.3 | 2.14 (1.09-3.74) | 1.3 (1.1 to 1.5) |
| Eritrea | 3 (1-4) | 2 | 0.08 (0.04-0.13) | 0.1 (-0.3 to 0.4) | 122 (67-197) | 2.0 | 3.54 (1.93-5.71) | 0.1 (-0.3 to 0.5) |
| Estonia | 2 (1-3) | -0.4 | 0.37 (0.25-0.53) | -2 (-2.4 to -1.6) | 95 (64-137) | -0.4 | 16.41 (11.03-23.48) | -2 (-2.4 to -1.6) |
| Eswatini | 1 (0-1) | 1.6 | 0.09 (0.05-0.15) | 1.3 (0.9 to 1.7) | 26 (13-43) | 1.6 | 4.24 (2.11-7.09) | 1.3 (0.9 to 1.8) |
| Ethiopia | 11 (6-17) | 0.9 | 0.02 (0.01-0.03) | -1.1 (-1.7 to -0.6) | 499 (285-785) | 0.9 | 0.95 (0.54-1.5) | -1.1 (-1.6 to -0.6) |
| Fiji | 0 (0-0) | 0.5 | 0.05 (0.03-0.07) | 1.1 (0.8 to 1.5) | 11 (7-16) | 0.5 | 2.37 (1.53-3.39) | 1.2 (0.8 to 1.6) |
| Finland | 2 (2-3) | -0.3 | 0.09 (0.07-0.12) | -1.2 (-1.5 to -0.9) | 102 (76-133) | -0.3 | 4.35 (3.27-5.71) | -1.1 (-1.4 to -0.9) |
| France | 105 (80-137) | -0.7 | 0.37 (0.28-0.48) | -5.1 (-5.7 to -4.6) | 4,726 (3,643-6,141) | -0.7 | 16.62 (12.81-21.6) | -5.1 (-5.6 to -4.6) |
| Gabon | 1 (0-1) | 0.9 | 0.08 (0.05-0.14) | -0.4 (-0.6 to -0.3) | 36 (21-60) | 0.9 | 3.87 (2.28-6.47) | -0.5 (-0.6 to -0.3) |
| Gambia | 0 (0-0) | 2.1 | 0.02 (0.01-0.04) | 0.6 (0.4 to 0.8) | 11 (6-19) | 2.1 | 1.01 (0.58-1.69) | 0.7 (0.5 to 0.9) |
| Georgia | 4 (3-5) | 0.6 | 0.25 (0.19-0.33) | 4 (3.3 to 4.8) | 191 (145-245) | 0.6 | 11.54 (8.79-14.83) | 3.9 (3.2 to 4.6) |
| Germany | 120 (95-152) | -0.6 | 0.34 (0.26-0.42) | -3 (-3.4 to -2.7) | 5,411 (4,283-6,772) | -0.6 | 15.07 (11.93-18.87) | -3.1 (-3.4 to -2.7) |
| Ghana | 3 (2-4) | 1.7 | 0.02 (0.01-0.02) | -0.4 (-0.8 to -0.1) | 127 (78-190) | 1.7 | 0.77 (0.47-1.15) | -0.4 (-0.8 to -0.1) |
| Greece | 3 (2-4) | 0.5 | 0.07 (0.05-0.09) | 1.7 (1.6 to 1.9) | 141 (111-176) | 0.5 | 3.15 (2.47-3.95) | 1.7 (1.5 to 1.8) |
| Greenland | 0 (0-0) | -0.6 | 0.47 (0.31-0.7) | -1.6 (-2.2 to -1) | 6 (4-8) | -0.6 | 21.1 (13.92-31) | -1.7 (-2.2 to -1.1) |
| Grenada | 0 (0-0) | 0.5 | 0.18 (0.13-0.25) | 0.3 (0.2 to 0.4) | 4 (3-6) | 0.4 | 8.22 (6.04-11.08) | 0.1 (0 to 0.3) |
| Guam | 0 (0-0) | 2.5 | 0.07 (0.04-0.11) | 4.7 (4.3 to 5.1) | 3 (2-4) | 2.5 | 3.63 (2.08-5.36) | 4.7 (4.3 to 5.1) |
| Guatemala | 2 (2-3) | 0.5 | 0.02 (0.02-0.03) | -3.6 (-4 to -3.2) | 115 (80-158) | 0.5 | 1.21 (0.83-1.65) | -3.6 (-4 to -3.2) |
| Guinea | 1 (1-2) | 1.9 | 0.03 (0.02-0.04) | 0.8 (0.6 to 0.9) | 66 (40-106) | 1.9 | 1.16 (0.7-1.86) | 0.8 (0.7 to 1) |
| Guinea-Bissau | 0 (0-0) | 0.7 | 0.02 (0.01-0.04) | -0.9 (-1 to -0.8) | 10 (6-16) | 0.8 | 1.08 (0.62-1.74) | -0.8 (-0.9 to -0.7) |
| Guyana | 0 (0-1) | 0.1 | 0.1 (0.07-0.14) | 0.6 (0.5 to 0.8) | 19 (13-26) | 0.1 | 4.52 (3.03-6.38) | 0.6 (0.4 to 0.7) |
| Haiti | 5 (3-9) | 0.7 | 0.08 (0.05-0.14) | -1.1 (-1.4 to -0.9) | 249 (145-421) | 0.7 | 3.8 (2.22-6.43) | -1.1 (-1.4 to -0.9) |
| Honduras | 2 (1-2) | 1.4 | 0.03 (0.02-0.05) | -0.6 (-0.9 to -0.3) | 68 (38-111) | 1.3 | 1.32 (0.73-2.15) | -0.7 (-1 to -0.4) |
| Hungary | 51 (36-71) | -0.4 | 1.14 (0.82-1.59) | -3.5 (-4.8 to -2.2) | 2,231 (1,602-3,105) | -0.4 | 50.24 (36.09-69.93) | -3.6 (-4.8 to -2.3) |
| Iceland | 0 (0-0) | 0.3 | 0.09 (0.07-0.11) | 0 (-0.1 to 0.2) | 6 (5-8) | 0.3 | 3.97 (3.04-5.17) | 0.1 (0 to 0.2) |
| India | 4,434 (3,269-5,624) | 1.6 | 0.58 (0.43-0.74) | 1.3 (1.2 to 1.5) | 205,741 (152,006-261,339) | 1.6 | 27.09 (20.01-34.41) | 1.4 (1.3 to 1.5) |
| Indonesia | 58 (34-116) | 0.8 | 0.04 (0.02-0.08) | 0.6 (0.5 to 0.7) | 2,593 (1,503-5,190) | 0.8 | 1.82 (1.05-3.63) | 0.5 (0.5 to 0.6) |

| | | | | | | | | |
|---|---|---|---|---|---|---|---|---|
| Iran | 6 (4-7) | 1.7 | 0.01 (0.01-0.02) | 1.3 (1.1 to 1.4) | 269 (208-328) | 1.7 | 0.57 (0.44-0.69) | 1.3 (1.2 to 1.5) |
| Iraq | 7 (4-10) | 1.4 | 0.03 (0.02-0.04) | -0.6 (-0.7 to -0.5) | 305 (187-471) | 1.4 | 1.32 (0.81-2.04) | -0.6 (-0.7 to -0.5) |
| Ireland | 3 (2-4) | 0.8 | 0.12 (0.09-0.16) | 0.5 (0.2 to 0.9) | 132 (100-174) | 0.8 | 5.68 (4.29-7.44) | 0.7 (0.3 to 1) |
| Israel | 1 (1-2) | 1.3 | 0.03 (0.02-0.04) | 0.4 (0 to 0.7) | 69 (51-86) | 1.3 | 1.57 (1.17-1.97) | 0.4 (0.1 to 0.7) |
| Italy | 41 (35-48) | -0.5 | 0.16 (0.14-0.19) | -2.1 (-2.3 to -2) | 1,877 (1,604-2,160) | -0.5 | 7.4 (6.32-8.52) | -2.1 (-2.3 to -2) |
| Jamaica | 1 (1-1) | 0.7 | 0.07 (0.05-0.1) | 1 (0.5 to 1.5) | 48 (32-69) | 0.8 | 3.12 (2.1-4.49) | 1 (0.5 to 1.5) |
| Japan | 49 (43-56) | 0 | 0.1 (0.08-0.11) | 0.2 (-0.2 to 0.6) | 2,258 (1,960-2,557) | 0.1 | 4.36 (3.79-4.94) | 0.3 (0 to 0.7) |
| Jordan | 1 (1-2) | 3.4 | 0.02 (0.02-0.03) | 1 (0.9 to 1.2) | 66 (44-95) | 3.4 | 1.04 (0.69-1.5) | 1 (0.9 to 1.2) |
| Kazakhstan | 14 (11-19) | -0.2 | 0.15 (0.12-0.2) | -2.8 (-3.4 to -2.1) | 664 (498-870) | -0.3 | 7.15 (5.36-9.37) | -2.8 (-3.5 to -2.2) |
| Kenya | 24 (16-33) | 3.1 | 0.09 (0.06-0.13) | 2.1 (1.8 to 2.4) | 1,085 (737-1,510) | 3.1 | 4.17 (2.83-5.8) | 2.1 (1.8 to 2.3) |
| Kiribati | 0 (0-0) | 1.2 | 0.14 (0.08-0.22) | 0.7 (0.6 to 0.9) | 4 (3-7) | 1.2 | 7.13 (4.16-11.4) | 0.8 (0.6 to 0.9) |
| Kuwait | 0 (0-1) | 0.7 | 0.02 (0.01-0.02) | -1.5 (-2 to -1) | 22 (14-32) | 0.7 | 0.75 (0.49-1.09) | -1.5 (-2 to -1) |
| Kyrgyzstan | 3 (2-3) | 0.6 | 0.08 (0.06-0.1) | -0.1 (-0.6 to 0.5) | 123 (92-163) | 0.6 | 3.67 (2.74-4.85) | -0.1 (-0.6 to 0.4) |
| Laos | 2 (1-3) | 0.4 | 0.05 (0.03-0.08) | -1.6 (-2 to -1.3) | 100 (60-155) | 0.4 | 2.56 (1.54-3.97) | -1.6 (-1.9 to -1.2) |
| Latvia | 4 (3-5) | -0.3 | 0.46 (0.32-0.66) | 0 (-0.3 to 0.4) | 169 (120-241) | -0.3 | 20.68 (14.6-29.44) | 0.1 (-0.3 to 0.4) |
| Lebanon | 1 (1-2) | 0.7 | 0.05 (0.03-0.07) | 0 (-0.4 to 0.3) | 57 (38-83) | 0.7 | 2.15 (1.43-3.12) | 0 (-0.3 to 0.3) |
| Lesotho | 1 (1-2) | 1.8 | 0.11 (0.06-0.18) | 2.7 (2.5 to 2.9) | 58 (32-97) | 1.8 | 5.09 (2.79-8.44) | 2.7 (2.5 to 2.9) |
| Liberia | 1 (0-1) | 1.3 | 0.02 (0.01-0.03) | -0.8 (-1.4 to -0.2) | 25 (13-40) | 1.3 | 1 (0.52-1.61) | -0.7 (-1.3 to -0.1) |
| Libya | 1 (1-1) | 2.1 | 0.02 (0.01-0.03) | 1.7 (1.3 to 2.1) | 37 (23-58) | 2.1 | 0.9 (0.55-1.42) | 1.7 (1.3 to 2.1) |
| Lithuania | 8 (5-11) | -0.1 | 0.64 (0.44-0.9) | 0.8 (0.5 to 1.1) | 344 (240-479) | -0.1 | 28.42 (19.83-39.55) | 0.8 (0.5 to 1.1) |
| Luxembourg | 1 (1-1) | 0 | 0.33 (0.25-0.42) | -1.6 (-1.8 to -1.5) | 46 (35-59) | 0.0 | 15.03 (11.36-19.17) | -1.6 (-1.7 to -1.4) |
| Madagascar | 7 (4-11) | 0.7 | 0.05 (0.03-0.09) | -1.4 (-1.5 to -1.3) | 310 (169-517) | 0.7 | 2.35 (1.28-3.91) | -1.5 (-1.6 to -1.3) |
| Malawi | 1 (1-2) | 2.3 | 0.02 (0.01-0.03) | 1.5 (1.5 to 1.6) | 69 (39-111) | 2.3 | 0.77 (0.44-1.24) | 1.6 (1.5 to 1.7) |
| Malaysia | 12 (8-18) | 0.9 | 0.07 (0.04-0.1) | -0.7 (-1.1 to -0.3) | 550 (356-803) | 0.9 | 3.15 (2.04-4.6) | -0.8 (-1.2 to -0.4) |
| Maldives | 0 (0-0) | 4.4 | 0.06 (0.04-0.08) | 1.2 (0.8 to 1.6) | 8 (5-12) | 4.5 | 2.62 (1.69-3.72) | 1.3 (0.9 to 1.7) |
| Mali | 1 (1-2) | 2 | 0.01 (0.01-0.02) | 0.4 (0.2 to 0.6) | 61 (36-103) | 2.0 | 0.64 (0.37-1.06) | 0.4 (0.3 to 0.6) |
| Malta | 0 (0-0) | 0.3 | 0.15 (0.11-0.19) | -0.7 (-1.1 to -0.2) | 13 (10-17) | 0.3 | 6.77 (5.26-8.73) | -0.5 (-1 to -0.1) |
| Marshall Islands | 0 (0-0) | 1.4 | 0.05 (0.03-0.09) | 1.3 (1.2 to 1.5) | 1 (0-1) | 1.4 | 2.65 (1.55-4.44) | 1.3 (1.2 to 1.5) |
| Mauritania | 0 (0-0) | 0.4 | 0.01 (0-0.02) | -1.5 (-1.6 to -1.5) | 8 (4-20) | 0.4 | 0.43 (0.19-1.03) | -1.5 (-1.6 to -1.4) |
| Mauritius | 1 (0-1) | 1.6 | 0.09 (0.07-0.13) | 4 (3.3 to 4.7) | 28 (20-39) | 1.6 | 4.3 (3.04-6.03) | 3.9 (3.3 to 4.6) |
| Mexico | 15 (11-19) | 0.6 | 0.02 (0.02-0.03) | 0 (-0.2 to 0.2) | 689 (521-869) | 0.6 | 1.04 (0.79-1.31) | 0 (-0.2 to 0.2) |
| Micronesia (Federated States of) | 0 (0-0) | 0.1 | 0.05 (0.01-0.1) | -0.2 (-0.3 to -0.1) | 1 (0-3) | 0.1 | 2.5 (0.73-4.71) | -0.2 (-0.3 to -0.2) |

| | | | | | | | | |
|---|---|---|---|---|---|---|---|---|
| Monaco | 0 (0-0) | 0.4 | 0.14 (0.08-0.22) | 1.5 (1.2 to 1.9) | 1 (1-1) | 0.4 | 6.58 (3.73-10.1) | 1.6 (1.2 to 1.9) |
| Mongolia | 1 (1-1) | 3 | 0.06 (0.04-0.08) | 2.8 (2.7 to 2.9) | 49 (33-69) | 3.0 | 2.69 (1.83-3.84) | 2.8 (2.7 to 2.9) |
| Montenegro | 1 (0-1) | 0 | 0.24 (0.16-0.33) | 0.1 (-0.3 to 0.4) | 32 (22-44) | 0.0 | 10.66 (7.35-14.71) | 0 (-0.3 to 0.3) |
| Morocco | 8 (5-14) | 0.7 | 0.04 (0.02-0.07) | 0.3 (0.2 to 0.5) | 361 (204-623) | 0.6 | 1.88 (1.06-3.25) | 0.3 (0.1 to 0.4) |
| Mozambique | 4 (3-7) | 3.3 | 0.03 (0.02-0.05) | 2.7 (2.4 to 2.9) | 204 (116-331) | 3.3 | 1.53 (0.87-2.48) | 2.7 (2.4 to 3) |
| Myanmar | 13 (8-20) | 0.4 | 0.04 (0.03-0.07) | 0.1 (0 to 0.2) | 576 (347-906) | 0.5 | 1.99 (1.2-3.14) | 0.1 (0 to 0.2) |
| Namibia | 3 (2-4) | 2.4 | 0.22 (0.13-0.33) | 2 (1.8 to 2.3) | 125 (73-192) | 2.4 | 9.95 (5.85-15.29) | 2.1 (1.8 to 2.3) |
| Nauru | 0 (0-0) | 0 | 0.08 (0.04-0.12) | -0.5 (-0.6 to -0.4) | 0 (0-0) | 0.0 | 3.74 (2-5.88) | -0.5 (-0.6 to -0.3) |
| Nepal | 43 (24-68) | 1.9 | 0.26 (0.15-0.42) | 2.1 (1.9 to 2.2) | 1,937 (1,096-3,057) | 2.0 | 11.97 (6.77-18.89) | 2.1 (1.9 to 2.3) |
| Netherlands | 9 (6-11) | -0.2 | 0.12 (0.09-0.15) | -1.4 (-2.1 to -0.7) | 404 (303-516) | -0.2 | 5.38 (4.04-6.87) | -1.3 (-2 to -0.7) |
| New Zealand | 2 (2-2) | 0.5 | 0.1 (0.08-0.12) | 1 (0.5 to 1.6) | 90 (70-112) | 0.5 | 4.52 (3.53-5.64) | 1 (0.5 to 1.5) |
| Nicaragua | 1 (0-1) | 0.8 | 0.02 (0.01-0.03) | -0.5 (-0.7 to -0.3) | 31 (21-45) | 0.8 | 0.89 (0.61-1.27) | -0.5 (-0.7 to -0.4) |
| Niger | 0 (0-1) | 1.3 | 0 (0-0.01) | -0.5 (-0.7 to -0.4) | 22 (11-37) | 1.4 | 0.23 (0.12-0.39) | -0.4 (-0.6 to -0.3) |
| Nigeria | 11 (7-17) | 1.5 | 0.01 (0.01-0.02) | 0.4 (0.2 to 0.6) | 535 (345-811) | 1.6 | 0.53 (0.34-0.8) | 0.5 (0.3 to 0.7) |
| Niue | 0 (0-0) | -0.4 | 0.05 (0.02-0.08) | -0.9 (-1 to -0.8) | 0 (0-0) | -0.4 | 2.26 (1.1-3.86) | -0.9 (-1 to -0.8) |
| Macedonia | 3 (2-5) | 0.3 | 0.3 (0.21-0.42) | 0.4 (0.1 to 0.6) | 148 (102-204) | 0.2 | 13.61 (9.4-18.82) | 0.3 (0 to 0.6) |
| Northern Mariana Islands | 0 (0-0) | -0.3 | 0.17 (0.09-0.27) | 0.9 (0.4 to 1.3) | 2 (1-3) | -0.3 | 8.02 (4.26-12.76) | 0.6 (0.1 to 1) |
| Norway | 2 (2-2) | -0.1 | 0.08 (0.06-0.09) | -1.2 (-1.5 to -0.9) | 91 (75-108) | -0.1 | 3.66 (2.99-4.34) | -1 (-1.3 to -0.7) |
| Oman | 0 (0-1) | 1 | 0.01 (0.01-0.03) | -2.3 (-2.7 to -2) | 21 (11-37) | 1.0 | 0.67 (0.35-1.18) | -2.3 (-2.6 to -1.9) |
| Pakistan | 349 (223-509) | 1.7 | 0.31 (0.2-0.46) | 0.8 (0.5 to 1.1) | 15,753 (10,150-23,007) | 1.8 | 14.09 (9.08-20.58) | 0.8 (0.5 to 1.1) |
| Palau | 0 (0-0) | 0.6 | 0.05 (0.03-0.08) | 1.3 (1.2 to 1.4) | 0 (0-0) | 0.5 | 2.49 (1.39-3.91) | 1.3 (1.2 to 1.4) |
| Palestine | 0 (0-0) | 2.4 | 0.01 (0.01-0.01) | 0.4 (0.2 to 0.6) | 10 (8-14) | 2.4 | 0.41 (0.3-0.56) | 0.4 (0.2 to 0.6) |
| Panama | 1 (1-2) | 1 | 0.06 (0.04-0.09) | 1 (0.5 to 1.4) | 62 (41-90) | 1.0 | 2.95 (1.97-4.29) | 0.9 (0.5 to 1.4) |
| Papua New Guinea | 1 (1-2) | 1.5 | 0.02 (0.01-0.04) | 0.1 (-0.1 to 0.3) | 61 (36-101) | 1.5 | 1.2 (0.7-1.98) | 0.1 (-0.1 to 0.3) |
| Paraguay | 5 (3-7) | 1.4 | 0.14 (0.09-0.2) | 0.1 (-0.2 to 0.3) | 234 (155-343) | 1.4 | 6.29 (4.18-9.22) | 0 (-0.2 to 0.3) |
| Peru | 5 (3-7) | 0.1 | 0.03 (0.02-0.04) | -1.6 (-2.2 to -1.1) | 217 (138-327) | 0.1 | 1.22 (0.77-1.84) | -1.7 (-2.2 to -1.1) |
| Philippines | 33 (24-43) | 0.5 | 0.06 (0.04-0.07) | -1.5 (-1.8 to -1.2) | 1,538 (1,155-2,011) | 0.5 | 2.63 (1.98-3.44) | -1.5 (-1.8 to -1.2) |
| Poland | 72 (55-92) | 0 | 0.39 (0.3-0.5) | -0.8 (-1.1 to -0.4) | 3,224 (2,499-4,116) | 0.0 | 17.71 (13.73-22.62) | -0.8 (-1.1 to -0.4) |
| Portugal | 33 (26-41) | 1.2 | 0.7 (0.55-0.87) | 3 (2.3 to 3.8) | 1,484 (1,161-1,832) | 1.2 | 31.63 (24.75-39.05) | 3 (2.2 to 3.8) |
| Puerto Rico | 2 (1-3) | -0.6 | 0.11 (0.07-0.16) | -3.1 (-3.5 to -2.8) | 79 (52-116) | -0.6 | 4.96 (3.27-7.31) | -3.1 (-3.5 to -2.8) |
| Qatar | 0 (0-1) | 6 | 0.02 (0.01-0.03) | -0.5 (-0.9 to -0.1) | 16 (10-25) | 6.0 | 0.73 (0.44-1.17) | -0.5 (-0.9 to -0.1) |
| South Korea | 19 (14-26) | 0.7 | 0.07 (0.05-0.1) | 2.1 (1.4 to 2.9) | 884 (643-1,179) | 0.7 | 3.39 (2.47-4.53) | 2.1 (1.4 to 2.8) |

| | | | | | | | | |
|---|---|---|---|---|---|---|---|---|
| Moldova | 13 (10-17) | 0.1 | 0.71 (0.55-0.92) | 0.4 (0.1 to 0.8) | 591 (456-757) | 0.1 | 32.14 (24.82-41.2) | 0.4 (0 to 0.7) |
| Romania | 101 (74-132) | 0.8 | 1.16 (0.86-1.52) | 2.7 (2.3 to 3.1) | 4,482 (3,338-5,828) | 0.8 | 51.77 (38.55-67.31) | 2.6 (2.2 to 3) |
| Russia | 274 (219-350) | 0.2 | 0.4 (0.32-0.51) | -0.4 (-0.9 to 0.2) | 12,690 (10,234-16,070) | 0.2 | 18.53 (14.95-23.47) | -0.3 (-0.8 to 0.2) |
| Rwanda | 7 (4-10) | 0.7 | 0.1 (0.06-0.16) | -1.8 (-2.1 to -1.4) | 303 (177-478) | 0.6 | 4.68 (2.74-7.39) | -1.8 (-2.1 to -1.4) |
| Saint Kitts and Nevis | 0 (0-0) | 0.2 | 0.09 (0.03-0.18) | -1.6 (-1.9 to -1.2) | 1 (0-2) | 0.1 | 4.2 (1.31-7.94) | -1.7 (-2.1 to -1.4) |
| Saint Lucia | 0 (0-0) | 0.6 | 0.21 (0.15-0.28) | 0.6 (0.1 to 1) | 9 (6-12) | 0.5 | 9.36 (6.92-12.35) | 0.5 (0 to 0.9) |
| Saint Vincent and the Grenadines | 0 (0-0) | 0.7 | 0.26 (0.19-0.34) | 1.9 (1.5 to 2.3) | 7 (5-9) | 0.6 | 11.59 (8.61-15.25) | 1.7 (1.4 to 2.1) |
| Samoa | 0 (0-0) | 0.7 | 0.04 (0.02-0.06) | 1 (0.7 to 1.3) | 2 (1-3) | 0.7 | 1.75 (0.97-2.79) | 0.9 (0.6 to 1.2) |
| San Marino | 0 (0-0) | 0.4 | 0.15 (0.08-0.25) | 0.5 (0.4 to 0.7) | 1 (1-2) | 0.4 | 6.74 (3.52-11.16) | 0.6 (0.4 to 0.7) |
| Sao Tome and Principe | 0 (0-0) | 3.1 | 0.02 (0.01-0.03) | 2.2 (1.9 to 2.4) | 1 (1-1) | 3.1 | 0.78 (0.47-1.19) | 2.1 (1.9 to 2.4) |
| Saudi Arabia | 6 (4-10) | 4 | 0.03 (0.02-0.04) | 1.7 (1.6 to 1.9) | 291 (176-469) | 4.0 | 1.2 (0.72-1.93) | 1.8 (1.7 to 1.9) |
| Senegal | 1 (0-1) | 0.8 | 0.01 (0.01-0.02) | -0.7 (-0.9 to -0.5) | 41 (23-66) | 0.8 | 0.56 (0.32-0.9) | -0.6 (-0.8 to -0.4) |
| Serbia | 17 (12-25) | -0.2 | 0.44 (0.29-0.63) | -1 (-1.5 to -0.6) | 775 (523-1,102) | -0.2 | 19.35 (13.07-27.54) | -1.1 (-1.5 to -0.6) |
| Seychelles | 0 (0-1) | 3 | 0.79 (0.56-1.11) | 2.9 (2.4 to 3.3) | 19 (14-27) | 3.0 | 35.7 (25.5-49.62) | 2.8 (2.3 to 3.3) |
| Sierra Leone | 1 (1-1) | 1 | 0.02 (0.01-0.03) | -1.1 (-1.3 to -0.8) | 38 (24-58) | 1.0 | 0.92 (0.57-1.39) | -1 (-1.2 to -0.7) |
| Singapore | 1 (1-2) | 0.1 | 0.04 (0.03-0.06) | -2.2 (-2.7 to -1.7) | 56 (39-78) | 0.1 | 1.83 (1.28-2.53) | -2.2 (-2.7 to -1.7) |
| Slovakia | 29 (19-42) | -0.1 | 1.09 (0.71-1.58) | -1.7 (-2.3 to -1.1) | 1,267 (829-1,829) | -0.1 | 48.15 (31.5-69.52) | -1.7 (-2.3 to -1.1) |
| Slovenia | 6 (4-9) | -0.3 | 0.72 (0.48-1.05) | -1.6 (-2.1 to -1.2) | 286 (194-417) | -0.3 | 31.94 (21.68-46.52) | -1.6 (-2 to -1.2) |
| Solomon Islands | 0 (0-0) | 1.9 | 0.05 (0.02-0.11) | 1.3 (1.2 to 1.5) | 8 (4-17) | 1.9 | 2.47 (1.17-5.22) | 1.4 (1.2 to 1.5) |
| Somalia | 2 (1-5) | 1.1 | 0.02 (0.01-0.05) | -2.2 (-2.7 to -1.8) | 91 (39-197) | 1.1 | 0.96 (0.41-2.09) | -2.2 (-2.6 to -1.7) |
| South Africa | 24 (19-30) | 0.1 | 0.08 (0.06-0.1) | -1.9 (-2.1 to -1.6) | 1,137 (902-1,377) | 0.1 | 3.73 (2.96-4.52) | -1.9 (-2.2 to -1.6) |
| South Sudan | 2 (1-3) | 0.3 | 0.04 (0.02-0.07) | -0.6 (-0.8 to -0.3) | 74 (31-140) | 0.3 | 1.76 (0.72-3.31) | -0.6 (-0.8 to -0.3) |
| Spain | 55 (42-69) | -0.3 | 0.27 (0.21-0.33) | -2.9 (-3.3 to -2.4) | 2,479 (1,936-3,101) | -0.3 | 12 (9.37-15.01) | -2.9 (-3.3 to -2.4) |
| Sri Lanka | 18 (11-28) | 0.5 | 0.17 (0.1-0.26) | 0.4 (0 to 0.8) | 825 (517-1,234) | 0.4 | 7.57 (4.74-11.32) | 0.3 (-0.1 to 0.7) |
| Sudan | 2 (1-3) | 0.5 | 0.01 (0.01-0.02) | -1.4 (-1.7 to -1.2) | 94 (49-159) | 0.5 | 0.45 (0.23-0.76) | -1.5 (-1.8 to -1.2) |
| Suriname | 0 (0-0) | 0.7 | 0.09 (0.06-0.13) | 0.8 (0.4 to 1.1) | 12 (8-16) | 0.7 | 4.05 (2.84-5.61) | 0.6 (0.3 to 1) |
| Sweden | 3 (2-4) | -0.1 | 0.07 (0.05-0.08) | -0.3 (-0.5 to -0.1) | 138 (110-171) | -0.1 | 3.09 (2.46-3.82) | -0.2 (-0.4 to 0) |
| Switzerland | 6 (4-7) | 0.4 | 0.15 (0.11-0.19) | -0.2 (-1.1 to 0.7) | 269 (207-344) | 0.5 | 6.72 (5.18-8.62) | -0.1 (-1 to 0.8) |
| Syria | 1 (1-2) | 0.6 | 0.02 (0.01-0.03) | 1.1 (0.8 to 1.4) | 63 (40-93) | 0.6 | 0.85 (0.53-1.25) | 1 (0.7 to 1.2) |
| Taiwan | 136 (99-187) | 3.5 | 1.17 (0.84-1.6) | 5.3 (4.5 to 6.2) | 6,226 (4,480-8,499) | 3.5 | 53.28 (38.34-72.73) | 5.3 (4.5 to 6.1) |
| Tajikistan | 2 (1-3) | 0.7 | 0.04 (0.03-0.06) | -1.2 (-1.7 to -0.7) | 98 (68-135) | 0.7 | 1.97 (1.36-2.71) | -1.4 (-1.9 to -0.8) |

| | | | | | | | | |
|---|---|---|---|---|---|---|---|---|
| Thailand | 58 (38-87) | 0.6 | 0.16 (0.11-0.24) | 0.7 (0.4 to 1) | 2,647 (1,750-3,902) | 0.6 | 7.42 (4.9-10.93) | 0.6 (0.3 to 0.9) |
| Timor-Leste | 0 (0-0) | 0.7 | 0.03 (0.01-0.06) | 0 (-0.5 to 0.5) | 10 (2-19) | 0.7 | 1.48 (0.3-2.87) | 0 (-0.5 to 0.4) |
| Togo | 1 (0-1) | 1.5 | 0.02 (0.01-0.03) | 0 (-0.1 to 0.2) | 39 (23-62) | 1.6 | 1 (0.58-1.57) | 0.1 (-0.1 to 0.2) |
| Tokelau | 0 (0-0) | 0.1 | 0.04 (0.02-0.06) | 0.4 (0.3 to 0.6) | 0 (0-0) | 0.1 | 1.97 (0.92-3.19) | 0.5 (0.3 to 0.6) |
| Tonga | 0 (0-0) | 0.6 | 0.02 (0.01-0.04) | 1.4 (1.2 to 1.7) | 1 (0-1) | 0.6 | 1.19 (0.65-1.96) | 1.5 (1.2 to 1.8) |
| Trinidad and Tobago | 1 (1-1) | 0 | 0.13 (0.09-0.19) | -1.3 (-1.5 to -1) | 42 (27-61) | 0.0 | 5.98 (3.93-8.78) | -1.3 (-1.5 to -1) |
| Tunisia | 4 (2-6) | 1.6 | 0.06 (0.04-0.09) | 1.8 (1.5 to 2) | 173 (108-250) | 1.6 | 2.87 (1.79-4.15) | 1.7 (1.5 to 1.9) |
| Turkey | 16 (11-23) | 0.2 | 0.04 (0.02-0.05) | -1.4 (-1.6 to -1.1) | 754 (531-1,043) | 0.2 | 1.65 (1.16-2.28) | -1.4 (-1.6 to -1.1) |
| Turkmenistan | 5 (4-7) | 1.5 | 0.19 (0.14-0.26) | 1.4 (1.2 to 1.6) | 227 (166-308) | 1.4 | 8.63 (6.31-11.67) | 1.4 (1.2 to 1.5) |
| Tuvalu | 0 (0-0) | 0.4 | 0.05 (0.03-0.08) | -0.1 (-0.1 to 0) | 0 (0-0) | 0.4 | 2.26 (1.24-3.92) | 0 (0 to 0.1) |
| Uganda | 37 (23-57) | 2.8 | 0.2 (0.12-0.3) | 1.3 (1.1 to 1.5) | 1,702 (1,072-2,569) | 2.8 | 9.01 (5.68-13.61) | 1.3 (1.1 to 1.5) |
| Ukraine | 181 (133-244) | 1.2 | 0.87 (0.64-1.17) | 2.9 (2.5 to 3.4) | 8,148 (5,992-10,930) | 1.2 | 39.2 (28.83-52.59) | 2.9 (2.5 to 3.4) |
| United Arab Emirates | 7 (3-13) | 6.5 | 0.09 (0.05-0.18) | -0.1 (-1 to 0.7) | 302 (152-590) | 6.4 | 4.33 (2.18-8.46) | -0.1 (-0.9 to 0.7) |
| UK | 48 (42-52) | 0.4 | 0.16 (0.14-0.17) | 1 (0.8 to 1.2) | 2,263 (2,024-2,479) | 0.5 | 7.42 (6.64-8.13) | 1.1 (1 to 1.3) |
| Tanzania | 29 (16-46) | 1.6 | 0.11 (0.06-0.17) | 0.4 (0.3 to 0.5) | 1,312 (744-2,121) | 1.6 | 4.92 (2.79-7.96) | 0.4 (0.3 to 0.5) |
| USA | 125 (111-137) | -0.2 | 0.08 (0.07-0.09) | -1 (-1.3 to -0.8) | 6,038 (5,373-6,652) | -0.1 | 4.01 (3.57-4.42) | -1 (-1.3 to -0.8) |
| United States Virgin Islands | 0 (0-0) | -0.2 | 0.2 (0.08-0.33) | 0.3 (0 to 0.5) | 4 (1-6) | -0.2 | 8.69 (3.42-14.61) | 0.1 (-0.1 to 0.4) |
| Uruguay | 3 (2-4) | -0.2 | 0.17 (0.13-0.22) | -1.6 (-1.8 to -1.5) | 128 (99-164) | -0.2 | 7.78 (6.03-9.95) | -1.6 (-1.7 to -1.4) |
| Uzbekistan | 21 (15-28) | 3.3 | 0.12 (0.08-0.16) | 2.6 (2.2 to 3.1) | 1,012 (738-1,339) | 3.3 | 5.6 (4.08-7.4) | 2.6 (2.2 to 2.9) |
| Vanuatu | 0 (0-0) | 1.4 | 0.03 (0.02-0.06) | 0.4 (0.1 to 0.6) | 2 (1-4) | 1.4 | 1.61 (0.89-2.79) | 0.4 (0.1 to 0.7) |
| Venezuela | 10 (7-15) | 0.6 | 0.07 (0.05-0.11) | 0.3 (0.1 to 0.5) | 481 (319-692) | 0.6 | 3.34 (2.22-4.81) | 0.2 (0 to 0.5) |
| Vietnam | 271 (165-413) | 4.6 | 0.51 (0.31-0.78) | 4.8 (4.4 to 5.3) | 12,194 (7,526-18,394) | 4.6 | 23 (14.19-34.69) | 4.8 (4.4 to 5.3) |
| Yemen | 3 (1-4) | 1.2 | 0.02 (0.01-0.03) | -1.1 (-1.3 to -0.8) | 117 (65-203) | 1.2 | 0.75 (0.42-1.3) | -1 (-1.3 to -0.8) |
| Zambia | 11 (6-17) | 2.2 | 0.12 (0.07-0.19) | 0.6 (0.4 to 0.8) | 493 (296-791) | 2.2 | 5.46 (3.28-8.76) | 0.6 (0.4 to 0.8) |
| Zimbabwe | 6 (4-9) | 2.2 | 0.08 (0.05-0.12) | 2.3 (1.9 to 2.7) | 264 (159-415) | 2.2 | 3.5 (2.11-5.51) | 2.3 (1.9 to 2.7) |

# Supplementary Figure Legends

**Supplementary Fig. S1.** Age-standardized rate of mortality and DALYs rate for tobacco- and alcohol-attributable (A) early-onset lip and oral cavity cancer (LOC) and (B) early-onset other pharyngeal cancer (OPC) versus Socio-demographic Index (SDI) in 2019. The size of each circle is proportional to the number of deaths or DALYs due to tobacco- and alcohol-attributable (A) early-onset LOC and (B) early-onset OPC in 2019, respectively. The ρ indices and p values were derived from Pearson correlation analysis. DALYs=Disability adjusted life years.

**Supplementary Fig. S2.** Age-specific mortality and DALYs rate of tobacco- and alcohol-attributable (A) early-onset lip and oral cavity cancer (LOC) and (B) early-onset other pharyngeal cancer (OPC) versus Socio-demographic Index (SDI) in 2019. The size of each circle is proportional to the number of deaths or DALYs due to tobacco- and alcohol-attributable (A) early-onset LOC and (B) early-onset OPC in 2019, respectively. The ρ indices and p values were derived from Pearson correlation analysis. DALYs=Disability adjusted life years.

**Supplementary Fig. S3.** Male-to-female ratio in age-standardized mortality and age-specific mortality for tobacco- and alcohol-attributable early-onset lip and oral cavity cancer (LOC) and early-onset other pharyngeal cancer (OPC) in 2019, globally and for 5 Socio-demographic Index (SDI) quintiles and 21 GBD regions.

**Supplementary Fig. S4.** The temporal trend in the age-specific mortality and DALYs rate for early-onset LOC attributable to tobacco and alcohol across age groups (in both sexes combined) by Socio-demographic Index (SDI) quintile, 1990-2019. (A) High SDI quintiles;

(B) High-middle SDI quintiles; (C) Middle SDI quintiles; (D) Low-middle SDI quintiles; (E) Low SDI quintiles. LOC=Lip and oral cavity cancer. OPC=Other pharyngeal cancer. DALYs=Disability adjusted life years.

**Supplementary Fig. S5.** The temporal trend in the age-specific mortality and DALYs rate for early-onset OPC attributable to tobacco and alcohol across age groups (in both sexes combined) by Socio-demographic Index (SDI) quintile, 1990-2019. (A) High SDI quintiles; (B) High-middle SDI quintiles; (C) Middle SDI quintiles; (D) Low-middle SDI quintiles; (E) Low SDI quintiles. LOC=Lip and oral cavity cancer. OPC=Other pharyngeal cancer. DALYs=Disability adjusted life years.

**Supplementary Fig. S6.** The temporal trend in the age-specific mortality and DALYs rate for early-onset LOC attributable to tobacco and alcohol across age groups, by sex and Socio-demographic Index (SDI) quintile, 1990-2019. (A) High SDI quintiles; (B) High-middle SDI quintiles; (C) Middle SDI quintiles; (D) Low-middle SDI quintiles; (E) Low SDI quintiles. LOC=Lip and oral cavity cancer. OPC=Other pharyngeal cancer. DALYs=Disability adjusted life years.

**Supplementary Fig. S7.** The temporal trend in the age-specific mortality and DALYs rate for early-onset OPC attributable to tobacco and alcohol across age groups, by sex and Socio-demographic Index (SDI) quintile, 1990-2019. (A) High SDI quintiles; (B) High-middle SDI quintiles; (C) Middle SDI quintiles; (D) Low-middle SDI quintiles; (E) Low SDI quintiles. LOC=Lip and oral cavity cancer. OPC=Other pharyngeal cancer. DALYs=Disability adjusted life years.

**Supplementary Fig. S8.** Global absolute number and age-specific rate of tobacco- and alcohol-attributable burden for early-onset lip, oral cavity, and pharyngeal cancer by sex, 1990-2019. (A) The number of death cases and age-specific mortality rate for early-onset LOC

attributable to tobacco and alcohol; (B) The number of death cases and age-specific mortality rate for early-onset OPC attributable to tobacco and alcohol; (A) The number of DALYs and age-specific DALYs rate for early-onset LOC attributable to risks; (A) The number of DALYs and age-specific DALYs rate for early-onset OPC attributable to tobacco and alcohol. LOC=Lip and oral cavity cancer. OPC=Other pharyngeal cancer. DALYs=Disability adjusted life years. Bars indicate absolute numbers, and lines indicate rates, with shaded areas representing respective 95% uncertainty interval (UI).

**Supplementary Fig. S9.** The estimated annual percentage change (EAPC) of the ASMR and ASDR for tobacco- and alcohol-attributable early-onset LOC among males and females and both sexes combined from 1990 to 2019, by country. ASMR=Age-standardized mortality rate; ASDR=Age-standardized disability adjusted life years rate; LOC=Lip and oral cavity cancer.

**Supplementary Fig. S10.** The estimated annual percentage change (EAPC) of the ASMR and ASDR for tobacco- and alcohol-attributable early-onset OPC among males and females and both sexes combined from 1990 to 2019, by country. ASMR=Age-standardized mortality rate; ASDR=Age-standardized disability adjusted life years rate; OPC=Other pharyngeal cancer.

**Supplementary Fig. S11.** Proportion of deaths and disability adjusted life years (DALYs) burden attributable to tobacco and alcohol in both sexes combined globally and in 21 GBD regions, 2019, for (A) early-onset lip and oral cavity cancer (LOC) and (B) early-onset other pharyngeal cancer (OPC).

**Supplementary Fig. S12.** Time trends of risk-specific population attributable fraction (PAF) of disability adjusted life years (DALYs) burden for (A) early-onset LOC and (B) early-onset OPC in both sexes combined, by Socio-demographic Index (SDI) quintile, 1990-

2019. LOC=Lip and oral cavity cancer. OPC=Other pharyngeal cancer. DALYs=Disability adjusted life years.

**Supplementary Fig. S13.** Relationship between population attributable fraction (PAF) of disability adjusted life years (DALYs) burden for (A) early-onset LOC and (B) early-onset OPC and Socio-demographic Index (SDI) in 2019. The size of each circle is proportional to the number of early-onset LOC DALYs attributable to corresponding risk factors in 2019. The ρ indices and p values were derived from Pearson correlation analysis. LOC=Lip and oral cavity cancer, OPC=Other pharyngeal cancer.

**Supplementary Fig. S14.** Change from 1990 to 2019 in population attributable fraction for alcohol use, smoking, and chewing tobacco in relation to early-onset (A) LOC and (B) OPC in both sexes combined, by 21 GBD region. LOC=Lip and oral cavity cancer. OPC=Other pharyngeal cancer.

**Supplementary Fig. S15.** Tobacco- and alcohol-attributable proportion of both deaths (upper) and DALYs (bottom) burden for both sexes globally and in 21 GBD regions, 2019, for (A) early-onset lip and oral cavity cancer (LOC) and (B) early-onset other pharyngeal cancer (OPC). DALYs=Disability adjusted life years.

**Supplementary Fig. S16.** Global distribution of age-specific summary exposure value (SEV) rates of risk factors for individuals aged 15-49 years in both sexes combined in 2019, with annualized rate of change (ARC) from 1990 to 2019. (A) Smoking; (B) Chewing tobacco; (C) Alcohol use.

**Supplementary Fig. S17.** Annualized rate of change in alcohol use summary exposure values (SEV) in people aged 15-49 years, for

both sexes, from 1990 to 2019 versus the number of DALYs for (A) early-onset LOC and (B) early-onset OPC attributable to alcohol use in 2019, by country. Countries with different levels of socio-economic development are indicated by different colors. LOC=Lip and oral cavity cancer; OPC=Other pharyngeal cancer; DALYs=Disability adjusted life years.

**Supplementary Fig. S18.** Annualized rate of change in smoking summary exposure values (SEV) in people aged 15-49 years, for both sexes, from 1990 to 2019 versus the number of DALYs for (A) early-onset LOC and (B) early-onset OPC attributable to smoking in 2019, by country. Countries with different levels of socio-economic development are indicated by different colors. LOC=Lip and oral cavity cancer; OPC=Other pharyngeal cancer; DALYs=Disability adjusted life years.

**Supplementary Fig. S19.** Annualized rate of change in chewing tobacco summary exposure values (SEV) in people aged 15-49 years, for both sexes, from 1990 to 2019 versus the number of early-onset LOC DALYs attributable to chewing tobacco in 2019, by country. Countries with different levels of socio-economic development are indicated by different colors. LOC=Lip and oral cavity cancer; OPC=Other pharyngeal cancer; DALYs=Disability adjusted life years.

**Supplementary Fig. S20.** Trends of age-standardized mortality rate (ASMR) in the early-onset lip and oral cavity cancer (LOC) attributable to (A) smoking, (B) chewing tobacco, and (C) alcohol use, for males (upper) and females (bottom): observed rate (1990–2019) and predicted rates (2020–2040). The blue region in shows the upper and lower limits of the 95% uncertainty interval (UI).

**Supplementary Fig. S21.** Trends of age-standardized mortality rate (ASMR) in the early-onset other pharyngeal cancer (OPC) attributable to (A) smoking and (B) alcohol use, for males (upper) and females (bottom): observed rate (1990–2019) and predicted rates

(2020–2040). The blue region in shows the upper and lower limits of the 95% uncertainty interval (UI).

**Supplementary Fig. S22.** Time trends of age-standardized mortality rate (ASMR) for risk-related early-onset (A) LOC) and (B) OPC in population aged 15-34 years or aged 35-49 years, by sex, 1990-2019. LOC=Lip and oral cavity cancer. OPC=Other pharyngeal cancer.

**Supplementary Fig. S23.** Time trends of age-standardized mortality rate (ASMR) for early-onset lip and oral cavity cancer (LOC) attributable to (A) smoking, (B) chewing tobacco, and (C) alcohol use, by sex and Socio-demographic Index (SDI) quintile, 1990-2019.

**Supplementary Fig. S24.** Time trends of age-standardized DALYs rate (ASDR) for early-onset lip and oral cavity cancer (LOC) attributable to (A) smoking, (B) chewing tobacco, and (C) alcohol use, by sex and Socio-demographic Index (SDI) quintile, 1990-2019.

**Supplementary Fig. S25.** Time trends of age-standardized mortality rate (ASMR) for early-onset other pharyngeal cancer (OPC) attributable to (A) smoking and (B) alcohol use, by sex and Socio-demographic Index (SDI) quintile, 1990-2019.

**Supplementary Fig. S26.** Time trends of age-standardized DALYs rate (ASDR) for early-onset other pharyngeal cancer (OPC) attributable to (A) smoking and (B) alcohol use, by sex and Socio-demographic Index (SDI) quintile, 1990-2019.

**Supplementary Fig. S27.** Time trends of age-specific mortality and DALYs rate for (A) early-onset lip and oral cavity cancer (LOC) and (B) early-onset other pharyngeal cancer (OPC) attributable to tobacco and alcohol in both sexes combined, by Socio-demographic Index (SDI) quintile, 1990-2019. DALYs=Disability adjusted life years.

**Supplementary Fig. S28.** Time trends of age-specific mortality and DALYs rate for tobacco- and alcohol-attributable (A) early-onset

lip and oral cavity cancer (LOC) and (B) early-onset other pharyngeal cancer (OPC) in both sexes combined, by risk factors, 1990-2019. DALYs=Disability adjusted life years.

**Supplementary Fig. S29.** The temporal trend in the age-specific DALYs rate for early-onset lip and oral cavity cancer (LOC) attributable to tobacco and alcohol across age groups in males (left) and females (right), by Socio-demographic Index (SDI) quintile, 1990-2019. (A) High SDI quintiles; (B) High-middle SDI quintiles; (C) Middle SDI quintiles; (D) Low-middle SDI quintiles; (E) Low SDI quintiles. LOC=Lip and oral cavity cancer. OPC=Other pharyngeal cancer. DALYs=Disability adjusted life years.

**Supplementary Fig. S30.** The temporal trend in the age-specific DALYs rate for early-onset other pharyngeal cancer (OPC) attributable to tobacco and alcohol across age groups in males (left) and females (right), by Socio-demographic Index (SDI) quintile, 1990-2019. (A) High SDI quintiles; (B) High-middle SDI quintiles; (C) Middle SDI quintiles; (D) Low-middle SDI quintiles; (E) Low SDI quintiles. LOC=Lip and oral cavity cancer. OPC=Other pharyngeal cancer. DALYs=Disability adjusted life years.

**Supplementary Fig. S21.** Time trends of risk- and age-specific DALYs rate for (A) early-onset lip and oral cavity cancer (LOC) and (B) early-onset other pharyngeal cancer (OPC) attributable to tobacco and alcohol in both sexes combined, by Socio-demographic Index (SDI) quintile, 1990-2019. DALYs=Disability adjusted life years.

**Supplementary Fig. S32.** Global distribution and trends of death burden for tobacco- and alcohol-attributable early-onset lip and oral cavity cancer (LOC) and other pharyngeal cancer (OPC) in both sexes combined. (A) The percentage change in the number of tobacco- and alcohol-attributable early-onset LOC deaths from 1990 to 2019; (B) The percentage change in the number of tobacco- and alcohol-

attributable early-onset OPC deaths from 1990 to 2019; (C) Age-specific mortality rate of tobacco- and alcohol-attributable early-onset LOC in 2019; (D) Age-specific mortality rate of tobacco- and alcohol-attributable early-onset OPC in 2019; (E) The estimated annual percentage change of the age-specific mortality rate for tobacco- and alcohol-attributable early-onset LOC from 1990 to 2019; (F) The estimated annual percentage change of the age-specific mortality rate for tobacco- and alcohol-attributable early-onset OPC from 1990 to 2019.

**Supplementary Fig. S33.** Global distribution and trends of disability adjusted life years (DALYs) for tobacco- and alcohol-attributable early-onset lip and oral cavity cancer (LOC) and other pharyngeal cancer (OPC) in both sexes combined. (A) The percentage change in the number of tobacco- and alcohol-attributable early-onset LOC DALYs from 1990 to 2019; (B) The percentage change in the number of tobacco- and alcohol-attributable early-onset OPC DALYs from 1990 to 2019; (C) Age-specific DALYs rate of tobacco- and alcohol-attributable early-onset LOC in 2019; (D) Age-specific DALYs rate of tobacco- and alcohol-attributable early-onset OPC in 2019; (E) The estimated annual percentage change of the age-specific DALYs rate for tobacco- and alcohol-attributable early-onset LOC from 1990 to 2019; (F) The estimated annual percentage change of the age-specific DALYs rate for tobacco- and alcohol-attributable early-onset OPC from 1990 to 2019.

Supplementary Fig. S1

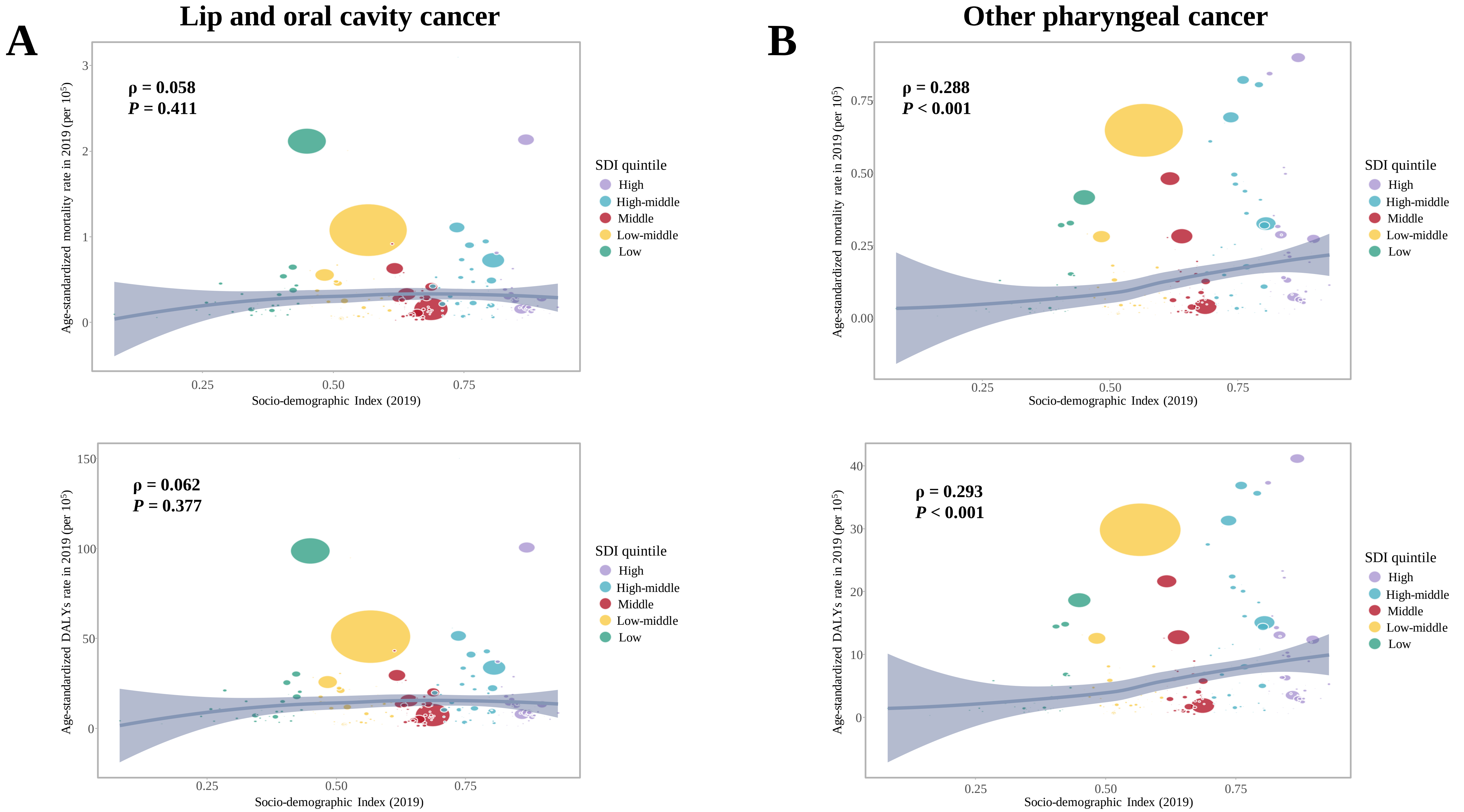

Supplementary Fig. S2

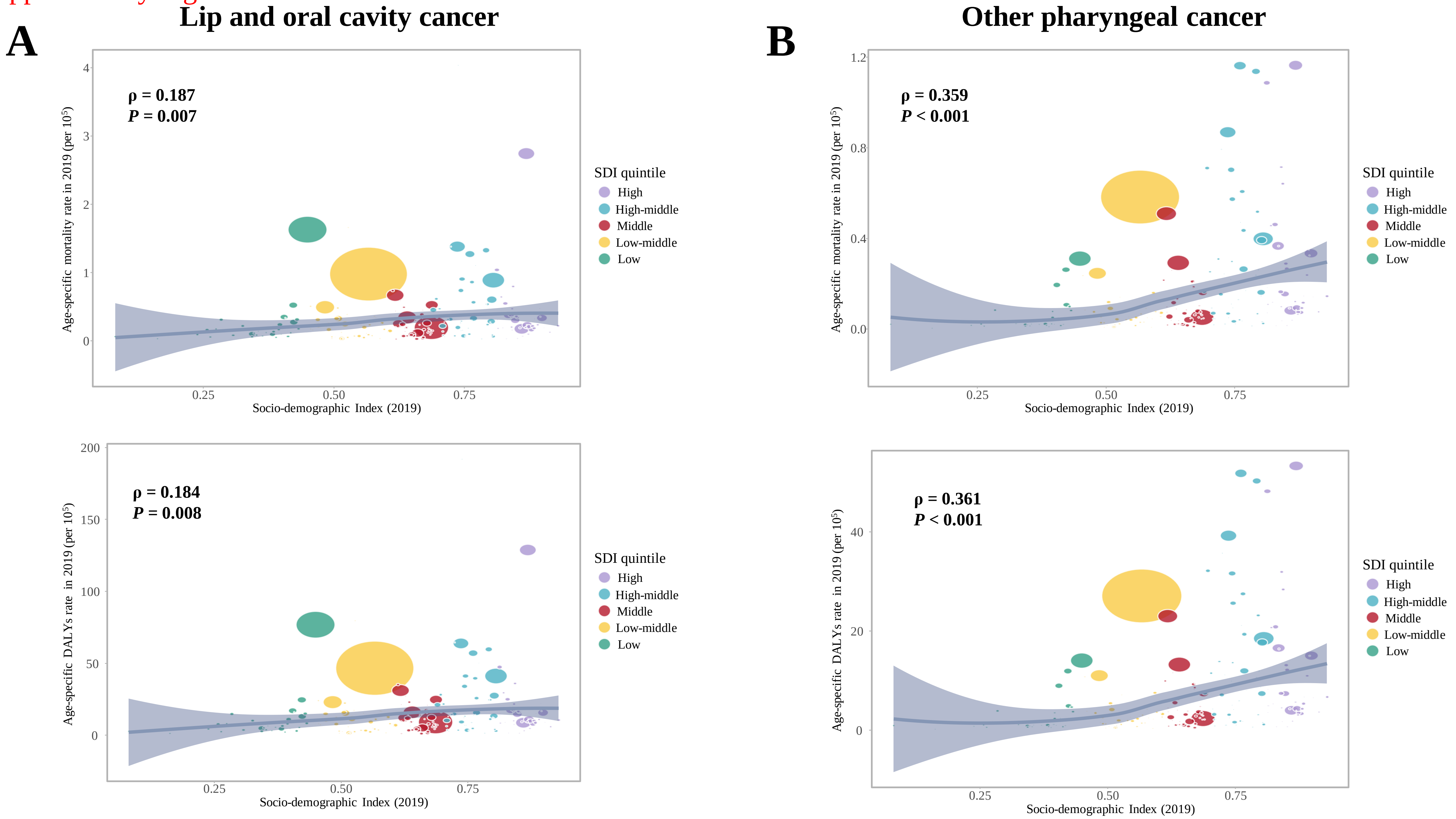

Supplementary Fig. S3

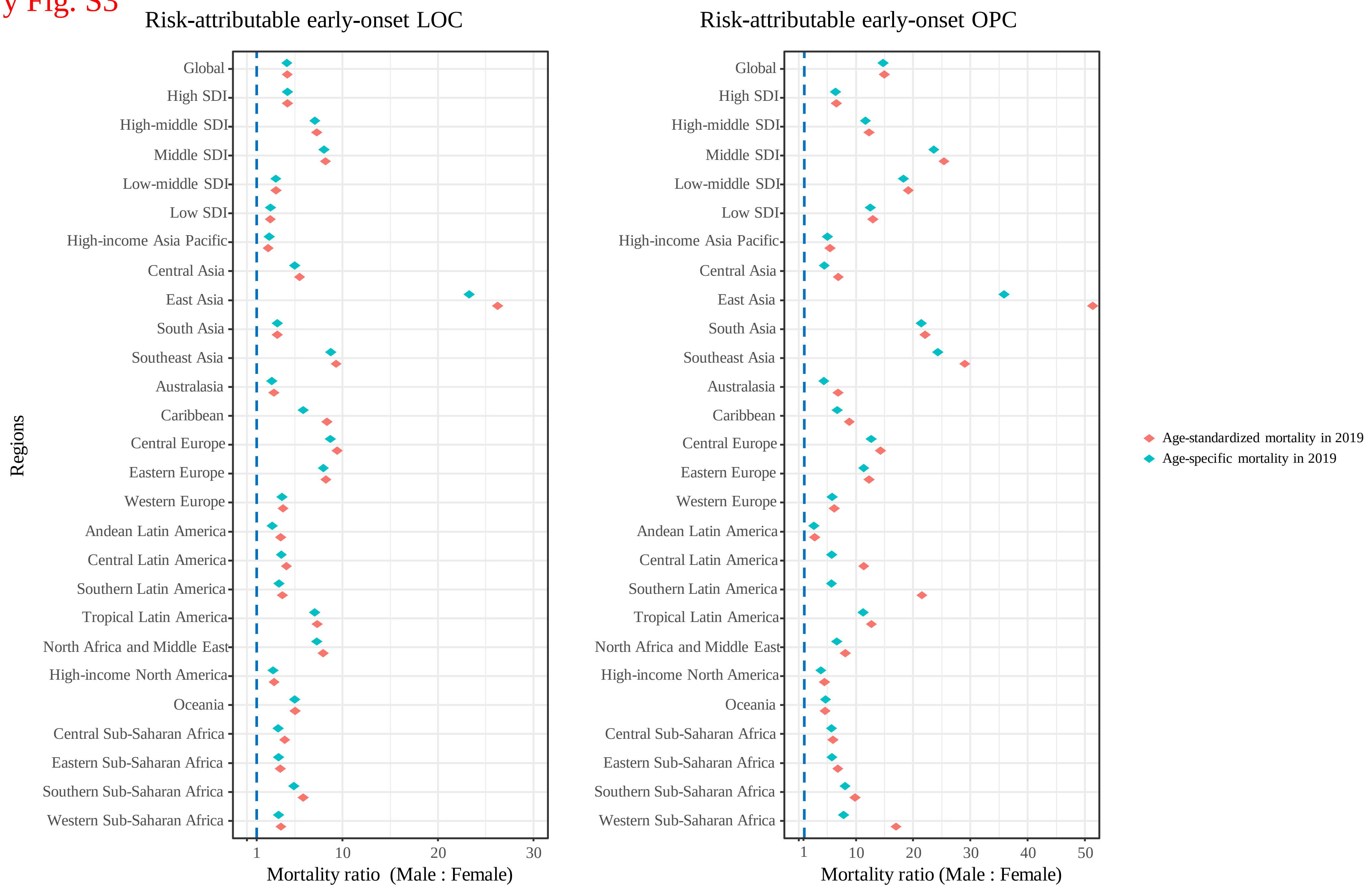

Supplementary Fig. S4

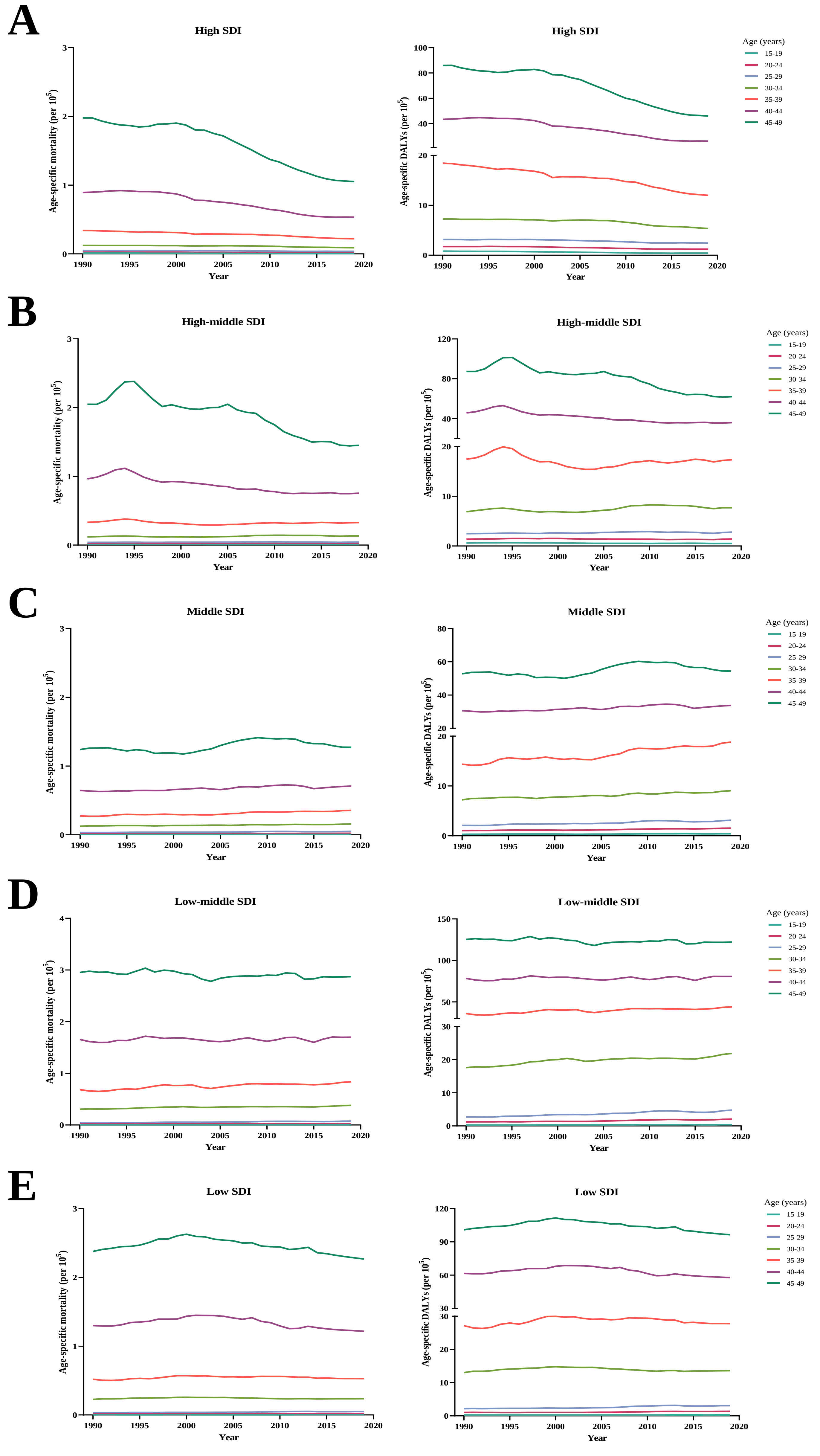

Supplementary Fig. S5

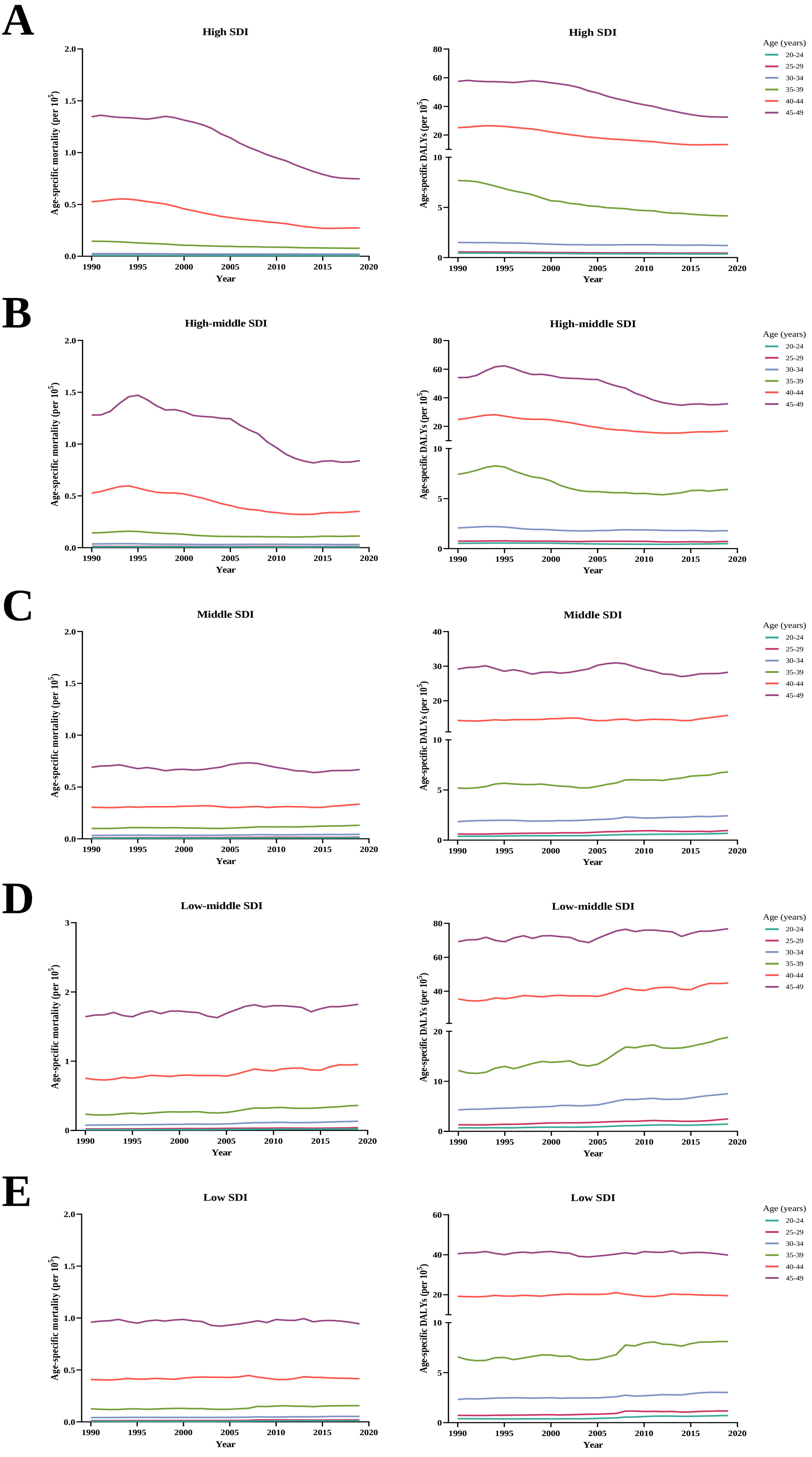

Supplementary Fig. S6

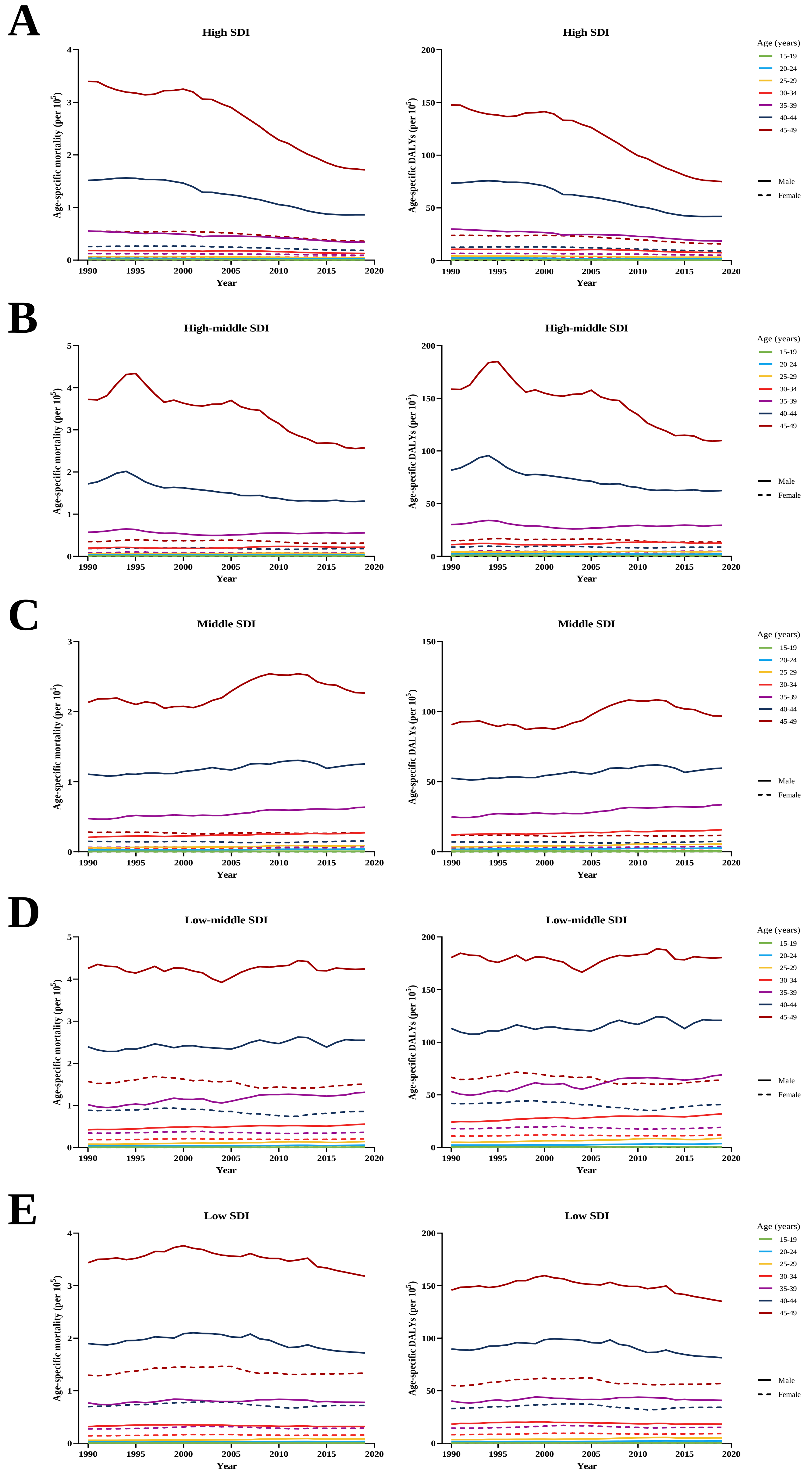

Supplementary Fig. S7

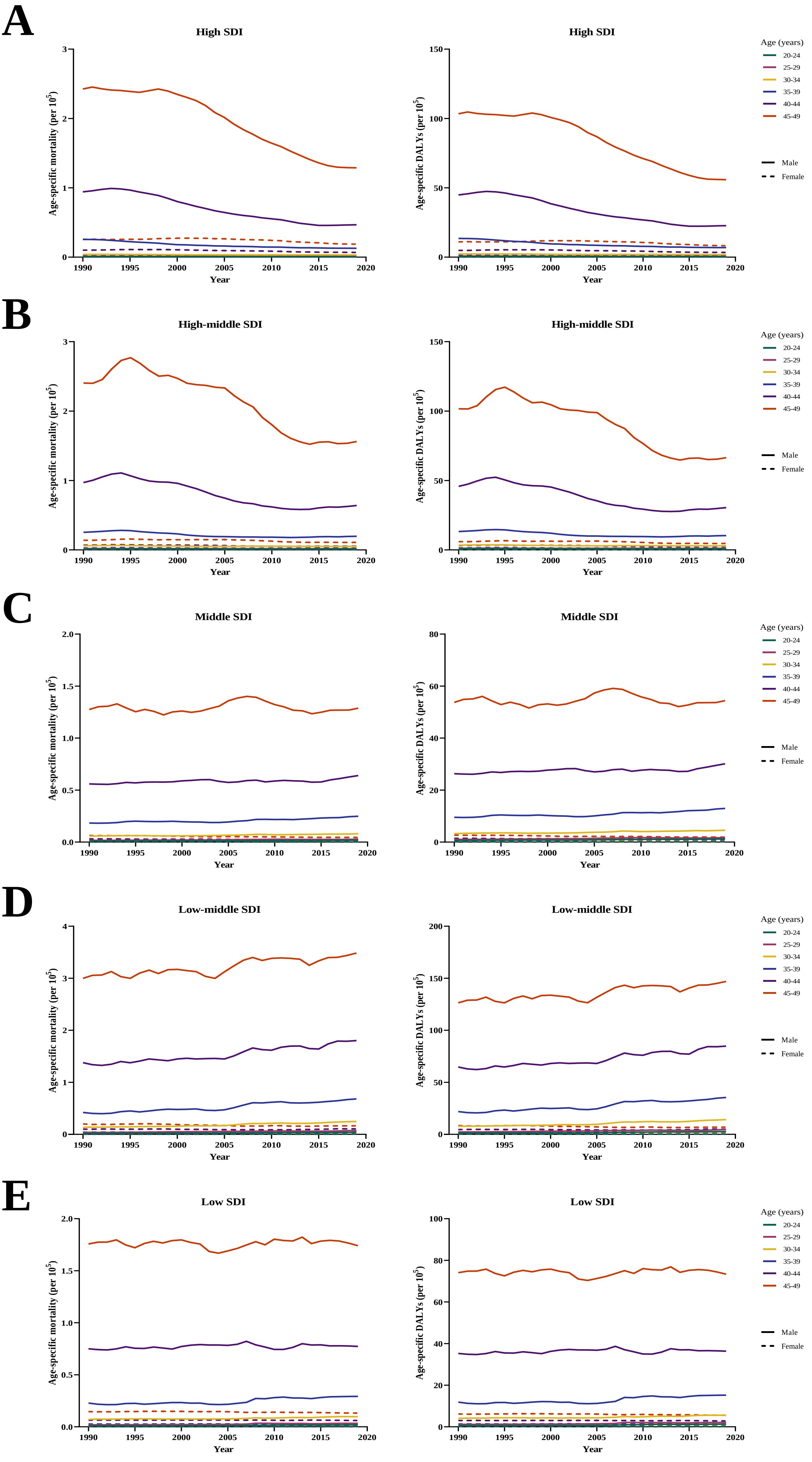

Supplementary Fig. S8

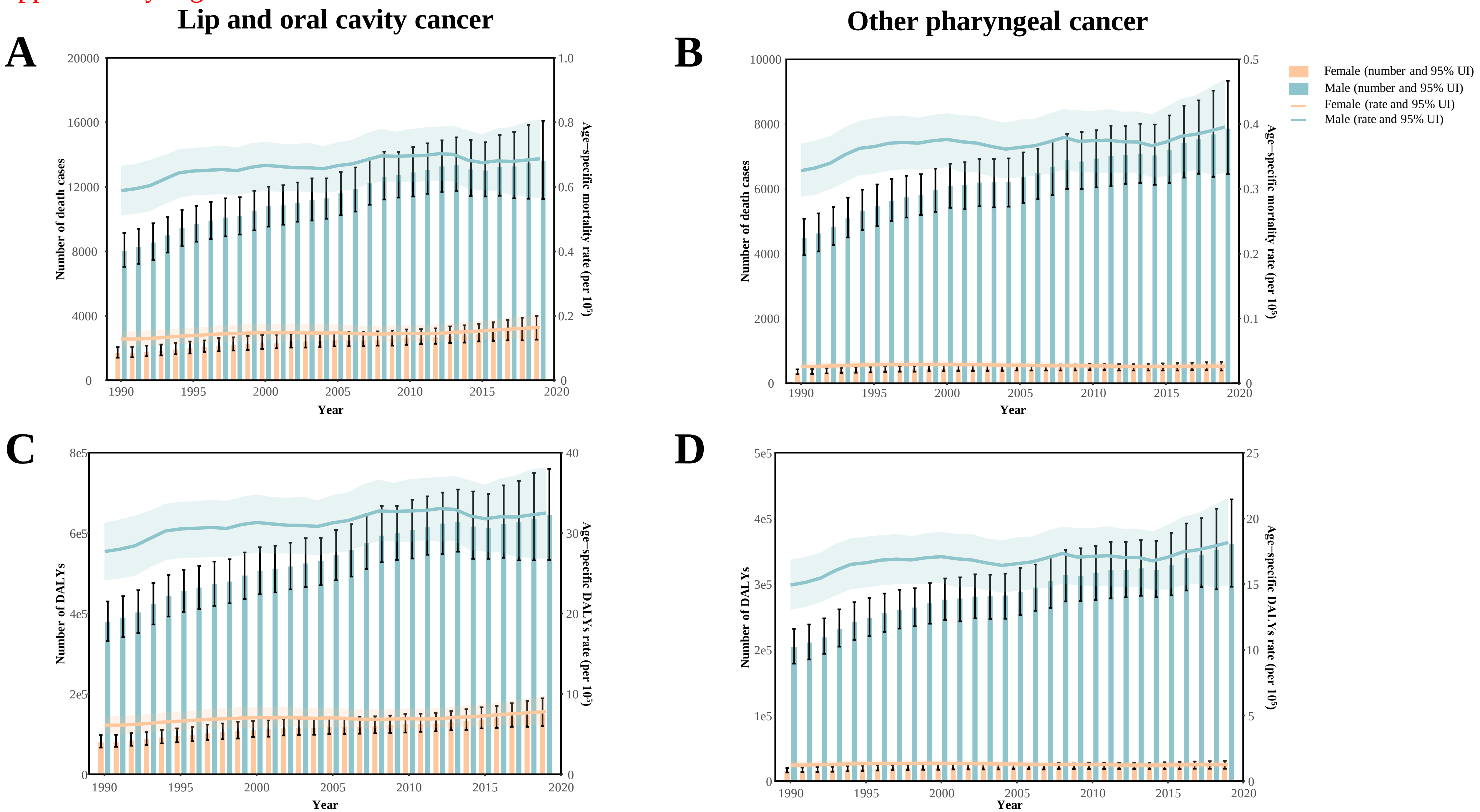

Supplementary Fig. S9

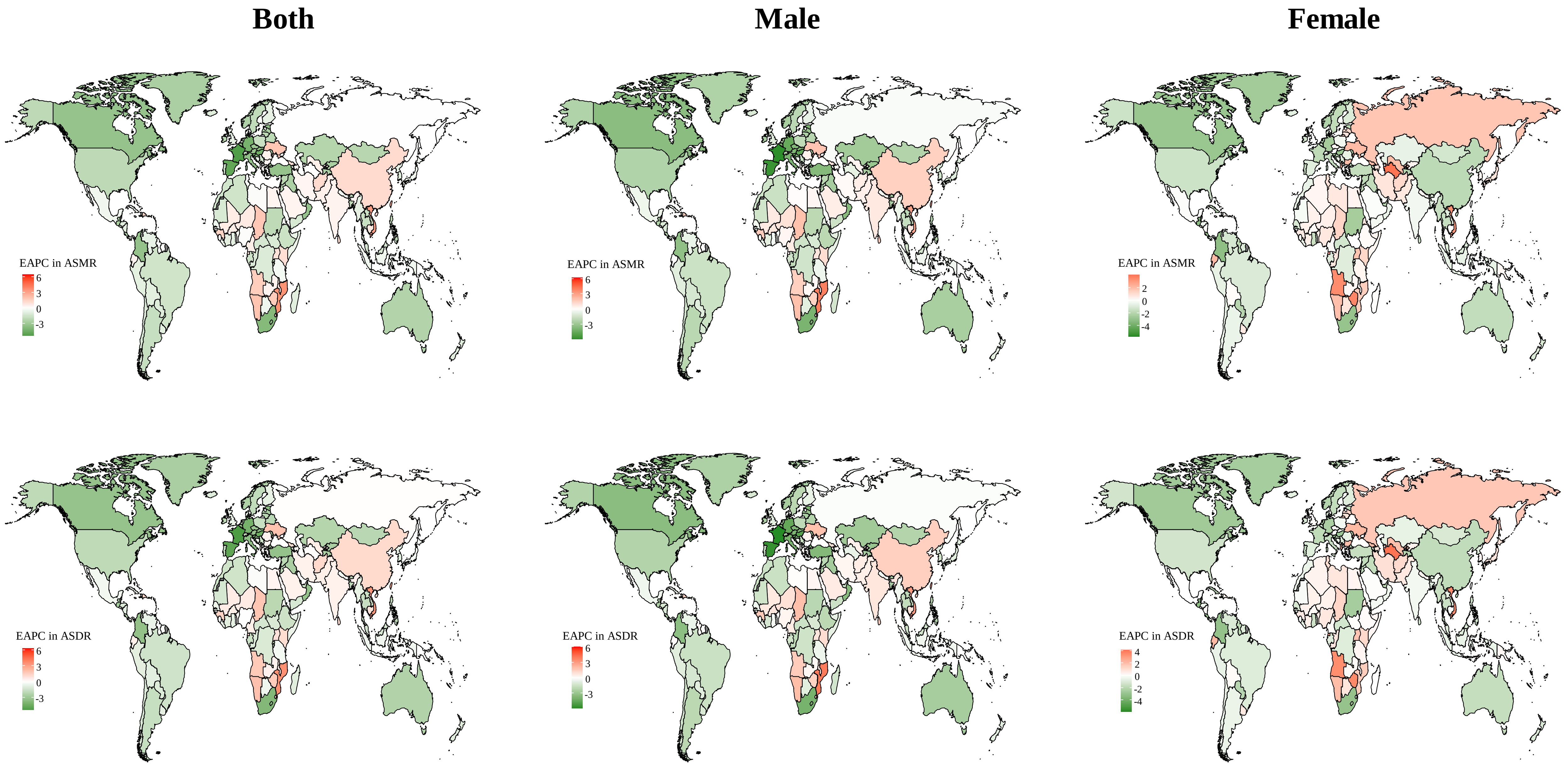

Supplementary Fig. S10

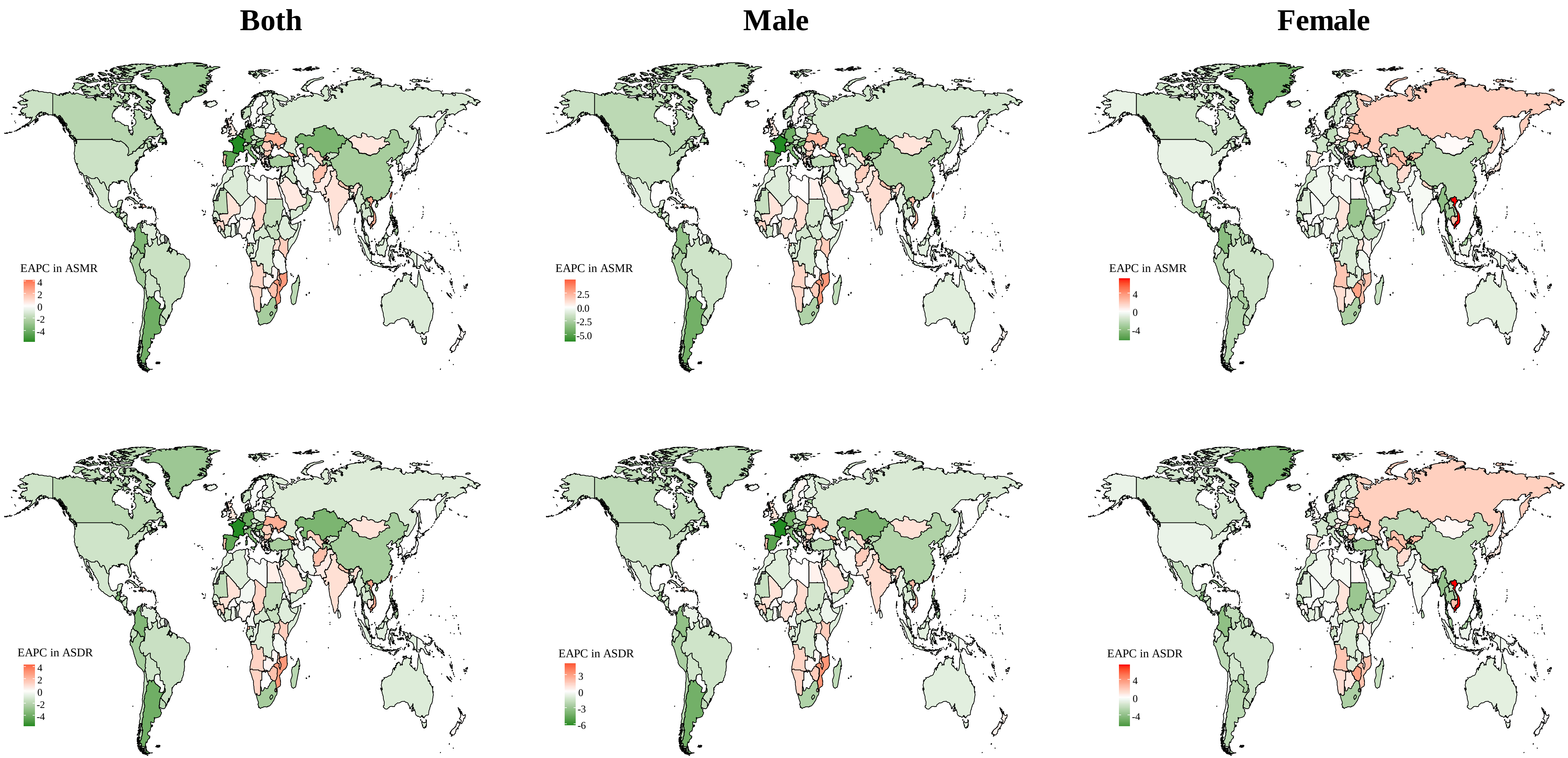

Supplementary Fig. S11

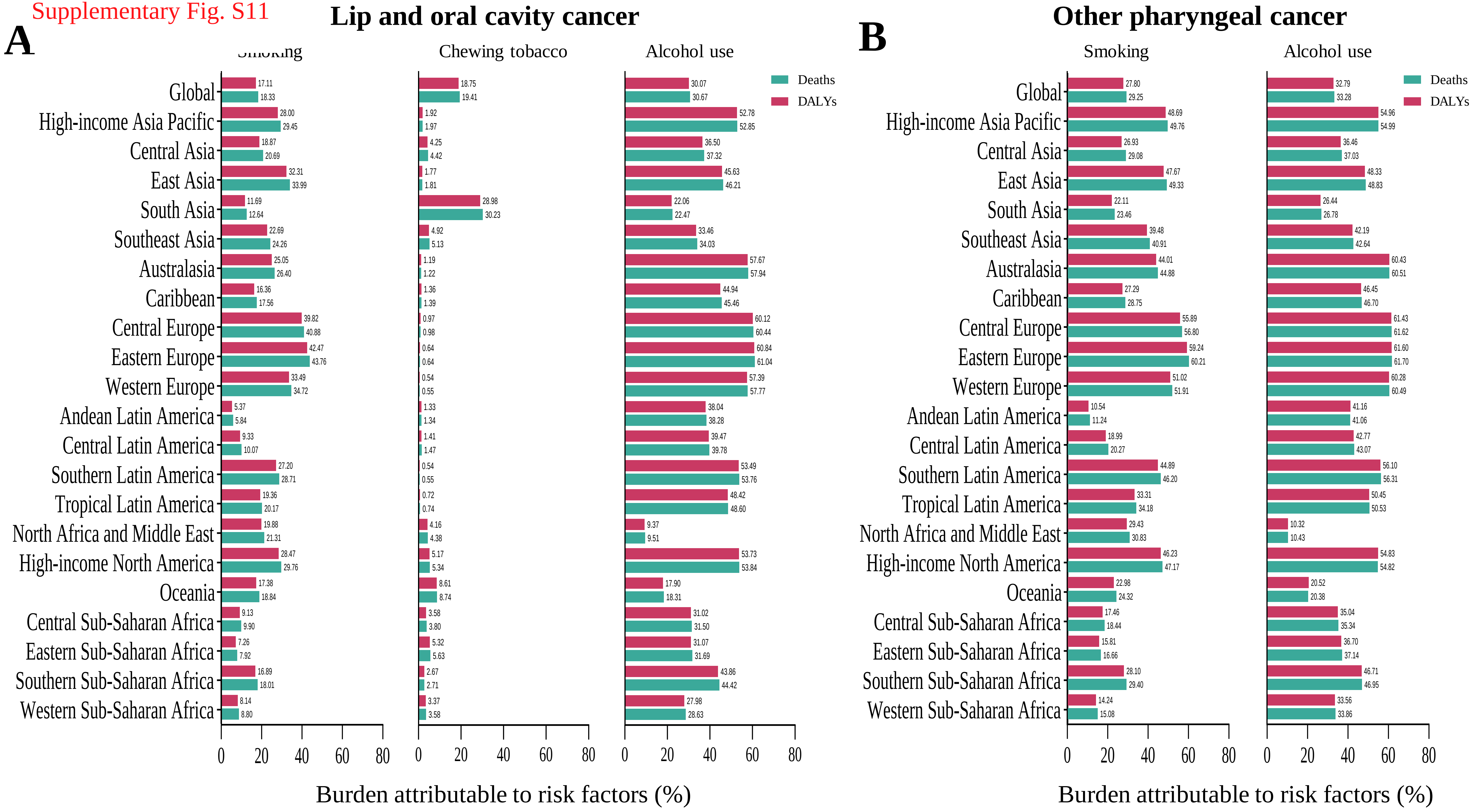

Supplementary Fig. S12

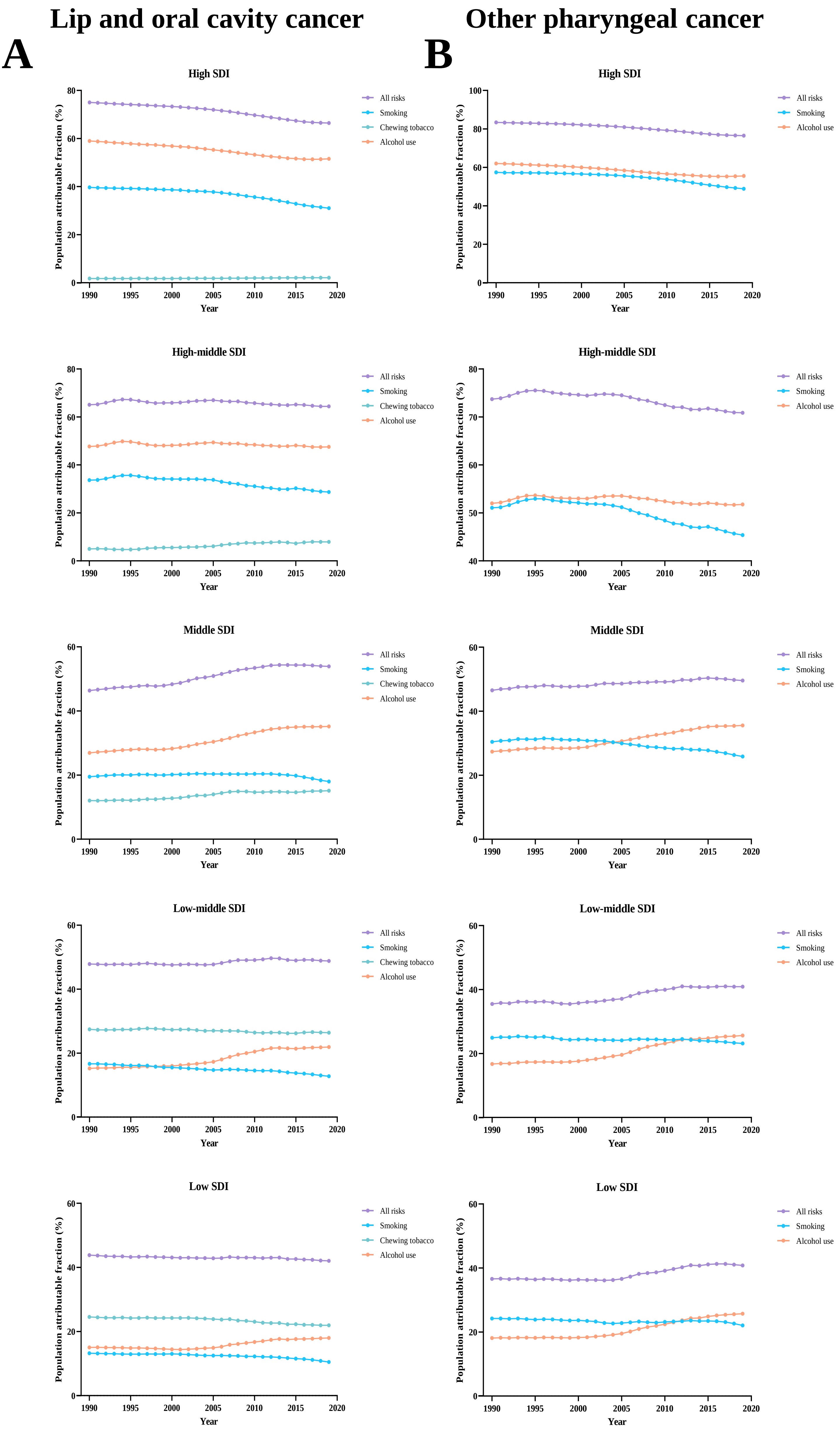

Supplementary Fig. S13

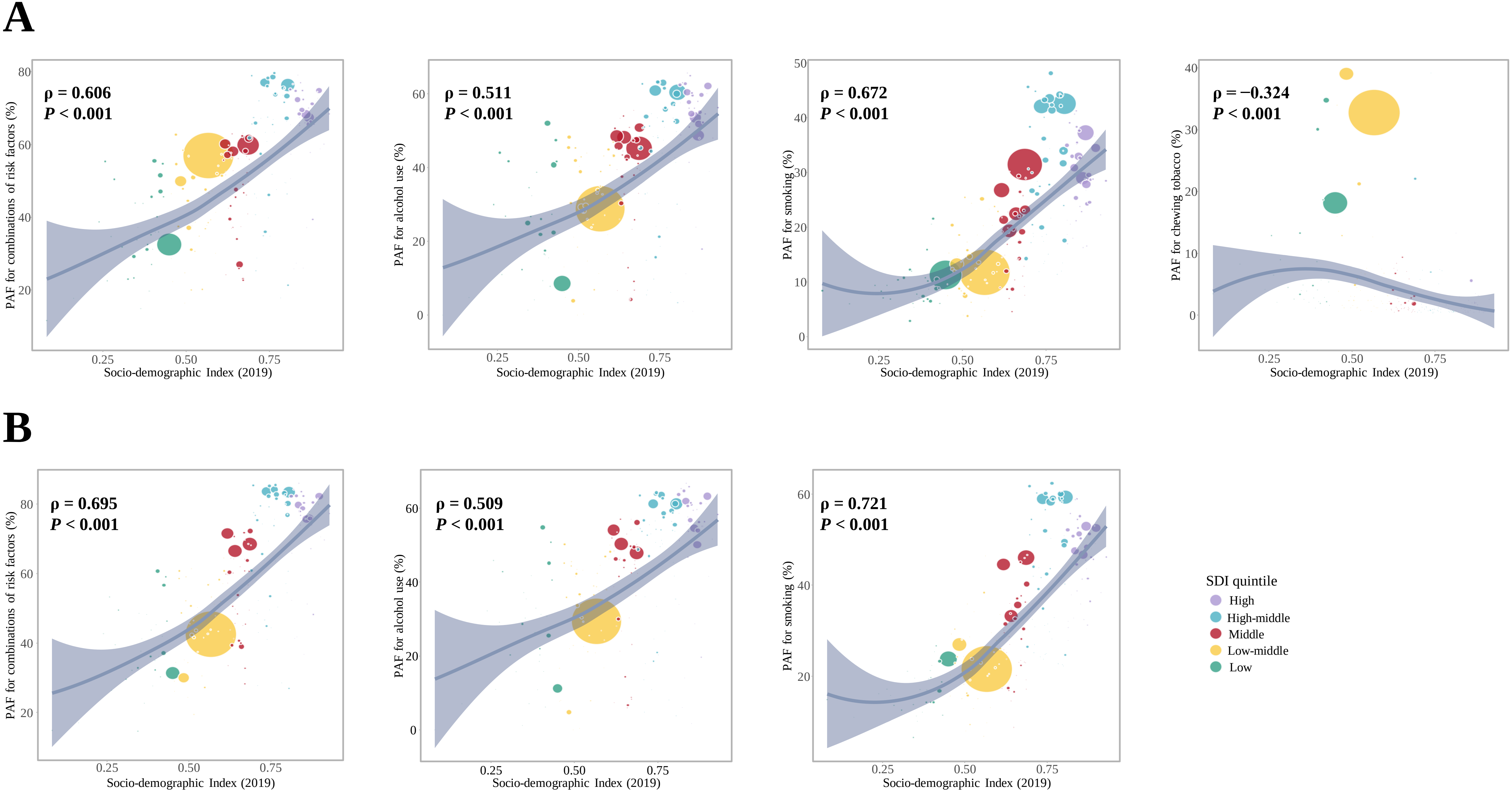

Supplementary Fig. S14

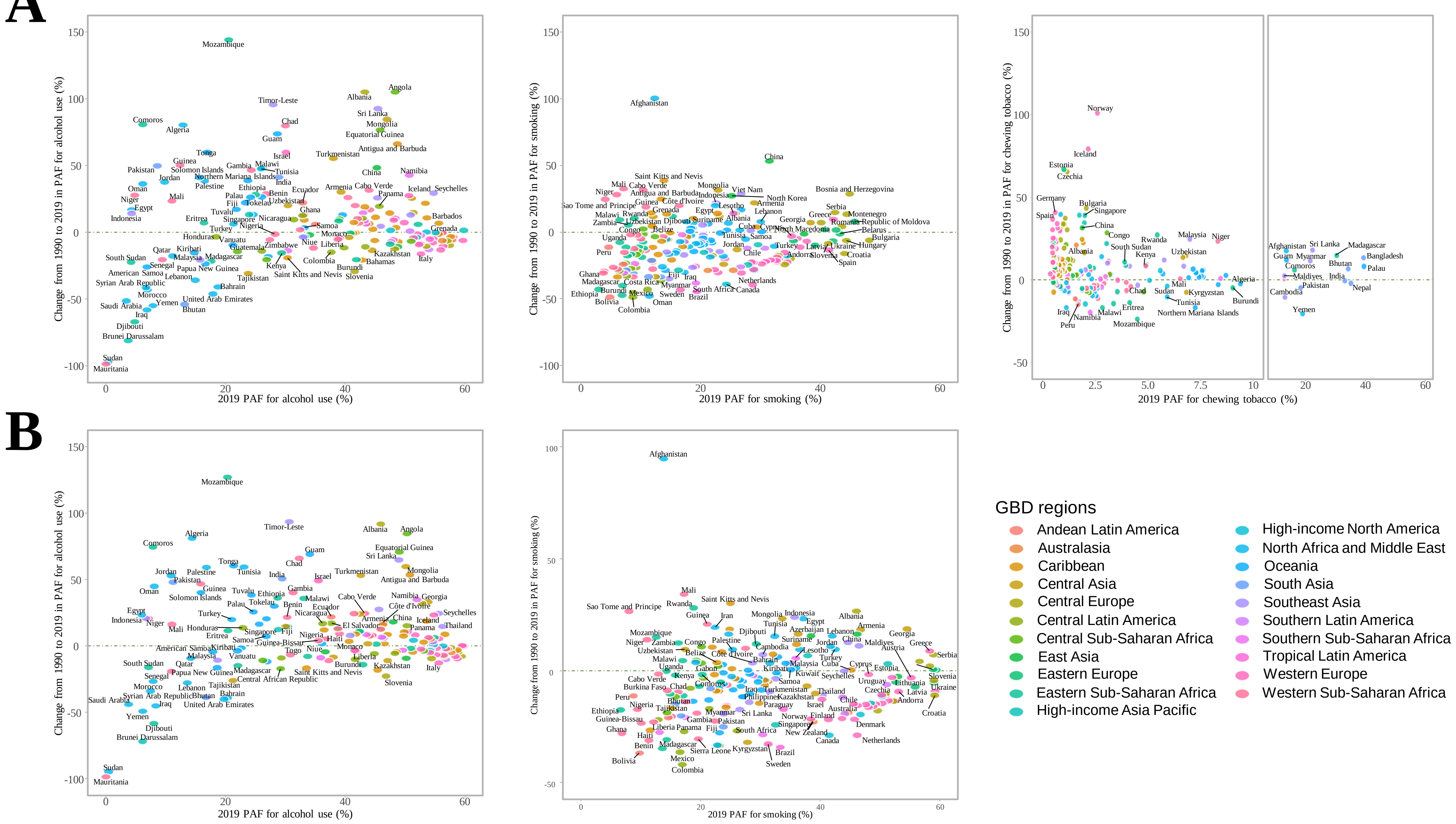

Supplementary Fig. S15

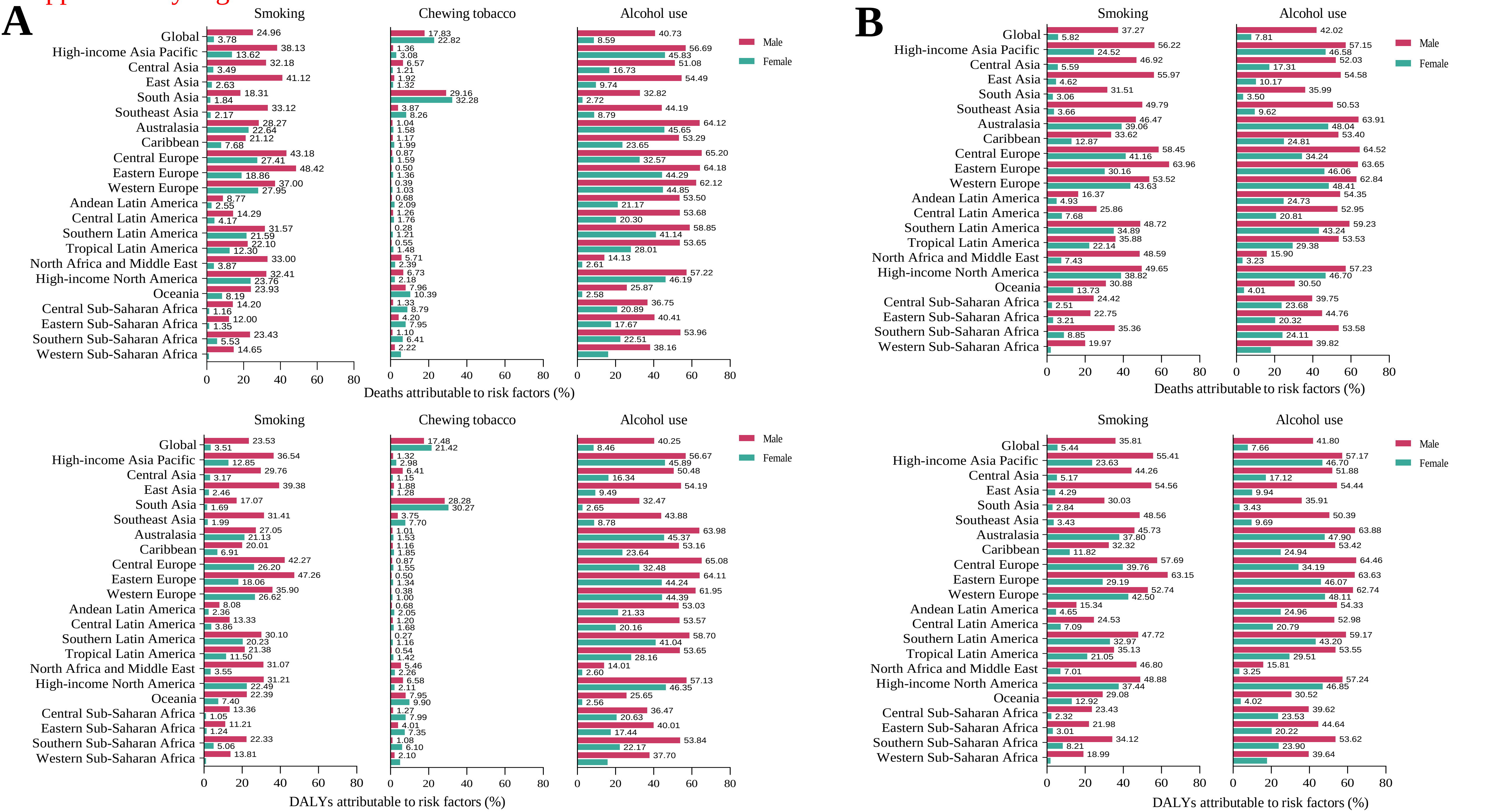

Supplementary Fig. S16

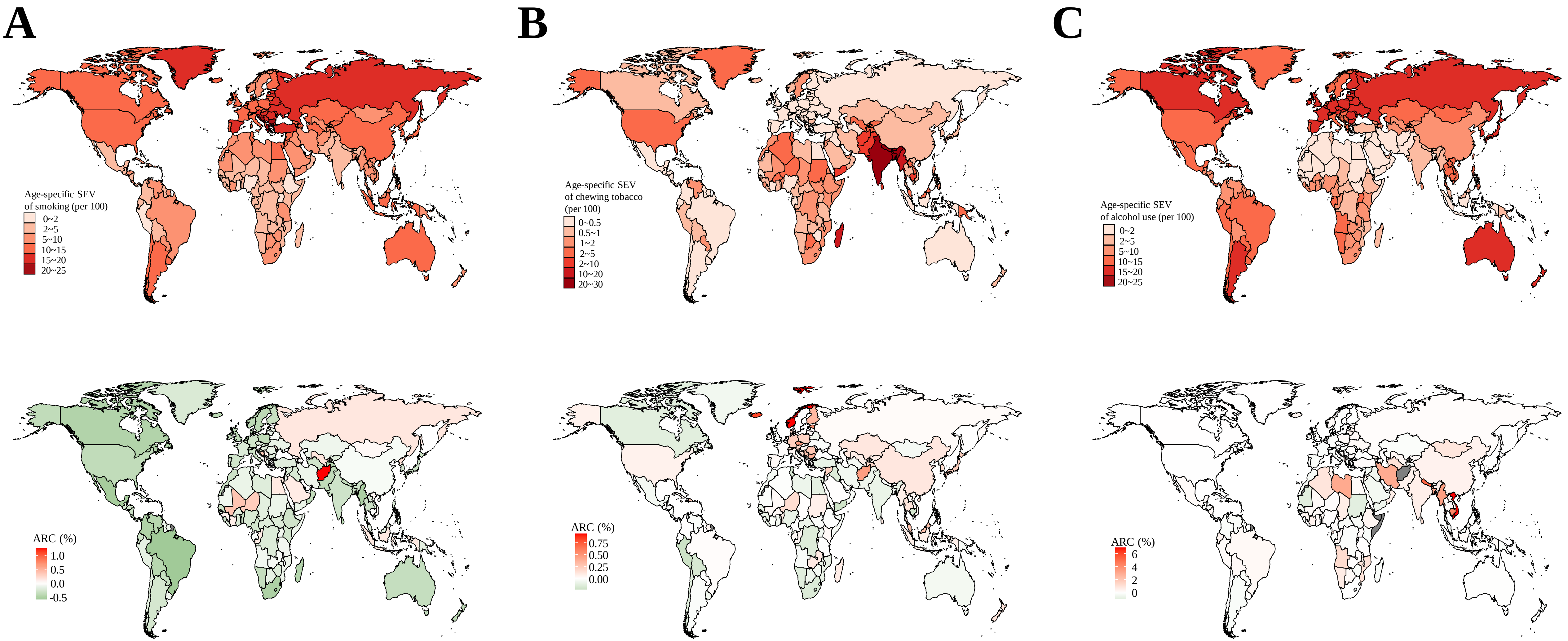

Supplementary Fig. S17

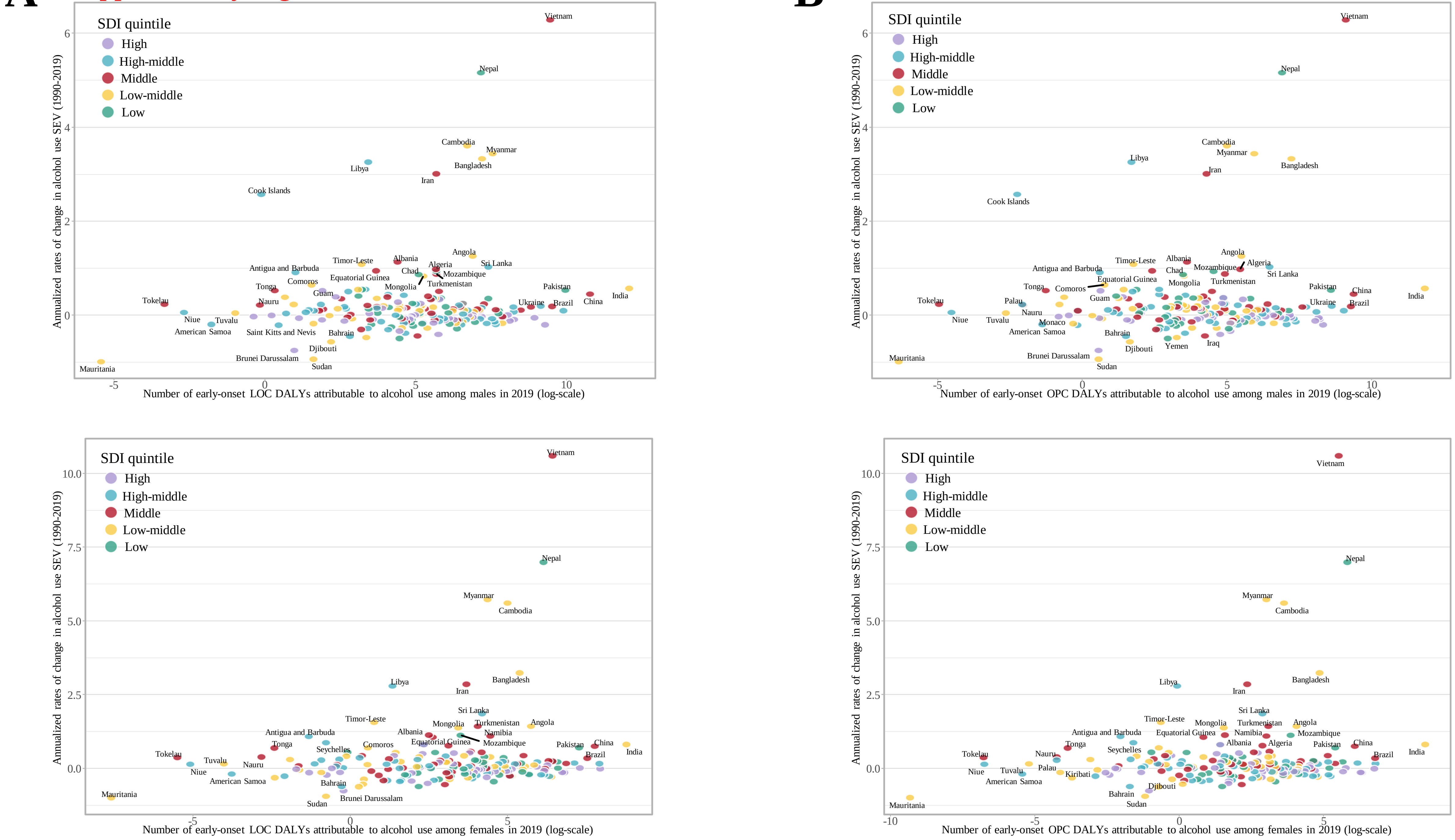

Supplementary Fig. S18

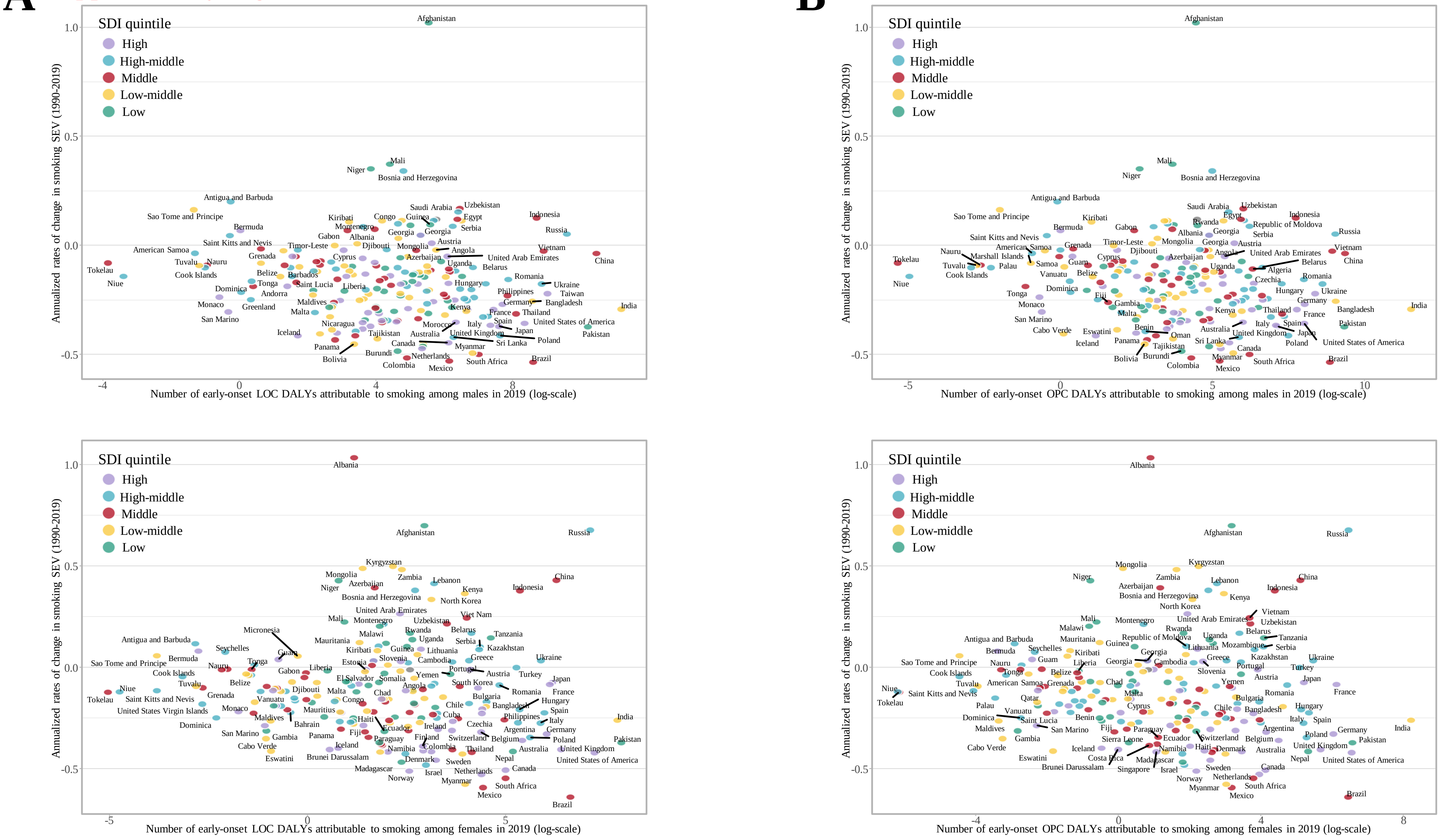

Supplementary Fig. S19

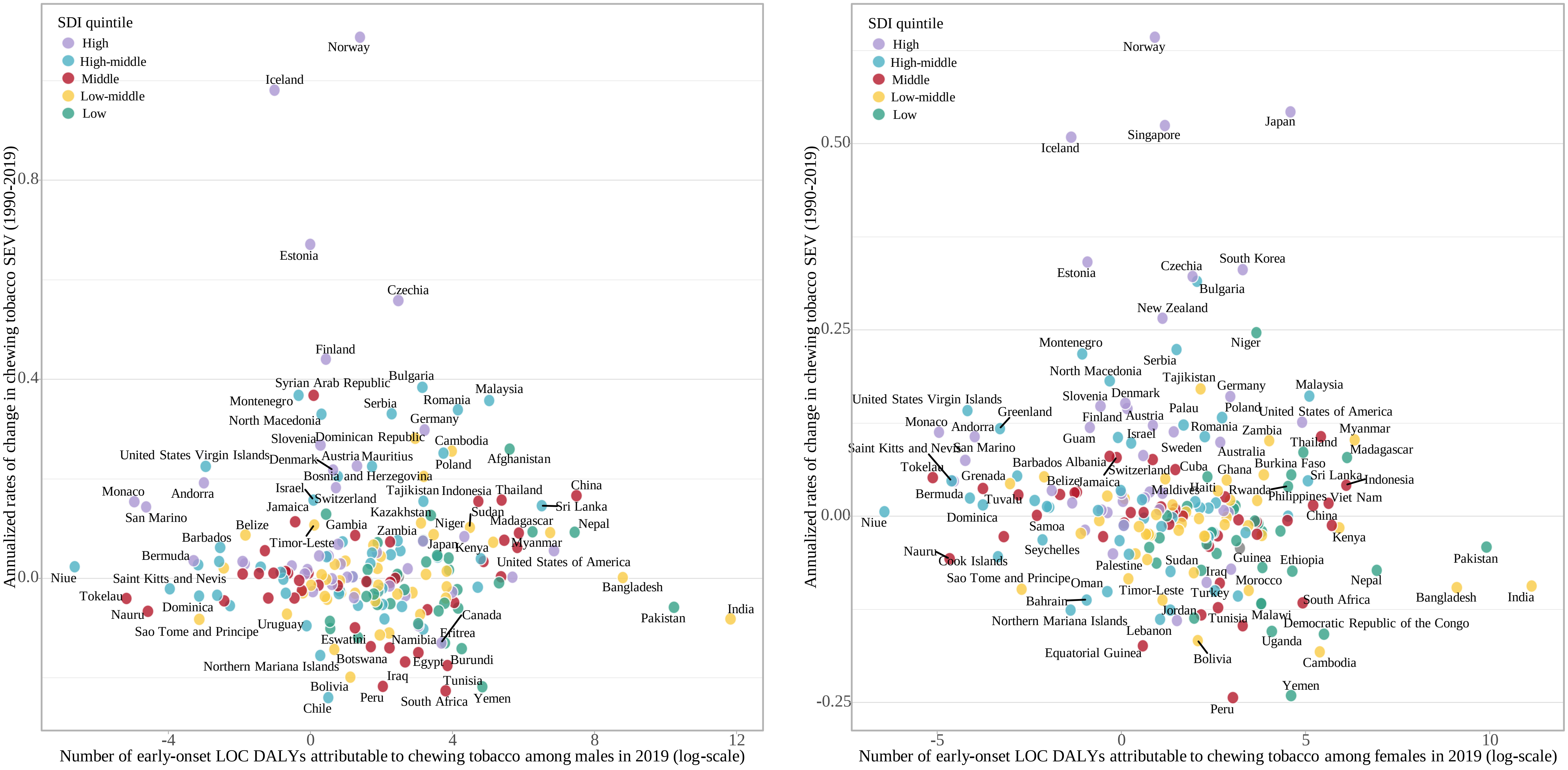

Supplementary Fig. S20

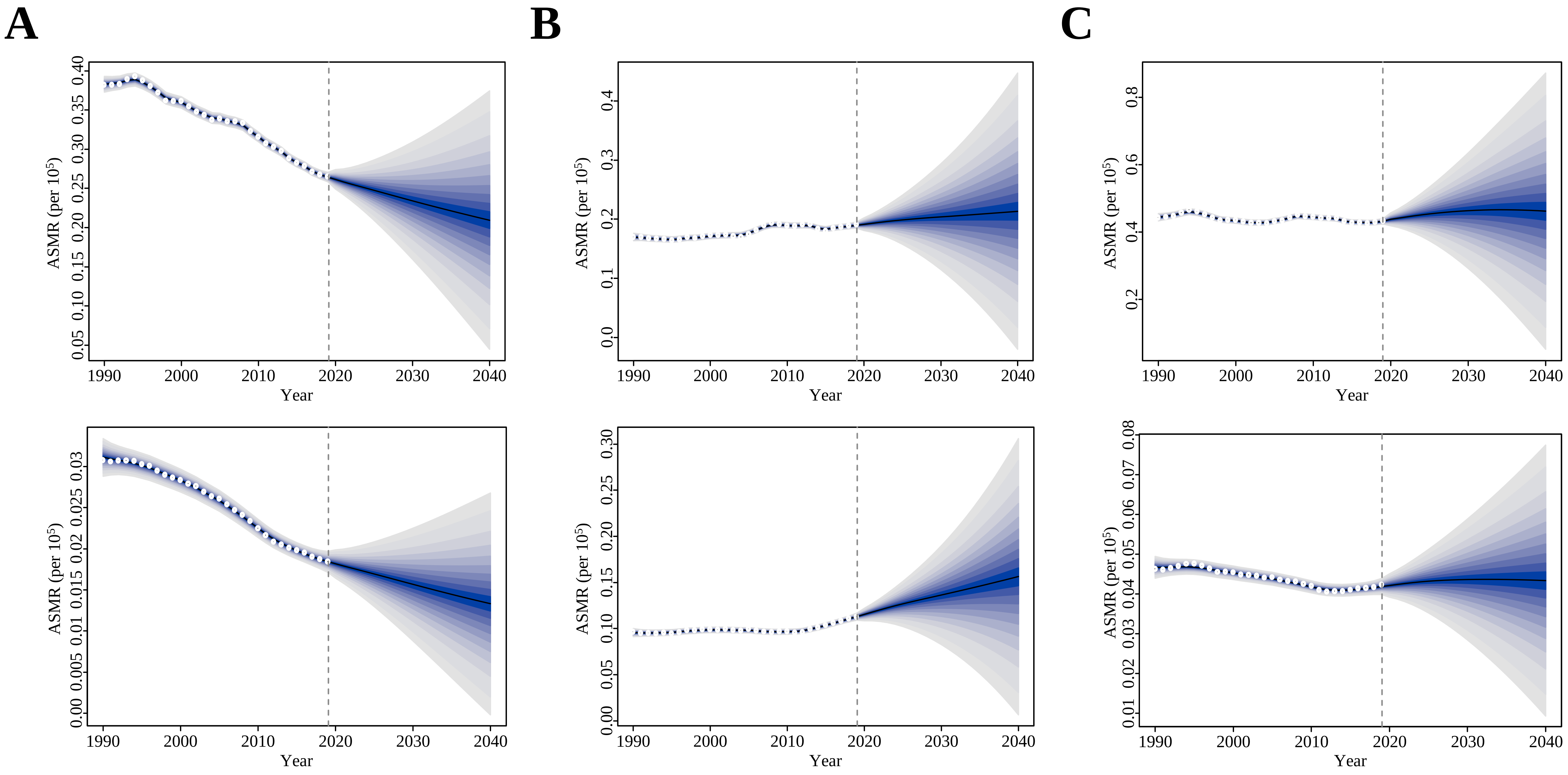

Supplementary Fig. S21

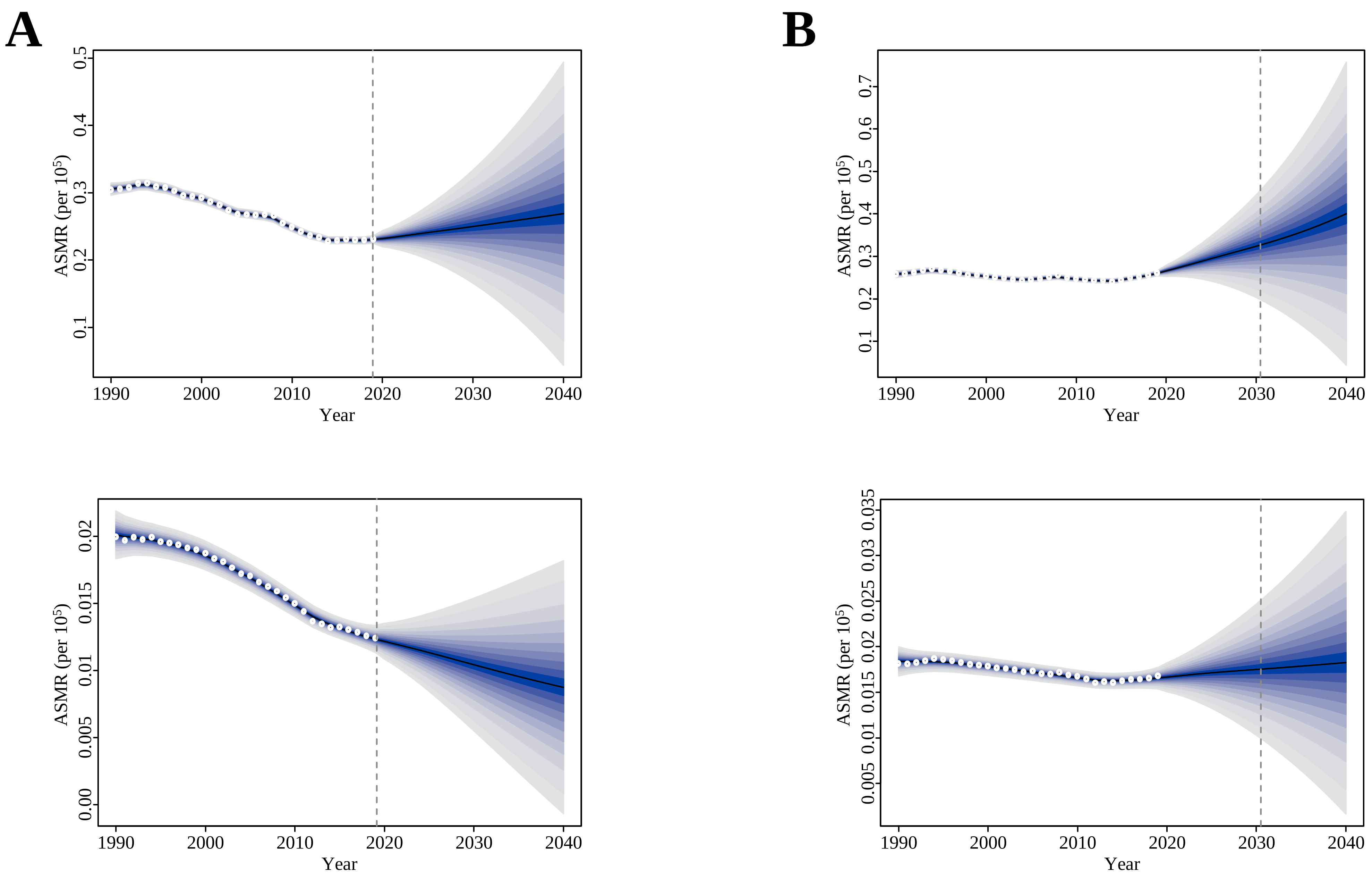

Supplementary Fig. S22

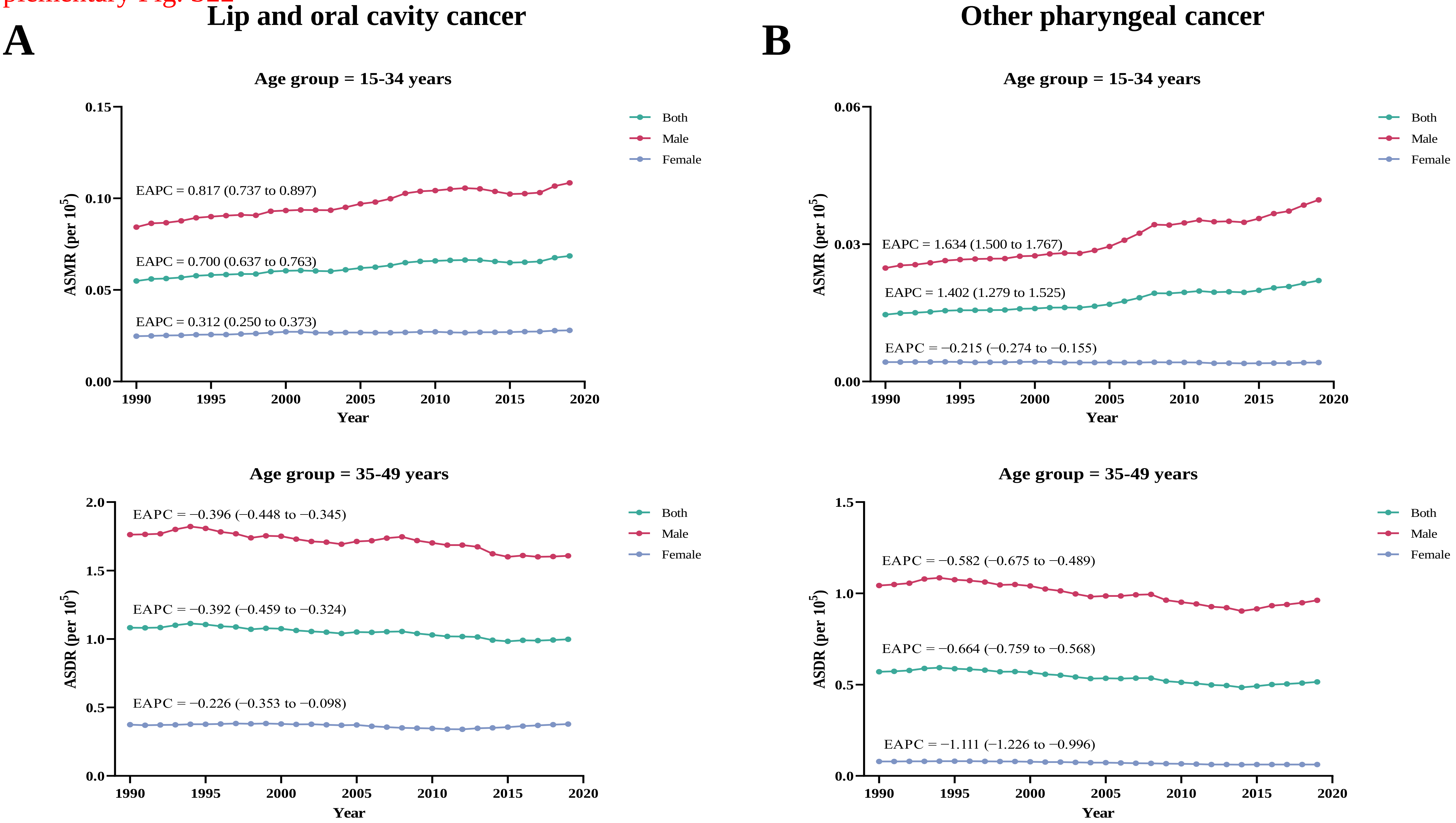

Supplementary Fig. S23

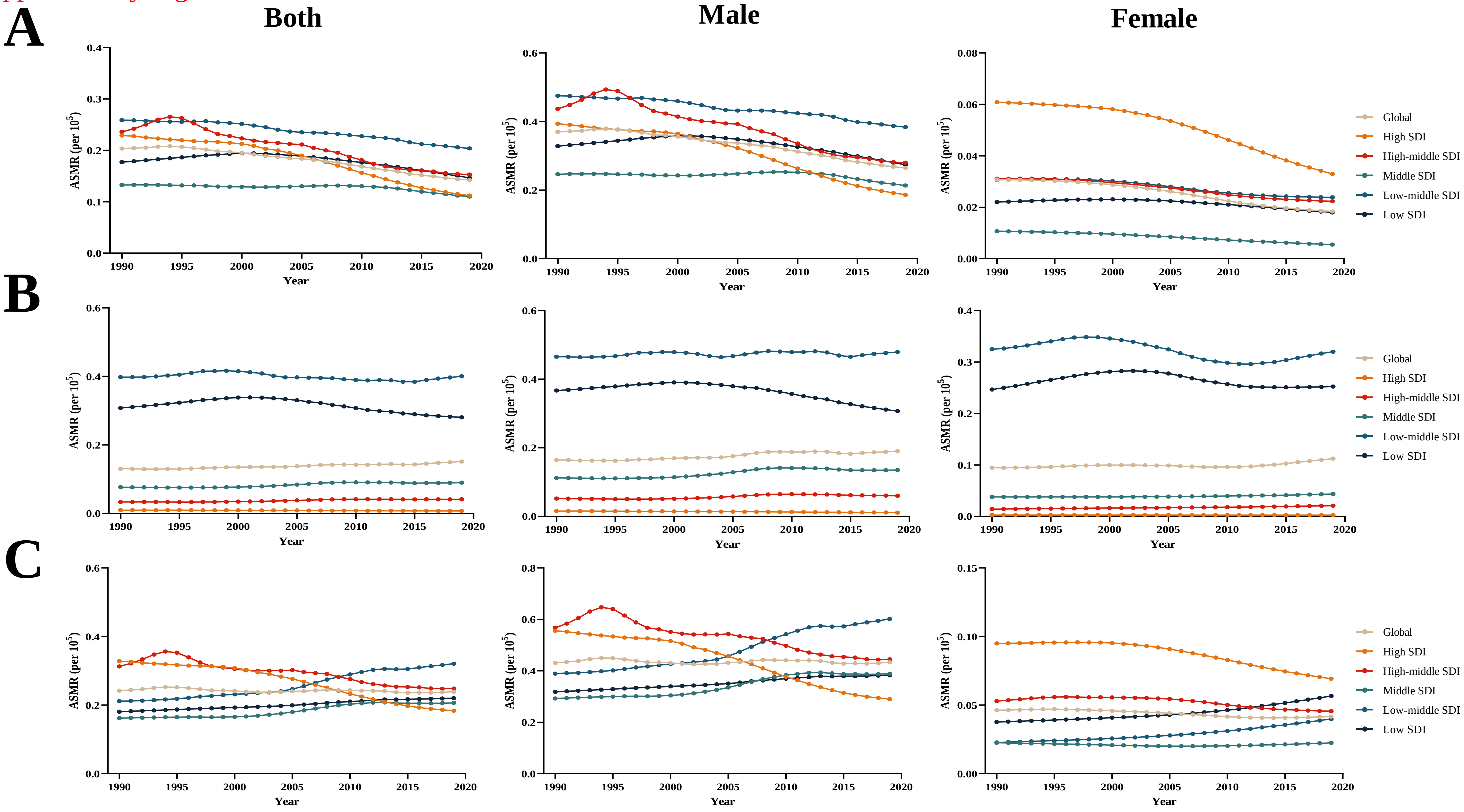

# Supplementary Fig. S24

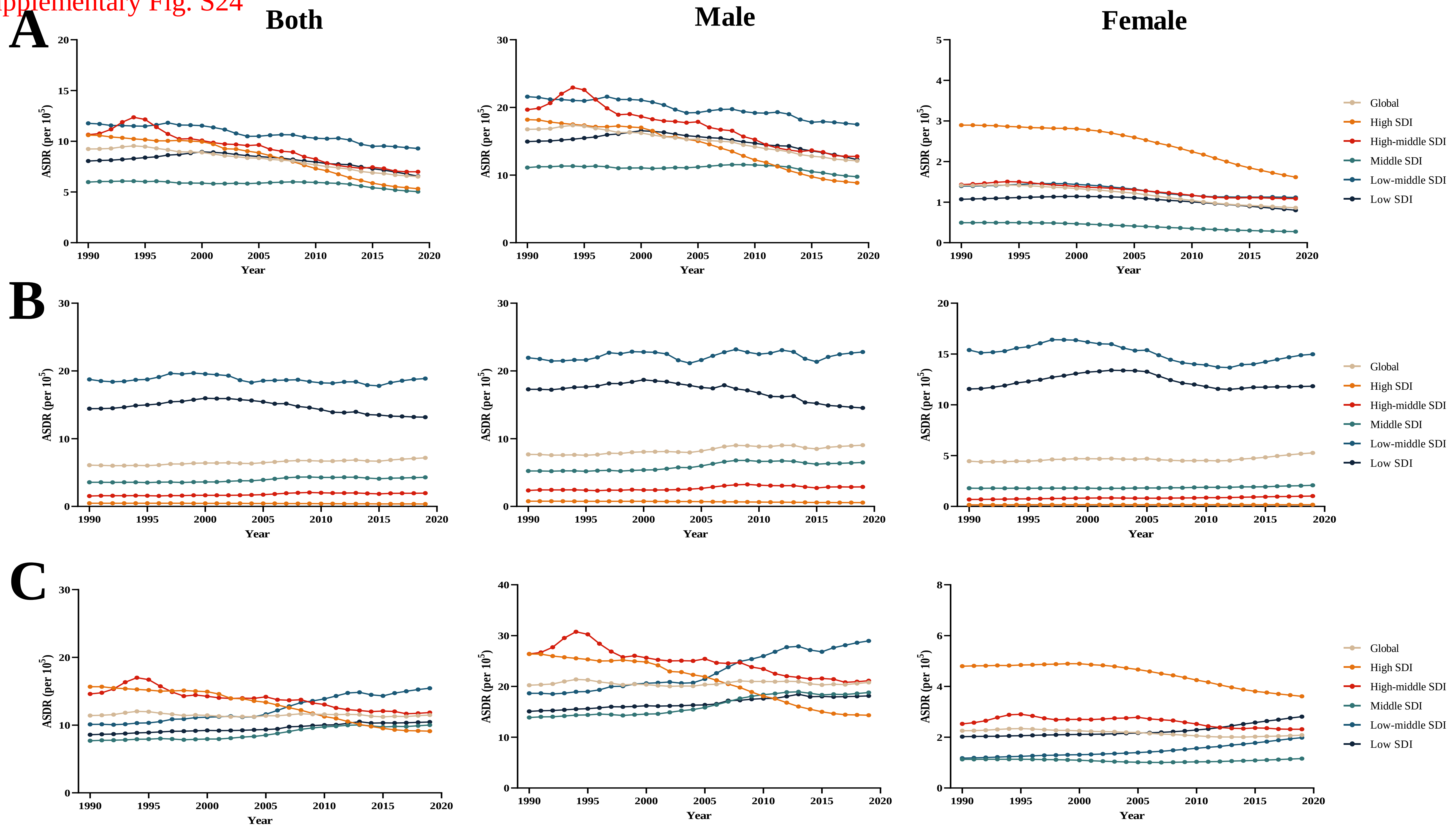

Supplementary Fig. S25

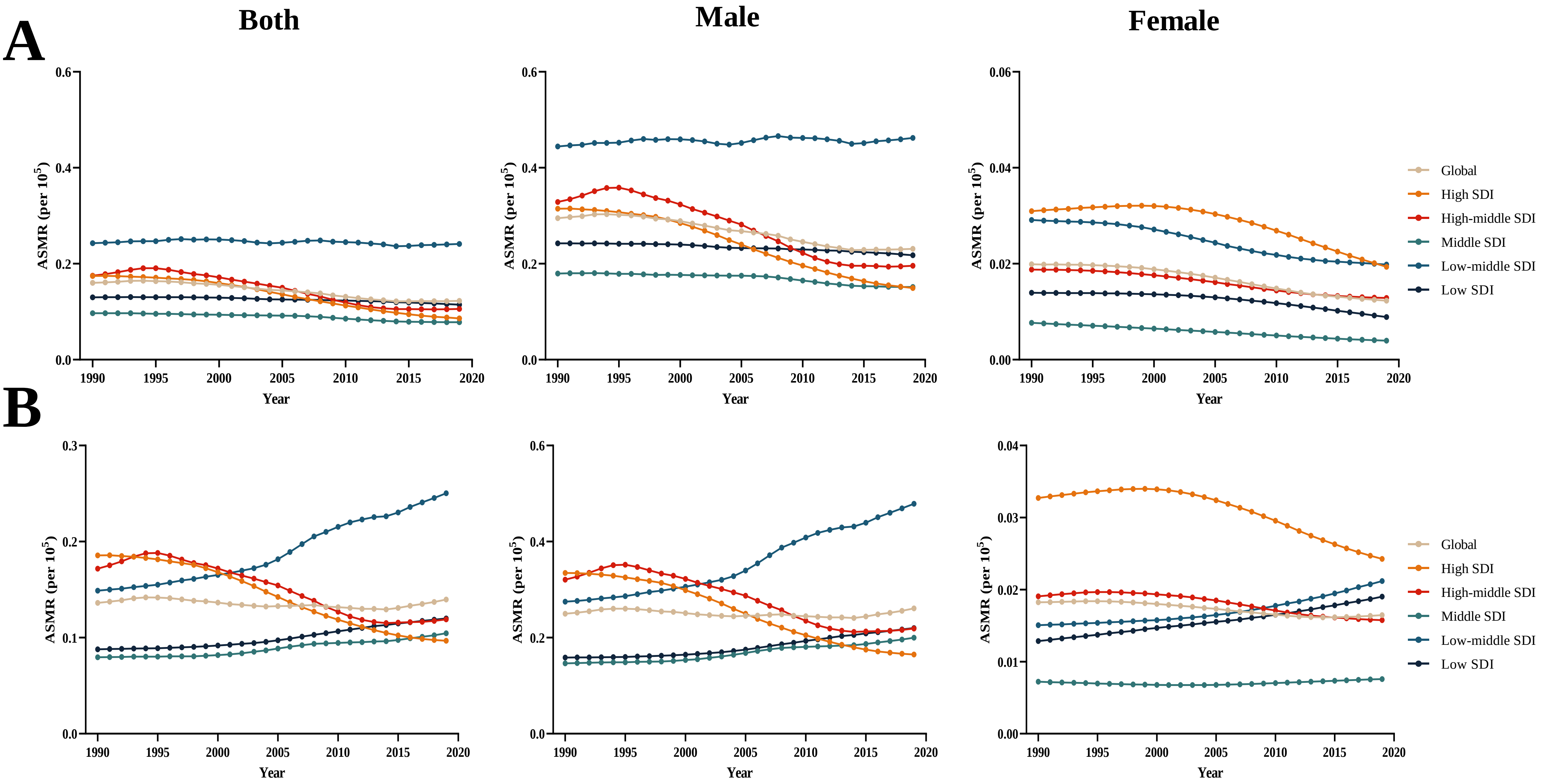

Supplementary Fig. S26

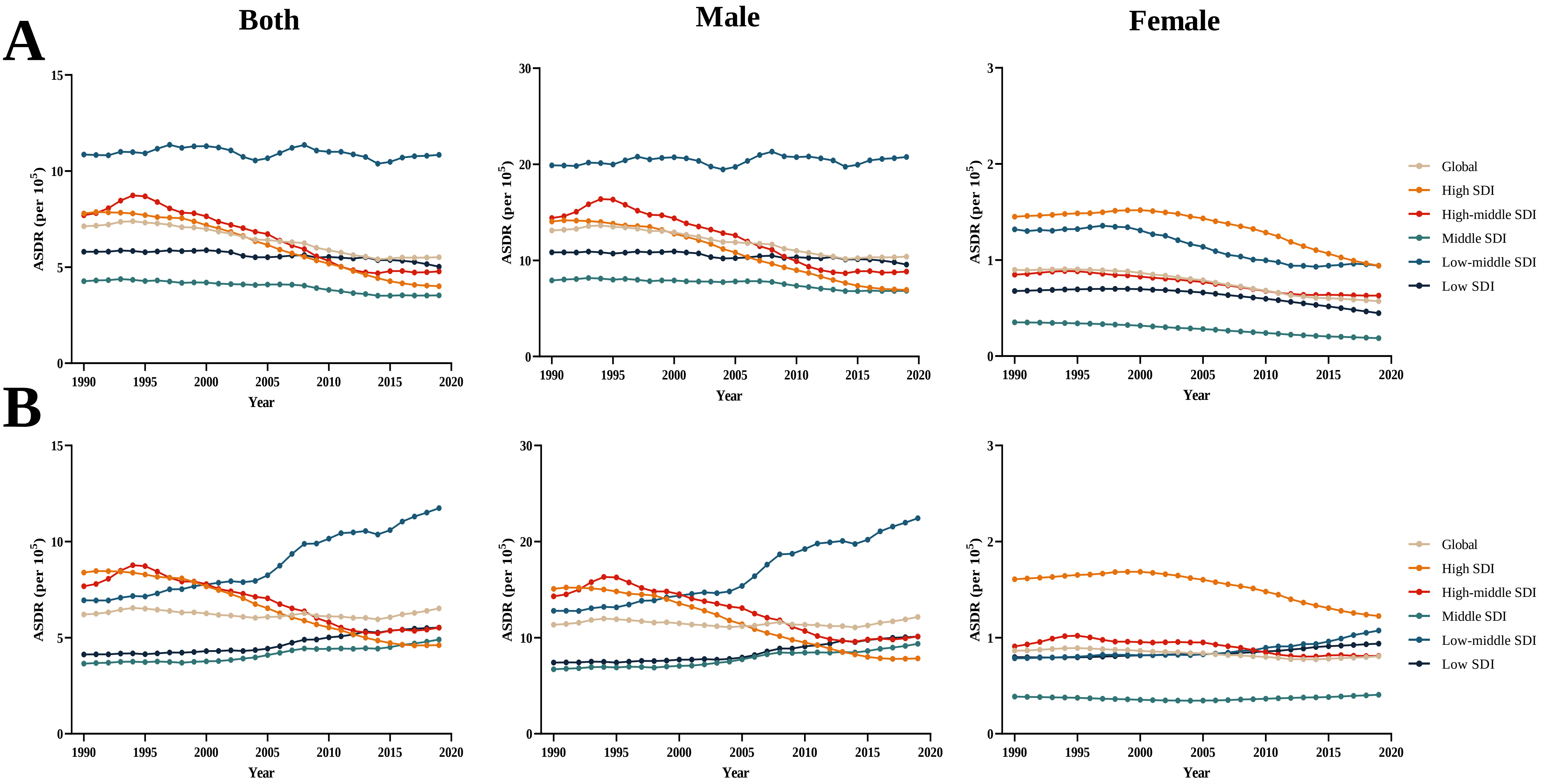

Supplementary Fig. S27

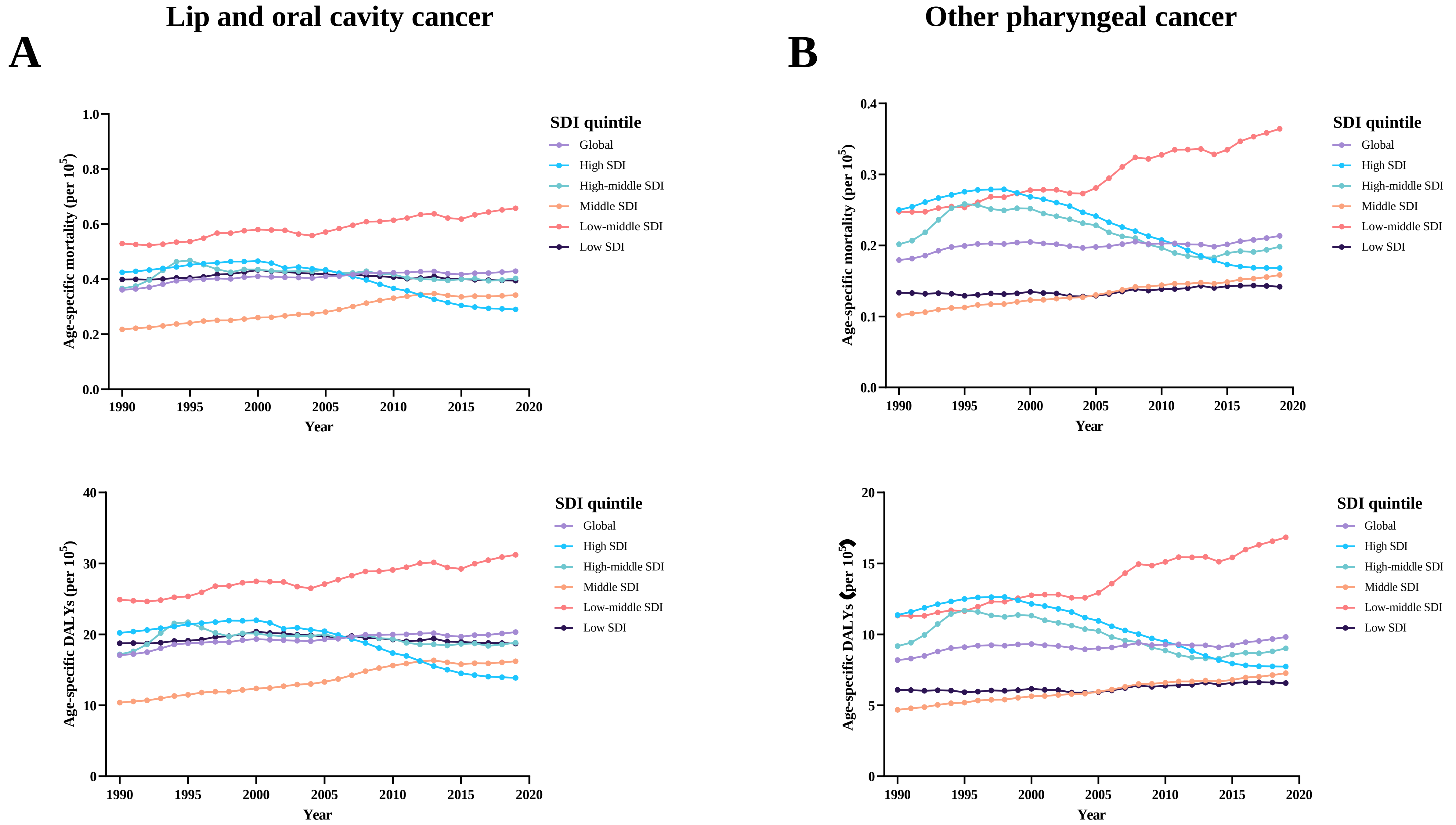

Supplementary Fig. S28

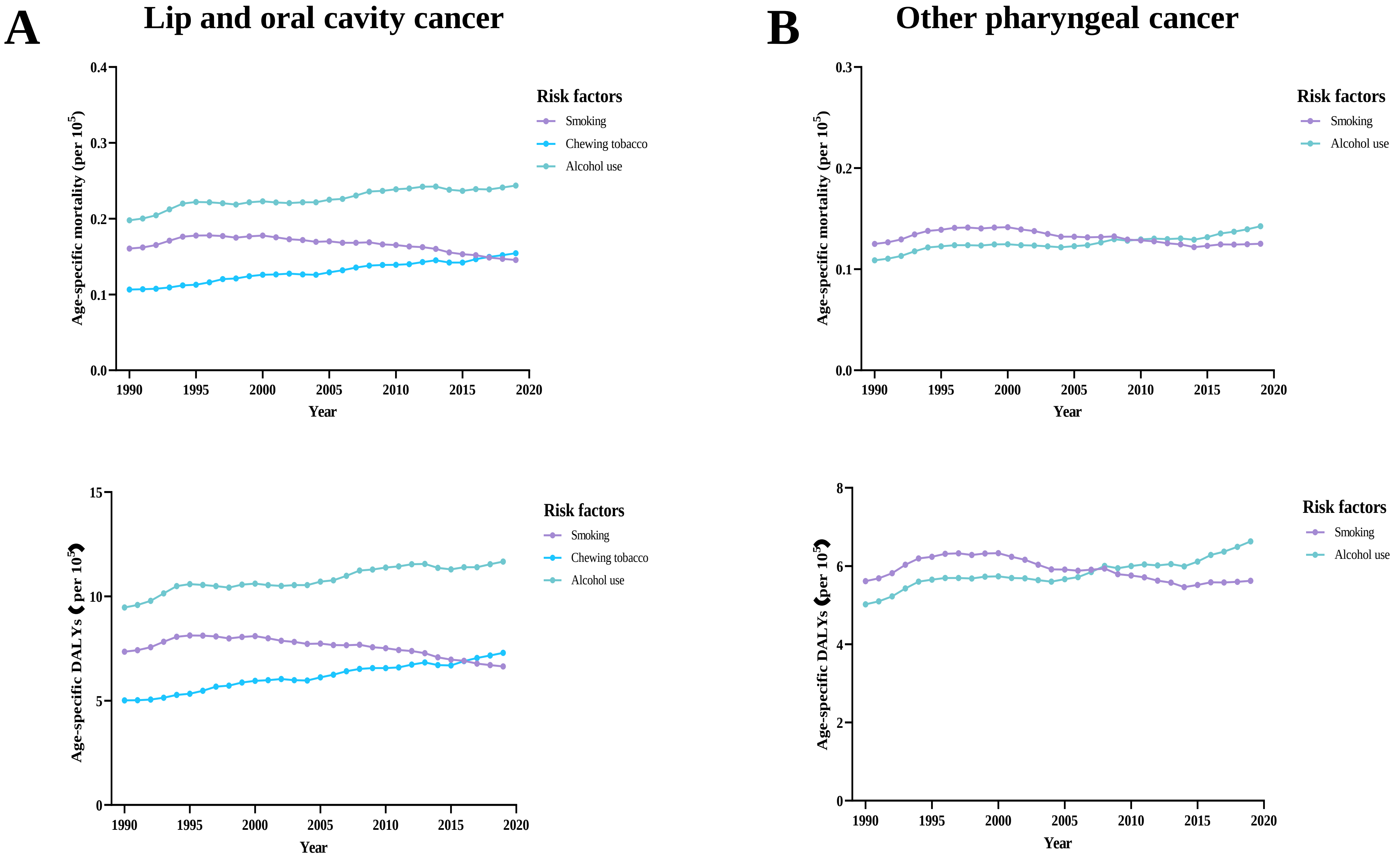

Supplementary Fig. S29

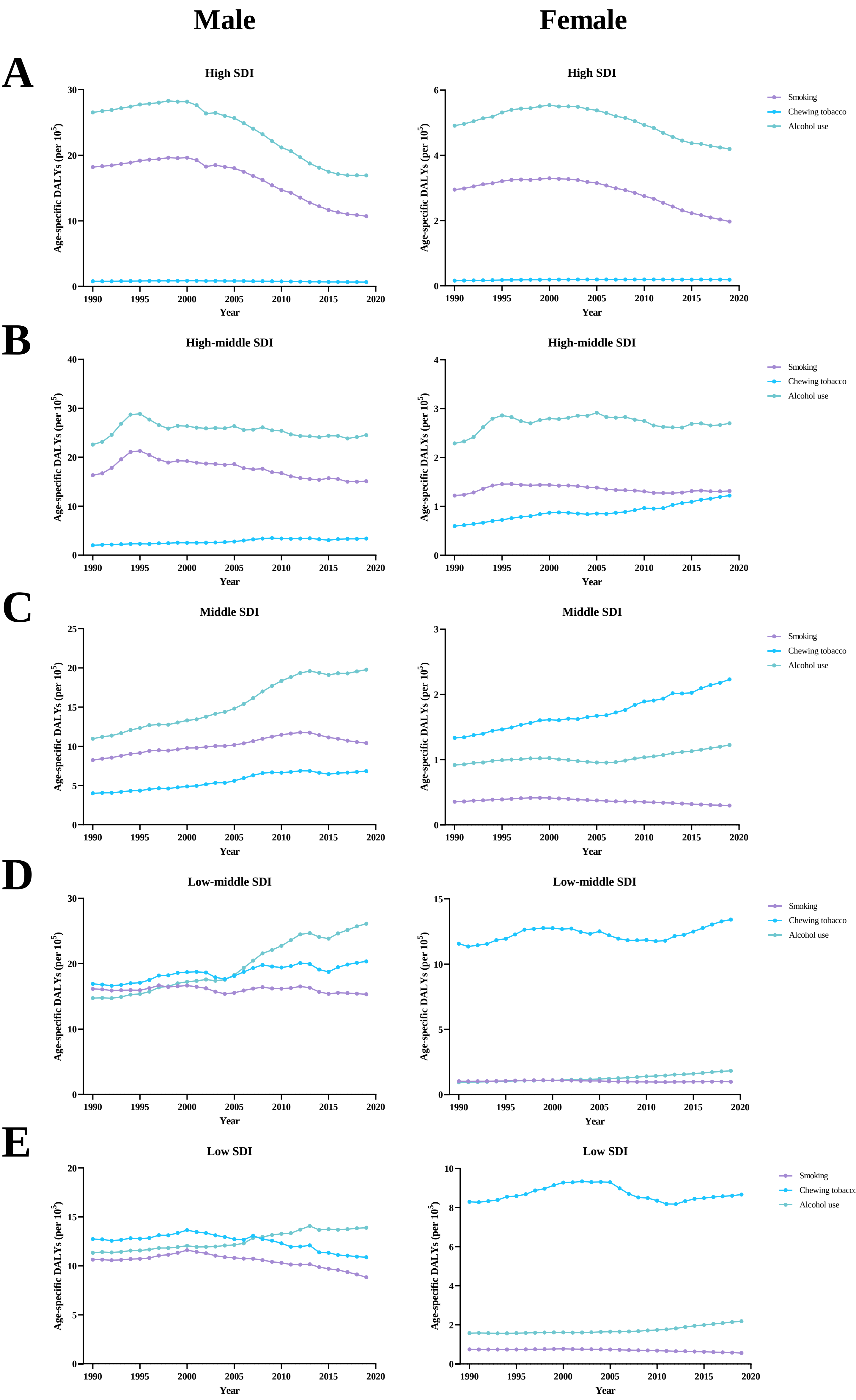

Supplementary Fig. S30

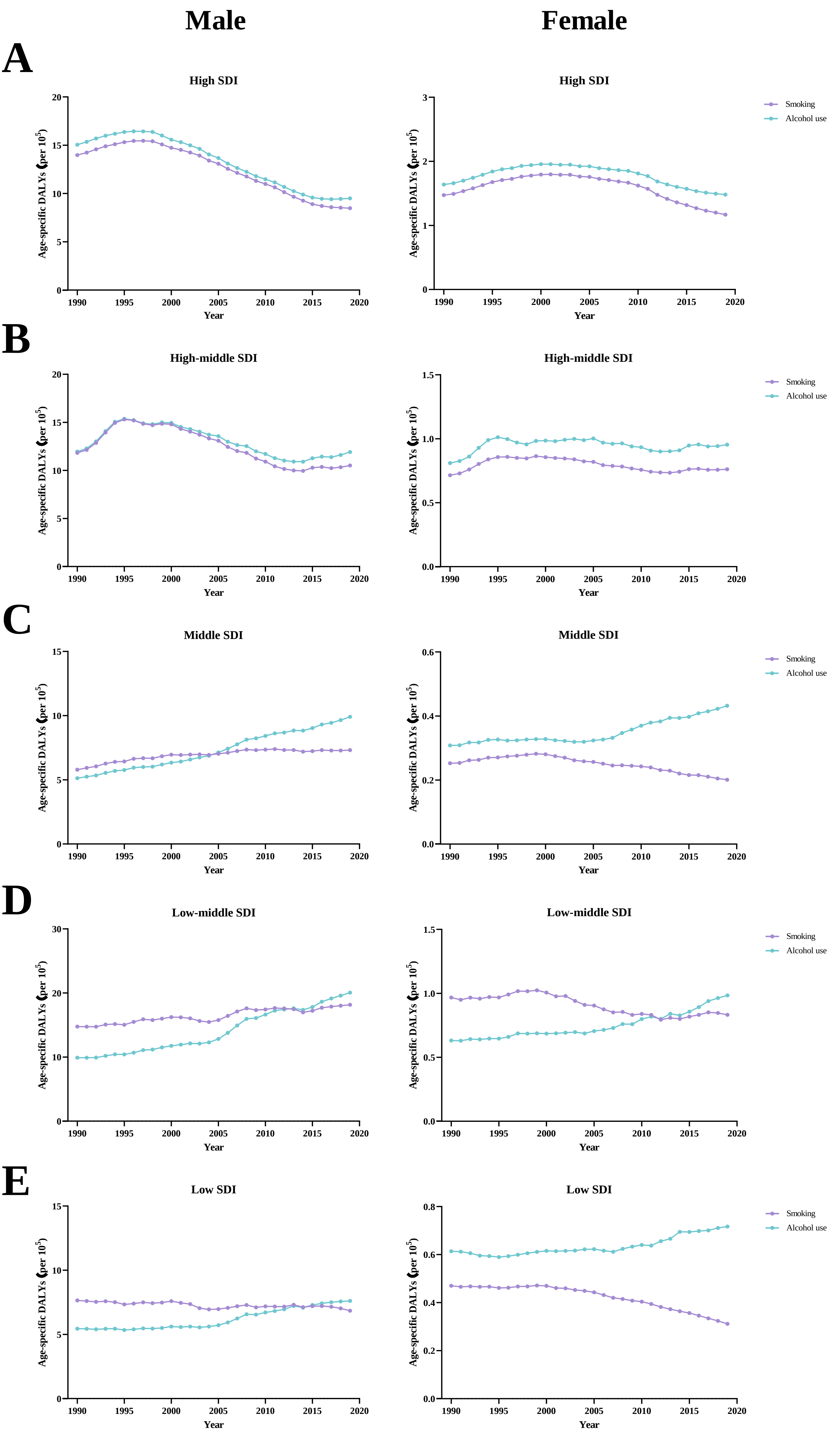

Supplementary Fig. S31

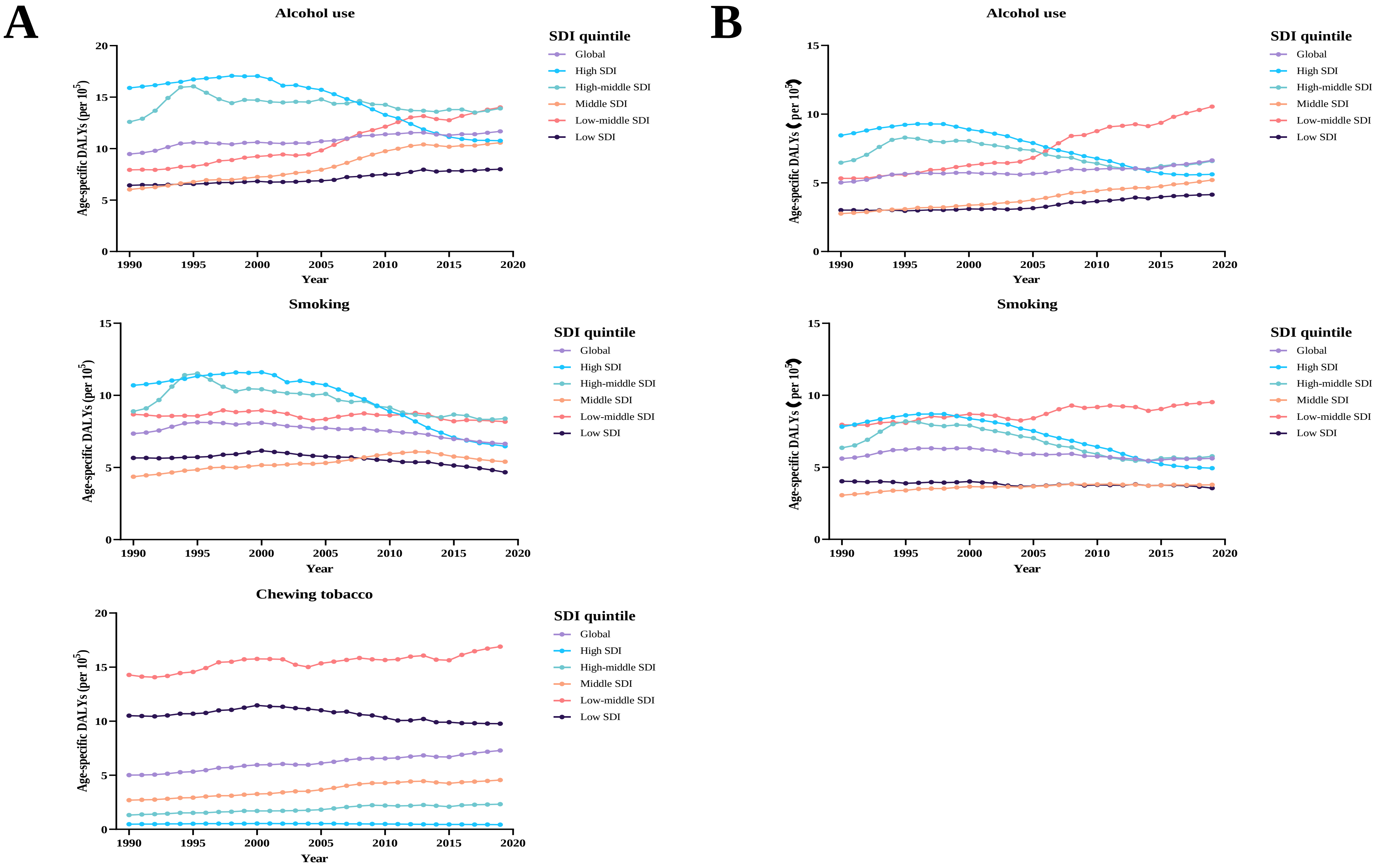

Supplementary Fig. S32

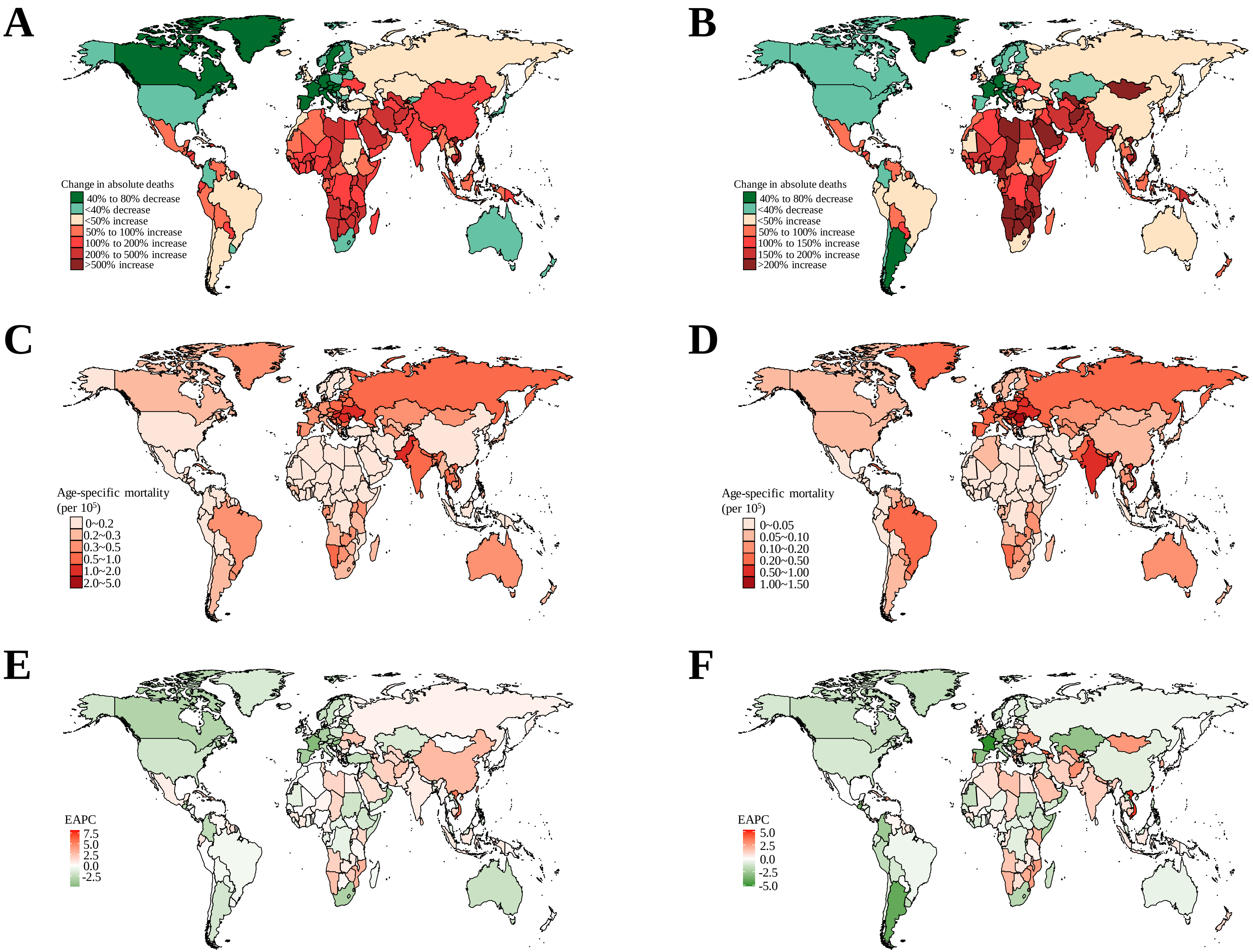

Supplementary Fig. S33

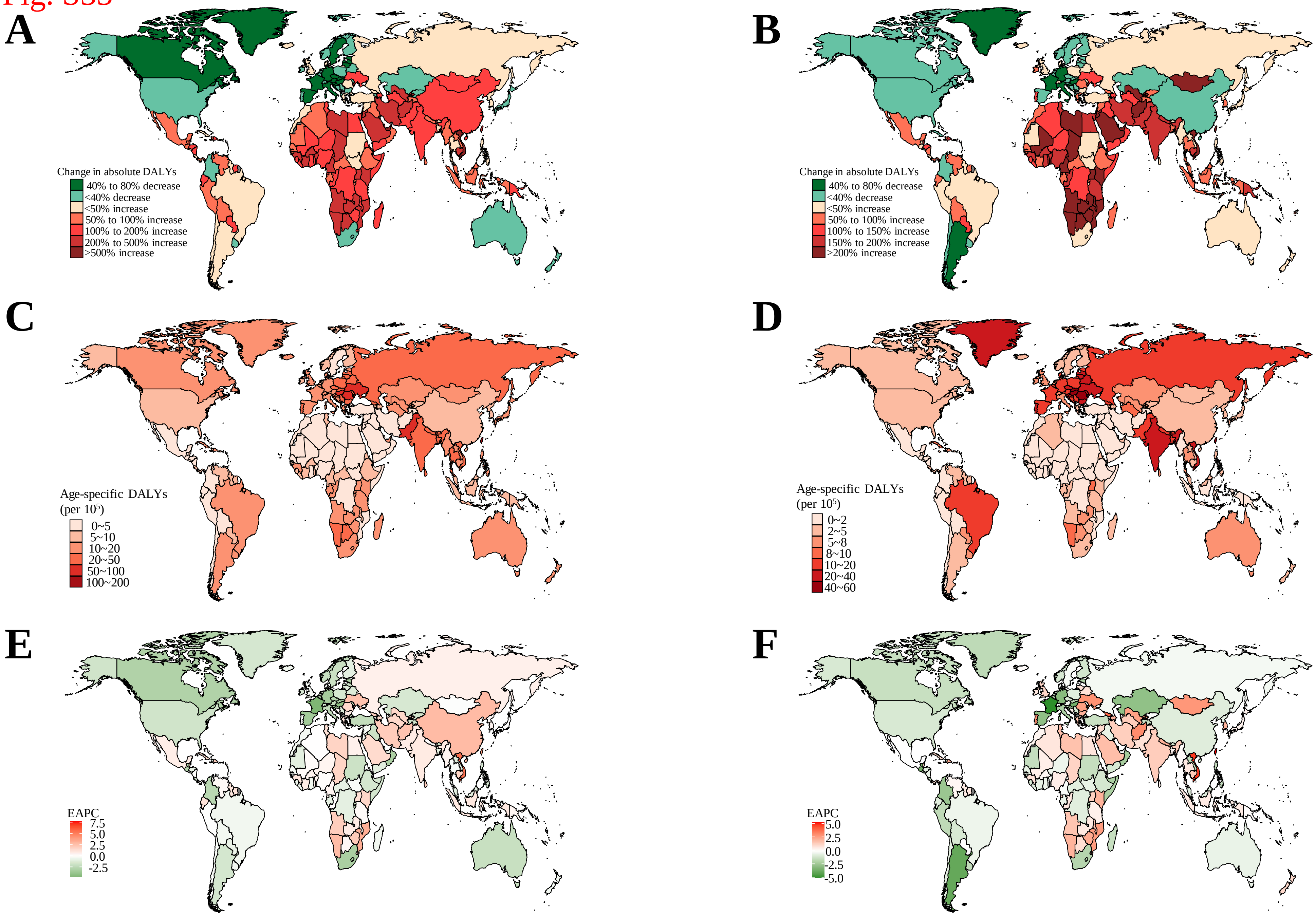

Supplement: Supplementary file 1 [file DataSheet1.pdf]
